# Designing the DNA-Intercalating Moiety to Improve the Safety and Efficacy of Novel Bacterial Topoisomerase Inhibitors

**DOI:** 10.1021/acs.jmedchem.6c00415

**Published:** 2026-07-27

**Authors:** Maša Zorman, Živa Zajec, Irena Zdovc, Lidija Senerovic, Natasa Radakovic, Marija Atanaskovic, Marko Anderluh, Nikola Minovski, Martina Hrast Rambaher

**Affiliations:** † Theory Department, Laboratory for Cheminformatics, 68913 National Institute of Chemistry, Hajdrihova 19, Ljubljana 1001, Slovenia; ‡ Department of Pharmaceutical Chemistry, Faculty of Pharmacy, 63721 University of Ljubljana, Aškerčeva Cesta 7, Ljubljana 1000, Slovenia; § Institute of Microbiology and Parasitology, Veterinary Faculty, 54767 University of Ljubljana, Gerbičeva 60, Ljubljana 1000, Slovenia; ∥ Department of Microbiology and Plant Biology, Institute of Molecular Genetics and Genetic Engineering, 229792 University of Belgrade, Vojvode Stepe 444a, Belgrade 11 042, Serbia

## Abstract

Novel bacterial topoisomerase inhibitors (NBTIs) target bacterial topoisomerases through binding modes distinct from fluoroquinolones but are often limited by hERG channel inhibition. Our previous findings indicated that the introduction of a non-aminopiperidine linker and a halogenated phenyl enzyme-binding moiety can partially reduce cardiotoxicity. In this paper, we describe whether optimizing the DNA-intercalating 1,5-naphthyridine moiety could further improve safety while maintaining antibacterial potency. Several optimized compounds showed strong enzyme inhibition and potent antibacterial activity, particularly against Gram-positive pathogens. Compound **13** displayed broad-spectrum activity, with MICs as low as 0.008 μg/mL against Gram-positive and 0.125 μg/mL against Gram-negative bacteria. Safety profiling revealed reduced cytotoxicity and hERG binding compared to earlier series. Compounds **6**, **20**, **24**, and **25** were nontoxic in zebrafish embryo assays. Compound **13** showed toxicity only at high doses and protected embryos in a lethal*Staphylococcus aureus* infection model, highlighting its potential as a preclinical lead.

## Graphical abstract

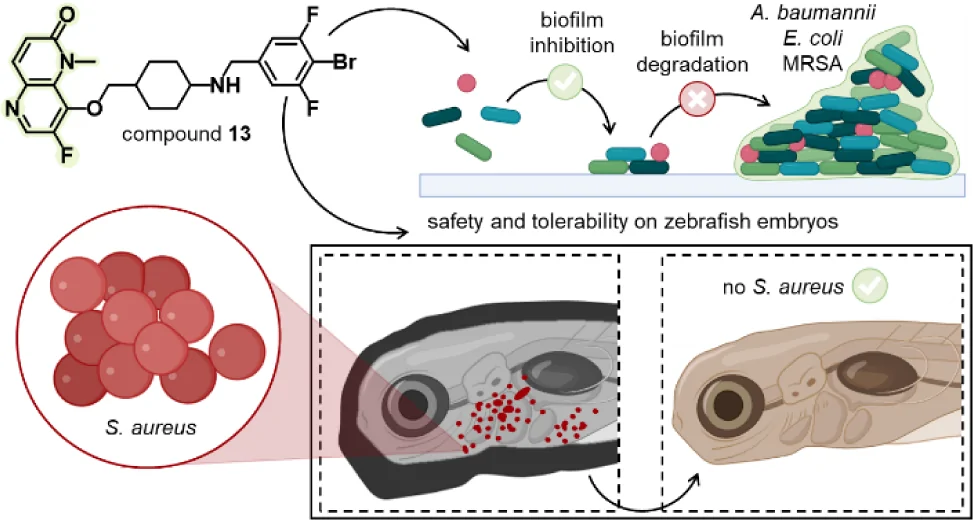

## 1 Introduction

Fluoroquinolones are among the most widely used antibiotics that target bacterial type II topoisomerases—DNA gyrase and topoisomerase IV (TopoIV)—but their extensive use has led to widespread bacterial resistance, significantly reducing their effectiveness and usage.^1–3^ In the last two decades, several alternatives to fluoroquinolones have been introduced.^4–7^ Among them, a novel class of antibacterial agents, known as novel bacterial topoisomerase inhibitors (NBTIs), has shown particular promise.^8,9^ Unlike traditional fluoroquinolones, NBTIs feature distinct chemical structures, alternative binding sites on bacterial type II topoisomerases, and employ unique mechanisms of inhibition, making them promising candidates against fluoroquinolone-resistant bacteria.^8,9^ NBTIs are composed of three distinct structural features which enables them to form a ternary complex with the enzyme and DNA.^10,11^ The DNA-intercalating left-hand-side (LHS) moiety intercalates between the central DNA base pairs, while the right-hand-side (RHS) moiety binds into a deep, hydrophobic, noncatalytic binding pocket formed at the interface of two catalytic GyrA/ParC subunits of the enzyme (Figure 1).^12–14^ The linker bridges the LHS and the RHS fragments, guaranteeing correct spatial orientation of the NBTIs and influencing their overall physicochemical properties (Figure 1).^10,11^ So far, the most advanced NBTI, gepotidacin (GSK2140944), successfully completed Phase III clinical trials for the treatment of urinary tract infections caused by*Escherichia coli*, and Phase III trials for the treatment of uncomplicated urogenital gonorrhea and was recently approved by the FDA for both indications.^6,15–17^ Besides gepotidacin, only two other NBTIs reached clinical trials: viquidacin (NXL-101), which was discontinued due to safety concerns, and BWC0977, which is currently in Phase I clinical trials.^5,18,19^ A major challenge in development of NBTIs is their tendency to inhibit the human ether-à-go-go related gene (hERG) potassium channels, since they often share many of the structural features of known hERG inhibitors.^20–24^ Inhibition of hERG channels in the heart may lead to QT interval prolongation that may cause life-threatening arrhythmias.^20,21^ Current strategies to mitigate NBTI-induced hERG inhibition include modifying their structural and physicochemical properties, by removing/replacing secondary/tertiary amines, reducing overall lipophilicity, and eliminating oxygen–hydrogen bond acceptors.^21,25,26^ And still, most NBTIs contain secondary or tertiary amines within their central linker moiety, due to their role in binding the bacterial topoisomerases.^27,28^ Structural studies of*Staphylococcus aureus* DNA gyrase-NBTI complexes show that the linker’s secondary amine is important for high-affinity binding and antibacterial potency as it forms a key salt bridge interaction with the Asp83 residue of GyrA.^26,29^ Experimental data suggest that the presence of the secondary amine is significant for potent broad-spectrum activity, although compounds lacking this structural feature still exhibit antibacterial activity.^28,30,31^ Tertiary amines are a common structural feature of a piperidine NBTI linker moiety that contribute to antibacterial potency and favorable physicochemical properties of the compounds. However, they also have the potential to significantly contribute to hERG inhibition due to structural matching with the hERG toxicophore.^21,26,32^

**Figure 1:**
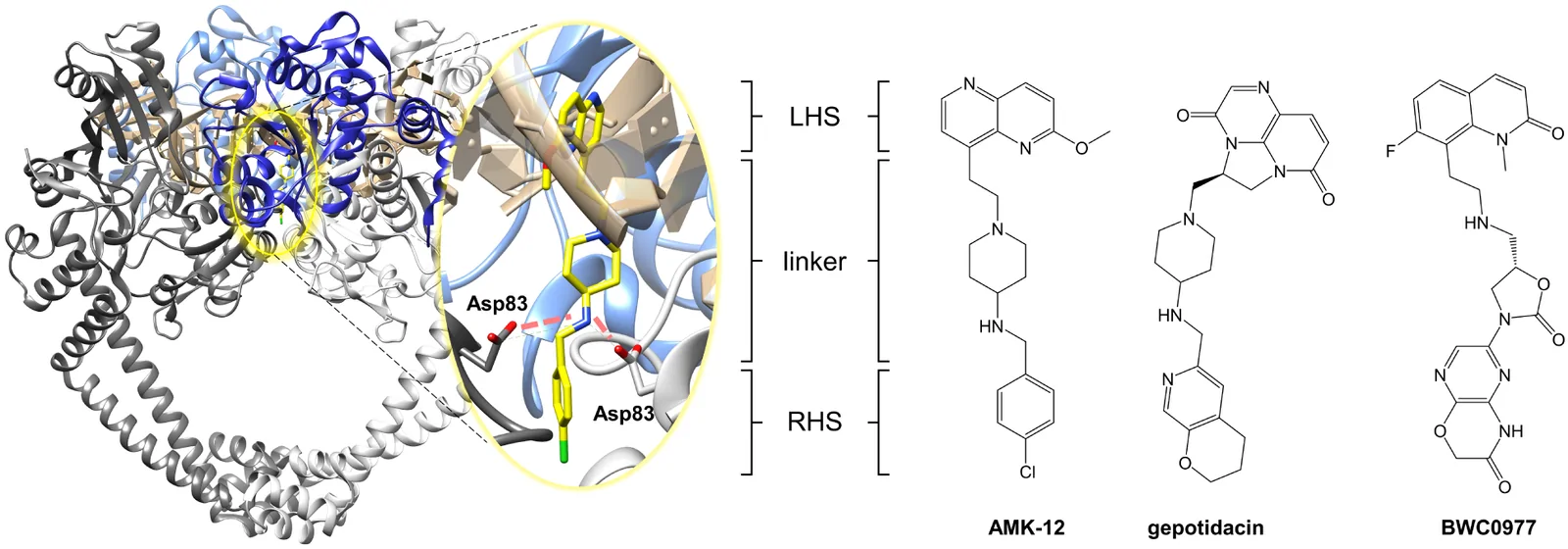
Our recently disclosed crystal structure of *S. aureus* DNA gyrase and DNA in complex with AMK-12 ligand (PDB ID: 6Z1A).^13^ Two GyrA subunits are depicted in black and white, the two GyrB subunit are depicted in light and dark blue. The cocrystallized ligand is presented in yellow, with red dashed lines showing its interaction with the Asp83 residue. The key structural features of NBTIs are shown along with structural representations of the AMK-12, gepotidacin, and BWC0977.

Recognizing the dual role of the linker in both target engagement and hERG binding, we previously investigated alternative linker designs.^25,26^ It has been previously shown that the replacement of the secondary amine in the NBTI linker with an amide results in reduced hERG inhibition while maintaining potent antibacterial activity against Gram-positive bacteria.^26,31,33^ However in our previous study this replacement lead to a complete loss of antibacterial activity in Gram-negative species.^26^ Excluding the tertiary amine by substituting the piperidine with a cyclohexane or tetrahydropyran linker as well as reducing the overall ligand’s lipophilicity by introducing a polar group (e.g., −OH) resulted in NBTIs with reduced hERG inhibition.^25^ Implementing an oxymethylene cyclohexyl linker instead of the classic aminopiperidine linker reduced hERG inhibition 10-fold while retaining potent antibacterial activity. While some improvement in lowering hERG inhibition was achieved by replacing the linker alone, we did not reach the desired hERG threshold inhibitory value of IC_50_ > 30 μM.^25^ Therefore, we have opted to investigate whether optimizing the DNA-intercalating moiety can contribute to further hERG reduction while retaining good antibacterial activity, particularly against Gram-negative bacteria. With the LHS optimization we wanted to modulate lipophilicity, aromatic surface area, and substituent distribution, all of which may contribute to hERG binding, while maintaining the previously optimized linker-RHS framework.

## 2 Results and Discussion

### 2.1 NBTI Structural Optimization Strategy

In our previous studies, we have reported that the halogen atom at the *para*-position of the phenyl RHS moiety strongly contributes to the interaction of the NBTI with GyrA.^34^ This was supported by our recently reported crystal structure of *S. aureus* DNA gyrase in complex with AMK-12 ligand (PDB ID: 6Z1A), in which a strong symmetrical bifurcated halogen-bond was formed between the chlorine substituent of the NBTI and both backbone carbonyl oxygens of GyrA Ala68 residues in*S. aureus* DNA gyrase.^13^ We have also shown that the halogen bond strength can be enhanced by introducing electron-withdrawing groups at the *meta*-positions of the *p*-halogenated phenyl RHSs (i.e., *ortho*-relative to the halogen substituent), which further improved the NBTI’s enzyme inhibitory and antibacterial activity.^35^ In a 2021 SAR investigation the compound containing 4-bromo-3,5-difluorobenzyl RHS exhibited the most potent antibacterial activities among the series.^35^ However, a key drawback of a highly lipophilic RHS is its tendency to elevate hERG inhibitory activity. We addressed this issue by substituting the piperidine linker bearing an unwanted tertiary amine with more polar alternatives.^25^ Compounds with the oxymethylene tetrahydropyranyl exhibited lower hERG binding, but were also less potent.^25^ Compounds containing the 1-hydroxyethylene cyclohexyl linker might have been the best of both worlds, moderately cardiotoxic while displaying excellent biological activity, yet their synthetic pathway was by far the most unfavorable, with very low yields and more extreme synthetic conditions.^25^ These structural alterations led to excellent enzyme inhibition and antibacterial activity, as well as diminished hERG inhibition that still needed optimization to reach desired safety for *in vivo* studies. To further reduce the hERG inhibitory activity, we have designed a new, focused library of NBTIs, focusing on altering the DNA-intercalating left-hand-side (LHS) moiety (Figure 2). In this study we preserved the oxymethylene cyclohexyl linker and 4-bromo-3,5-difluorobenzyl RHS, as both showed optimal properties and synthetic feasibility. The novel LHS fragments were chosen either by employing scaffold hopping or by literature review.^36,37^ The performance of our new series of compounds was compared to representative compounds from our previous studies, compounds **1**-**4** (Table 1).

**Figure 2:**
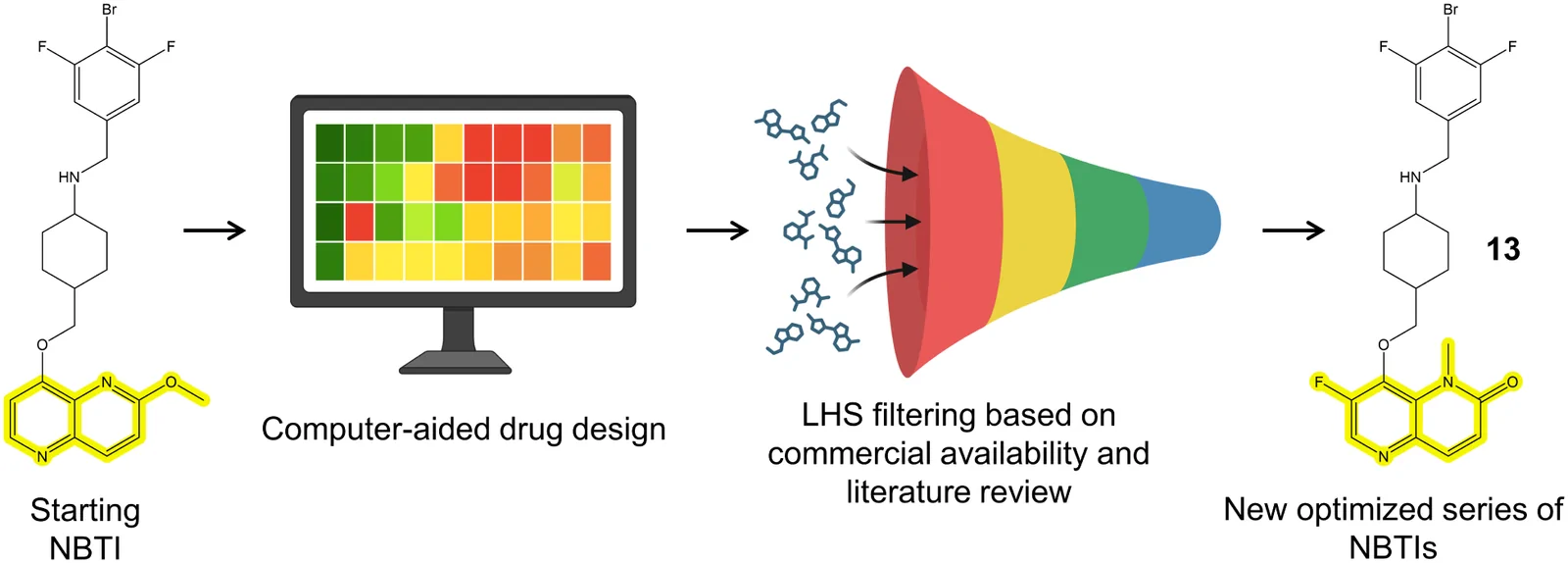
LHS diversification. More than 20 different NBTIs with diverse LHS moieties were synthesized. Compound **13** performed best in terms of antibacterial activity and showed a slight reduction in hERG inhibition.

**Table 1:**
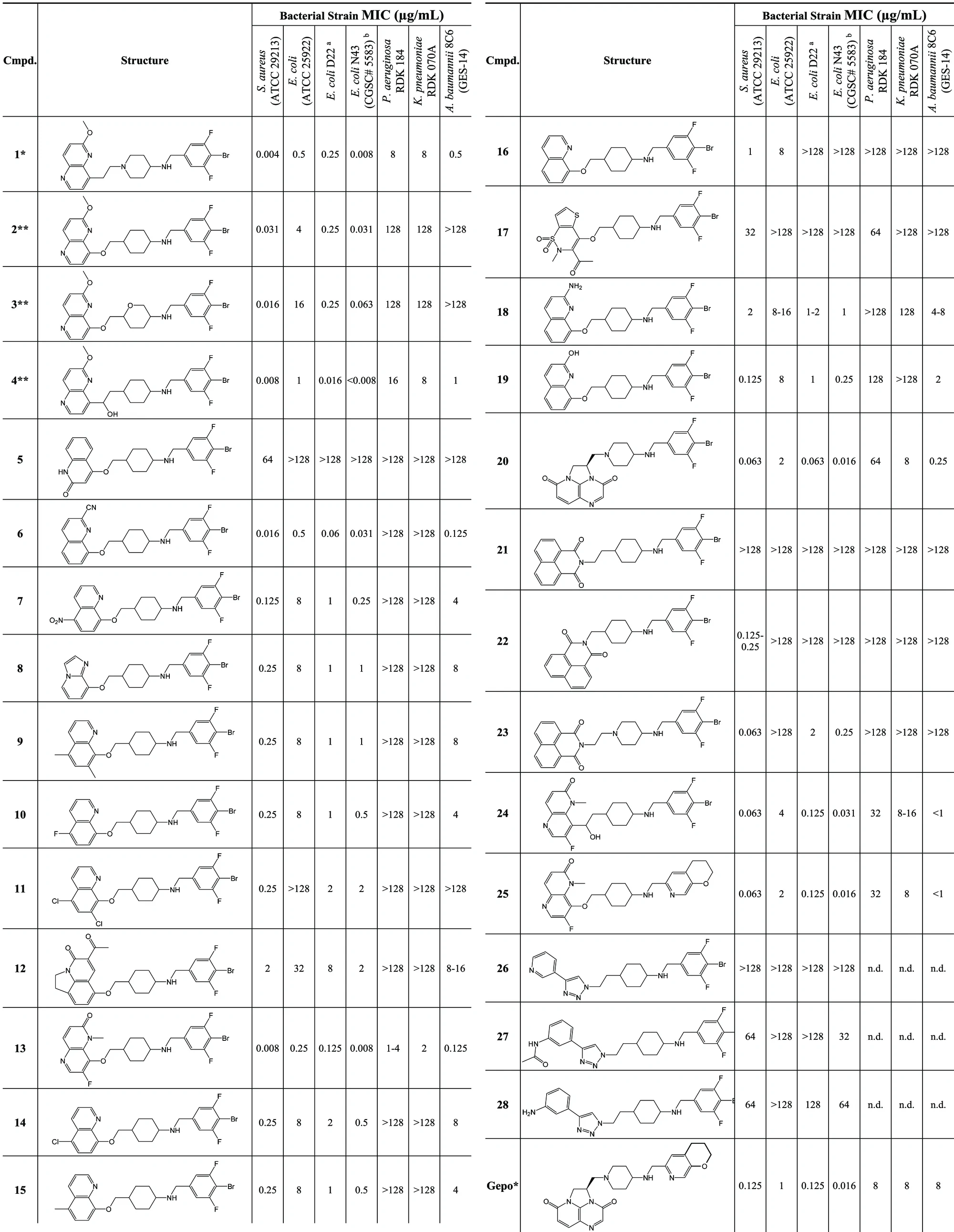
Antibacterial Activity of the Optimized Series of NBTI against a Panel of Gram-Positive and Gram-Negative Bacteria^c^

a *E. coli* strain with mutation in the lpxC gene that increases membrane permeability.

b *E. coli* strain lacking the AcrA component of the AcrAB-TolC multidrug efflux pump.

c The MIC readouts may vary by one doubling dilution step. n.d.: not determined; *ref 35; **ref 25.

### 2.2 Scaffold Hopping and Molecular Docking Calculations

The initial step in designing new NBTI analogs was LHS-based scaffold hopping through commercially available chemical libraries using SwissSimilarity web tool.^38^ New potential LHS fragments were chosen based on similarity to known LHS fragments and filtered based on the presence of either a hydroxyl or bromine group that would enable coupling with the linker. A virtual combinatorial library was constructed by using an *in-house*-devised BIOVIA, Dassault Systèmes, Pipeline Pilot 9.2.0 protocol, where the potential LHS fragments were linked the oxymethylene cyclohexyl linker and the 4-bromo-3,5-difluorobenzyl RHS.^25^ Prior to the synthesis, all combinatorially assembled NBTI analogs were subjected to a series of flexible molecular docking calculations to predict their binding conformations. The library was docked into DNA gyrase in complex with DNA from both *S. aureus* (PDB ID: 6Z1A) and *E. coli* (PDB ID: 6RKS).^13,14^ The docked poses were visually inspected and several new LHS fragments were selected, based on the docking scores and the economic feasibility of the synthesis.

### 2.3 Chemistry

The synthesis of most new NBTIs generally consisted of three main reaction steps: Mitsunobu reaction, Boc deprotection, and reductive amination. While most LHS fragments were commercially available, some had to be synthesized. The structures of all intermediates (i–xlii) are available in Supporting Information Table S1. Stereochemistry is not defined, unless specifically stated. Most new LHS scaffolds had hydroxyl group as an attachment functionality, and were easily linked to the oxymethylene cyclohexyl linker using Mitsunobu reaction (Scheme 1). The preparation of compounds **8–17** started with reductive amination of 4-bromo-3,5-difluorobenzaldehyde and (4-aminocyclohexyl)­methanol, which produced the RHS-linker intermediate **i** (Scheme 1A). The secondary amine of **i** was then protected using Boc_2_O, producing **ii**, and coupled with various LHS alcohols using Mitsunobu reaction to produce intermediates **iii-xii** (Table S1). In the final step, the Boc-protecting group of these intermediates was removed under acidic conditions, yielding products **8–17** (Scheme 1A). Compounds **5, 18,** and **19** were prepared by transformation of hydroxy group of the linker into an alkyl sulfonate, followed by a nucleophilic substitution with the LHS alcohols, which yielded the LHS-linker adducts (compound **xiv-xvi**) (Scheme 1B, Table S1). Boc-deprotection followed, producing intermediates **xx-xxii** (Table S1), and finally, reductive amination was performed, giving the final compounds **5**, **18** and **19**. Compounds **6**, **7,** and **25** were prepared by first joining the LHS alcohols and the *tert-*butyl (4-(hydroxymethyl)­cyclohexyl)­carbamate using Mitsunobu reaction (**xvii-xix**), then deprotecting the Boc group on the linker under acidic conditions (**xxiii-xxv**) and combining the LHS-linker with the RHS using reductive amination (Scheme 1B, Table S1). A gepotidacin “hybrid” molecule was prepared by combining our designed LHS-linker moiety with the RHS from gepotidacin, therefore 3,4-dihydro-2*H*-pyrano­[2,3-*c*]­pyridine-6-carbaldehyde was used to produce **25**.

**Scheme 1:**
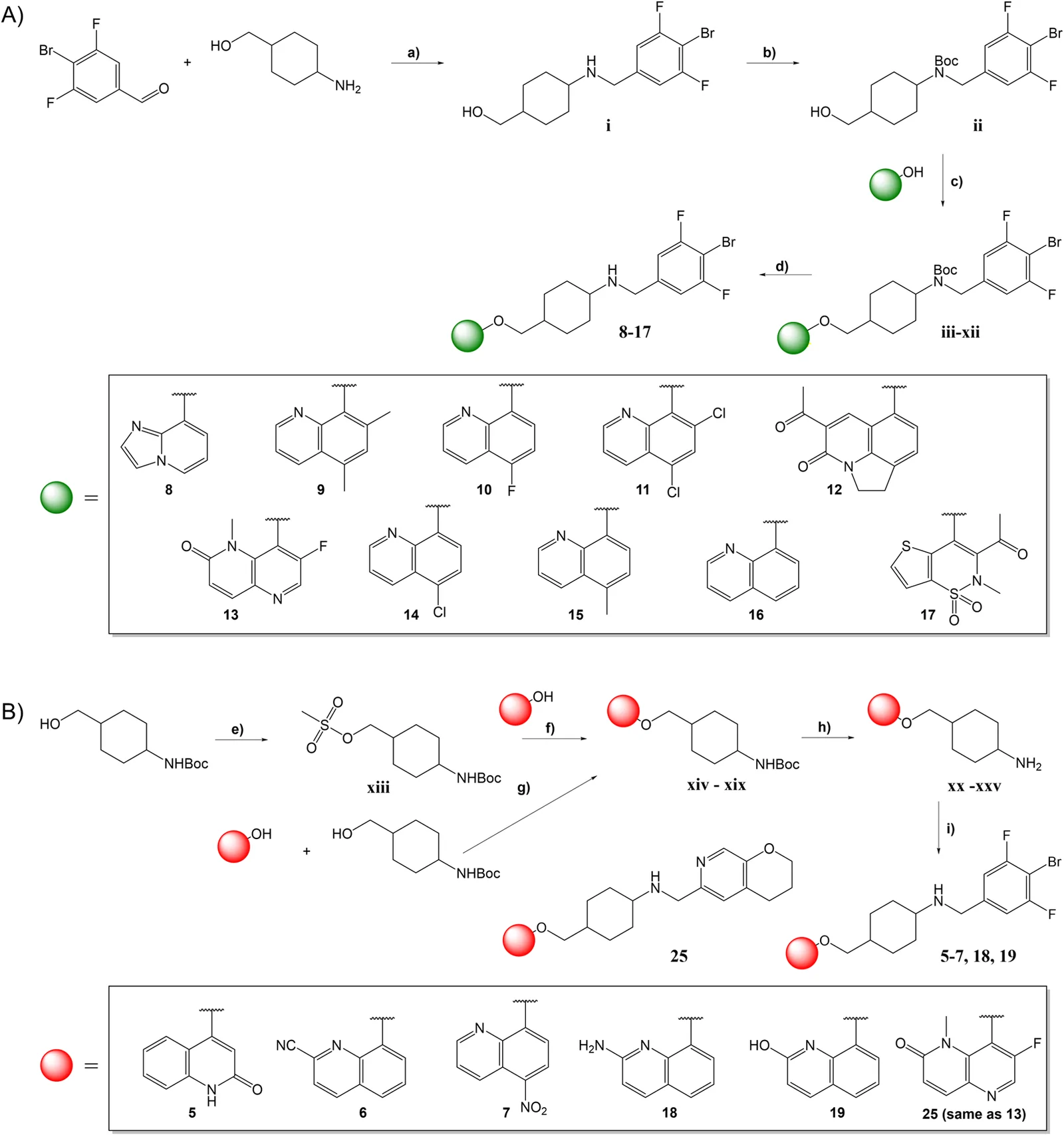
Synthetic Pathways for the Synthesis of New NBTIs; A) Synthesis of Compound **8**-**17**; The Various LHS Moieties Are Shown as a Green Circle; B) Synthesis of Compounds **5**-**7**, **18**, **19**, and **25**; The Various LHS Moieties Are Shown as a Red Circle^a^

a Reagents and conditions: a) NaBH_3_CN/NaBH­(OAc)_3_, CH_3_COOH, MeOH, 50 °C; b) Boc_2_O, TEA, DCM, rt; c) Appropriate alcohol (see Materials and Methods), Ph_3_P, DIAD, THF, 60 °C; d) TFA, DCM, rt; e) MsCl, TEA, DCM, 0 °C; f) 1. Appropriate alcohol (see Materials and Methods), NaH, DMF, 50 °C, 2. DMF, 110 °C; g) Ph_3_P, DIAD, THF, 60 °C; h) TFA, DCM, rt; i) NaBH_3_CN/NaBH­(OAc)_3_, CH_3_COOH, MeOH, 50 °C.

Compound **20** is another gepotidacin “hybrid”, with the same LHS-linker moiety as gepotidacin and a 4-bromo-3,5-difluorobenzyl RHS. The LHS-linker moiety was synthesized as described in WO 2009/141398, giving **xxxvi**.^36^ The Boc protecting group of **xxxvi** was removed under acidic conditions, yielding **xxxvii**, which was transformed into the final compound, **20**, using reductive amination (Scheme 2).

**Scheme 2:**
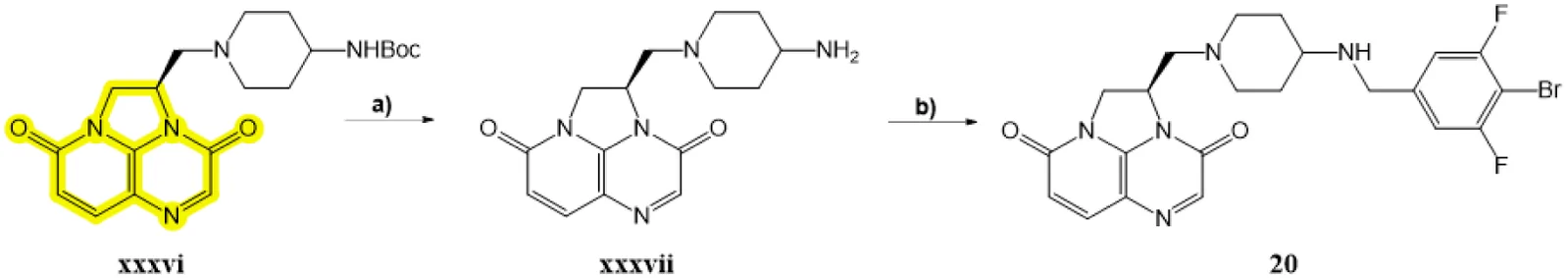
Synthesis of Compound **20** [Fn sch2-fn2]

a Reagents and conditions: a) TFA, DCM, rt; b) NaBH_3_CN/NaBH­(OAc)_3_, CH_3_COOH, MeOH, 50 °C.

Compounds **21**-**23** were synthesized using 1H-benzo­[*de*]­isoquinoline-1,3­(2*H*)-dione as the LHS and coupled with different linkers under Mitsunobu conditions in order to obtain intermediates **xxvi**-**xxviii** (Scheme 3). The Boc-protective group on these intermediates was then removed under acidic conditions, yielding intermediates **xxix**-**xxxi**. Finally, the LHS-linker was coupled with the 4-bromo-3,5-difluorobenzaldehyde under reductive amination conditions to produce the final compounds **21**-**23**.

**Scheme 3:**
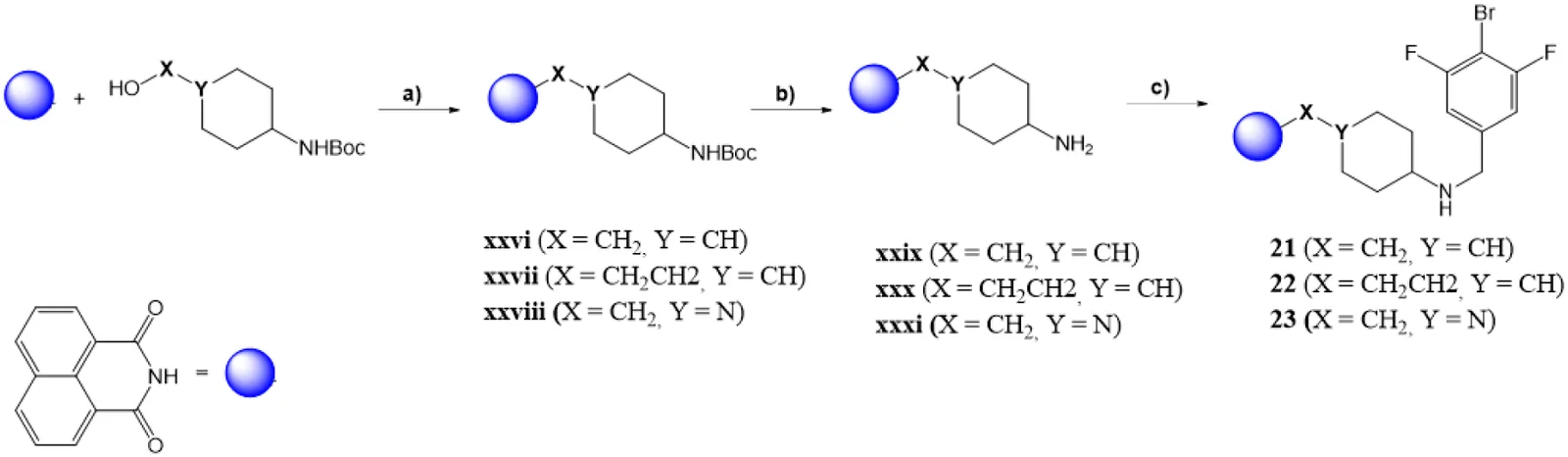
Synthesis of Compounds **21**-**23** [Fn sch3-fn3]

a Reagents and conditions: a) Ph_3_P, DIAD, THF, 60 °C; b) TFA, DCM, rt; c) NaBH_3_CN/NaBH­(OAc)_3_, CH_3_COOH, MeOH, 50 °C.

In our previous publications, we have established an NBTI compound containing a 1-hydroxyethylene cyclohexyl linker and a 4-bromo-3,5-difluorobenzyl RHS (compound **4**, Table 1), as the most active compound of the series, with the most balanced antibacterial activity to toxicity ratio.^25,39^ As described in the design section, the synthesis of this compound was rather low-yielding and challenging, this is why we prepared a single analogue, **24**, using the most promising new LHS-scaffold, 7-fluoro-1-methyl-1,5-naphthyridin-2­(1*H*)-one (Scheme 4). This new scaffold has been previously described in patent literature and is structurally related to the LHS used in BWC0977 by BugWorks.^5,37^ The synthesis of **24** began by synthetizing the LHS alcohol, as described in WO 2010/045987,^37^ giving **xxxii**. The alcohol was then transformed into a bromide by nucleophilic substitution with PBr_3_ (**xxxiii**), the treatment of **xxxiii** with *n*-butyl lithium at −78 °C produced the lithioanion, which reacted with the *tert-*butyl (4-(2-oxoethyl)­cyclohexyl)­carbamate to give benzylic alcohol (**xxxiv**).^25,40^ The Boc protecting group on **xxxiv** was removed under acidic conditions, producing **xxxv**, and reductive amination was used to give **24**.

**Scheme 4:**
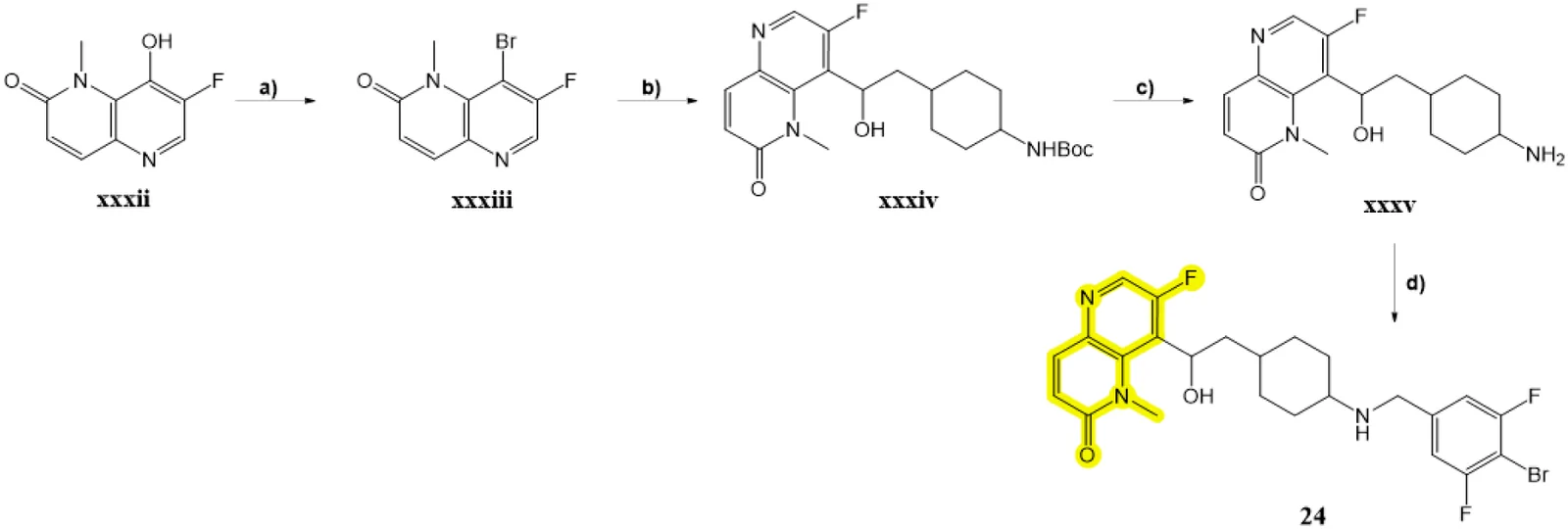
Synthesis of Compound **24** [Fn sch4-fn4]

a Reagents and conditions: a) PBr_3_, DMF, 0 °C; b) *tert*-butyl (4-(2-oxoethyl)­cyclohexyl)­carbamate, *n*BuLi, THF, −78 °C; c) TFA, DCM, rt; d) NaBH_3_CN/NaBH­(OAc)_3_, CH_3_COOH, MeOH, 50 °C.

Compounds **26**-**28** were prepared first by transforming the alcohol into a mesylate and then adding NaN_3_, to obtain the azide **xlix** (Scheme 5). The azide was reacted with an appropriate (hetero)­aromatic alkyne to give **l** and **li**. The Boc protecting group was then removed giving **lii** and **liii**, which were transformed into **26** and **27** via reductive amination. **28** was prepared from **27** by amide hydrolysis.

**Scheme 5:**
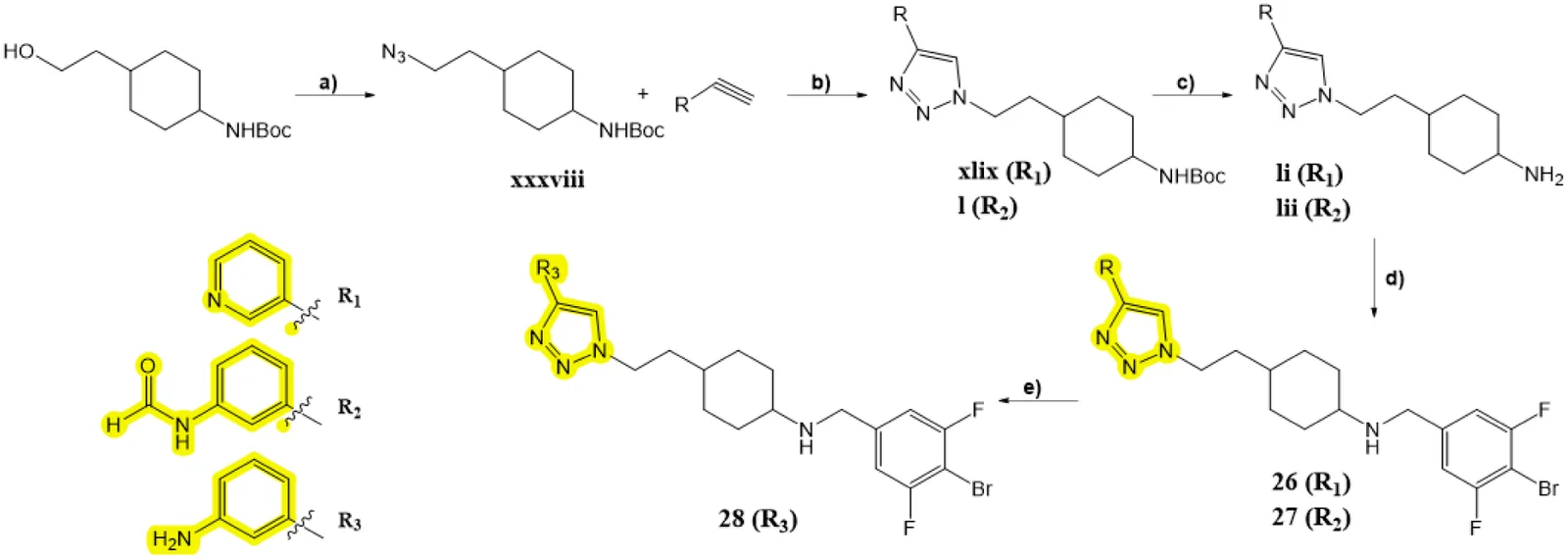
Synthesis of Compounds **26-28** [Fn sch5-fn5]

a Reagents and conditions: a) 1. MsCl, TEA, 0 °C, 2. NaN_3_, DMF, rt; b) CuSO_4_, ascorbic acid, MeOH, rt; c) TFA, DCM, rt; d) NaBH_3_CN/NaBH­(OAc)_3_, CH_3_COOH, MeOH, 50 °C; e) 1. conc. HCl, 1,4-dioxane, 80 °C, 2. 50% NaOH

### 2.4 MIC-Driven Structure–Activity Relationship Exploration

*In vitro* antibacterial activity, expressed as minimum inhibitory concentration (MIC), was determined against a panel of Gram-positive (*S. aureus*, methicillin-resistant *S. aureus* (MRSA), *Enterococcus faecalis*, and *Enterococcus faecium*) and Gram-negative bacterial pathogens (*E. coli*, *Klebsiella pneumoniae*, *Pseudomonas aeruginosa*, *Acinetobacter baumannii*) enriched by various MRSA and *E. coli* strains resistant to multiple antibiotics as well as *E. coli* strains with increased membrane permeability and lack of efflux pump (Table 1). The MIC values for MRSA strains, *Enterococcus faecalis*, *Enterococcus faecium*, and *A. baumannii* 2012–70–100–69 are presented separately in Supporting Information Table S2. As demonstrated, the majority of NBTIs exhibit stronger antibacterial activity against Gram-positive bacteria (such as *S. aureus*, MRSA, *E. faecalis*, and *E. faecium*) compared to Gram-negative bacteria. This result was not unexpected, as we have had similar results with our previous series of compounds, most likely explained by the difference in bacterial cell wall structure and efflux pump mechanisms. MRSA strains were similarly susceptible as *S. aureus* to all of the assayed NBTIs. The comparison of MIC values against *S. aureus* and MIC values against MRSA QA-11.7 and MRSA QA-12.1, which are both resistant to ciprofloxacin, also substantiate the claim that this new series of NBTIs shows no cross-resistance with fluoroquinolones (Tables 1 and S2). We have also tested our compounds against three ciprofloxacin-resistant Gram-negative strains (Table S2). A 2- to 4-fold increase in MIC was noticed against the ciprofloxacin-resistant *A. baumannii* 2012–70–100–69 compared to the reference *A. baumannii* strain (Table S2). An even higher increase in MIC was noticed against the two ciprofloxacin-resistant tested *E. coli* clinical isolates (MIC­(ciprofloxacin) > 8 μg/mL in both *E. coli* 11477 and *E. coli* 12344) (Table S2). The underlying cause of fluoroquinolone resistance in the two clinical isolates tested remains to be determined. Although fluoroquinolone-NBTI cross-resistance is improbable, and the resistance of the two stains to ciprofloxacin may be due to other bacterial adaptations, we are unable to definitively exclude this possibility. Furthermore, the comparison of antibacterial activities for the wild-type *E. coli*, *E. coli* *N43* strain (that lacks the AcrA component of the AcrAB-TolC multidrug efflux pump), and *E. coli* *D22* strain (with increased membrane permeability) clearly indicates suboptimal membrane permeation and increased efflux in Gram-negative bacteria. This in turn explains the weaker activity of these NBTIs in *E. coli*, and may explain weaker activity against other tested Gram-negative bacteria (e.g., *K. pneumoniae*, *A. baumannii*, *P. aeruginosa*).

Based solely on the antibacterial activity, compounds **6** and **13** show most promise as effective broad-spectrum antibacterials (Table 1). In fact, **13** is even more potent than any of our previously disclosed NBTIs, displaying MIC values as low as 0.008 μg/mL in Gram-positive bacteria and 0.125 μg/mL in Gram-negative bacteria, respectively. It also exhibits greater antibacterial activity and a broader spectrum than the clinically relevant NBTI gepotidacin, with overall lower MIC values across the panel. The improvement of antibacterial activity compared to gepotidacin is especially pronounced in *A. baumannii*, where compounds **13** displays a 64-fold lower MIC.

Additionally, compounds **20**, **24** and **25** all display favorable antibacterial activity across the bacterial spectrum, similar to compounds from our previous series. All three compounds display an MIC of 0.063 μg/mL MICs in *S. aureus* and an MIC of 2–4 μg/mL in *E. coli*, as well as improved activity against *A. baumannii*, with sub-1 μg/mL MICs.

### 2.5 Antibiofilm Assays

The antibiofilm activity of the five compounds that displayed the most favorable or improved antibacterial activity, **6**, **13**, **20**, **24**, and **25**, was explored and compared to gepotidacin. All tested compounds showed a dose-dependent inhibition of biofilm formation in *A. baumannii* and *E. coli* (Figure 3A,B). Interestingly, they displayed a dose-dependent stimulation of biofilm formation in MRSA (Figure 3C). In *A. baumannii*, **13** and **20** displayed 80% and 85% inhibition of biofilm formation at the concentration equal to 1/2 MIC, respectively, whereas gepotidacin only achieved 45% inhibition. In *E. coli*, at the concentration equal to 1/2 MIC, all tested compound displayed inhibition above 92%, while gepotidacin showed slightly lower inhibition, around 75%. In MRSA, a slight inhibition was observed at the concentrations equal to 1/4 MIC and 1/2 MIC, while at higher concentrations, all compounds stimulated biofilm growth. The stimulation of biofilm formation at sub-MIC concentrations is a phenomenon that has been broadly described in the literature and may vary significantly depending on the bacterial strain and antibiotic.^41–44^ The same reasoning can be applied to try to explain why biofilm formation in MRSA is promoted at MIC concentration or higher, however this phenomenon has not been extensively studied in the literature. If we compare the MIC and minimal bactericidal concentration (MBC) values of the of **13** and gepotidacin in MRSA (Table S3), we can observe a several-fold difference. The MBC/MIC ratio of **13** is 8, and 4 for gepotidacin, indicating that although the bacteria are inhibited at the MIC concentration, they are not yet killed, which might initiate a bacterial stress response, promote biofilm formation, and antibacterial tolerance.^45^ ^,^ The majority of compounds exhibited minimal to no ability to eradicate mature biofilms (Figure 3C–F).

**Figure 3:**
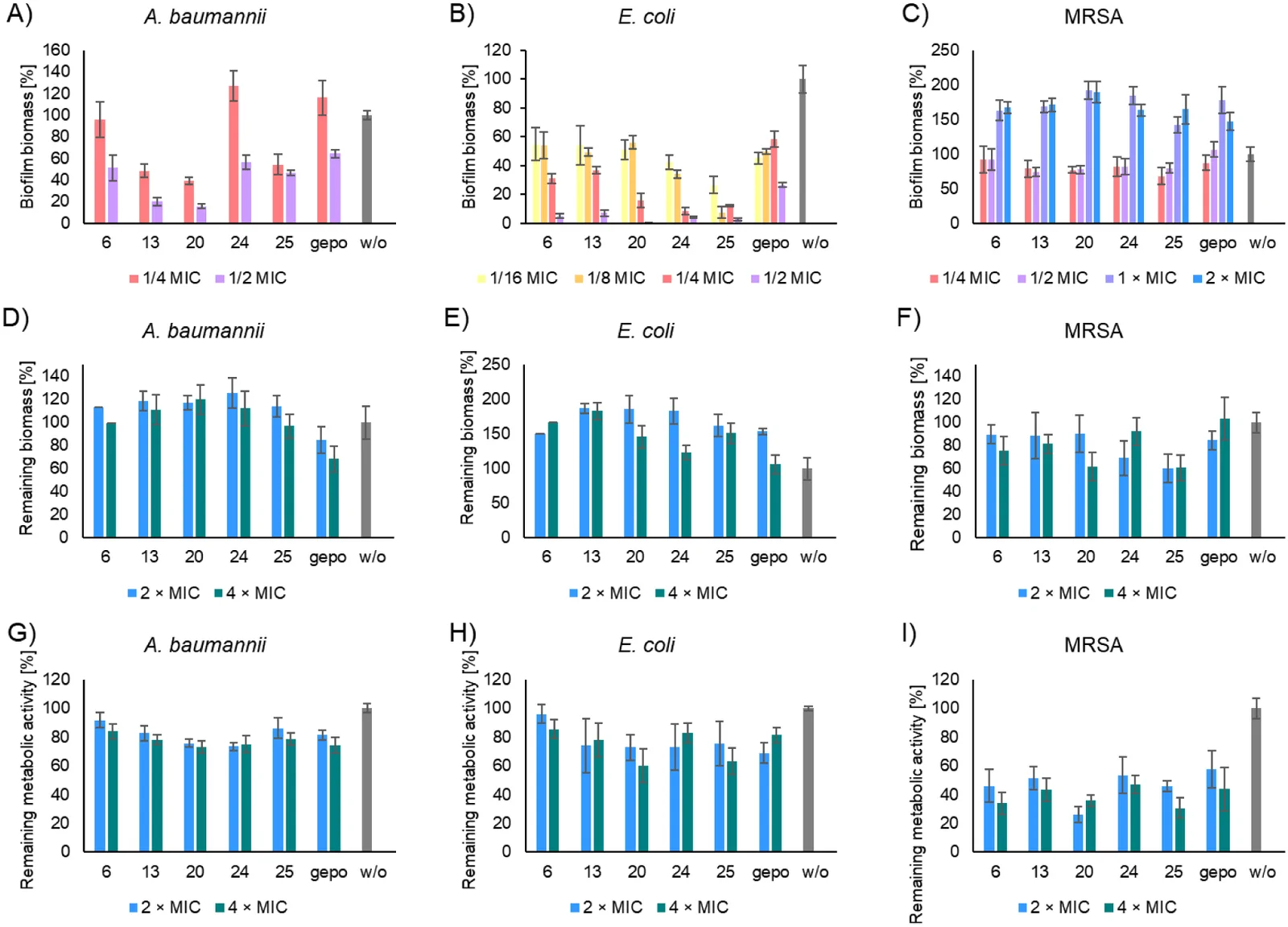
Antibiofilm assays. A–C) Inhibition of biofilm formation in *A. baumannii*, *E. coli*, and MRSA quantified using CV staining. D–F) Mature biofilm eradication in *A. baumannii*, *E. coli*, and MRSA quantified with CV. G–I) Metabolic activity of *A. baumannii*, *E. coli*, and MRSA mature biofilms determined by resazurin staining. In all figures, gepo denotes the antibacterial gepotidacin and w/o denotes conditions where no compound was used (negative control). Values are presented as mean ± SD.

In *A. baumannii*, all tested compounds slightly stimulated mature biofilm, however, gepotidacin displayed a 30% reduction in mature biofilm biomass at the concentration equal to 4 × MIC (Figure 3D). In *E. coli*, all compounds, including gepotidacin, stimulated biofilm growth compared to untreated mature biofilm (Figure 3E). In MRSA, compounds **20** and **25** showed 40% reduction in biofilm biomass at the concentration equal to 4 × MIC (Figure 3F), while gepotidacin did not have any significant effect at this concentration.

Resazurin staining of treated mature biofilms revealed a modest reduction in bacterial viability across all tested bacterial strains (Figure 3G–I). The most pronounced reduction of viability was observed in MRSA, where the proportion of viable cells was around 30–50%. In contrast, viability remained higher in *E. coli* and *A. baumannii*, at roughly 65–95%.

### 2.6 Topoisomerase Selectivity

The on-target potency of the optimized NBTI compounds was determined by *in vitro* enzyme inhibition assays on isolated DNA gyrase and TopoIV enzymes from both *S. aureus* and *E. coli*. Similar to our previous series, these compounds exhibited low nanomolar IC_50_ values on *S. aureus* DNA gyrase and *E. coli* TopoIV, which are the primary targets in Gram-positive and Gram-negative bacteria, respectively (Table 2). The IC_50_ values for *S. aureus* TopoIV and *E. coli* DNA gyrase were several-fold higher. The inhibition of the enzymes was determined for all compounds in a DNA gyrase supercoiling microtiter high-throughput plate-based assay from Inspiralis Ltd. and expressed as residual activities (RA) at 1 μM. The IC_50_ determinations were conducted by using DNA gyrase/TopoIV gel-based assay from Inspiralis Ltd. only for three compounds, **6**, **13**, and **19**, that exhibited a combination of low RA and good MIC values. Additionally, the selectivity of the compounds was evaluated using the human orthologue enzyme, human topoisomerase IIα. All compounds displayed favorable selectivity (Table 2).

**Table 2:** Potencies of New NBTI Compounds against Target Enzymes and Human TopoIIα, Their hERG Inhibitory Activity, and Cytotoxicity Data on HepG2 and HUVEC Cell Lines^a^

	RA @ 1 μM [%] or IC_50_ ^b^ [μM]							
Compound	*S. aureus* DNA Gyrase	*S. aureus* TopoIV	*E. coli* DNA Gyrase	*E. coli* TopoIV	Human TopoIIα (RA @ 10 μM)^e^	HepG2 RA @ 50 μM [%] or IC_50_[μM]	HUVEC RA @ 50 μM [%] or IC_50_ [μM]	hERG IC_50_ [μM]
1*	0.004 ± 0.000^c^ μM or 0.058 ± 0.002^f^ μM	0.050 ± 0.007^d^ μM or 0.421 ± 0.101^g^ μM	0.087 ± 0.007^c^ μM or 0.268 ± 0.109^f^ μM	0.033 ± 0.001^d^ μM or 0.002 ± 0.001^g^ μM	99.5 ± 0.02%	12.4 ± 1.84 μM	n.d.	0.296 μM
2**	0.113 ± 0.003^c^ μM	3.12 ± 0.275^d^ μM	0.234 ± 0.048^c^ μM	0.018 ± 0.002^d^ μM	109 ± 1.70%	22.6 ±0.57 μM	n.d.	4.63 μM
3**	0.056 ± 0.001^c^ μM	>100^d^ μM	0.334 ± 0.114^c^ μM	0.016 ± 0.005^d^ μM	104 ± 2.62%	68 ± 8%	n.d.	16.1 μM
4**	0.021 ± 0.001^c^ μM	1.56 ± 0.159^d^ μM	0.054 ± 0.002^c^ μM	0.009 ± 0.001^d^ μM	96.8 ± 19.57%	9.83 ± 0.030 μM	n.d.	2.02 μM
**5**	48.1 ± 4.77%	98.1 ± 3.63%	49.3 ± 2.75%	103 ± 1.77%	91.9 ± 8.13%	81.2 ± 2.25%	96.2 ± 9.21%	n.d.
**6**	12.1 ± 1.03%	0 ± 21.87%	0 ± 2.84%	0 ± 10.9%	95.6 ± 6.12%	109 ± 9.97%	99.4 ± 5.19%	^a^
	0.197 ± 0.104^f^ μM	(>1)^g^ μM	(>1)^f^ μM	0.005 ± 0.001^g^ μM				
**7**	17.9 ± 1.42%	0 ± 17.99%	3.55 ± 7.32%	9.22 ± 5.70%	100 ± 4.38%	33.5 ± 4.49 μM	26.5 ± 3.20 μM	0.79 μM
**8**	102 ± 11.4%	98.3 ± 0.92%	69.6 ± 7.45%	103 ± 2.46%	97.0 ± 6.38%	43.9 ± 0.89 μM	21.2 ± 3.03 μM	3.31 μM
**9**	16.1 ± 1.24%	24.3 ± 7.66%	8.80 ± 6.05%	20.0 ± 9.51%	96.5 ± 8.77%	24.0 ± 0.45 μM	19.8 ± 0.75 μM	9.38 μM
**10**	17.3 ± 1.83%	0 ± 34.11%	16.7 ± 7.76%	34.6 ± 1.12%	88.9 ± 8.02%	28.1 ± 3.67 μM	20.9 ± 3.09 μM	0.87 μM
**11**	19.4 ± 2.06%	51.8 ± 0.63%	28.9 ± 12.8%	46.3 ± 7.89%	94.0 ± 2.09%	44.3 ± 3.18 μM	28.0 ± 8.21 μM	12.22 μM
**12**	33.5 ± 3.17%	93.2 ± 1.82%	35.1 ± 0.26%	64.3 ± 8.36%	93.8 ± 6.67%	36.3 ± 3.30 μM	29.0 ± 0.31 μM	5.65 μM
**13**	10.31 ± 1.87%	0 ± 7.88%	0 ± 1.38%	0 ± 11.15%	90.6 ± 5.55%	35.9 ± 0.71 μM	46.5 ± 0.27 μM	5.36 μM
	0.047 ± 0.0003^f^ μM	(>1)^g^ μM	(0.085 ± 0.003)^f^ μM	0.001 ± 0.0003^g^ μM				
**14**	24.7± 3.02%	51.9 ± 5.63%	17.5 ± 3.15%	25.4 ± 13.35%	104 ± 1.81%	23.8 ± 0.35 μM	15.8 ± 0.35 μM	2.41 μM
**15**	26.9 ± 2.57%	25.9 ± 1.10%	13.8 ± 5.06%	38.8 ± 5.07%	99.7 ± 1.00%	44.8 ± 4.99 μM	37.7 ± 0.030 μM	0.75 μM
**16**	n.d.	n.d.	n.d.	n.d.	n.d.	n.d.	n.d.	n.d.
**17**	108 ± 8.73%	101 ± 0.75%	63.9 ± 15.44%	95.2 ± 12.6%	106 ± 1.88%	95.8 ± 24.4%	71.9 ± 15.9%	n.d.
**18**	33.4 ± 3.39%	16.3 ± 15.1%	15.8 ± 7.39%	34.75 ± 14.53%	97.0 ± 1.90%	11.9 ± 0.260 μM	11.9 ± 0.09 μM	0.470 μM
**19**	7.85 ± 6.36%	5.81 ± 3.21%	1.21 ± 1.93%	0 ± 13.4%	106 ± 2.20%	23.5 ± 0.06 μM	19.2 ± 0.300 μM	4.48 μM
	0.592 ± 0.300^f^ μM	(>1)^g^ μM	(>1)^f^ μM	0.014 ± 0.003^g^ μM				
**20**	10.6 ± 2.65%	5.55 ± 18.55%	5.60 ± 3.86%	38.1 ± 1.59%	106 ± 2.05%	87.6 ± 10.5%	73.0 ± 15.6%	13.7 μM
**21**	66.4 ± 12.4%	99.3 ± 0.22%	54.3 ± 21.3%	87.4 ± 12.7%	109 ± 0.33%	86.4 ± 13.1%	74.7 ± 7.72%	1.55 μM
**22**	42.4 ± 2.73%	47.4 ± 3.68%	34.0 ± 1.93%	18.7 ± 8.14%	95.8 ± 12.39%	84.4 ± 3.57%	95.6 ± 14.71%	0.153 μM
**23**	22.01 ± 2.09%	18.80 ± 18.58%	0 ± 1.83%	2.55 ± 15.24%	104 ± 0.45%	115 ± 6.14%	68.4 ± 10.73%	0.0121 μM
**24**	44.2 ± 3.79%	2.53 ± 0.92%	18.6 ± 5.31%	23.4 ± 8.75%	102 ± 2.20%	68.6 ± 18.0%	78.0 ± 12.9%	0.377 μM
**25**	26.5 ± 7.87%	0 ± 14.60%	1.78 ± 3.90%	18.0 ± 6.71%	92.2 ± 7.81%	105 ± 11.9%	75.2 ± 0.62%	13.7 μM
**26**	n.d.	n.d.	n.d.	n.d.	n.d.	n.d.	n.d.	n.d.
**27**	n.d.	n.d.	n.d.	n.d.	n.d.	n.d.	n.d.	n.d.
**28**	n.d.	n.d.	n.d.	n.d.	n.d.	n.d.	n.d.	n.d.
Gepo*	0.047^f^ μM	8.30 ± 0.361 μM	0.244 ± 0.040 μM	0.049 ± 0.003 μM	n.d.	n.d.	n.d.	>30 μM

a n.d.: not determined; *ref 35; **ref 25.

b IC_50_, means of two independent measurements ± SD.

c DNA gyrase supercoiling high-throughput microtiter plate-based assay.

d TopoIV relaxation high-throughput microtiter plate-based assay.

e RA, means ± SD of residual activity (%) at 10 μM compound concentration from three independent experiments.

f DNA gyrase supercoiling gel-based assay.

g DNA TopoIV relaxation gel-based assay.

h IC_50_ determination not possible due to solubility issues at higher concentrations.

The potency and activity of compounds was compared to compound **2** (Table 1, Figure 3) that contains the same linker-RHS fragment as most of the compounds in this new series. Effects of various modifications/substitutions compared to compound **2** are shown in Figure 4. A replacement of the naphthyridine ring in **2** for an unsubstituted quinoline ring in **16** lead to diminished antibacterial potency across all tested bacteria. It has been previously established that the methoxy group bound to the naphthyridine intercalating LHS, in **2**, importantly influences the observed DNA asymmetry.^13^ As has been noted in previous studies, LHS fragments containing other polar groups at position 2 similarly disturb the symmetry of the DNA molecule, thus stabilizing the intercalation between the DNA and the NBTI.^10,11^ Introduction of such groups at position 2, e.g., cyano group in **6** or carbonyl group in **13**, indeed resulted in good antibacterial activity across both Gram-positive and Gram-negative species. It should be noted that both **6** and **13** display lower MIC values and similar on-target potencies than the reference compound **2**. The improvement of antibacterial activity is evident across most tested bacteria, with 2- to 4-fold improvement in MIC in *S. aureus* and 4- to 8-fold improvement in MIC in *E. coli*. The most prominent improvement of MIC, more than 128-fold, is in *A. baumannii*, where **2** showed no activity at all (MIC > 128 μg/mL), whereas both **6** and **13** display a MIC value of 0.125 μg/mL. Compounds **18** and **19**, which contain either an amino or hydroxy group at position 2, however show diminished potency relative to compound **2**.

**Figure 4:**
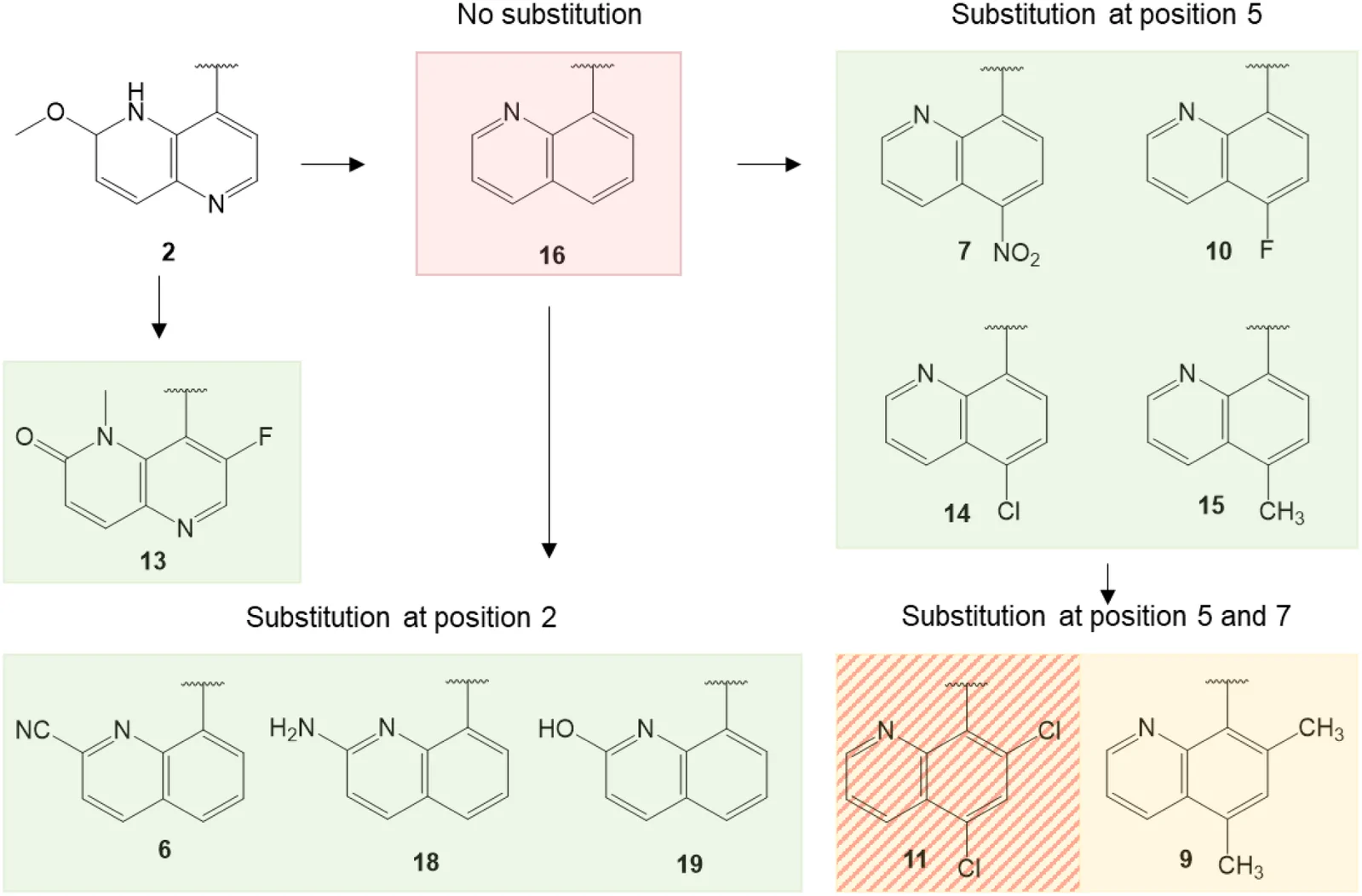
Chemical modifications and substitutions on the naphthyridine/quinoline ring. Red signifies negative change, yellow signifies a neutral change, and green a positive change in terms of antibacterial activity against *S. aureus* and *E. coli* and overall spectrum.

The introduction of substituents at position 5 on the quinoline ring, as seen in compounds **7**, **10**, **14**, and **15**, in comparison to the unsubstituted quinoline of **16**, lead to improved antibacterial activity and higher on-target potency, but not as high as in the reference, compound **2**. And additional lipophilic substituent, e.g., methyl or chlorine, at position 7, as seen in **9** and **11**, in respect to **15** and **14,** did not significantly affect activity.

An introduction of a bulkier tricyclic moiety in **21**-**23**, completely diminished antibacterial activity in Gram-negative bacteria—with the exception of very modest activity of **23** in *E. coli*; and produces modest antibacterial effects in *S. aureus* species.

A replacement of the classic quinoline/naphthyridine LHS for a triazole-conjugated monocycle in **26**-**28** yields compounds with very low antibacterial activity in both Gram-positive and Gram-negative species, therefore on-target potencies were not determined. The introduction of the carbonyl group into **13** may also influence the conformational rotation of the compound that might explain its excellent on-target potency.

Compounds **20** and **25** share their structural similarity with gepotidacin: **20** contains the LHS-linker moiety of gepotidacin combined with our previously published 4-bromo-3,5-difluorobenzyl RHS, whereas **25** contains gepotidacin’s RHS moiety in combination with the LHS and linker from **13**.^46^ The antibacterial activities of gepotidacin “hybrid” molecules were similar to that of gepotidacin in terms of MICs and slightly lower than that of **13** (Table 2). Compound **20** displayed slightly lower MIC values than **1**, which also contains the same linker-RHS combination and was identified as the most active compound from our previous series.

Overall, compound **13** showed most promise, in terms of antibacterial activity, as well as on-target potency. While the IC_50_ value of **13** against *S. aureus* DNA gyrase was the same as that of gepotidacin, 0.047 μM, it differed significantly in experiments with the other tested targets. Compound **13** displayed more balanced on-target inhibition in Gram-positive and Gram-negative bacterial targets compared to gepotidacin, as well as a broader antibacterial spectrum.

Compound **4**, which contains a 1-hydroxyethylene cyclohexyl linker, was identified as a potential lead compound in our previous series, since it displayed a good balance between activity and safety linker.^25^ Its antibacterial activity against Gram-negative bacteria was similar to **1**, which contains the aminopiperidine linker, and superior to **2**, which contains oxymethylene cyclohexyl linker. The hERG IC_50_ value of **4** was 10-fold higher than that of **1** and comparable to **2**, making it the most balanced compound of the previous series. Compound **24** was therefore synthesized using the same linker-RHS scaffold as in **4** (1-hydroxyethylene cyclohexyl linker and 4-bromo-3,5-difluorobenzyl RHS), combined with the new 7-fluoro-1-methyl-1,5-naphthyridin-2­(1*H*)-one LHS, found in **13**. Based on our previous observations, we expected **24** to be the most active compound of the series, however surprisingly both its MIC values and on-target potency were actually lower than in **13**.

### 2.7 *In Vitro* Safety Evaluation

The *in vitro* safety profile of the compounds was determined by cytotoxicity assessment on HepG2 liver cancer cell line, HUVEC endothelial cell line, and by inhibition of the hERG potassium ion channels. The cytotoxicity on HepG2 and HUVEC cell lines was determined by evaluating the effect of the compounds on cell metabolic activity (Table 2). Most quinolone/naphthyridine-based NBTIs displayed residual activity below 50% at 50 μM, therefore IC_50_ was determined. An exception was **6**, which displayed good residual activity, around 100%, in both cell lines. In general, the cytotoxic IC_50_ values against the HepG2 and HUVEC cell lines were in the micromolar range, comparable to **2** and importantly, much higher than the corresponding *S. aureus* MIC values (Table S4, Supporting Information). The average difference between the MICs and cell-based toxicity for the series was > 50-fold, with a > 2000-fold difference in **13**, illustrating the compound’s potential as an early-stage lead compound for the treatment of Gram-positive pathogen-associated infections, however further preclinical evaluations and physicochemical modifications would still be needed. The differences between the cytotoxic IC_50_ and the MIC values against Gram-negative bacteria, however, were much lower, indicating these compounds would likely cause toxic side effects if used for infections caused by Gram-negative bacteria (Table S4, Supporting Information). Compound **13** could be an exception to this, since its HepG2 IC_50_ was 35.94 ± 0.71 μM and the HUVEC IC_50_ equaled 46.52 ± 0.27 μM, whereas its MIC value on *E. coli* was 0.25 μg/mL or 0.49 μM, which indicates an approximately 100-fold difference between the compound’s cytotoxicity and antibacterial activity (Table S4). Other exceptions would be **6**, **20**, **24**, and **25**, all of which displayed residual activity above 50% at 50 μM, as well as MIC values below 1 μg/mL in both *A. baumannii* and *E. coli*.

### 2.8 hERG Binding Assessment

It is known that NBTIs can suffer from an undesirable inhibition of the hERG potassium channels in the heart, resulting in potentially lethal cardiotoxicity.^20,25,26,47–49^ This unwanted toxic effect is commonly related to the basicity and lipophilicity of the compounds, stemming from their structural features, e.g., aminopiperidine linker and aromatic LHS and RHS moieties.^20,49^ Accordingly, all newly designed NBTI compounds are routinely evaluated for their hERG inhibition. In our previous studies, we demonstrated that modification of the linker region can substantially reduce hERG inhibition. Replacement of the aminopiperidine linker (**1**) with less basic oxymethylene cyclohexyl (**2**), oxymethylene tetrahydropyranyl (**3**), and hydroxyethylene cyclohexyl (**4**) linkers resulted in markedly improved hERG profiles and retained antibacterial activity, however, the desired safety threshold was not achieved. Therefore, in the present study, we investigate whether further optimization of the DNA-intercalating LHS moiety could provide additional reductions in hERG liability while maintaining favorable antibacterial activity.

The hERG IC_50_ values of this series of compounds are summarized in Table 2 and reveal several noteworthy trends. Among the quinoline- and naphthyridine-based compounds, hERG binding varied considerably, despite similar core structures. Compounds **9** and **11** exhibited some of the highest hERG IC_50_ values in the series, 9.38 and 12.22 μM, respectively. Compounds **14** and **15**, lacking the substituent at position 7 on the quinoline ring, however, displayed substantially lower values, 2.41 and 0.75 μM, respectively. These results suggest that the spatial arrangement of substituents on the LHS scaffold can influence hERG binding, potentially by altering molecular shape and lipophilicity. Particular attention was given to compound **13**, which emerged as the most potent NBTI of the series. The replacement of the 6-methoxy-1,5-naphthyridine LHS of compound **2** with the 7-fluoro-1-methyl-1,5-naphthyridin-2-one scaffold of **13** was expected to influence physicochemical properties while maintaining favorable DNA intercalation. While activity of compound **13** was enhanced due to this structural modification, the hERG IC_50_ values of compounds **13** and **2** remained comparable, 5.36 and 4.63 μM, respectively. Both compounds remained several-fold more potent hERG binders than gepotidacin, indicating that additional structural modifications of the compounds would be needed in the future to overcome potential cardiotoxicity.

The two gepotidacin-inspired hybrid compounds, **20** and **25**, provided additional insight into the role of individual NBTI structural elements. Although both compounds displayed the most improved hERG profiles among the biologically active analogues (IC_50_ = 13.7 μM), they were still far more potent binders than gepotidacin. Compound **20** incorporates the gepotidacin-derived LHS-linker framework together with the 4-bromo-3,5-difluorobenzyl RHS, whereas compound **25** combines the optimized naphthyridinone LHS with the gepotidacin RHS. Neither structural modifications, however, result in a satisfactory reduction of hERG binding. This shows that while careful optimization of the LHS moiety can produce incremental safety gains, the overall safety profile of NBTIs is determined by the combination of all three structural features of NBTIs—the LHS, linker, and the RHS.

Compounds containing the naphthalimide LHS scaffold (**21**-**23**) consistently display pronounced hERG binding, with hERG IC_50_ values ranging from 0.012 to 1.55 μM. This is consistent with larger aromatic surface and increased lipophilicity of the naphthalimide moiety relative to the quinoline and naphthyridine-based LHS scaffolds. Unsurprisingly, **23** has by far the lowest hERG IC_50_ value, 0.012 μM, likely due to the presence of 4-aminopiperidine in the central linker moiety. In fact, it actually has a 10-fold lower hERG IC_50_ value than **1** from our previous series, likely due to the combination of the LHS and linker which contribute to both increased basicity and lipophilicity.

The hERG IC_50_ determination of **6** was unsuccessful, due to the solubility issues in the test buffer. The cell-based toxicity assays, however, suggest that **6** does not display high cytotoxicity, with its high residual activity at the tested concentration (108.59 ± 9.97% in HepG2 and 99.40 ± 5.19% in HUVEC cells).

Overall, the results of hERG binding assay of this series of compounds indicate that further optimization will still be required to achieve clinically desirable cardiac safety margins. The data further suggest that successful mitigation of hERG liability will require simultaneous optimization of the LHS, linker, and RHS regions.^41–45^

### 2.9 Zebrafish Embryo Toxicity Study and Lethal Infection Model

To assess the toxicological profile of the most promising compounds of the series, i.e., **6**, **13**, **20**, **24**, and **25**, and establish their LC_50_ values, the fish embryo acute toxicity (FET) assay was performed. The zebrafish embryos were exposed to increasing concentrations of the compounds and daily examinations for signs of toxicity were conducted up to 120 h postfertilization (Figure 4). Due to their genetic, molecular, and immunological similarity to humans, as well as highly correlated response to pharmaceuticals, zebrafish embryos present a suitable model for early-stage toxicity studies.^50^ Additionally, zebrafish embryos express a hERG orthologue protein and display sensitivity toward a range of QT-prolonging drugs and may be used for early-stage screening of hERG inhibition, although they may not fully recapitulate human electrophysiological responses.^51^

All of the tested compounds showed no lethality in any of the tested concentrations (Figure 5A). Compounds **13** and **20** exhibited signs of sublethal toxicity above 2 μg/mL and above 10 μg/mL, respectively. This toxic effect was evidenced by lowered heart rate and liver necrosis in **13** (Figure 5B), as well as scoliosis, pericardial edema, and impaired growth in **20**. The visual representation of the effects of compound **20** and other tested compounds on zebrafish embryos is presented in Figure S1, Supporting Information. The groups of zebrafish embryos exposed to compounds **20**, **24**, and **25** displayed a lowered heart rate with no other signs of toxicity. This occurred at concentrations above 6.25 μg/mL for **20** and **24** and above 10 μg/mL for **25**. Since heart rate was quantified and analyzed at the group level, this represents a significant reduction in mean heart rate rather than an effect observed in every individual embryo. If we compare the gepotidacin “hybrids”, compound **25** performed better on the FET test, showing no toxicity in any tested concentration. This difference might be due to structural differences between **20** and **25**, namely the presence of an aminopiperidine linker and significantly more lipophilic RHS in **20**. It is, however, interesting that there was almost no difference in their hERG IC_50_ values, potentially indicating that **20** might affect other toxicity pathways. Compound **6** displayed no toxicity at all in any of the tested concentrations making it the least toxic compound of the new series.

**Figure 5:**
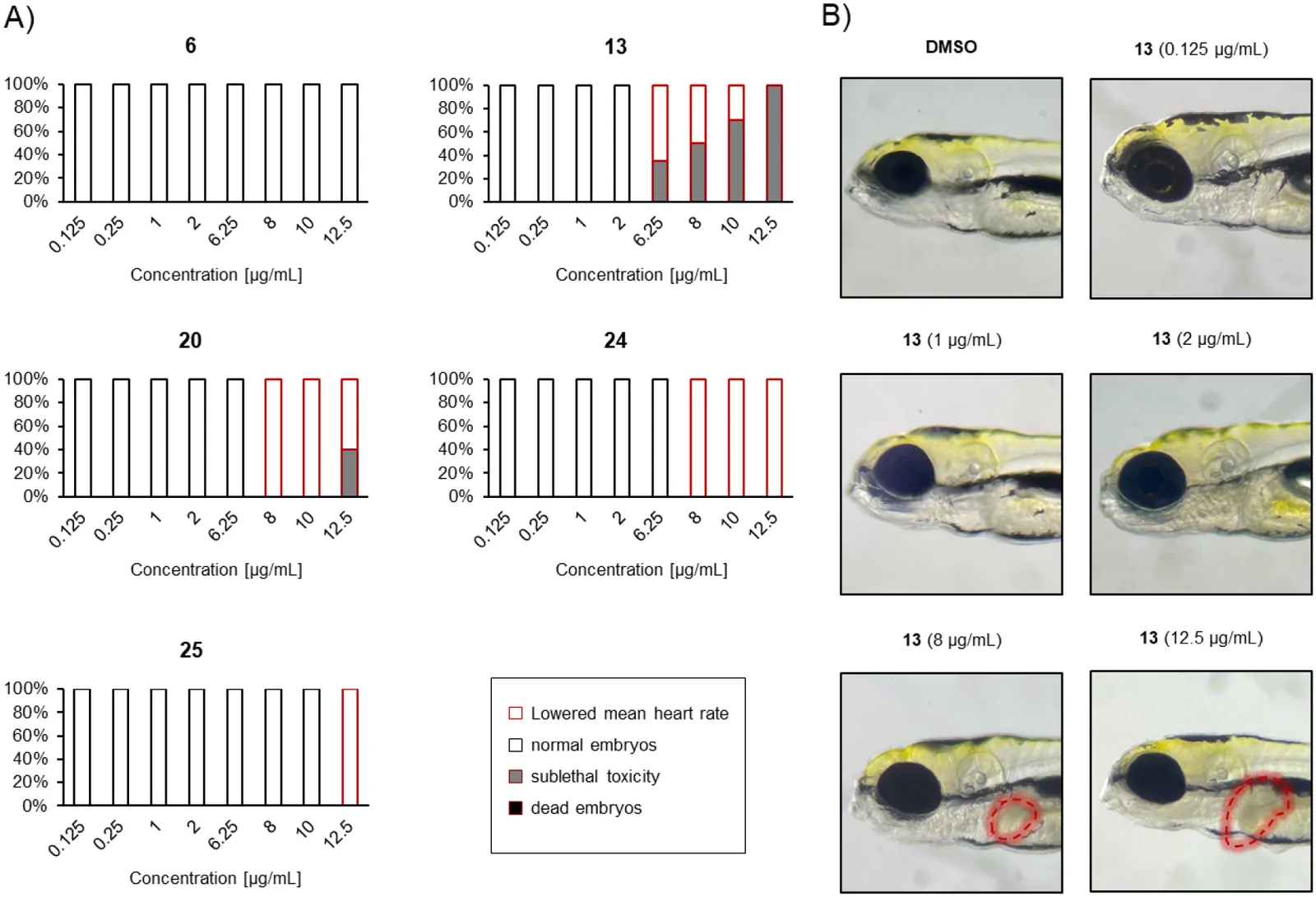
*In vivo* toxicity of **6**, **13**, **20**, **24**, and **25** was evaluated in wild-type zebrafish embryos. Twenty-four embryos were used per group, the experiment was performed in triplicate. A) Dose- dependent toxicity of compound **13**. B) Sublethal toxicity signs of **13** in zebrafish embryos (dotted: liver necrosis). Figure has been cropped to shape.

Compared to our previously disclosed compounds (**1**-**4**), this new series of NBTIs definitely showed improvement in terms of lowering developmental toxicity (Tables S5 and S6 in Supporting Information). Compound **2**, which contains the oxymethylene cyclohexyl linker, was one of the most active compounds in terms of antibacterial activity in our previous series and performed significantly worse in the FET test compared to its new NBTI analogues (i.e., **6**, **13**, and **25**). Although **13** showed modest toxicity in FET, it did not induce lethality at any tested concentration and is considered safe at concentrations up to 2 μg/mL. Compound **2**, however, induced lethality above 6.25 μg/mL and was only considered safe at concentrations up to 0.25 μg/mL. Additionally, **4** that contained the 1-hydroxyethylene cyclohexyl linker also displayed higher toxicity than its analogue **24**. A comparison of **1** and **20**, which both contain the aminopiperidine linker, showcases a drastic drop of toxicity of **20** compared to **1**. Compound **20** induces only sublethal toxicity at concentrations above 10 μg/mL, whereas compound **1** is toxic even at the lowest tested concentration. This shows that the choice of LHS moiety can have a significant effect on overall toxicity.

Compound **13** has the best biological activity in the series, with the lowest MIC values in both Gram-positive and Gram-negative bacteria, similar to the activity of **1** and **2**. Besides showing good antibacterial activity it also displays an improved safety profile compared to both **1** and **2**. Although **13** displays sublethal toxicity at concentrations above 2 μg/mL, its therapeutic window in Gram-negative bacteria is between 0.25 and 2 μg/mL in *E. coli* and 0.125 and 2 μg/mL in *A. baumannii*. This is significantly narrower than the therapeutic window of gepotidacin. In *S. aureus*, however, where the MIC value of **13** is 0.008 μg/mL, concentrations of **13** as high as 250 × MIC would still be considered safe; these findings illustrate the substantial therapeutic window of compound **13**, especially in Gram-positive bacteria. In light of this broad therapeutic window, we proceeded to establish a lethal *S. aureus* ATCC25923 infection model in zebrafish. Fluorescently labeled bacterial cells were injected into embryos (Figure 6A).^52^ Following infection, the embryos were exposed to varying concentrations of compound **13** (MIC = 0.008 μg/mL) and monitored for survival and infection-related signs over a 4-day period postinfection (dpi). Ciprofloxacin was used as an internal standard, at the concentration 2 × MIC (MIC = 0.25 μg/mL). The survival of the ciprofloxacin group was 80% at the end of the experiment. Our results demonstrated that the administered compound effectively prevented zebrafish embryos from succumbing to a lethal *S. aureus* infection during the treatment period. The protective effect was notably evident within the first 24 h postinfection, as shown in Figure 5A. Untreated embryos exhibited strong red fluorescence, indicative of labeled *S. aureus*, by 24 h postinfection and succumbed progressively by 4 dpi. In contrast, embryos treated with **13** showed significantly reduced bacterial loads and survived through the entire 4-day monitoring period (Figure 6B).

**Figure 6:**
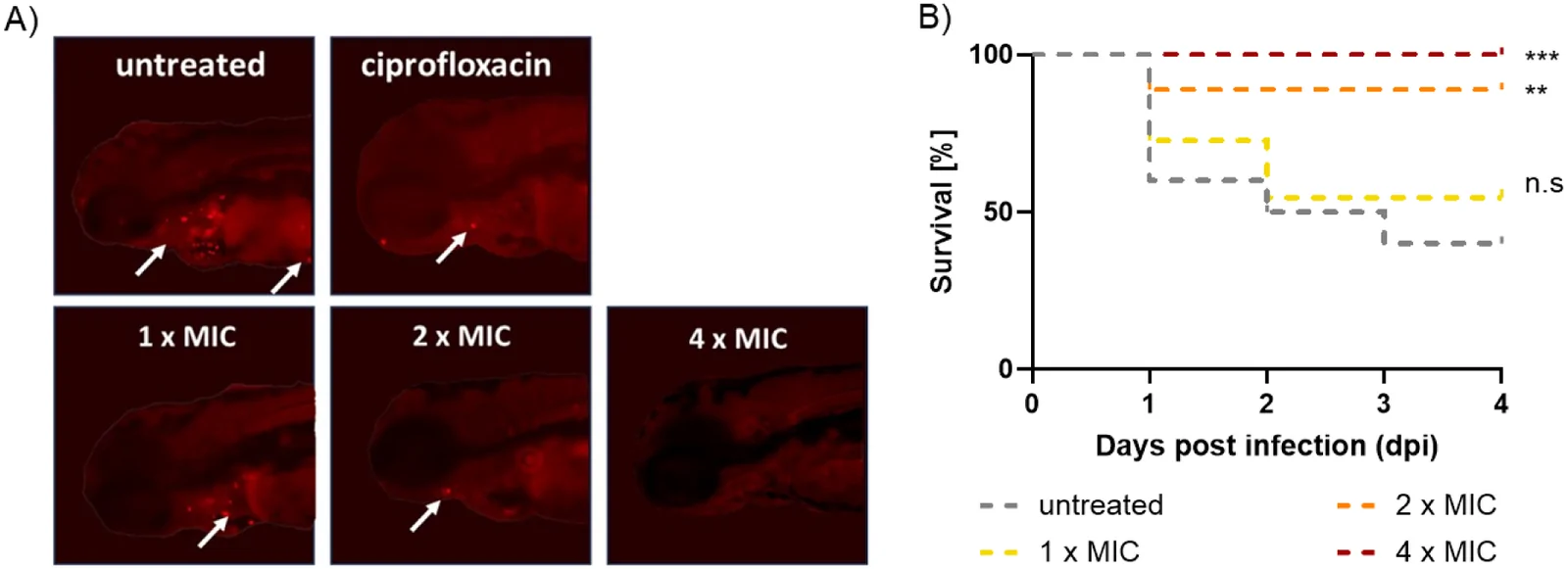
**13** successfully cured *S. aureus* infection in zebrafish embryos. Twenty embryos were used per group, the experiment was performed in triplicate. A) Bacterial loads, as depicted by red fluorescence corresponding to bacterial cells (indicated by white arrows) 24 h postinfection. B) Survival comparison between embryos exposed to **13** and control embryos shows a marked improvement in survival for the treated embryos. ***p < 0.001, **p < 0.01, n.s., not significant.

### 2.10 Time-Kill Kinetics, Postantibiotic Effect, and Resistance Development Assays

To evaluate the kinetic properties of this series of NBTIs in bacteria, the most promising compound, **13**, was further evaluated in additional assays. Due to structural resemblance, comparable results would be expected for other compounds in the series. We conducted three types of experiments—time-kill assay, postantibiotic effect determination, and resistance development study, across three bacterial species: *A. baumannii*, *E. coli*, and MRSA. Gepotidacin was used as a positive control in all the assays.

#### 2.10.1 Time-Kill Assay

We examined the bactericidal kinetics of **13** by performing time-kill assays in three bacterial species, i.e., *A. baumannii*, *E. coli*, and MRSA. The assay was conducted at the concentration of 1 × MIC and 8 × MIC, while gepotidacin was used as a positive control. Both gepotidacin and **13** showed a dose-dependent reduction of bacteria in all tested bacteria (Figure 7A–C). At the concentration of 1 × MIC, both gepotidacin and **13** showed inhibition of bacterial growth. Regrowth was observed after 6 h for both **13** and gepotidacin in *A. baumannii*, after 6 h with gepotidacin and 8 h with **13** in *E. coli*, and after 24 h for both **13** and gepotidacin in MRSA. At the concentration 8 × MIC of **13** and gepotidacin, no bacterial recovery was observed after 24 h in either of tested bacterial species, confirming the bactericidal effect of these compounds.

**Figure 7:**
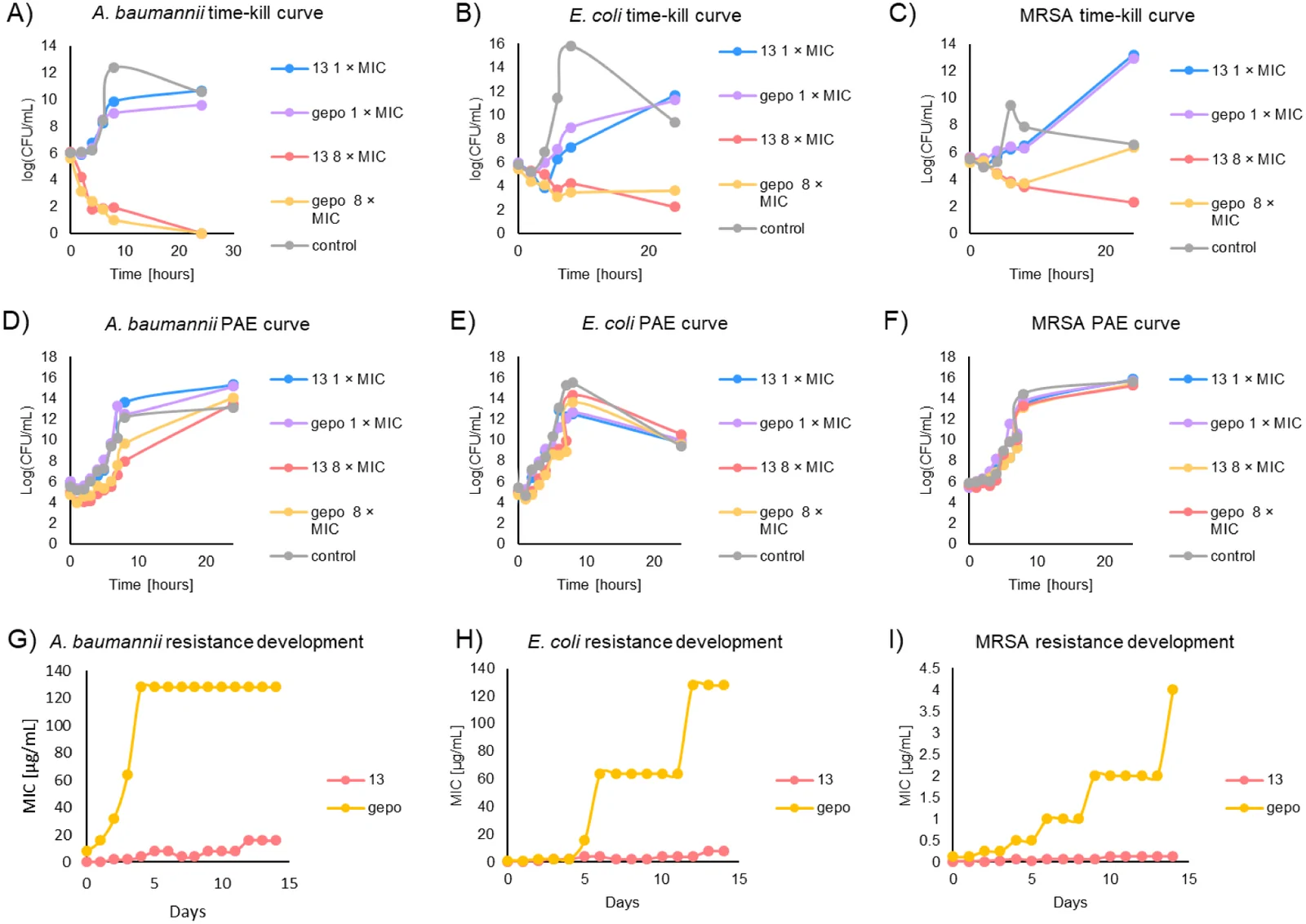
Antibacterial kinetic assays. A–C) Time-kill assay curves. D–F) Postantibiotic effect (PAE) curves. G–I) Resistance development curves.

#### 2.10.2 Postantibiotic Effect

To determine the postantibiotic effect (PAE), three bacterial cultures, *A. baumannii*, *E. coli*, and MRSA, were briefly exposed to either 1 × MIC or 8 × MIC of compound **13** or gepotidacin, and bacterial recovery was monitored over 24 h. PAE durations are summarized in Supporting Information (Table S7) and Figure 7D–F. In *A. baumannii* PAE was observed in both tested concentrations in both compounds. **13** displayed a slightly longer PAE compared to gepotidacin at both concentrations. In case of *E. coli*, PAE was only observed at the 8 × MIC concentration of both **13** and gepotidacin. In this case, gepotidacin displayed a longer PAE, 2 h compared to 1 h for **13**. No PAE was observed in MRSA, at any concentration.

#### 2.10.3 Resistance Development

To compare the rate at which different bacterial strains develop resistance to compound **13** and gepotidacin, we performed a resistance development study. Three bacterial strains (*A. baumannii*, *E. coli* and MRSA) were exposed to either **13** or gepotidacin and the MIC was determined. The bacteria that were exposed to the concentration of the compounds equal to 1/2 MIC were appropriately diluted and again exposed to the two compounds. This was done daily for 2 weeks. 128 μg/mL was used as a cutoff for MIC determination, since gepotidacin has a clinical susceptibility breakpoint of 8 μg/mL in *E. coli*; therefore, MIC values ≥ 128 μg/mL are at least 1 order of magnitude higher than concentrations considered clinically meaningful.^53^ Thus, compounds with MIC values at or above this level were classified as having no relevant antibacterial activity within a therapeutically applicable concentration range.

As shown in Figure 7G–I, all three bacterial species exhibited increases in MIC values over time. While the MIC increases appeared more pronounced for gepotidacin, it is important to note that its initial MIC values (Day = 0) are higher than those of compound **13** (Table S8, Supporting Information). The fold-changes in MIC of **13** and gepotidacin are shown in Table S2, Supporting Information. After 5 days, the MIC of gepotidacin in *A. baumannii* reached and plateaued at 128 μg/mL. The jump in MIC values for **13** was 32-fold (from 0.5 to 16 μg/mL), whereas the jump for gepotidacin was 16-fold (from 8 to 128 μg/mL). In *E. coli* the jump in MIC values in gepotidacin was significantly greater than that of **13**, 128-fold (from 1 to 128 μg/mL) compared to 16-fold (from 0.5 to 8 μg/mL), respectively. A MIC of gepotidacin above the EUCAST resistance breakpoint of 8 μg/mL was obtained after just 5 days and after 12 days the MIC value of gepotidacin stabilized at 128 μg/mL. This is why the experiment was concluded after 2 weeks, although a longer experiment might be needed to establish resistance phenotypes with higher certainty. In MRSA, the MIC increase over the two-week period was 16-fold (from 0.008 to 0.125 μg/mL) for **13** and 32-fold for gepotidacin (from 0.125 to 4 μg/mL). These results indicate that both compounds show comparable level of resistance development in *A. baumannii* and MRSA, as reflected by similar fold increases in MIC values. However, resistance development in *E. coli* was significantly slower with **13** compared to gepotidacin. Considering slower resistance development and that the starting MIC values of **13** are lower than those of gepotidacin, compound **13** could represent a new lead compound in treating *E. coli* infections, with future optimizations needed in order to satisfy the physiochemical profile of a preclinical candidate.

### 2.11 *In Vitro* Drug Metabolism/ADME Properties

Kinetic solubility and mouse liver microsomal stability were assessed for some of the most promising compounds (i.e., **6**, **7**, **13**, **24**, and **25**). These two assays represent key *in vitro* ADME evaluations prior to progression to *in vivo* studies. The results are summarized in Table 3.

**Table 3:** Kinetic Solubility and Mouse Liver Microsomal Stability of Compounds **6**, **7**, **13**, **24**, and **25**

	Kinetic solubility		Mouse liver microsomal stability			
Compound	Solubility in PBS pH 7.4 (μM)	Solubility class	% Remaining after 1 h	Half-life (min)	Intrinsic clearance Clint (μL/min/mg protein)	Clearance classification
**6**	1.72	Poor	51.57	72.62	19.39	Medium
**7**	<1	Poor	52.16	67.13	20.82	Medium
**13**	**77.40**	**Moderate**	**37.52**	**41.83**	**26.95**	**Medium**
**24**	11.53	Moderate	23.63	18.35	42.85	Medium
**25**	>100	High	45.01	48.96	23.71	Medium

While all five selected compounds exhibited medium stability and intrinsic clearance in a mouse liver microsomal assay, only three of the tested compounds exhibited favorable solubility (i.e., **13**, **24**, and **25**). With further optimization to improve aqueous solubility and pharmacokinetic properties, these compounds may become suitable for oral administration. In the absence of such optimization, parenteral administration (e.g., intravenous) could be considered, although this would still require sufficient solubility for formulation.

LogD_7.4_ and plasma protein binding (PPB) were measured for compound **13** and gepotidacin (Table S9), providing complementary insights into the behavior of the compounds in a biological system. Unsurprisingly, the measured LogD_7.4_ of compound **13** was several-fold higher than that of gepotidacin, with values of 4.00 and −0.0877, respectively, suggesting that solubility, potential off-target effects, and tissue accumulation could be a cause for concern for **13**. The PPB experiment done at 2 μM revealed that only around 1% of **13** is in the form of free drug, compared to 97% for gepotidacin. While high PPB means that only a fraction of compound **13** would be available to actually reach the bacterial target enzymes, it also lowers the potential of hERG binding. However, high PPB also increases risks of displacement drug–drug interactions and may affect clearance, making it harder to maintain a safe therapeutic window. In summary, **13** exhibits a much less favorable *in vitro* kinetic profile, compared to gepotidacin, indicating the need for additional future optimizations of this lead compound.

### 2.12 Matched Molecular Pair Analysis

In hopes of better understanding the structure–property relationship of this series of compounds and how the choice of LHS actually affects activity, matched molecular pair analysis (MMPA) was performed. Prior to performing MMPA, various additional chemical properties were calculated using Chemicalize online platform (i.e., cLogP, cLogD_7.4_, and tPSA). Lipophilic efficiency (LipE) was calculated, as well. Since experimentally determined logD_7.4_ values were only available for compound **13**, calculated cLogD_7.4_ values were used to assess lipophilicity trends across the compound series. While cLogD_7.4_ descriptors are useful for comparative analyses within structurally related compounds, experimentally determined logD_7.4_ values would provide a more reliable basis for evaluating the relationship between lipophilicity and hERG liability. Future studies including experimental logD_7.4_ measurements for a larger set of compounds would enable a more robust assessment of the contribution of lipophilicity to the cardiotoxicity profile of this NBTI series. The IC_50_ values against hERG and *S. aureus* DNA gyrase were used to assess the effects of chemical transformations on activity. Gepotidacin and compound **2** served as references. Since the data set of our novel compounds is relatively small, it limits the statistical robustness of the MMPA. Consequently, the analysis should be interpreted as providing qualitative, directional insight rather than definitive, quantitatively supported conclusions regarding LHS optimization. This limitation is further compounded by the inclusion of transformations significantly larger than the traditionally used single-atom or small substructural substitutions; nevertheless, the analysis could still be successfully performed. MMPA was conducted for compounds sharing the same linker–RHS combination and differing only in the LHS.

The present study has revealed an intricate correlation between these parameters and hERG inhibition, as evidenced by the findings of the matched molecular pair analysis (Figure 8). In general, compounds with higher cLogP and cLogD_7.4_ exhibit stronger hERG inhibition. However, it is imperative to exercise caution when interpreting the MMPA data, as two compounds, **9** and **11** (Table 1), deviate significantly from the cLogP and cLogD_7.4_/hERG correlation. These two compounds exhibit increased bulkiness, evidenced by substituents at position 2 of the LHS. This observation suggests a potential mechanism whereby LHS bulkiness might result in diminished hERG inhibition potency. The tPSA correlation, however, is not as definitive. Generally, it could be hypothesized that an increase in tPSA would lead to a decrease in hERG inhibition; nevertheless, this assumption is contradicted by the data from four compounds (**7**, **9**, **11**, and **18**). Although **9** and **11** may be regarded as outliers due to structural reasons, no such explanation can be proposed for **7** and **18**.

**Figure 8:**
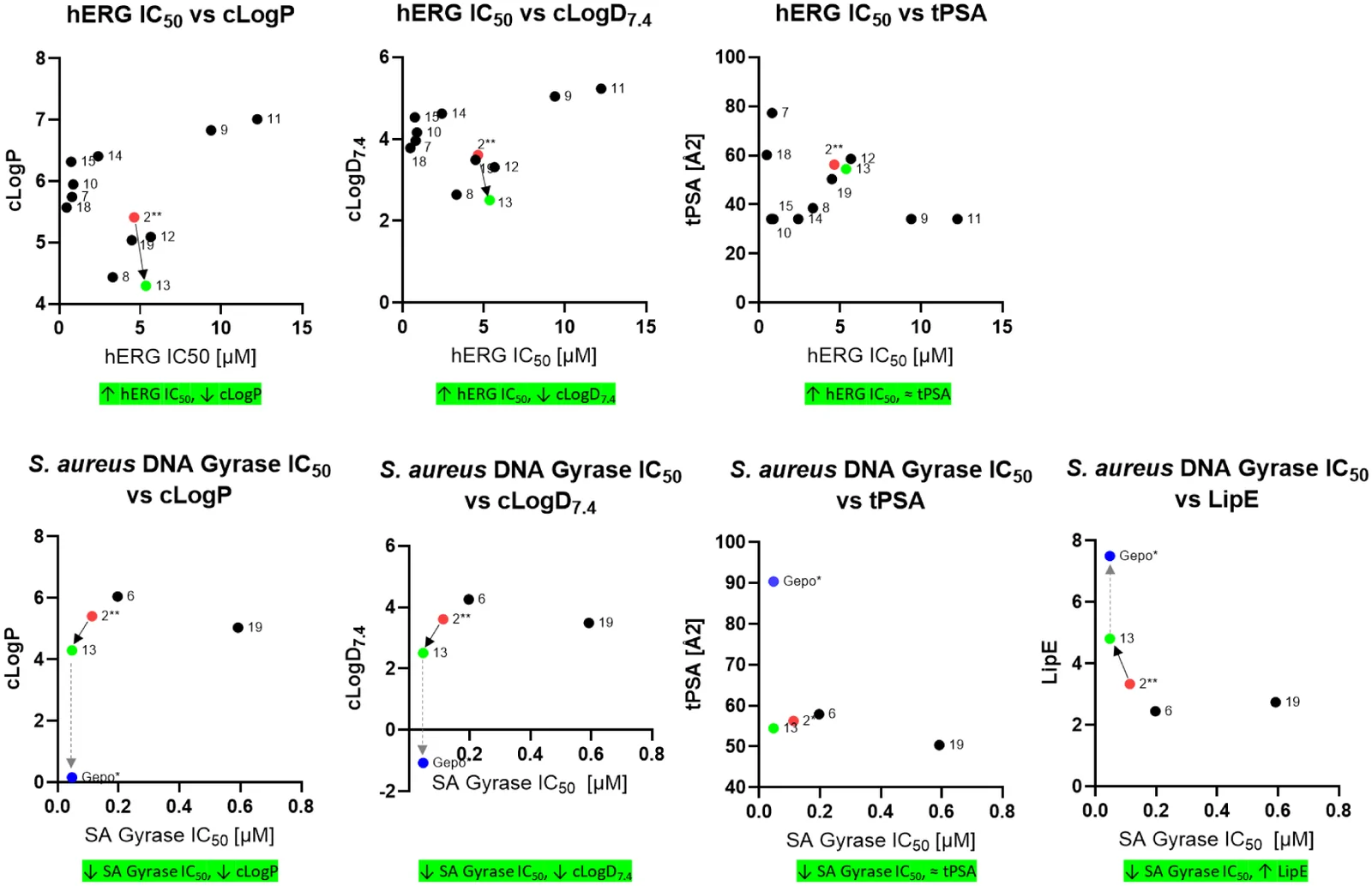
MMP analysis of our new series of NBTIs. Red dot represents reference compound **2**, green dot represents our current lead compound **13**, while blue dot depicts gepotidacin. Black arrow depicts the translation of chemical properties of **13** relative to **2**, whereas gray dotted arrows show the desired direction of future optimizations.

Compound **13** showed lower hERG binding and on-target activity on *S. aureus* DNA gyrase, as well as improved cLogP, tPSA, and logD_7.4_ compared to **2**, illustrating that the modification was indeed beneficial (Figure 8). Although the observed chemical properties of these series of compounds are not yet ideal for a preclinical candidate, the modifications made to the LHS in compound **13** were a step in the right directions, since **13** now more closely resembles the desired physicochemical profile of gepotidacin than our previous early-stage lead, compound **2**. These results show that LHS optimization indeed contributes to overall improvement of the activity profile of novel NBTI compounds.

### 2.13 Molecular Dynamics Insights into DNA Intercalation Geometry and Strand Break Stabilization

To assess the impact of intercalation geometry of the various LHS fragments to the nascent DNA topology as well as to provide a dynamic insight into the investigated DNA gyrase-DNA-NBTIs ternary complexes, a series of all-atom molecular dynamics (MD) simulations were performed. For this purpose, docking poses of compounds **6**, **13**, **20**, **24**, and **25**, as well as gepotidacin in *S. aureus* DNA gyrase (PDB ID: 6Z1A) served as initial NBTI’s configurations. Each system was subjected to fully unrestrained MD production simulations (*NVT* ensemble) in total duration of 500 ns. All simulated compounds displayed a similar, stable intercalation mode over the entire simulation time as exemplified by the calculated RMSD and radius of gyration (Rg) metrics accounting for the overall stability and compactness of each simulated system (Supporting Information Figures S3 and S4).

To further elucidate the structural impact of NBTI binding, the conformational effects on DNA geometry were examined. For this purpose, the obtained MD production trajectories were sampled at 100, 300, and 500 ns, and obtained snapshots were utilized to visually assess the intercalation geometry of NBTI’s LHS fragments and their impact on the overall nascent DNA topology and symmetry (Supporting Information Figure S5). The top view of superimposed DNA structures in the presence and absence of various intercalated NBTI’s LHS fragments reveals a disruption of DNA symmetry, with the intercalated DNA preferentially distorted in one direction, i.e., stabilization of a single-strand DNA break. Mechanistically, all simulated NBTIs act through stabilization of a single-strand DNA break (Supporting Information Figure S6), which finding is strongly corroborated with previous experimental evidence.^13,54^ The stabilization of single-strand DNA cleavage complexes and their catalytically active, i.e., catalytically inactive sites driven by the compound **13** and gepotidacin is depicted in Figure 9. Due to the spatial proximity of the DNA scissile phosphate to the catalytically active site, a compact octahedral Mn^2+^ coordination state can be observed, formed between the scissile phosphate, GyrB residues Asp508 and Asp510, and three water molecules. In contrast, at the catalytically inactive site, the scissile phosphate of the second DNA break is positioned too far from the catalytic Mn^2+^ to participate in coordination; consequently, the octahedral coordination sphere is instead completed by a small cluster of surrounding water molecules. Although MD simulations present a useful insight into the behavior of NBTIs, future experimental structural validation of the newly synthesized compounds in ternary DNA gyrase complexes would be needed to definitively confirm DNA intercalation geometry and strand break stabilization.

**Figure 9:**
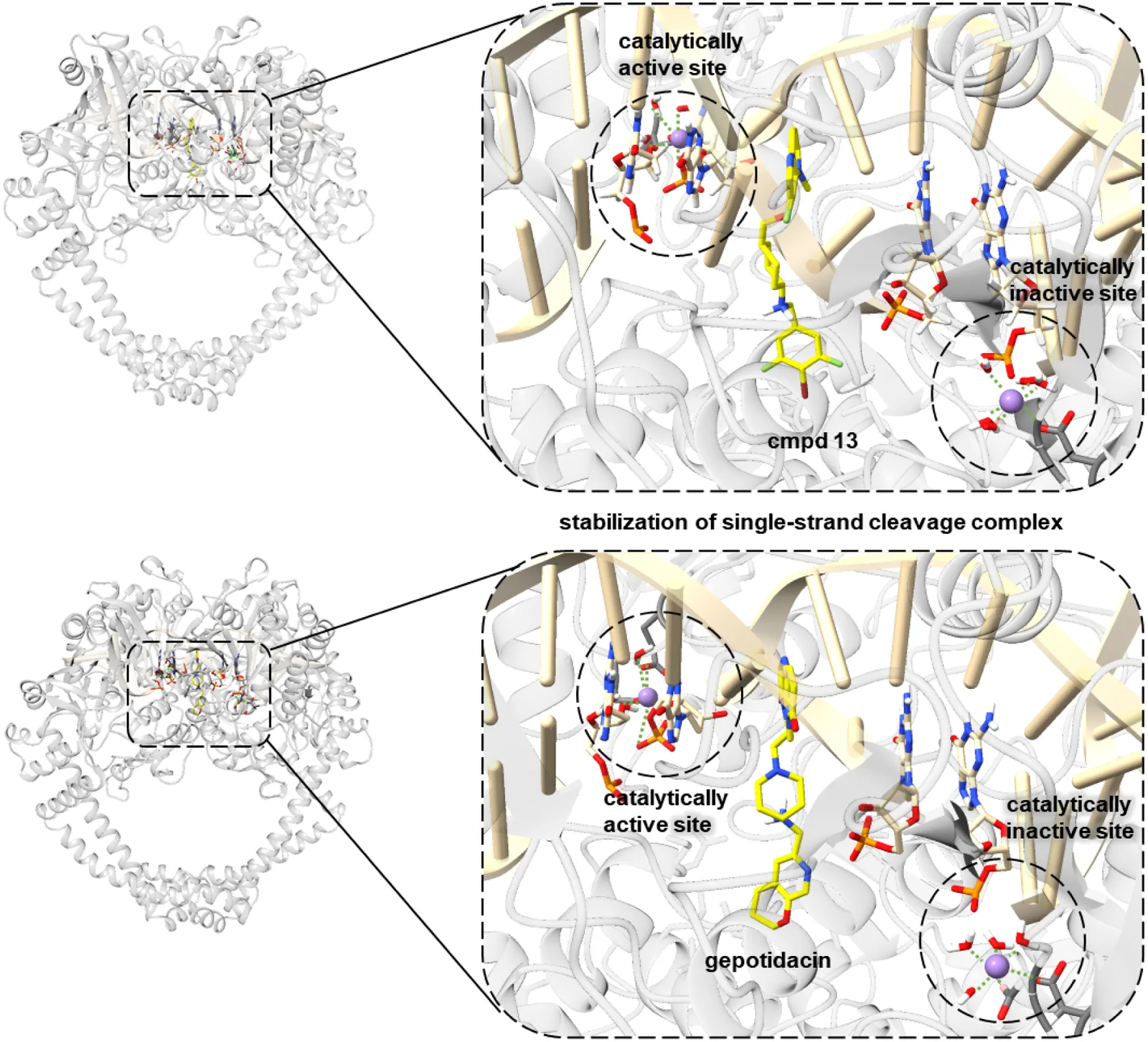
Stabilization of single-strand cleavage complex by compound **13** and gepotidacin. The coordinative bonds are represented in green. Catalytically active (metal–phosphate contact) and inactive sites (no metal–phosphate contact) are circled.

## 3 Conclusions

Using computer-aided drug design along with rational design focused on the DNA-intercalating LHS region of NBTIs, we successfully identified new moieties that enhance enzyme inhibition and antibacterial potency while contributing to improved safety profiles. When combined with the oxymethylene cyclohexyl linker and 4-bromo-3,5-difluorobenzyl RHS, several of the newly developed compounds demonstrated strong on-target activity, reduced hERG inhibition, and acceptable mammalian cytotoxicity. Compound **13** emerged as the most balanced candidate, exhibiting exceptional activity across Gram-positive and some Gram-negative strains, low nanomolar inhibition of both DNA gyrase and TopoIV, and moderately improved safety compared to earlier generations of NBTIs. Compound **13** exhibits a wider range of antibacterial activity compared to gepotidacin, with lower MIC values across the bacterial spectrum and more balanced on-target potency. *In vivo* zebrafish embryo toxicity studies further demonstrated that **13** is well-tolerated at therapeutically relevant concentrations and capable of curing lethal *S. aureus* infections. Its therapeutic window compared to gepotidacin remains more narrow, especially in Gram-negative bacteria.

Several compounds, particularly **13**, **24**, and **25**, exhibited potent inhibition of biofilm formation in *A. baumannii* and *E. coli*, outperforming gepotidacin at equivalent sub-MIC concentrations. Although stimulation of biofilm formation was observed in MRSA at higher concentrations, these effects were moderate and compound-dependent. Mature biofilms remained challenging to eradicate, with only modest inhibition observed, highlighting the inherent difficulty of targeting established biofilm structures. Nevertheless, reductions in bacterial viability across all strains indicate that these NBTIs retain activity even within protective biofilm matrices.

Kinetic antibacterial studies identified compound **13** as a fast-acting agent with strong bactericidal activity at elevated concentrations, comparable to gepotidacin in terms of MIC equivalents, but with absolute more potent activity. Importantly, resistance development studies revealed a slower increase in MIC values for **13** than for gepotidacin across all tested bacterial strains, suggesting a potentially more favorable long-term resistance profile.

Compounds **13**, **24**, and **25** showed moderate metabolic stability and solubility, while compound **13** displayed less favorable pharmacokinetics than gepotidacin due to high lipophilicity and plasma protein binding, indicating the need for further optimization.

Collectively, these findings confirm that LHS engineering is a viable, though not infallible, strategy for mitigating key liabilities of NBTIs and support the advancement of compound **13** or its close analogues toward comprehensive preclinical evaluation as next-generation antibacterial agents.

## 4 Materials and Methods

### 4.1 General Chemical Methods

Starting materials, reagents, and solvents were obtained from commercial sources and used without additional preparation. Thin-layer chromatography (TLC) analysis was used for reaction progress, product isolation, and solvent selection for purification. Analytical TLC was performed on Merck 60 F254 (0.25 mm) silica gel plates, and products were visualized with UV light and spray reagents. Final products were purified either by column chromatography on silica gel 60 (particle size 240–400 mesh) or an automated flash chromatography system using gradient elution (MF = 0.1 M TFA:CH_3_CN, 0 to 100%) (Isolera One, Biotage). Purity and identity were further confirmed by NMR spectroscopy. The resonance frequency for ^1^H and ^13^C in NMR was recorded at 400 and 100 MHz using an AVANCE III 400 spectrometer (Bruker Corporation, Billerica, MA, USA) in CDCl_3_. Chemical shifts (δ) are given in parts per million (ppm), with respect to the deuterated solvent as the internal standard (δH: CDCl_3_ 7.26 ppm), and coupling constants (*J*) in hertz (Hz). Peak multiplicities are given as follows: singlet (s), doublet (d), doublet of doublets (dd), triplet (t), quartet (q), multiplet (m). High-resolution mass spectra were recorded using a LCMS/MS system (Q Exactive Plus; Thermo Scientific, MA, USA). Unless otherwise stated, all compounds had a purity of ≥95% as determined by HPLC on an 1100 system (Thermo Scientific Dionex UltiMate 3000 (Thermo Fisher Scientific, Inc.). Compounds with purity of ≥88% were considered for preliminary biological evaluation. For the general method, a Waters Acquity UPLC HSS C_18_ SB column (2.1 × 50 mm, 1.8 μm) thermostated at 40 °C was used, with injection volume, 5 μL; sample, 0.1–0.2 mg/mL in MeOH; flow rate, 0.4 mL/min; detector λ, 220 and 254 nm; mobile phase A: 0.1% TFA (v/v) in water; mobile phase B: MeCN. Gradient: 0–2 min, 20% B; 2–5 min, 20%–90% B; 5–8 min, 90% B.

#### 4.1.1 Synthesis of Intermediates

7-Fluoro-8-hydroxy-1-methyl-1,5-naphthyridin-2­(1*H*)-one (**xxxii**) was synthesized according to the patent WO 2010/045987 ^37^ and (*R*)-2-((4-aminopiperidin-1-yl)­methyl)-1,2-dihydro-3*H*,8*H*-2a,5,8a-triazaacenaphthylene-3,8-dione (**xxxvii**) was synthesized according to the patent WO 2009/141398.^36^

#### 4.1.2 General Procedure A: Mitsunobu Reaction

The substituted quinolone or other LHS-alcohol (1.2 equiv), an appropriate linker (1.0 equiv), and Ph_3_P (1.5 equiv) were dissolved in dry THF, under argon atmosphere. DIAD (1.5 equiv) was added dropwise at room temperature. The reaction was stirred at 50 °C for 16 h. Then the solvent was evaporated and water (20 mL) was added to the crude product and suspension was extracted with 3 × 10 mL of DCM. The combined organic phases were dried over Na_2_SO_4_, filtered, and evaporated. The crude product was purified by flash chromatography on silica gel to afford the title compounds.

#### 4.1.3 General Procedure B: Boc Deprotection

To the solution of the appropriate compounds in DCM, TFA (19 equiv) was added. The reaction mixture was stirred at room temperature overnight. To the reaction mixture, water was added and the pH was adjusted to about 12 with 4 M NaOH. The suspension was extracted with DCM. The combined organic phases were dried over Na_2_SO_4_, filtered, and evaporated to give the title compound.

#### 4.1.4 General Procedure C: Reductive Amination

To a solution of suitable amine (va, via, or ixa) (1 equiv) and aldehyde (1.1 equiv) in methanol, acetic acid (2 drops) was added and the mixture was stirred for 1 h at 60 °C. To the reaction mixture, NaCNBH_3_ (4 equiv) was added and the reaction was stirred for 16 h at 60 °C. Then the solvent was evaporated and the crude product was dissolved in ethyl acetate (20 mL) and washed with 3 × 10 mL of 0.1 M NaOH. The organic phase was dried over Na_2_SO_4_, filtered, and evaporated. The crude product was purified by flash chromatography on silica gel to give the title compounds.

#### 4.1.5 General Procedure D: Preparation of Azides from Primary Alcohols

The primary alcohol (1.05 equiv) and TEA (2.5–4 equiv) were dissolved in DCM (10 mL). The mixture was cooled to 0 °C, methanesulfonyl chloride (1.2 equiv) was added and the mixture was stirred for 1 h. The crude intermediate was obtained by extraction with citric acid (1 × 10 mL), followed by extraction with NaHCO_3_ (1 × 10 mL). The organic phase was dried over Na_2_SO_4_ and the solvent was evaporated. The intermediate was dissolved in DMF (10 mL), NaN_3_ (1.2 equiv) was added and the reaction was stirred at room temperature for 24 h. The product was obtained by extracting the reaction mixture with water (10 mL) and Et_2_O (2 × 25 mL), before drying it over Na_2_SO_4_ and evaporating the organic solvent. Due to the high toxicity of azides and their potential for explosions, the reactions were handled in a fume hood with appropriate personal protective equipment and later disposed of according to hazardous waste protocols.

#### 4.1.6 General Procedure E: Click Reaction

The azide (1 equiv) and appropriate alkyne (1.2 equiv) were dissolved in MeOH, 0.25 M CuSO_4_ (0.1 equiv) and 0.25 M ascorbic acid (0.1 equiv) were added. The reaction was stirred at room temperature for 24 h. Then the solvent was evaporated and the crude product was dissolved in DCM (2 × 15 mL) and washed with water (15 mL). The organic phase was dried over Na_2_SO_4_, filtered, and evaporated. The crude product was purified by flash chromatography on silica gel to give the title compounds.

##### 4.1.6.1 Synthesis of (4-((4-Bromo-3,5-difluorobenzyl)­amino)­cyclohexyl)­methanol (**i**)

The title compound was obtained by modifying general procedure C, using (4-aminocyclohexyl)­methanol (500 mg, 3.88 mmol, 1.0 equiv), 4-bromo-3,5-difluorobenzaldehyde (1.02 g, 4.66 mmol, 1.2 equiv), and NaBH­(OAc)_3_ (1.27 g, 5.81 mmol, 1.5 equiv) instead of NaBH_3_CN. The crude product was purified by flash column chromatography (SiO_2_, DCM:MeOH = 50:1) to give **i** (600 mg, 46%). ^1^H NMR (400 MHz, CDCl_3_) δ: 7.01–6.93 (m, 2H), 3.80 (s, 2H), 3.46 (d, *J* = 6.4 Hz, 2H), 2.41 (tt, *J* = 11.0, 3.7 Hz, 1H), 2.04–1.94 (m, 2H), 1.83 (d, *J* = 12.9 Hz, 2H), 1.47 (dtq, *J* = 12.9, 6.4, 3.7 Hz, 1H), 1.12 (m, *J* = 12.6, 12.1, 3.2 Hz, 2H), 0.98 (m, *J* = 13.0, 3.2 Hz, 2H). “Hydroxyl and amine H are exchangeable and not visible in the NMR spectrum.”

##### 4.1.6.2 Synthesis of *tert*-Butyl (4-Bromo-3,5-difluorobenzyl)­(4-(hydroxymethyl)­cyclohexyl)­carbamate (**ii**)

To a solution of **i** (600 mg, 1.8 mmol, 1 equiv) in DCM (20 mL), TEA (250 μL, 1.8 mmol, 1 equiv) and Boc_2_O (420 mg, 1.05 equiv) were added. The reaction was stirred under argon for 16 h at room temperature. The crude product was partitioned between DCM (3 × 20 mL) and 20 mL 10% citric acid to give **ii** in quantitative yield. ^1^H NMR (400 MHz, CDCl_3_) δ: 6.84 (d, *J* = 8.0 Hz, 2H), 4.28 (s, 2H), 3.44 (d, *J* = 6.3 Hz, 2H), 1.84 (d, *J* = 13.1 Hz, 2H), 1.76 (s, 2H), 1.54 (s, 9H), 1.47–1.30 (m, 4H), 1.08 (d, *J* = 14.1 Hz, 2H). “Hydroxyl proton is exchangeable and not visible in the NMR spectrum.”

##### 4.1.6.3 Synthesis of *tert*-Butyl (4-Bromo-3,5-difluorobenzyl)­(4-((imidazo­[1,2-*a*]­pyridin-8-yloxy)­methyl)­cyclohexyl)­carbamate (**iii**)

The title compound was obtained by general procedure A, using imidazo­[1,2-*a*]­pyridin-8-ol (60 mg, 0.41 mmol, 1.2 equiv), **ii** (150 mg, 0.35 mmol, 1.0 equiv), DIAD (105 μL, 0.52 mmol, 1.5 equiv), Ph_3_P (145 mg, 0.52 mmol, 1.5 equiv). The crude product was purified by an automated flash chromatography system using gradient elution (MF = 0.1 M TFA:CH_3_CN, 0 to 100%) (Isolera One, Biotage) to give **iii** (54 mg, 28%). ^1^H NMR (400 MHz, CDCl_3_) δ: 7.76 (d, *J* = 17.1 Hz, 1H), 7.57 (s, 1H), 6.93 (s, 1H), 6.86 (d, *J* = 7.7 Hz, 2H), 6.41 (d, *J* = 8.1 Hz, 1H), 5.00 (q, *J* = 7.1 Hz, 2H), 4.28 (s, 2H), 3.88 (d, *J* = 6.4 Hz, 2H), 2.03 (d, *J* = 9.0 Hz, 2H), 1.76 (d, *J* = 11.9 Hz, 3H), 1.37 (s, 2H), 1.30–1.23 (m, 9H), 1.20–1.07 (m, 3H).

##### 4.1.6.4 Synthesis of *tert*-Butyl (4-Bromo-3,5-difluorobenzyl)­(4-(((5,7-dimethylquinolin-8-yl)­oxy)­methyl)­cyclohexyl)­carbamate (**iv**)

The title compound was obtained by general procedure A, using 5,7-dimethylquinolin-8-ol (73 mg, 0.41 mmol, 1.2 equiv), **ii** (150 mg, 0.35 mmol, 1.0 equiv), DIAD (105 μL, 0.52 mmol, 1.5 equiv), Ph_3_P (145 mg, 0.52 mmol, 1.5 equiv). The crude product was purified by an automated flash chromatography system using gradient elution (MF = 0.1 M TFA:CH_3_CN, 0 to 100%) (Isolera One, Biotage) to give **iv** (35 mg, 17%). ^1^H NMR (400 MHz, CDCl_3_) δ: 8.87 (dd, *J* = 4.2, 1.7 Hz, 1H), 8.22 (dd, *J* = 8.5, 1.7 Hz, 1H), 7.34 (dd, *J* = 8.5, 4.2 Hz, 1H), 7.18 (s, 1H), 6.87 (d, *J* = 7.9 Hz, 2H), 4.30 (s, 2H), 3.99 (d, *J* = 6.6 Hz, 2H), 2.58 (d, *J* = 0.9 Hz, 3H), 2.42 (s, 3H), 2.19–2.07 (m, 2H), 1.98–1.86 (m, 2H), 1.80 (s, 2H), 1.61–1.34 (m, 13H).

##### 4.1.6.5 Synthesis of *tert*-Butyl (4-Bromo-3,5-difluorobenzyl)­(4-(((5-fluoroquinolin-8-yl)­oxy)­methyl)­cyclohexyl)­carbamate (**v**)

The title compound was obtained by general procedure A, using 5-fluoroquinolin-8-ol (119 mg, 0.73 mmol, 1.2 equiv), **ii** (263 mg, 0.61 mmol, 1.0 equiv), DIAD (184 μL, 0.92 mmol, 1.5 equiv), Ph_3_P (254 mg, 0.92 mmol, 1.5 equiv). The crude product was purified by flash column chromatography (SiO_2_, ethyl acetate:hexane = 4:1) to give **v** (187 mg, 53%). ^1^H NMR (400 MHz, CDCl_3_) δ: 8.97 (dd, *J* = 4.2, 1.7 Hz, 1H), 8.36 (dd, *J* = 8.4, 1.8 Hz, 1H), 7.47 (dd, *J* = 8.4, 4.2 Hz, 1H), 7.10 (t, *J* = 9.0 Hz, 1H), 6.90 (dd, *J* = 8.7, 4.7 Hz, 1H), 6.86 (d, *J* = 8.0 Hz, 2H), 4.33 (d, 2H), 3.98 (d, *J* = 6.7 Hz, 2H), 2.16–2.07 (m, 2H), 1.99 (m, 1H), 1.81 (d, *J* = 11.7 Hz, 2H), 1.60–1.31 (m, 14H).

##### 4.1.6.6 Synthesis of *tert*-Butyl (4-Bromo-3,5-difluorobenzyl)­(4-(((5,7-dichloroquinolin-8-yl)­oxy)­methyl)­cyclohexyl)­carbamate (**vi**)

The title compound was obtained by general procedure A, using 5,7-dichloroquinolin-8-ol (156 mg, 0.73 mmol, 1.2 equiv), **ii** (263 mg, 0.61 mmol, 1.0 equiv), DIAD (184 μL, 0.92 mmol, 1.5 equiv), Ph_3_P (254 mg, 0.92 mmol, 1.5 equiv). The crude product was purified by an automated flash chromatography system using gradient elution (MF = 0.1 M TFA:CH_3_CN, 0 to 100%) (Isolera One, Biotage) to give **vi** (200 mg, 52%). ^1^H NMR (400 MHz, CDCl_3_) δ: 8.95 (dd, *J* = 4.2, 1.7 Hz, 1H), 8.48 (dd, *J* = 8.6, 1.7 Hz, 1H), 7.61 (s, 1H), 7.50 (dd, *J* = 8.6, 4.2 Hz, 1H), 6.87 (d, *J* = 8.0 Hz, 2H), 4.44–4.25 (m, 2H), 4.14 (dd, *J* = 6.8, 4.4 Hz, 2H), 2.20–2.09 (m, 2H), 1.96–1.75 (m, 3H), 1.60–1.33 (m, 13H).

##### 4.1.6.7 Synthesis of *tert-*Butyl (4-(((5-Acetyl-4-oxo-1,2-dihydro-4*H*-pyrrolo­[3,2,1-ij]­quinolin-6-yl)­oxy)­methyl)­cyclohexyl)­(4-bromo-3,5-difluorobenzyl)­carbamate (**vii**)

The title compound was obtained by general procedure A, using 5-acetyl-6-hydroxy-1,2-dihydro-4*H*-pyrrolo­[3,2,1-ij]­quinolin-4-one (176 mg, 0.55 mmol, 1.2 equiv), **ii** (200 mg, 0.46 mmol, 1.0 equiv), DIAD (141 μL, 0.69 mmol, 1.5 equiv), Ph_3_P (195 mg, 0.69 mmol, 1.5 equiv). The crude product was purified by an automated flash chromatography system using gradient elution (MF = 0.1 M TFA:CH_3_CN, 0 to 100%) (Isolera One, Biotage) to give **vii** (176 mg, 59%). ^1^H NMR (400 MHz, CDCl_3_) δ: 7.56 (dd, *J* = 8.1, 1.0 Hz, 1H), 7.37 (dd, *J* = 7.2, 1.1 Hz, 1H), 7.14 (dd, *J* = 8.1, 7.2 Hz, 1H), 6.87 (d, *J* = 7.8 Hz, 2H), 4.42–4.27 (m, 4H), 3.85 (d, *J* = 5.9 Hz, 2H), 3.41 (t, *J* = 8.0 Hz, 2H), 2.65 (s, 3H), 1.94 (d, *J* = 12.7 Hz, 2H), 1.87–1.67 (m, 3H), 1.54–1.36 (m, 13H), 1.30–1.19 (m, 2H).

##### 4.1.6.8 Synthesis of *tert-*Butyl (4-Bromo-3,5-difluorobenzyl)­(4-(((7-fluoro-1-methyl-2-oxo-1,2-dihydroquinolin-8-yl)­oxy)­methyl)­cyclohexyl)­carbamate (**viii**)

The title compound was obtained by general procedure A, using 7-fluoro-8-hydroxy-1-methyl-1,5-naphthyridin-2­(1*H*)-one (**xxxii**) (100 mg, 0.55 mmol, 1.2 equiv), **ii** (200 mg, 0.46 mmol, 1.0 equiv), DIAD (141 μL, 0.69 mmol, 1.5 equiv), Ph_3_P (195 mg, 0.69 mmol, 1.5 equiv). The crude product was purified by an automated flash chromatography system using gradient elution (MF = 0.1 M TFA:CH_3_CN, 0 to 100%) (Isolera One, Biotage) to give **viii** (137 mg, 49%). ^1^H NMR (400 MHz, CDCl_3_) δ: 8.36 (d, *J* = 2.3 Hz, 1H), 7.78 (d, *J* = 9.7 Hz, 1H), 6.85 (dd, *J* = 12.7, 8.6 Hz, 3H), 4.34 (s, 2H), 4.03 (dd, *J* = 6.4, 1.9 Hz, 2H), 3.90 (s, 3H), 2.02–1.95 (m, 2H), 1.84 (d, *J* = 14.5 Hz, 4H), 1.60–1.31 (m, 11H), 1.31–1.17 (m, 4H).

##### 4.1.6.9 Synthesis of *tert*-Butyl (4-Bromo-3,5-difluorobenzyl)­(4-(((5-chloroquinolin-8-yl)­oxy)­methyl)­cyclohexyl)­carbamate (**ix**)

The title compound was obtained by general procedure A, using 5-chloroquinolin-8-ol (105 mg, 0.55 mmol, 1.2 equiv), **ii** (200 mg, 0.46 mmol, 1.0 equiv), DIAD (141 μL, 0.69 mmol, 1.5 equiv), Ph_3_P (195 mg, 0.69 mmol, 1.5 equiv). The crude product was purified by an automated flash chromatography system using gradient elution (MF = 0.1 M TFA:CH_3_CN, 0 to 100%) (Isolera One, Biotage) to give **ix** (97 mg, 35%). ^1^H NMR (400 MHz, CDCl_3_) δ: 8.98 (dd, *J* = 4.2, 1.7 Hz, 1H), 8.52 (dd, *J* = 8.5, 1.7 Hz, 1H), 7.59–7.43 (m, 2H), 6.94 (d, *J* = 8.5 Hz, 1H), 6.85 (d, *J* = 8.0 Hz, 3H), 4.29 (s, 2H), 4.00 (d, *J* = 6.7 Hz, 2H).

##### 4.1.6.10 Synthesis of *tert-*Butyl (4-Bromo-3,5-difluorobenzyl)­(4-(((5-methylquinolin-8-yl)­oxy)­methyl)­cyclohexyl)­carbamate (**x**)

The title compound was obtained by general procedure A, using 5-methylquinolin-8-ol (88 mg, 0.55 mmol, 1.2 equiv), **ii** (200 mg, 0.46 mmol, 1.0 equiv), DIAD (141 μL, 0.69 mmol, 1.5 equiv), Ph_3_P (195 mg, 0.69 mmol, 1.5 equiv). The crude product was purified by an automated flash chromatography system using gradient elution (MF = 0.1 M TFA:CH_3_CN, 0 to 100%) (Isolera One, Biotage) to give **x** (87 mg, 33%). ^1^H NMR (400 MHz, CDCl_3_) δ: 8.78 (d, *J* = 4.3 Hz, 1H), 7.28–7.16 (m, 1H), 7.01 (dd, *J* = 7.8, 1.2 Hz, 1H), 6.86 (d, *J* = 7.8 Hz, 2H), 4.29 (s, 2H), 4.01 (d, *J* = 6.7 Hz, 2H), 2.65 (s, 3H), 2.20–2.06 (m, 2H), 2.06–1.94 (m, 1H), 1.80 (d, *J* = 11.9 Hz, 3H), 1.59–1.30 (m, 16H), 1.30–1.14 (m, 4H).

##### 4.1.6.11 Synthesis of *tert-*Butyl (4-Bromo-3,5-difluorobenzyl)­(4-((quinolin-8-yloxy)­methyl)­cyclohexyl)­carbamate (**xi**)

The title compound was obtained by general procedure A, using quinolin-8-ol (80 mg, 0.55 mmol, 1.2 equiv), **ii** (200 mg, 0.46 mmol, 1.0 equiv), DIAD (141 μL, 0.69 mmol, 1.5 equiv), Ph_3_P (195 mg, 0.69 mmol, 1.5 equiv). The crude product was purified by an automated flash chromatography system using gradient elution (MF = 0.1 M TFA:CH_3_CN, 0 to 100%) (Isolera One, Biotage) to give **xi** (130 mg, 44%). ^1^H NMR (400 MHz, CDCl_3_) δ: 8.93 (dd, *J* = 4.2, 1.7 Hz, 1H), 8.10 (dd, *J* = 8.3, 1.7 Hz, 1H), 7.40–7.29 (m, 2H), 7.02 (dd, *J* = 7.6, 1.4 Hz, 1H), 6.86 (d, *J* = 8.1 Hz, 2H), 4.29 (s, 2H), 4.11 (m, 1H), 2.13 (dt, *J* = 14.2, 3.5 Hz, 2H), 1.79 (s, 2H), 1.58–1.32 (m, 14H), 1.25 (t, *J* = 7.1 Hz, 2H).

##### 4.1.6.12 Synthesis of *tert-*Butyl (4-(((3-Acetyl-2-methyl-1,1-dioxido-2*H*-thieno­[2,3-*e*]­[1,2]­thiazin-4-yl)­oxy)­methyl)­cyclohexyl)­(4-bromo-3,5-difluorobenzyl)­carbamate (**xii**)

The title compound was obtained by general procedure A, using 1-(4-hydroxy-2-methyl-1,1-dioxido-2*H*-thieno­[2,3-*e*]­[1,2]­thiazin-3-yl)­ethan-1-one (160 mg, 0.55 mmol, 1.2 equiv), **ii** (200 mg, 0.46 mmol, 1.0 equiv), DIAD (141 μL, 0.69 mmol, 1.5 equiv), Ph_3_P (195 mg, 0.69 mmol, 1.5 equiv). The crude product was purified by an automated flash chromatography system using gradient elution (MF = 0.1 M TFA:CH_3_CN, 0 to 100%) (Isolera One, Biotage) to give **xii** (200 mg, 75%). ^1^H NMR (400 MHz, CDCl_3_) δ: 7.62 (d, *J* = 5.2 Hz, 1H), 7.37 (d, *J* = 5.2 Hz, 1H), 6.85 (d, *J* = 8.0 Hz, 2H), 4.29 (s, 2H), 3.89 (s, 3H), 3.85 (d, *J* = 6.1 Hz, 2H), 3.10 (s, 3H), 1.94 (d, *J* = 12.9 Hz, 2H), 1.79 (s, 2H), 1.56 (s, 3H), 1.44 (d, *J* = 51.0 Hz, 13H), 1.27–1.11 (m, 2H).

##### 4.1.6.13 Synthesis of (4-((*tert*-Butoxycarbonyl)­amino)­cyclohexyl)­methylmethanesulfonate (**xiii**)

The title compound was obtained by combining *tert-*butyl (4-(hydroxymethyl)­cyclohexyl)­carbamate (700 mg, 2.9 mmol, 1 equiv) and TEA (404 μL, 2.9 mmol, 1 equiv) in DCM (20 mL). The mixture was cooled on an ice bath to 0 °C and methanesulfonyl chloride (224 μL, 2.9 mmol, 1 equiv) was added dropwise. The reaction was left to warm up to room temperature and stirred overnight. The crude product was partitioned between DCM (3 × 20 mL) and NaHCO_3_ (20 mL). The joint organic phases were dried over Na_2_SO_4_ and the solvent was evaporated to give **xiii** (804 mg, 90%). ^1^H NMR (400 MHz, CDCl_3_) δ: 4.40 (s, 1H), 4.03 (d, *J* = 6.5 Hz, 2H), 3.39 (s, 1H), 3.00 (s, 3H), 2.17–1.99 (m, 2H), 1.92–1.79 (m, 2H), 1.77–1.66 (m, 1H), 1.44 (s, 9H), 1.17–1.05 (m, 4H).

##### 4.1.6.14 Synthesis of *tert-*Butyl (4-(((2-Oxo-1,2-dihydroquinolin-4-yl)­oxy)­methyl)­cyclohexyl)­carbamate (**xiv**)

To a solution of 4-hydroxyquinolin-2­(1*H*)-one (300 mg, 1.27 mmol, 1.0 equiv) in dry DMF sodium hydride was added (1.65 mmol, 1.3 equiv, 60% in mineral oil) under argon. The reaction was stirred for 30 min at 50 °C, when **xiii** (400 mg, 1.27 mmol, 1 equiv) was added and heated to 110 °C for 48 h. The reaction was quenched with water, and extracted with DCM (3 × 15 mL). The organic phase was dried over Na_2_SO_4_, filtered, and evaporated. The crude product was purified by washing with hexane and filtering to give **xiv** (366 mg, 77%). ^1^H NMR (400 MHz, CDCl_3_) δ: 10.58 (s, 1H), 7.90 (dd, *J* = 8.3, 1.4 Hz, 1H), 7.56–7.46 (m, 1H), 7.24–7.16 (m, 1H), 5.93 (s, 1H), 4.48–4.37 (m, 1H), 3.93 (d, *J* = 6.0 Hz, 2H), 3.46 (s, 1H), 2.11 (d, *J* = 11.9 Hz, 2H), 1.97 (d, *J* = 13.0 Hz, 2H), 1.89 (s, 1H), 1.46 (s, 8H), 1.31 (q, *J* = 12.9 Hz, 2H), 1.18 (q, *J* = 11.5 Hz, 2H).

##### 4.1.6.15 Synthesis of *tert*-Butyl (4-(((2-Aminoquinolin-8-yl)­oxy)­methyl)­cyclohexyl)­carbamate (**xv**)

To a solution of 2-aminoquinolin-8-ol (165 mg, 1.5 mmol, 1.0 equiv) in dry DMF sodium hydride was added (1.95 mmol, 1.3 equiv, 60% in mineral oil) under argon. The reaction was stirred for 30 min at 50 °C, when **xiii** (315 mg, 1.5 mmol, 1.0 equiv) was added and heated to 110 °C for 48 h. The reaction was quenched with water, and extracted with DCM (3 × 15 mL). The organic phase was dried over Na_2_SO_4_, filtered, and evaporated. The crude product was purified by washing with hexane and filtering to give **xv** (200 mg, 36%). ^1^H NMR (400 MHz, CDCl_3_) δ: 7.85 (dd, *J* = 8.8, 4.2 Hz, 1H), 7.25–7.10 (m, 2H), 7.02–6.91 (m, 1H), 6.73 (dd, *J* = 8.7, 4.0 Hz, 1H), 4.88 (s, 2H), 4.00 (dd, *J* = 6.7, 4.0 Hz, 2H), 3.43–3.39 (m, 4H), 2.67 (s, 1H), 2.06 (d, *J* = 10.1 Hz, 2H), 1.92 (s, 1H), 1.74–1.47 (m, 8H), 1.24–1.04 (m, 3H).

##### 4.1.6.16 Synthesis of *tert-*Butyl (4-(((2-Hydroxyquinolin-8-yl)­oxy)­methyl)­cyclohexyl)­carbamate (**xvi**)

To a solution of quinoline-2,8-diol (164 mg, 1.5 mmol, 1.0 equiv) in dry DMF sodium hydride was added (1.95 mmol, 1.3 equiv, 60% in mineral oil) under argon. The reaction was stirred for 30 min at 50 °C, when **xiii** (315 mg, 1.5 mmol, 1.0 equiv) was added and heated to 110 °C for 48 h. The reaction was quenched with water, and extracted with DCM (3 × 15 mL). The organic phase was dried over Na_2_SO_4_, filtered, and evaporated. The crude product was purified by washing the solid with hexane and filtering to give **xvi** (120 mg, 21%), which was used without further analysis in the next step.

##### 4.1.6.17 Synthesis of *tert-*Butyl (4-(((2-Cyanoquinolin-8-yl)­oxy)­methyl)­cyclohexyl)­carbamate (**xvii**)

The title compound was obtained by general procedure A, using 8-hydroxyquinoline-2-carbonitrile (400 mg, 2.31 mmol, 1.3 equiv), *tert-*butyl (4-(hydroxymethyl)­cyclohexyl)­carbamate (428 mg, 1.77 mmol, 1.0 equiv), DIAD (535 μL, 2.66 mmol, 1.5 equiv), Ph_3_P (2.66 mmol, 1.5 equiv). The crude product was purified by an automated flash chromatography system using gradient elution (MF = 0.1 M TFA:CH_3_CN, 0 to 100%) (Isolera One, Biotage) to give **xvii** (603 mg, 89%). ^1^H NMR (400 MHz, CDCl_3_) δ: 8.25 (d, *J* = 8.4 Hz, 1H), 7.71 (d, *J* = 8.4 Hz, 1H), 7.60 (t, *J* = 8.0 Hz, 1H), 7.43 (dd, *J* = 8.3, 1.2 Hz, 1H), 7.12 (dd, *J* = 7.8, 1.2 Hz, 1H), 6.34 (s, 1H), 4.45 (s, 1H), 4.02 (d, *J* = 6.6 Hz, 2H), 3.47 (s, 1H), 2.12 (d, *J* = 8.9 Hz, 4H), 1.45 (s, 9H), 1.25–1.18 (m, 4H).

##### 4.1.6.18 Synthesis of *tert-*Butyl (4-(((5-Nitroquinolin-8-yl)­oxy)­methyl)­cyclohexyl)­carbamate (**xviii**)

The title compound was obtained by general procedure A, using 5-nitroquinolin-8-ol (450 mg, 2.36 mmol, 1.2 equiv), *tert-*butyl (4-(hydroxymethyl)­cyclohexyl)­carbamate (450 mg, 1.96 mmol, 1.0 equiv), DIAD (560 μL, 2.94 mmol, 1.5 equiv), Ph_3_P (775 mg, 2.94 mmol, 1.5 equiv). The crude product was purified by automated flash chromatography system to give **xviii** (505 mg, 53%) and directly used without any further analysis.

##### 4.1.6.19 Synthesis of *tert-*Butyl (4-(((3-Fluoro-5-methyl-6-oxo-5,6-dihydro-1,5-naphthyridin-4-yl)­oxy)­methyl)­cyclohexyl)­carbamate (**xix**)

The title compound was obtained by general procedure A, using 7-fluoro-8-hydroxy-1-methyl-1,5-naphthyridin-2­(1*H*)-one (**xxxii**) (300 mg, 1.55 mmol, 1 equiv), *tert-*butyl (4-(hydroxymethyl)­cyclohexyl)­carbamate (380 mg, 1.55 mmol, 1.0 equiv), DIAD (464 μL, 2.31 mmol, 1.5 equiv), Ph_3_P (640 mg, 2.31 mmol, 1.5 equiv). The crude product was purified by an automated flash chromatography system using gradient elution (MF = 0.1 M TFA:CH_3_CN, 0 to 100%) (Isolera One, Biotage) to give **xix** (320 mg, 51%) and was used in the next step without any further analysis.

##### 4.1.6.20 Synthesis of 4-((4-Aminocyclohexyl)­methoxy)­quinolin-2­(1*H*)-one (**xx**)

The title compound was obtained by general procedure B, using **xiv** (366 mg, 0.98 mmol). The crude product was purified by an automated flash chromatography system using gradient elution (MF = 0.1 M TFA:CH_3_CN, 0 to 100%) (Isolera One, Biotage) to give **xx** (164 mg, 61%). ^1^H NMR (400 MHz, CDCl_3_) δ: 11.00 (s, 1H), 7.92 (dd, *J* = 8.2, 1.5 Hz, 1H), 7.51 (ddd, *J* = 8.4, 7.2, 1.5 Hz, 1H), 7.30 (d, *J* = 8.2 Hz, 1H), 7.20 (ddd, *J* = 8.2, 7.2, 1.1 Hz, 1H), 5.95 (s, 1H), 3.93 (d, *J* = 6.1 Hz, 2H), 2.69 (d, *J* = 9.2 Hz, 1H), 2.18 (d, *J* = 2.3 Hz, 1H), 2.04–1.87 (m, 5H), 1.29–1.15 (m, 5H).

##### 4.1.6.21 Synthesis of 8-((4-minocyclohexyl)­methoxy)­quinolin-2-amine (**xxi**)

The title compound was obtained by general procedure B, using **xv** (200 mg, 0.53 mmol). The crude product was purified by an automated flash chromatography system to give **xxi** (146 mg, 99%). The product was used in the next step without any further characterization.

##### 4.1.6.22 Synthesis of 8-((4-Aminocyclohexyl)­methoxy)­quinolin-2-ol (**xxii**)

The title compound was obtained by general procedure B, using **xvi** (120 mg, 0.32 mmol). The crude product was purified by an automated flash chromatography system to give **xxii** (87 mg, 99%). ^1^H NMR (400 MHz, CDCl_3_) δ: 7.62 (s, 1H), 7.19–6.95 (m, 2H), 6.88 (d, *J* = 8.2 Hz, 1H), 6.58 (s, 1H), 3.84 (s, 2H), 2.55 (s, 1H), 1.85 (s, 4H), 1.04 (s, 5H).

##### 4.1.6.23 Synthesis of 8-((4-Aminocyclohexyl)­methoxy)­quinoline-2-carbonitrile (**xxiii**)

The title compound was obtained by general procedure B, using **xvii** (603 mg, 1.58 mmol). The crude product was purified by an automated flash chromatography system using gradient elution (MF = 0.1 M TFA:CH_3_CN, 0 to 100%) (Isolera One, Biotage) to give **xxiii** (442 mg, 99%). ^1^H NMR (400 MHz, CDCl_3_) δ: 8.25 (d, *J* = 8.4 Hz, 1H), 7.71 (d, *J* = 8.4 Hz, 1H), 7.60 (t, *J* = 8.0 Hz, 1H), 7.42 (dd, *J* = 8.3, 1.1 Hz, 1H), 7.13 (dd, *J* = 7.9, 1.2 Hz, 1H), 4.04 (d, *J* = 6.3 Hz, 2H), 2.76–2.66 (m, 1H), 2.11–2.05 (m, 2H), 1.97 (d, *J* = 8.0 Hz, 2H), 1.32 (d, *J* = 6.3 Hz, 1H), 1.24–1.13 (m, 4H).

##### 4.1.6.24 Synthesis of 4-(((5-Nitroquinolin-8-yl)­oxy)­methyl)­cyclohexan-1-amine (**xxiv**)

The title compound was obtained by general procedure B, using **xviii** (505 mg, 1.26 mmol). The crude product was purified by an automated flash chromatography system using gradient elution (MF = 0.1 M TFA:CH_3_CN, 0 to 100%) (Isolera One, Biotage) to give **xxiv** (371 mg, 99%). ^1^H NMR (400 MHz, CDCl_3_) δ: 9.23 (dd, *J* = 8.9, 1.7 Hz, 1H), 9.05 (dd, *J* = 4.2, 1.6 Hz, 1H), 8.52 (d, *J* = 8.9 Hz, 1H), 7.68 (dd, *J* = 8.9, 4.1 Hz, 1H), 7.05 (d, *J* = 8.9 Hz, 1H), 4.15 (d, *J* = 6.5 Hz, 2H), 2.75–2.64 (m, 1H), 2.16–2.03 (m, 3H), 1.95 (s, 2H), 1.27 (d, *J* = 6.2 Hz, 1H), 1.20 (t, *J* = 9.5 Hz, 3H).

##### 4.1.6.25 Synthesis of 8-((4-((4-Bromo-3,5-difluorobenzyl)­amino)­cyclohexyl)­methoxy)-7-fluoro-1-methyl-1,5-naphthyridin-2­(1*H*)-one (**xxv**)

The title compound was obtained by general procedure B, using **xix** (320 mg, 0.79 mmol). The crude product was purified by an automated flash chromatography system using gradient elution (MF = 0.1 M TFA:CH_3_CN, 0 to 100%) (Isolera One, Biotage) to give **xxv** (241 mg, 100%). ^1^H NMR (400 MHz, CDCl_3_) δ: 8.37 (d, *J* = 2.4 Hz, 1H), 7.79 (d, *J* = 9.7 Hz, 1H), 6.87 (s, 1H), 4.96 (dd, *J* = 12.6, 6.3 Hz, 1H), 4.04 (dd, *J* = 6.3, 2.1 Hz, 2H), 3.92 (s, 3H), 3.33 (s, 1H), 2.75–2.54 (m, 1H), 1.94 (t, *J* = 9.3 Hz, 4H), 1.23–1.11 (m, 4H).

##### 4.1.6.26 Synthesis of *tert*-Butyl (4-((1,3-Dioxo-1*H*-benzo­[*de*]­isoquinolin-2­(3*H*)-yl)­methyl)­cyclohexyl)­carbamate (**xxvi**)

The title compound was obtained by general procedure A, using 1*H*-benzo­[*de*]­isoquinoline-1,3­(2*H*)-dione (300 mg, 1.5 mmol, 1 equiv), *tert-*butyl (4-(hydroxymethyl)­cyclohexyl)­carbamate (360 mg, 1.5 mmol, 1 equiv), DIAD (234 μL, 2.25 mmol, 1.5 equiv), Ph_3_P (589 mg, 2.25 mmol, 1.5 equiv). The crude product was purified by an automated flash chromatography system using gradient elution (MF = 0.1 M TFA:CH_3_CN, 0 to 100%) (Isolera One, Biotage) to give **xxvi** (560 mg, 91%). ^1^H NMR (400 MHz, CDCl_3_) δ: 8.60 (dd, *J* = 7.3, 1.2 Hz, 2H), 8.22 (dd, *J* = 8.3, 1.1 Hz, 2H), 7.76 (dd, *J* = 8.2, 7.3 Hz, 2H), 4.98 (p, *J* = 6.2 Hz, 1H), 4.09 (d, *J* = 7.2 Hz, 2H), 1.98–1.66 (m, 11H), 1.32–1.17 (m, 9H), 1.14–0.98 (m, 2H).

##### 4.1.6.27 Synthesis of *tert-*Butyl (4-(2-(1,3-Dioxo-1*H*-benzo­[d*e*]­isoquinolin-2­(3*H*)-yl)­ethyl)­cyclohexyl)­carbamate (**xxvii**)

The title compound was obtained by general procedure A, using 1*H*-benzo­[*de*]­isoquinoline-1,3­(2*H*)-dione (200 mg, 1 mmol, 1 equiv), *tert-*butyl (4-(2-hydroxyethyl)­cyclohexyl)­carbamate (255 mg, 1 mmol, 1 equiv), DIAD (156 μL, 1.5 mmol, 1.5 equiv), Ph_3_P (398 mg, 1.5 mmol, 1.5 equiv). The crude product was purified by an automated flash chromatography system using gradient elution (MF = 0.1 M TFA:CH_3_CN, 0 to 100%) (Isolera One, Biotage) to give **xxvii** (355 mg, 84%). ^1^H NMR (400 MHz, CDCl_3_) δ: 8.60 (dd, *J* = 7.3, 1.2 Hz, 2H), 8.21 (dd, *J* = 8.4, 1.1 Hz, 2H), 7.76 (dd, *J* = 8.3, 7.2 Hz, 2H), 5.04–4.88 (m, 1H), 4.27–4.14 (m, 1H), 2.61 (dd, *J* = 9.6, 5.3 Hz, 1H), 1.96–1.83 (m, 4H), 1.69–1.56 (m, 2H), 1.57–1.31 (m, 2H), 1.27 (d, *J* = 6.3 Hz, 10H), 1.11 (d, *J* = 10.7 Hz, 3H).

##### 4.1.6.28 Synthesis of *tert-*Butyl (1-(2-(1,3-Dioxo-1*H*-benzo­[*de*]­isoquinolin-2­(3*H*)-yl)­ethyl)­piperidin-4-yl)­carbamate (**xxviii**)

The title compound was obtained by mixing 1*H*-benzo­[*de*]­isoquinoline-1,3­(2*H*)-dione (334 mg, 1.6 mmol, 1 equiv) and *tert-*butyl (1-(2-aminoethyl)­piperidin-4-yl)­carbamate (429 mg, 1.76 mmol, 1.2 equiv) in ethanol at 70 °C overnight. The mixture was cooled down and the product precipitated out of the solution to give **xxviii** (378 mg, 55%). ^1^H NMR (400 MHz, CDCl_3_) δ: 8.60 (dd, *J* = 7.3, 1.2 Hz, 2H), 8.22 (dd, *J* = 8.4, 1.1 Hz, 2H), 7.76 (dd, *J* = 8.3, 7.2 Hz, 2H), 4.40 (s, 1H), 4.38–4.28 (m, 2H), 3.47 (s, 1H), 2.99 (d, *J* = 11.8 Hz, 3H), 2.72–2.58 (m, 2H), 2.24 (t, *J* = 11.1 Hz, 2H), 1.92 (d, *J* = 12.6 Hz, 2H), 1.44 (s, 12H).

##### 4.1.6.29 Synthesis of 2-((4-Aminocyclohexyl)­methyl)-1*H*-benzo­[*de*]­isoquinoline-1,3­(2*H*)-dione (**xxix**)

The title compound was obtained by general procedure B, using **xxvi** (560 mg, 1.37 mmol) and used in the next step without any further purification to give **xviii** (419 mg, 99%).

##### 4.1.6.30 Synthesis of 2-(2-(4-Aminocyclohexyl)­ethyl)-1*H*-benzo­[*de*]­isoquinoline-1,3­(2*H*)-dione (**xxx**)

The title compound was obtained by general procedure B, using **xxvii** (355 mg, 0.84 mmol) and used in the next step without any further purification to give (270 mg, 9%).

##### 4.1.6.31 Synthesis of 2-(2-(4-Aminopiperidin-1-yl)­ethyl)-1*H*-benzo­[*de*]­isoquinoline-1,3­(2*H*)-dione (**xxxi**)

The title compound was obtained by general procedure B, using **xxviii** (378 mg, 0.89 mmol) and used in the next step without any further purification to give **xxxi** (224 mg, 78%).

##### 4.1.6.32 Synthesis of 7-Fluoro-8-hydroxy-1-methyl-1,5-naphthyridin-2­(1*H*)-one (**xxxii**)

The title compound was synthesized according to the procedure described in the patent WO 2010/045987 to give 2.2 g of **xxxii**. ^1^H NMR (400 MHz, DMSO-*d*_6_) δ 8.24 (d, *J* = 5.9 Hz, 1H), 7.69 (d, *J* = 9.7 Hz, 1H), 6.82 (d, *J* = 9.8 Hz, 1H), 4.02 (s, 3H).

##### 4.1.6.33 Synthesis of 8-Bromo-7-fluoro-1-methyl-1,5-naphthyridin-2­(1*H*)-one (**xxxiii**)

The title compound was synthesized by mixing **xxxii** (3 g, 20.6 mmol, 1 equiv) with phosphorus bromide (1.5 mL, 22.6 mmol, 1.1 equiv) at 0 °C under argon and letting it gradually warm up to room temperature overnight. The crude mixture was evaporated and purified using column chromatography (MF = DCM:MeOH = 50/1) to give **xxxiii** (2.8 g, 53%). ^1^H NMR (400 MHz, CDCl_3_) δ: 8.38 (s, 1H), 7.84 (d, *J* = 9.8 Hz, 1H), 6.90 (dd, *J* = 9.7, 0.7 Hz, 1H), 4.04 (s, 3H).

##### 4.1.6.34 Synthesis of *tert-*Butyl (4-(2-(3-Fluoro-5-methyl-6-oxo-5,6-dihydro-1,5-naphthyridin-4-yl)-2-hydroxyethyl)­cyclohexyl)­carbamate (**xxxiv**)

**xxxiii** (1.04 g, 4.34 mmol, 1 equiv) was dissolved in dry THF under argon and cooled to −78 °C. *n*-Butyllithium (1.8 mL, 4.55 mmol, 1.1 equiv) was added dropwise to the solution and the reaction was mixed for 30 min. The reaction mixture was quickly added to a solution of *tert-*butyl (4-(2-oxoethyl)­cyclohexyl)­carbamate (952 mg, 3.90 mmol, 0.9 equiv) in dry THF (10 mL), cooled to −78 °C, and the reaction mixture was stirred at −78 °C for 30 min. The solvent was evaporated and citric acid (10 mL) and was added to the crude mixture. The aqueous layer was extracted with ethyl acetate (2 × 10 mL). The combined organic layers were dried over Na_2_SO_4_, filtered, and evaporated. The residue was purified by flash column chromatography (MF = DCM:MeOH = 20:1) to give **xxxiv** (176 mg, 9.7%). ^1^H NMR (400 MHz, CDCl_3_) δ: 8.38 (d, *J* = 2.1 Hz, 1H), 7.79 (d, *J* = 9.7 Hz, 1H), 6.76 (d, *J* = 9.7 Hz, 1H), 5.43–5.29 (m, 1H), 4.43 (s, 1H), 3.73 (s, 3H), 3.04 (dd, *J* = 8.5, 3.6 Hz, 1H), 2.34–2.16 (m, 1H), 2.03–1.96 (m, 2H), 1.90 (ddd, *J* = 10.5, 5.0, 2.1 Hz, 1H), 1.81 (d, *J* = 22.2 Hz, 1H), 1.70 (ddd, *J* = 14.3, 8.8, 3.5 Hz, 1H), 1.43 (s, 10H), 1.20–0.95 (m, 4H).

##### 4.1.6.35 Synthesis of 8-(2-(4-Aminocyclohexyl)-1-hydroxyethyl)-7-fluoro-1-methyl-1,5-naphthyridin-2­(1*H*)-one (**xxxv**)

The title compound was obtained by general procedure B, using **xxxiv** (176 mg, 0.42 mmol) and used in the next step without any further purification to give **xxxv** (91 mg, 78%).

##### 4.1.6.36 Synthesis of *tert-*Butyl (*R*)-(1-((3,8-ioxo-1,2-dihydro-3*H*,8*H*-2a,5,8a-triazaacenaphthylen-2-yl)­methyl)­piperidin-4-yl)­carbamate (**xxxvi**)

The title compound was synthesized according to the procedure described in the patent WO 2009/141398 A1 to give 28 mg of **xxxvi**. ^1^H NMR (400 MHz, CDCl_3_) δ: 7.82 (s, 1H), 7.77 (d, *J* = 9.6 Hz, 1H), 6.38 (d, *J* = 9.6 Hz, 1H), 4.54 (dd, *J* = 12.5, 4.6 Hz, 1H), 4.38 (dd, *J* = 12.5, 9.3 Hz, 1H), 3.44 (s, 1H), 3.16 (dd, *J* = 13.0, 3.6 Hz, 1H), 2.95–2.84 (m, 1H), 2.73–2.59 (m, 2H), 2.36 (dtd, *J* = 37.7, 11.4, 2.7 Hz, 2H), 1.98–1.81 (m, 2H), 1.44 (s, 10H), 1.39–1.23 (m, 2H).

##### 4.1.6.37 Synthesis of (*R*)-2-((4-Aminopiperidin-1-yl)­methyl)-1,2-dihydro-3*H*,8*H*-2a,5,8a-triazaacenaphthylene-3,8-dione (**xxxvii**)

The title compound was synthesized from **xxxvi** (28 mg, 0.07 mmol) according to the procedure described in the patent WO 2009/141398 A1 to give of **xxxvii** (20 mg, 95%). The compound was used in the next step without any further purification.

##### 4.1.6.38 Synthesis of *tert-*Butyl (4-(2-Azidoethyl)­cyclohexyl)­carbamate (**xxxviii**)

The title compound was obtained by general procedure D, by combining *tert-*butyl (4-(2-hydroxyethyl)­cyclohexyl)­carbamate (1.3 g, 5.32 mmol, 1 equiv), TEA (2.3 mL, 16 mmol, 3 equiv), and mesyl chloride (494 μL, 6.38 mmol, 1.2 equiv). After a quick extraction with DCM the organic phases were dried and the crude product was added to a solution of NaN_3_ (415 mg, 6.38 mmol, 1.2 equiv). When the reaction was finished the product was purified by extraction with diethyl ether (2 × 25 mL) and dried over Na_2_SO_4_ and evaporated to give **xxxviii** in quantitative yield, that was used in the next step without any additional analytics.

##### 4.1.6.39 Synthesis of *tert-*Butyl (4-(2-(4-(Pyridin-3-yl)-1*H*-1,2,3-triazol-1-yl)­ethyl)­cyclohexyl)­carbamate (**xxxix**)

The title compound was obtained by general procedure E, by combining **xxxviii** (200 mg, 0.75 mmol, 1 equiv), 3-ethynylpyridine (81 mg, 0.78 mmol, 1.05 equiv), 0.25 M aqueous solution of CuSO_4_(aq) (0.5 mL) and 0.25 M aqueous solution of ascorbic acid (1 mL). The crude product was purified by an automated flash chromatography system using gradient elution (MF = 0.1 M TFA:CH_3_CN, 0 to 100%) (Isolera One, Biotage) to give **xxxix** (110 mg, 4%). ^1^H NMR (400 MHz, CDCl_3_) δ: 8.94 (d, *J* = 2.1 Hz, 1H), 8.51 (d, *J* = 4.7 Hz, 1H), 8.17–8.09 (m, 1H), 7.84 (s, 1H), 7.31 (dd, *J* = 7.9, 4.8 Hz, 1H), 4.50 (d, *J* = 8.2 Hz, 1H), 4.39 (t, *J* = 7.4 Hz, 2H), 3.32 (s, 1H), 1.95 (p, *J* = 5.1 Hz, 2H), 1.90–1.69 (m, 4H), 1.37 (s, 9H), 1.28–1.14 (m, 1H), 1.14–0.92 (m, 4H).

##### 4.1.6.40 Synthesis of *tert-*Butyl (4-(2-(4-(3-Acetamidophenyl)-1*H*-1,2,3-triazol-1-yl)­ethyl)­cyclohexyl)­carbamate (**xl**)

The title compound was obtained by general procedure E, by combining **xxxviii** (300 mg, 1.12 mmol, 1 equiv), *N*-(3-ethynylphenyl)­acetamide (121 mg, 1.17 mmol, 1.05 equiv), 0.25 M aqueous solution of CuSO_4_(aq) (0.75 mL) and 0.25 M aqueous solution of ascorbic acid (1.5 mL). The crude product was purified by an automated flash chromatography system using gradient elution (MF = 0.1 M TFA:CH_3_CN, 0 to 100%) (Isolera One, Biotage) to give **xl** (203 mg, 49%). ^1^H NMR (400 MHz, DMSO-*d*_6_) δ: 10.03 (s, 1H), 8.53 (s, 1H), 8.12 (t, *J* = 1.9 Hz, 1H), 7.53 (dq, *J* = 8.0, 1.3 Hz, 1H), 7.45 (dt, *J* = 7.6, 1.4 Hz, 1H), 7.34 (t, *J* = 7.9 Hz, 1H), 6.67 (d, *J* = 8.0 Hz, 1H), 4.41 (t, *J* = 7.3 Hz, 2H), 3.20–3.09 (m, 1H), 2.06 (s, 3H), 1.81–1.72 (m, 6H), 1.36 (s, 9H), 1.16–0.90 (m, 5H).

##### 4.1.6.41 Synthesis of 4-(2-(4-(Pyridin-3-yl)-1*H*-1,2,3-triazol-1-yl)­ethyl)­cyclohexan-1-amine (**xli**)

The title compound was obtained by general procedure B, using **xxxix** (110 mg, 0.30 mmol). The crude product was purified by an automated flash chromatography system using gradient elution (MF = 0.1 M TFA:CH_3_CN, 0 to 100%) (Isolera One, Biotage) to give **xli** (92 mg, 100%) and was used in the next step without any further analysis.

##### 4.1.6.42 Synthesis of *N*-(3-(1-(2-(4-Aminocyclohexyl)­ethyl)-1*H*-1,2,3-triazol-4-yl)­phenyl)­acetamide (**xlii**)

The title compound was obtained by general procedure B, using **xl** (117 mg, 0.27 mmol). The crude product was purified by an automated flash chromatography system using gradient elution (MF = 0.1 M TFA:CH_3_CN, 0 to 100%) (Isolera One, Biotage) to give **xlii** (87 mg, 100%) and was used in the next step without any further analysis.

#### 4.1.7 Final Compounds

##### 4.1.7.1 Synthesis of 4-((4-((4-Bromo-3,5-difluorobenzyl)­amino)­cyclohexyl)­methoxy)­quinolin-2­(1*H*)-one (**5**)

The title compound was obtained by general procedure C, using **xx** (164 mg, 0.60 mmol, 1.0 equiv), 4-bromo-3,5-difluorobenzaldehyde (mg, mmol, 1.2 equiv), and NaBH_3_CN (152 mg, 2.4 mmol, 4 equiv). The crude product was purified by an automated flash chromatography system using gradient elution (MF = 0.1 M TFA:CH_3_CN, 0 to 100%) (Isolera One, Biotage) to give **5** as an off-white solid (132 mg, 46%). ^1^H NMR (400 MHz, DMSO-*d*_6_) δ: 11.25 (s, 1H), 7.71 (dd, *J* = 8.0, 1.5 Hz, 1H), 7.47–7.27 (m, 3H), 7.21–7.00 (m, 2H), 5.76 (s, 1H), 4.10 (s, 2H), 3.86 (d, *J* = 6.1 Hz, 2H), 2.91 (d, *J* = 12.3 Hz, 1H), 2.13–2.00 (m, 2H), 1.94–1.67 (m, 2H), 1.36–1.03 (m, 4H). ^13^C NMR (100 MHz, DMSO-*d*_6_) δ: 163.72, 162.78, 159.45 (dd, *J* = 246.5, 4.7 Hz), 139.12, 131.47, 122.72, 121.80, 115.69, 115.09, 114.33 (d, *J* = 23.9 Hz), 97.49, 72.84, 56.69, 46.83, 36.30, 28.86, 27.39. HRMS (ESI^+^) *m*/*z* for C_23_H_24_BrF_2_N_2_O_2_ ([M + H]^+^): found 477.0985; calculated 477.0984. HPLC: *t*_R_ = 6.572 min (96% at 220 nm). *T*_m_ = 170–172 °C.

##### 4.1.7.2 Synthesis of 8-((4-((4-Bromo-3,5-difluorobenzyl)­amino)­cyclohexyl)­methoxy)­quinoline-2-carbonitrile (**6**)

The title compound was obtained by general procedure C, using **xxiii** (442 mg, 1.57 mmol, 1.0 equiv), 4-bromo-3,5-difluorobenzaldehyde (417 mg, 1.88 mmol, 1.2 equiv), and NaBH_3_CN (366 mg, 6.3 mmol, 4 equiv). The crude product was purified by an automated flash chromatography system using gradient elution (MF = 0.1 M TFA:CH_3_CN, 0 to 100%) (Isolera One, Biotage) to give **6** as a white solid (103 mg, 13%). ^1^H NMR (400 MHz, CDCl_3_) δ: 8.25 (d, *J* = 8.4 Hz, 1H), 7.71 (d, *J* = 8.4 Hz, 1H), 7.60 (t, *J* = 8.0 Hz, 1H), 7.43 (dd, *J* = 8.4, 1.1 Hz, 1H), 7.13 (dd, *J* = 7.8, 1.1 Hz, 1H), 7.00 (dd, *J* = 8.0, 1.3 Hz, 2H), 4.04 (d, *J* = 6.3 Hz, 2H), 3.83 (d, *J* = 0.7 Hz, 2H), 2.57–2.43 (m, 1H), 2.14–2.02 (m, 5H), 1.30–1.12 (m, 5H). ^13^C NMR (100 MHz, CDCl_3_) δ: 159.84 (dd, *J* = 248.6, 4.5 Hz), 155.14, 143.83, 140.64, 137.19, 132.16, 130.06, 129.97, 123.98, 119.19, 117.86, 111.31, 111.29, 111.09, 111.06, 110.36, 95.47, 74.22, 56.50, 49.92, 37.10, 32.85, 28.56. HRMS (ESI^+^) *m*/*z* for C_24_H_23_F_2_BrN_3_O ([M + H]^+^): found 486.0990; calculated 486.0987. HPLC: *t*_R_ = 7.073 min (98% at 254 nm). *T*_m_ = 164–166 °C.

##### 4.1.7.3 Synthesis of *N*-(4-Bromo-3,5-difluorobenzyl)-4-(((5-nitroquinolin-8-yl)­oxy)­methyl)­cyclohexan-1-amine (**7**)

The title compound was obtained by general procedure C, using **xxiv** (301 mg, 1.23 mmol, 1.0 equiv), 4-bromo-3,5-difluorobenzaldehyde (326 mg, 1.48 mmol, 1.2 equiv), and NaBH­(OAc)_3_ (403 mg, 1.85 mmol, 1.5 equiv). The crude product was purified by flash column chromatography (SiO_2_, DCM:MeOH = 30:1) to give **7** as a yellow solid (108 mg, 17%). ^1^H NMR (400 MHz, CDCl_3_) δ: 9.23 (dd, *J* = 8.9, 1.6 Hz, 1H), 9.05 (dd, *J* = 4.2, 1.6 Hz, 1H), 8.52 (d, *J* = 8.9 Hz, 1H), 7.68 (dd, *J* = 8.9, 4.1 Hz, 1H), 7.05 (d, *J* = 8.9 Hz, 1H), 7.02–6.96 (m, 2H), 4.17–4.11 (m, 2H), 3.82 (d, *J* = 0.7 Hz, 2H), 2.55–2.44 (m, 1H), 2.19–2.00 (m, 5H), 1.30–1.11 (m, 5H). ^13^C NMR (100 MHz, CDCl_3_) δ: ^13^C NMR (101 MHz, Chloroform-d) δ 160.51, 159.85 (dd, *J* = 248.7, 4.4 Hz), 150.17, 143.68, 139.50, 137.40, 132.48, 127.57, 124.48, 123.13, 111.29, 111.26, 111.06, 111.03, 106.13, 95.54 (t, *J* = 24.6 Hz), 74.79, 56.31, 49.89, 36.77, 35.11, 32.72, 28.62, 28.55. HRMS (ESI^+^) *m*/*z* for C_23_H_23_F_2_BrN_3_O_3_ ([M + H]^+^): found 506.0886; calculated 506.0885. HPLC: *t*_R_ = 6.842 min (91% at 254 nm). *T*_m_ = 161–163 °C.

##### 4.1.7.4 Synthesis of *N*-(4-Bromo-3,5-difluorobenzyl)-4-((imidazo­[1,2-*a*]­pyridin-8-yloxy)­methyl)­cyclohexan-1-amine (**8**)

The title compound was obtained by general procedure B, using **iii** (54 mg, 0.10 mmol). The crude product was purified by an automated flash chromatography system using gradient elution (MF = 0.1 M TFA:CH_3_CN, 0 to 100%) (Isolera One, Biotage) to give **8** as a white solid (43 mg, 98%). ^1^H NMR (400 MHz, CDCl_3_) δ: 7.71 (s, 1H), 7.65–7.47 (m, 1H), 7.03–6.95 (m, 2H), 6.95–6.89 (m, 1H), 6.43 (d, *J* = 8.1 Hz, 1H), 5.00 (p, *J* = 6.4 Hz, 2H), 3.93 (d, *J* = 6.3 Hz, 2H), 3.82 (s, 2H), 2.49 (d, *J* = 3.7 Hz, 0H), 2.06–1.94 (m, 4H), 1.93 (s, 1H), 1.15 (d, *J* = 10.0 Hz, 4H). ^13^C NMR (100 MHz, CDCl_3_) δ: 161.12 (d, *J* = 4.4 Hz), 158.65 (d, *J* = 4.4 Hz), 148.51, 140.09, 132.46, 127.55, 113.34–109.99 (m), 101.12, 95.91 (t, *J* = 24.5 Hz), 73.74, 72.10, 70.62, 56.30, 49.58, 37.00, 32.33, 28.38, 21.86 (d, *J* = 18.6 Hz). HRMS (ESI^+^) *m*/*z* for C_21_H_23_F_2_BrN_3_O ([M + H]^+^): found 450.0984; calculated 450.0987. HPLC: *t*_R_ = 6.712 min (96% at 220 nm). *T*_m_ = 168–171 °C.

##### 4.1.7.5 Synthesis of *N*-(4-Bromo-3,5-difluorobenzyl)-4-(((5,7-dimethylquinolin-8-yl)­oxy)­methyl)­cyclohexan-1-amine (**9**)

The title compound was obtained by general procedure B, using **iv** (35 mg, 0.06 mmol). The crude product was purified by an automated flash chromatography system using gradient elution (MF = 0.1 M TFA:CH_3_CN, 0 to 100%) (Isolera One, Biotage) to give **9** as a white solid (27 mg, 94%). ^1^H NMR (400 MHz, CDCl_3_) δ: 8.96 (dd, *J* = 4.2, 1.7 Hz, 1H), 8.47 (dd, *J* = 8.6, 1.7 Hz, 1H), 7.60 (s, 1H), 7.49 (dd, *J* = 8.5, 4.2 Hz, 1H), 7.01–6.92 (m, 2H), 4.19–4.11 (m, 2H), 3.80 (s, 2H), 2.46 (tt, *J* = 9.9, 4.1 Hz, 1H), 2.12 (dq, *J* = 10.9, 4.6, 3.6 Hz, 2H), 2.03 (d, *J* = 8.9 Hz, 3H), 1.47–1.34 (m, 1H), 1.24–1.16 (m, 4H). ^13^C NMR (100 MHz, CDCl_3_) δ: ^13^C NMR (101 MHz, Chloroform-d) δ 159.87 (dd, *J* = 248.7, 4.5 Hz), 151.25, 148.95, 143.60, 143.26, 132.57, 130.29, 129.72, 129.00, 127.27, 119.86, 111.37, 111.13 (d, *J* = 2.6 Hz), 95.57, 79.26, 56.58, 49.85, 38.51, 32.95, 28.67, 21.96, 18.20, 16.44. HRMS (ESI^+^) *m*/*z* for C_25_H_28_F_2_BrN_2_O ([M + H]^+^): found 489.1346; calculated 489.1348. HPLC: *t*_R_ = 6.337 min (99% at 254 nm). *T*_m_ = 90–92 °C.

##### 4.1.7.6 Synthesis of *N*-(4-Bromo-3,5-difluorobenzyl)-4-(((5-fluoroquinolin-8-yl)­oxy)­methyl)­cyclohexan-1-amine (**10**)

The title compound was obtained by general procedure B, using **v** (187 mg, 0.32 mmol). The crude product was purified by an automated flash chromatography system using gradient elution (MF = 0.1 M TFA:CH_3_CN, 0 to 100%) (Isolera One, Biotage) to give **10** as a white solid (120 mg, 78%). ^1^H NMR (400 MHz, CDCl_3_) δ: 8.99 (dd, *J* = 4.2, 1.7 Hz, 1H), 8.36 (dd, *J* = 8.5, 1.8 Hz, 1H), 7.47 (dd, *J* = 8.5, 4.2 Hz, 1H), 7.10 (t, *J* = 9.0 Hz, 1H), 6.97 (d, *J* = 7.3 Hz, 2H), 6.91 (dd, *J* = 8.6, 4.6 Hz, 1H), 3.99 (d, *J* = 6.3 Hz, 2H), 3.79 (s, 2H), 2.47 (m, *J* = 10.6, 3.8 Hz, 1H), 2.15–1.98 (m, 5H), 1.25 (m, 6H). ^13^C NMR (100 MHz, CDCl_3_) δ: 159.81 (dd, *J* = 248.6, 4.5 Hz), 152.66, 151.42 (d, *J* = 3.4 Hz), 150.13 (d, *J* = 14.5 Hz), 143.77 (t, *J* = 7.9 Hz), 140.24 (d, *J* = 3.0 Hz), 129.18 (d, *J* = 4.1 Hz), 121.54 (d, *J* = 2.6 Hz), 119.88 (d, *J* = 17.6 Hz), 111.14 (dd, *J* = 23.1, 2.6 Hz), 109.40 (d, *J* = 20.7 Hz), 107.60 (d, *J* = 8.4 Hz), 95.45 (t, *J* = 24.6 Hz), 74.35, 56.44, 49.85 (d, *J* = 2.1 Hz), 36.92, 32.81, 28.69, 23.11–20.85 (m). HRMS (ESI^+^) *m*/*z* for C_23_H_23_F_3_BrN_2_O ([M + H]^+^): found 479.0943; calculated 479.0940. HPLC: *t*_R_ = 6.422 min (>99% at 254 nm). *T*_m_ = 132–134 °C.

##### 4.1.7.7 Synthesis of *N*-(4-Bromo-3,5-difluorobenzyl)-4-(((5,7-dichloroquinolin-8-yl)­oxy)­methyl)­cyclohexan-1-amine (**11**)

The title compound was obtained by general procedure B, using **vi** (200 mg, 0.32 mmol). The crude product was purified by an automated flash chromatography system using gradient elution (MF = 0.1 M TFA:CH_3_CN, 0 to 100%) (Isolera One, Biotage) to give **11** as an orange solid (120 mg, 69%). ^1^H NMR (400 MHz, CDCl_3_) δ: 8.96 (dd, *J* = 4.2, 1.7 Hz, 1H), 8.47 (dd, *J* = 8.6, 1.7 Hz, 1H), 7.60 (s, 1H), 7.49 (dd, *J* = 8.6, 4.2 Hz, 1H), 7.02–6.90 (m, 2H), 4.13 (dd, *J* = 10.8, 6.8 Hz, 2H), 3.80 (s, 2H), 2.46 (tt, *J* = 9.9, 4.0 Hz, 1H), 2.18–2.09 (m, 2H), 2.04 (d, *J* = 5.4 Hz, 2H), 1.44–1.34 (m, 1H), 1.20 (td, *J* = 10.7, 5.8 Hz, 4H). ^13^C NMR (100 MHz, CDCl_3_) δ: 171.11, 159.80 (dd, *J* = 248.5, 4.5 Hz), 151.02, 150.69, 144.23–143.29 (m), 133.22, 127.85, 126.49, 126.25, 125.84, 121.92, 112.00–110.40 (m), 95.42 (t, *J* = 24.5 Hz), 80.19, 60.38, 56.51, 49.89 (t, *J* = 2.0 Hz), 38.47, 32.99, 28.45, 21.05, 14.21. HRMS (ESI^+^) *m*/*z* for C_23_H_22_F_2_Cl_2_N_2_O ([M + H]^+^): found 529.0259; calculated 529.0255. HPLC: *t*_R_ = 7.493 min (95% at 220 nm). *T*_m_ = 100–103 °C.

##### 4.1.7.8 Synthesis of 5-Acetyl-6-((4-((4-bromo-3,5-difluorobenzyl)­amino)­cyclohexyl)­methoxy)-1,2-dihydro-4*H*-pyrrolo­[3,2,1-*ij*]­quinolin-4-one (**12**)

The title compound was obtained by general procedure B, using **vii** (176 mg, 0.27 mmol). The crude product was purified by an automated flash chromatography system using gradient elution (MF = 0.1 M TFA:CH_3_CN, 0 to 100%) (Isolera One, Biotage) to give **12** yellowish solid (61 mg, 41%). ^1^H NMR (400 MHz, CDCl_3_) δ: 7.59 (dd, *J* = 8.1, 1.0 Hz, 1H), 7.37 (dd, *J* = 7.2, 1.1 Hz, 1H), 7.15 (dd, *J* = 8.1, 7.2 Hz, 1H), 7.01–6.90 (m, 2H), 4.45–4.32 (m, 2H), 3.86 (d, *J* = 6.0 Hz, 2H), 3.81 (d, *J* = 0.9 Hz, 2H), 3.42 (tt, *J* = 8.1, 1.1 Hz, 2H), 2.65 (s, 4H), 2.46 (dq, *J* = 10.5, 6.6, 5.8 Hz, 1H), 2.08–1.98 (m, 3H), 1.98–1.89 (m, 3H), 1.83 (s, 1H), 1.23–1.08 (m, 5H). ^13^C NMR (100 MHz, CDCl_3_) δ: 201.78, 160.05, 161.10–158.35 (m), 143.70, 142.17, 130.61, 126.45, 123.24, 120.79, 120.02, 114.37, 111.17 (dd, *J* = 23.1, 2.6 Hz), 95.52 (t, *J* = 24.6 Hz), 79.57, 56.34, 49.89, 46.80, 38.31, 32.78, 32.38, 28.13, 27.25. HRMS (ESI^+^) *m*/*z* for C_27_H_28_F_2_BrN_2_O_3_ ([M + H]^+^): found 545.1244; calculated 545.1246. HPLC: *t*_R_ = 6.762 min (97% at 254 nm). *T*_m_ = 114–116 °C.

##### 4.1.7.9 Synthesis of 8-((4-((4-Bromo-3,5-difluorobenzyl)­amino)­cyclohexyl)­methoxy)-7-fluoro-1-methyl-1,5-naphthyridin-2­(1*H*)-one (**13**)

The title compound was obtained by general procedure B, using **viii** (137 mg, 0.22 mmol). The crude product was purified by an automated flash chromatography system using gradient elution (MF = 0.1 M TFA:CH_3_CN, 0 to 100%) (Isolera One, Biotage) to give **13** as a white solid (92 mg, 82%). ^1^H NMR (400 MHz, CDCl_3_) δ: 8.37 (d, *J* = 2.3 Hz, 1H), 7.79 (d, *J* = 9.7 Hz, 1H), 6.98 (d, *J* = 7.4 Hz, 2H), 6.84 (d, *J* = 9.7 Hz, 1H), 4.04 (dd, *J* = 6.2, 2.0 Hz, 2H), 3.91 (s, 3H), 3.82 (s, 2H), 2.48 (dt, *J* = 10.6, 5.3 Hz, 1H), 2.12–2.00 (m, 2H), 2.00–1.82 (m, 4H), 1.33–1.08 (m, 5H). ^13^C NMR (100 MHz, CDCl_3_) δ: 162.54, 159.85 (dd, *J* = 248.7, 4.4 Hz), 152.57 (dd, *J* = 257.1 Hz), 143.67 (t, *J* = 7.9 Hz), 141.45 (d, *J* = 11.0 Hz), 140.51, 137.20 (d, *J* = 3.6 Hz), 135.06 (d, *J* = 24.1 Hz), 131.90 (d, *J* = 2.4 Hz), 124.24 (d, *J* = 2.9 Hz), 111.14 (dd, *J* = 23.1, 2.6 Hz), 95.55 (t, *J* = 24.6 Hz), 80.85 (d, *J* = 7.2 Hz), 56.23, 49.88 (t, *J* = 2.0 Hz), 38.10, 34.29, 32.72, 28.18. HRMS (ESI^+^) *m*/*z* for C_23_H_24_F_3_BrN_3_O_2_ ([M + H]^+^): found 510.1000; calculated 510.0999. HPLC: *t*_R_ = 6.590 min (99% at 254 nm). *T*_m_ = 124–126 °C.

##### 4.1.7.10 Synthesis of *N*-(4-Bromo-3,5-difluorobenzyl)-4-(((5-chloroquinolin-8-yl)­oxy)­methyl)­cyclohexan-1-amine (**14**)

The title compound was obtained by general procedure B, using **ix** (97 mg, 0.16 mmol). The crude product was purified by an automated flash chromatography system using gradient elution (MF = 0.1 M TFA:CH_3_CN, 0 to 100%) (Isolera One, Biotage) to give **14** as a beige solid (33 mg, 42%). ^1^H NMR (400 MHz, CDCl_3_) δ: 8.99 (dd, *J* = 4.2, 1.7 Hz, 1H), 8.52 (dd, *J* = 8.5, 1.7 Hz, 1H), 7.60–7.44 (m, 2H), 6.96 (t, *J* = 8.2 Hz, 3H), 4.01 (d, *J* = 6.4 Hz, 2H), 3.80 (s, 2H), 2.47 (tt, *J* = 10.6, 3.6 Hz, 1H), 2.20–1.88 (m, 5H), 1.57 (s, 2H), 1.34–1.01 (m, 5H). ^13^C NMR (100 MHz, CDCl_3_) δ: 159.87 (dd, *J* = 248.7, 4.4 Hz), 154.22, 149.80, 140.97, 132.92, 127.11, 126.38, 122.11 (d, *J* = 29.4 Hz), 111.19 (dd, *J* = 23.0, 2.6 Hz), 108.61, 95.58 (t, *J* = 24.6 Hz), 74.19, 56.43, 49.87, 36.83, 32.77, 28.68. HRMS (ESI^+^) *m*/*z* for C_23_H_23_F_2_BrClN_2_O ([M + H]^+^): found 495.0644; calculated 495.0645. HPLC: *t*_R_ = 6.692 min (>99% at 254 nm). *T*_m_ = 103–105 °C.

##### 4.1.7.11 Synthesis of *N*-(4-Bromo-3,5-difluorobenzyl)-4-(((5-methylquinolin-8-yl)­oxy)­methyl)­cyclohexan-1-amine (**15**)

The title compound was obtained by general procedure B, using **x** (87 mg, 0.15 mmol). The crude product was purified by an automated flash chromatography system using gradient elution (MF = 0.1 M TFA:CH_3_CN, 0 to 100%) (Isolera One, Biotage) to give **15** as a white solid (33 mg, 46%). ^1^H NMR (400 MHz, CDCl_3_) δ: 8.80 (d, *J* = 4.3 Hz, 1H), 7.54 (dd, *J* = 8.5, 1.2 Hz, 1H), 7.46 (d, *J* = 7.9 Hz, 1H), 7.24 (dd, *J* = 4.4, 1.1 Hz, 1H), 7.03 (dd, *J* = 7.8, 1.2 Hz, 1H), 7.01–6.94 (m, 2H), 4.02 (d, *J* = 6.4 Hz, 2H), 3.80 (s, 2H), 2.68 (d, *J* = 0.9 Hz, 3H), 2.10 (dt, *J* = 11.1, 2.7 Hz, 3H), 2.02 (dd, *J* = 14.9, 3.5 Hz, 2H), 1.29–1.05 (m, 5H). ^13^C NMR (100 MHz, CDCl_3_) δ: 159.85 (dd, *J* = 248.6, 4.4 Hz), 155.36, 148.99, 144.02, 143.64, 140.22, 129.51, 126.24, 122.36, 115.45, 111.19 (dd, *J* = 23.0, 2.6 Hz), 108.49, 95.54 (t, *J* = 24.5 Hz), 73.98, 56.48, 49.86 (d, *J* = 2.1 Hz), 36.84, 32.81, 28.75, 19.20. HRMS (ESI^+^) *m*/*z* for C_24_H_26_F_2_BrN_2_O ([M + H]^+^): found 475.1197; calculated 475.1191. HPLC: *t*_R_ = 6.507 min (96% at 220 nm). *T*_m_ = 120–122 °C.

##### 4.1.7.12 Synthesis of *N*-(4-Bromo-3,5-difluorobenzyl)-4-((quinolin-8-yloxy)­methyl)­cyclohexan-1-amine (**16**)

The title compound was obtained by general procedure B, using **xi** (34 mg, 0.20 mmol). The crude product was purified by an automated flash chromatography system using gradient elution (MF = 0.1 M TFA:CH_3_CN, 0 to 100%) (Isolera One, Biotage) to give **16** (34 mg, 31%). ^1^H NMR (400 MHz, CDCl_3_) δ: 8.95 (dd, *J* = 4.2, 1.7 Hz, 1H), 8.11 (dd, *J* = 8.3, 1.8 Hz, 1H), 7.50–7.29 (m, 3H), 7.04 (dd, *J* = 7.7, 1.3 Hz, 1H), 7.02–6.92 (m, 2H), 4.03 (d, *J* = 6.4 Hz, 2H), 3.80 (s, 2H), 2.48 (dd, *J* = 10.6, 3.7 Hz, 1H), 2.16–2.09 (m, 2H), 2.07–1.96 (m, 2H), 1.29–0.99 (m, 5H). ^13^C NMR (100 MHz, CDCl_3_) δ: 159.85 (dd, *J* = 248.6, 4.5 Hz), 154.97, 149.36, 143.77, 140.46, 135.86, 129.53, 126.62, 121.50, 119.45, 111.28, 111.26, 111.05, 111.03, 108.72, 95.49, 73.96, 56.47, 49.89, 36.86, 32.86, 28.75. LC-MS (ESI^+^) *m*/*z* for C_23_H_23_F_2_BrN_2_O ([M + H]^+^): found 461.0; calculated 461.1. HPLC: *t*_R_ = 4.187 min (99% at 220 nm). *T*_m_ = 124–126 °C.

##### 4.1.7.13 Synthesis of 1-(4-((4-((4-Bromo-3,5-difluorobenzyl)­amino)­cyclohexyl)­methoxy)-2-methyl-1,1-dioxido-2*H*-thieno­[2,3-*e*]­[1,2]­thiazin-3-yl)­ethan-1-one (**17**)

The title compound was obtained by general procedure B, using **xii** (200 mg, 0.35 mmol). The crude product was purified by an automated flash chromatography system using gradient elution (MF = 0.1 M TFA:CH_3_CN, 0 to 100%) (Isolera One, Biotage) to give **17** as a white solid (97 mg, 58%). ^1^H NMR (400 MHz, CDCl_3_) δ: 7.64 (d, *J* = 5.2 Hz, 1H), 7.37 (d, *J* = 5.2 Hz, 1H), 6.98 (d, *J* = 7.3 Hz, 2H), 3.90 (s, 3H), 3.81 (s, 2H), 3.11 (s, 3H), 2.45 (dt, *J* = 9.0, 3.7 Hz, 1H), 2.03 (d, *J* = 12.7 Hz, 1H), 1.97–1.86 (m, 2H), 1.86–1.73 (m, 2H), 1.21–1.08 (m, 5H). ^13^C NMR (100 MHz, CDCl_3_) δ: 162.59, 150.65, 137.42, 132.10, 122.00, 121.45, 80.04, 66.64, 56.61, 56.14, 51.61, 39.65, 37.94, 36.28, 30.45 (d, *J* = 14.6 Hz), 27.55 (d, *J* = 4.3 Hz). HRMS (ESI^+^) *m*/*z* for C_23_H_26_F_2_BrN_2_O_4_S_2_ ([M + H]^+^): found 575.0485; calculated 575.0480. HPLC: *t*_R_ = 7.045 min (95% at 220 nm). *T*_m_ = 133–135 °C.

##### 4.1.7.14 Synthesis of 8-((4-((4-Bromo-3,5-difluorobenzyl)­amino)­cyclohexyl)­methoxy)­quinolin-2-amine (**18**)

The title compound was obtained by general procedure C, using **xxi** (146 mg, 0.53 mmol, 1.0 equiv), 4-bromo-3,5-difluorobenzaldehyde (145 mg, 0.64 mmol, 1.2 equiv), and NaBH­(OAc)_3_ (170 mg, 0.8 mmol, 1.5 equiv). The crude product was purified by an automated flash chromatography system using gradient elution (MF = 0.1 M TFA:CH_3_CN, 0 to 100%) (Isolera One, Biotage) to give **18** as a beige solid (54 mg, 21%). ^1^H NMR (400 MHz, CDCl_3_) δ: 7.82 (d, *J* = 8.7 Hz, 1H), 7.24–7.06 (m, 1H), 7.01–6.89 (m, 3H), 6.73 (d, *J* = 8.8 Hz, 1H), 5.17 (s, 2H), 3.96 (d, *J* = 6.5 Hz, 2H), 3.77 (s, 1H), 2.43 (ddd, *J* = 11.1, 7.1, 3.7 Hz, 1H), 2.02 (ddd, *J* = 31.5, 11.3, 4.0 Hz, 3H), 1.92–1.81 (m, 1H), 1.21–1.00 (m, 3H). ^13^C NMR (100 MHz, CDCl_3_) δ: 159.85 (dd, *J* = 248.5, 4.3 Hz), 156.54, 152.76, 144.46–142.51 (m), 139.05, 138.11 (d, *J* = 2.4 Hz), 124.58, 122.32 (d, *J* = 1.8 Hz), 119.63, 112.55–111.62 (m), 111.17 (dd, *J* = 23.0, 2.7 Hz), 110.15 (d, *J* = 3.0 Hz), 95.47 (t, *J* = 24.6 Hz), 73.96 (d, *J* = 2.9 Hz), 62.83, 56.53, 49.89, 37.06, 32.90, 29.36, 28.71. HRMS (ESI^+^) *m*/*z* for C_23_H_25_F_2_BrN_3_O ([M + H]^+^): found 476.1143; calculated 476.1144. HPLC: *t*_R_ = 6.383 min (96% at 254 nm). *T*_m_ = 128–130 °C.

##### 4.1.7.15 Synthesis of 8-((4-((4-Bromo-3,5-difluorobenzyl)­amino)­cyclohexyl)­methoxy)­quinolin-2-ol (**19**)

The title compound was obtained by general procedure C, using **xxii** (87 mg, 0.32 mmol, 1.0 equiv), 4-bromo-3,5-difluorobenzaldehyde (73 mg, 0.32 mmol, 1.0 equiv), and NaBH­(OAc)_3_ (85 mg, 0.4 mmol, 1.3 equiv). The crude product was purified by an automated flash chromatography system using gradient elution (MF = 0.1 M TFA:CH_3_CN, 0 to 100%) (Isolera One, Biotage) to give **19** as a white solid (46 mg, 30%). ^1^H NMR (400 MHz, CDCl_3_) δ: 9.34 (s, 1H), 7.73 (d, *J* = 9.5 Hz, 1H), 7.15–7.05 (m, 2H), 7.05–6.84 (m, 2H), 6.68 (d, *J* = 9.5 Hz, 1H), 3.88 (d, *J* = 6.4 Hz, 2H), 3.80 (s, 2H), 2.45 (dq, *J* = 10.6, 6.8, 5.4 Hz, 1H), 2.07–1.72 (m, 7H), 1.12 (q, *J* = 13.9 Hz, 4H). ^13^C NMR (100 MHz, CDCl_3_) δ: 162.47, 159.87 (dd, *J* = 248.7, 4.5 Hz), 144.94, 143.51, 140.94, 128.25, 122.51, 122.17, 120.17, 119.49, 111.22 (d, *J* = 25.7 Hz), 110.88, 95.62 (t, *J* = 24.6 Hz), 73.75, 56.29, 49.82, 37.21, 32.67, 28.48. HRMS (ESI^+^) *m*/*z* for C_23_H_24_F_2_BrN_2_O_2_ ([M + H]^+^): found 477.0984; calculated 477.0984. HPLC: *t*_R_ = 6.718 min (91% at 254 nm). *T*_m_ = 127–130 °C.

##### 4.1.7.16 Synthesis of 2-((4-((4-Bromo-3,5-difluorobenzyl)­amino)­piperidin-1-yl)­methyl)-1,2-dihydro-3*H*,8*H*-2*a*,5,8*a*-triazaacenaphthylene-3,8-dione (**20**)

The title compound was obtained by general procedure C, using **xxxvii** (20 mg, 0.066 mmol, 1.0 equiv), 4-bromo-3,5-difluorobenzaldehyde (17 mg, 0.08 mmol, 1.2 equiv), and NaBH­(OAc)_3_ (21 mg, 0.1 mmol, 1.5 equiv). The crude product was purified by an automated flash chromatography system using gradient elution (MF = 0.1 M TFA:CH_3_CN, 0 to 100%) (Isolera One, Biotage) to give **20** as a yellow solid (10 mg, 30%). ^1^H NMR (400 MHz, MeOD) δ: 7.78 (d, *J* = 9.6 Hz, 1H), 7.68 (s, 1H), 7.01 (d, *J* = 7.6 Hz, 2H), 6.27 (d, *J* = 9.6 Hz, 1H), 5.08–4.96 (m, 1H), 4.41–4.14 (m, 2H), 3.65 (s, 2H), 2.95 (ddd, *J* = 17.2, 12.3, 2.7 Hz, 2H), 2.74 (dd, *J* = 13.1, 8.3 Hz, 1H), 2.62–2.55 (m, 1H), 2.37–2.27 (m, 1H), 2.13 (dtd, *J* = 29.2, 11.7, 2.6 Hz, 2H), 1.84–1.67 (m, 2H), 1.33–1.14 (m, 2H). ^13^C NMR (100 MHz, CDCl_3_) δ: 161.02, 160.61, 158.56, 154.50, 143.22, 142.53, 140.04, 139.15, 115.06, 111.47, 94.91, 57.62, 56.48, 53.83, 53.60, 52.45, 31.70. HRMS (ESI^+^) *m*/*z* for C_22_H_23_F_2_BrN_5_O_2_ ([M + H]^+^): found 506.1001; calculated 506.0998. HPLC: *t*_R_ = 0.793 min (>99% at 280 nm). *T*_m_ = 135–137 °C.

##### 4.1.7.17 Synthesis of 2-(2-(4-((4-Bromo-3,5-difluorobenzyl)­amino)­cyclohexyl)­ethyl)-1*H*-benzo­[*de*]­isoquinoline-1,3­(2*H*)-dione (**21**)

The title compound was obtained by general procedure C, using **xxix** (419 mg, 1.36 mmol, 1.0 equiv), 4-bromo-3,5-difluorobenzaldehyde (363 mg, 1.6 mmol, 1.2 equiv), and NaBH­(OAc)_3_ (435 mg, 2 mmol, 1.5 equiv). The crude product was purified by an automated flash chromatography system using gradient elution (MF = 0.1 M TFA:CH_3_CN, 0 to 100%) (Isolera One, Biotage) to give **21** as a white solid (306 mg, 44%). ^1^H NMR (400 MHz, CDCl_3_) δ: 8.61 (dd, *J* = 7.3, 1.2 Hz, 2H), 8.22 (dd, *J* = 8.4, 1.2 Hz, 2H), 7.76 (dd, *J* = 8.3, 7.3 Hz, 2H), 6.95 (d, *J* = 7.6 Hz, 2H), 4.09 (d, *J* = 7.2 Hz, 2H), 3.77 (s, 2H), 2.44 (tt, *J* = 10.8, 3.6 Hz, 1H), 2.00–1.76 (m, 5H), 1.29–0.98 (m, 5H). ^13^C NMR (100 MHz, CDCl_3_) δ: 164.56, 133.94, 131.61, 131.36, 128.23, 126.98, 122.65, 111.16 (d, *J* = 24.1 Hz), 56.42, 45.67, 36.53, 33.03, 29.59. HRMS (ESI^+^) *m*/*z* for C_27_H_26_BrF_2_N_2_O_2_ ([M + H]^+^): found 527.1141; calculated 527.1140. HPLC: *t*_R_ = 7.145 min (99% at 220 nm). *T*_m_ = 164–166 °C.

##### 4.1.7.18 Synthesis of 2-((4-((4-Bromo-3,5-difluorobenzyl)­amino)­cyclohexyl)­methyl)-1*H*-benzo­[*de*]­isoquinoline-1,3­(2*H*)-dione (**22**)

The title compound was obtained by general procedure C, using **xxx** (270 mg, 0.84 mmol, 1.0 equiv), 4-bromo-3,5-difluorobenzaldehyde (230 mg, 0.92 mmol, 1.2 equiv), and NaBH­(OAc)_3_ (267 mg, 1.26 mmol, 1.5 equiv). The crude product was purified by an automated flash chromatography system using gradient elution (MF = 0.1 M TFA:CH_3_CN, 0 to 100%) (Isolera One, Biotage) to give **22** as a white solid (196 mg, 38%). ^1^H NMR (400 MHz, CDCl_3_) δ: 8.59 (d, *J* = 1.2 Hz, 2H), 8.32–8.07 (m, 2H), 7.76 (dd, *J* = 8.2, 7.3 Hz, 2H), 7.06–6.86 (m, 2H), 4.25–4.15 (m, 2H), 2.42 (d, *J* = 3.6 Hz, 1H), 2.05–1.86 (m, 4H), 1.69–1.59 (m, 2H), 1.55–1.31 (m, 4H), 1.09 (dt, *J* = 18.6, 12.2 Hz, 4H). ^13^C NMR (100 MHz, CDCl_3_) δ: 164.16, 133.89, 131.18, 126.95, 56.55, 35.70, 35.02, 33.44, 31.68. HRMS (ESI^+^) *m*/*z* for C_26_H_24_F_2_BrN_2_O_2_ ([M + H]^+^): found 513.0982; calculated 513.0984. HPLC: *t*_R_ = 7.270 min (98% at 220 nm). *T*_m_ = 135–138 °C.

##### 4.1.7.19 Synthesis of 2-(2-(4-((4-Bromo-3,5-difluorobenzyl)­amino)­piperidin-1-yl)­ethyl)-1*H*-benzo­[*de*]­isoquinoline-1,3­(2*H*)-dione (**23**)

The title compound was obtained by general procedure C, using **xxxi** (224 mg, 0.70 mmol, 1.0 equiv), 4-bromo-3,5-difluorobenzaldehyde (173 mg, 0.77 mmol, 1.1 equiv), and NaBH­(OAc)_3_ (223 mg, 1.05 mmol, 1.5 equiv). The crude product was purified by an automated flash chromatography system using gradient elution (MF = 0.1 M TFA:CH_3_CN, 0 to 100%) (Isolera One, Biotage) to give **23** as a white solid (151 mg, 40.9%). ^1^H NMR (400 MHz, CDCl_3_) δ: 8.60 (dd, *J* = 7.3, 1.1 Hz, 2H), 8.22 (dd, *J* = 8.3, 1.1 Hz, 2H), 7.76 (dd, *J* = 8.2, 7.3 Hz, 2H), 7.05–6.92 (m, 2H), 4.44–4.26 (m, 2H), 3.78 (s, 2H), 3.05 (d, *J* = 11.9 Hz, 2H), 2.76–2.66 (m, 2H), 2.46 (td, *J* = 10.3, 5.1 Hz, 1H), 2.15 (t, *J* = 11.3 Hz, 2H), 1.87 (d, *J* = 12.7 Hz, 2H), 1.45–1.32 (m, 2H). ^13^C NMR (100 MHz, CDCl_3_) δ: 164.20, 159.87 (dd, *J* = 248.6, 4.4 Hz), 143.73, 133.93, 131.62, 131.21, 128.22, 126.95, 122.71, 111.13 (dd, *J* = 23.1, 2.6 Hz), 55.65, 54.23, 52.57, 49.65, 37.75, 32.80. HRMS (ESI^+^) *m*/*z* for C_26_H_25_F_3_BrN_3_O_2_ ([M + H]^+^): found 528.1093; calculated 528.1093. HPLC: *t*_R_ = 4.437 min (>99% at 220 nm). *T*_m_ = 185–187 °C.

##### 4.1.7.20 Synthesis of 8-(2-(4-((4-Bromo-3,5-difluorobenzyl)­amino)­cyclohexyl)-1-hydroxyethyl)-7-fluoro-1-methyl-1,5-naphthyridin-2­(1*H*)-one (**24**)

The title compound was obtained by general procedure C, using **xxxv** (40 mg, 0.125 mmol, 1.0 equiv), 4-bromo-3,5-difluorobenzaldehyde (30 mg, 0.15 mmol, 1.2 equiv), and NaBH­(OAc)_3_ (31 mg, 0.19 mmol, 1.5 equiv). The crude product was purified by an automated flash chromatography system using gradient elution (MF = 0.1 M TFA:CH_3_CN, 0 to 100%) (Isolera One, Biotage) to give **24** as a white solid (53 mg, 81%). ^1^H NMR (400 MHz, CDCl_3_) δ: 8.38 (d, *J* = 2.1 Hz, 1H), 7.80 (d, *J* = 9.7 Hz, 1H), 7.02–6.90 (m, 2H), 6.79 (d, *J* = 9.7 Hz, 1H), 5.37 (ddd, *J* = 10.1, 3.5, 1.4 Hz, 1H), 3.77 (s, 2H), 3.73 (s, 3H), 2.48–2.33 (m, 1H), 2.33–2.18 (m, 1H), 2.08–1.86 (m, 3H), 1.79 (dt, *J* = 12.8, 3.0 Hz, 1H), 1.74–1.63 (m, 1H), 1.60–1.49 (m, 0H), 1.19–0.88 (m, 4H). ^13^C NMR (100 MHz, CDCl_3_) δ: 163.89, 161.11, 143.54, 140.89, 137.37, 136.86, 134.48 (d, *J* = 28.2 Hz), 126.75 (d, *J* = 11.0 Hz), 123.84, 111.76–110.46 (m), 67.01, 56.17, 49.84, 43.61, 39.11, 34.39, 34.10–31.84 (m), 30.78. HRMS (ESI^+^) *m*/*z* for C_24_H_26_F_3_BrN_3_O_2_ ([M + H]^+^): found 524.1157; calculated 524.1155. HPLC: *t*_R_ = 4.543 min (98% at 220 nm). *T*_m_ = 131–133 °C.

##### 4.1.7.21 Synthesis of 8-((4-(((3,4-Dihydro-2*H*-pyrano­[2,3-*c*]­pyridin-6-yl)­methyl)­amino)­cyclohexyl)­methoxy)-7-fluoro-1-methyl-1,5-naphthyridin-2­(1*H*)-one (**25**)

The title compound was obtained by general procedure C, using **xxv** (120 mg, 0.39 mmol, 1.0 equiv), 3,4-dihydro-2H-pyrano­[2,3-*c*]­pyridine-6-carbaldehyde (90 mg, 0.39 mmol, 1 equiv), and NaBH_3_CN (100 mg, 1.6 mmol, 4 equiv). The crude product was purified by an automated flash chromatography system using gradient elution (MF = 0.1 M TFA:CH_3_CN, 0 to 100%) (Isolera One, Biotage) to give **25** as a white solid (52 mg, 29%). ^1^H NMR (400 MHz, CDCl_3_) δ: 8.37 (d, *J* = 2.3 Hz, 1H), 8.09 (s, 1H), 7.79 (d, *J* = 9.7 Hz, 1H), 6.98 (s, 1H), 6.85 (d, *J* = 9.7 Hz, 1H), 4.26–4.16 (m, 2H), 4.03 (dd, *J* = 6.2, 2.0 Hz, 2H), 3.92 (s, 3H), 3.82 (s, 2H), 2.76 (t, *J* = 6.5 Hz, 2H), 2.51 (tt, *J* = 10.5, 3.8 Hz, 1H), 2.12–1.79 (m, 7H), 1.33–1.11 (m, 5H). ^13^C NMR (100 MHz, CDCl_3_) δ: 162.57, 152.59 (d, *J* = 257.1 Hz), 150.81 (d, *J* = 2.0 Hz), 141.53 (d, *J* = 11.0 Hz), 140.49, 138.64, 137.20 (d, *J* = 3.6 Hz), 135.07 (d, *J* = 24.1 Hz), 131.93 (d, *J* = 2.5 Hz), 130.95, 124.24 (d, *J* = 2.8 Hz), 122.69, 81.02 (d, *J* = 7.1 Hz), 66.54, 56.55, 51.93, 38.18, 34.32, 32.59, 28.21, 24.23, 21.70. HRMS (ESI^+^) *m*/*z* for C_25_H_30_FN_4_O_3_ ([M + H]^+^): found 453.2293; calculated 453.2297. HPLC: *t*_R_ = 4.340 min (95% at 220 nm). *T*_m_ = 166–170 °C.

##### 4.1.7.22 Synthesis of *N*-(4-Bromo-3,5-difluorobenzyl)-4-(2-(4-(pyridin-3-yl)-1*H*-1,2,3-triazol-1-yl)­ethyl)­cyclohexan-1-amine (**26**)

The title compound was obtained by general procedure C, using **li** (81 mg, 0.30 mmol, 1.0 equiv), 4-bromo-3,5-difluorobenzaldehyde (79 mg, 0.36 mmol, 1.2 equiv), and NaBH_3_CN (28 mg, 0.44 mmol, 1.5 equiv). The crude product was purified by an automated flash chromatography system using gradient elution (MF = 0.1 M TFA:CH_3_CN, 0 to 100%) (Isolera One, Biotage) to give **26** as a white solid (16 mg, 11%). ^1^H NMR (400 MHz, CDCl_3_) δ: 8.98 (dd, *J* = 2.2, 0.9 Hz, 1H), 8.57 (dd, *J* = 4.8, 1.7 Hz, 1H), 8.21 (dt, *J* = 7.9, 2.0 Hz, 1H), 7.82 (s, 1H), 7.37 (ddd, *J* = 8.0, 4.8, 0.9 Hz, 1H), 6.99–6.92 (m, 2H), 4.50–4.40 (m, 2H), 3.78 (s, 2H), 2.45–2.36 (m, 1H), 2.00–1.92 (m, 2H), 1.92–1.80 (m, 4H), 1.37–1.26 (m, 1H), 1.15–0.97 (m, 5H). ^13^C NMR (100 MHz, CDCl_3_) δ: 159.86 (dd, *J* = 248.6, 4.5 Hz), 149.24, 147.01, 144.76, 143.76 (t, *J* = 7.9 Hz), 132.98, 126.81, 123.79, 119.58, 111.11 (dd, *J* = 23.1, 2.5 Hz), 95.53 (t, *J* = 24.6 Hz), 56.19, 49.86, 48.54, 37.28, 34.67, 33.16, 31.42. HRMS (ESI^+^) *m*/*z* for C_21_H_24_N_6_BrF_2_ ([M + H]^+^): found 476.126; calculated 476.126. HPLC: *t*_R_ = 6.353 min (>99% at 220 nm). *T*_m_ = 119–121 °C.

##### 4.1.7.23 Synthesis of *N*-(3-(1-(2-(4-((4-Bromo-3,5-difluorobenzyl)­amino)­cyclohexyl)­ethyl)-1*H*-1,2,3-triazol-4-yl)­phenyl)­acetamide (**27**)

The title compound was obtained by general procedure C, using **lii** (87 mg, 0.27 mmol, 1.0 equiv), 4-bromo-3,5-difluorobenzaldehyde (73 mg, 0.32 mmol, 1.2 equiv), and NaBH_3_CN (26 mg, 0.41 mmol, 1.5 equiv). The crude product was purified by an automated flash chromatography system using gradient elution (MF = 0.1 M TFA:CH_3_CN, 0 to 100%) (Isolera One, Biotage) to give **27** as an off-white solid (60 mg, 41%). ^1^H NMR (400 MHz, CDCl_3_) δ: 8.13 (s, 1H), 7.99 (t, *J* = 1.9 Hz, 1H), 7.71 (s, 1H), 7.58–7.47 (m, 2H), 7.31 (t, *J* = 7.9 Hz, 1H), 6.99–6.89 (m, 2H), 4.36 (t, *J* = 7.5 Hz, 2H), 3.75 (s, 2H), 2.43–2.32 (m, 1H), 2.15 (s, 3H), 1.97–1.87 (m, 2H), 1.81–1.75 (m, 4H), 1.59 (br s, 1H), 1.28–1.18 (m, 1H), 1.08–0.94 (m, 4H). ^13^C NMR (100 MHz, CDCl_3_) δ: 169.02, 159.90 (dd, *J* = 248.6, 4.4 Hz), 147.50, 143.90 (t, *J* = 7.9 Hz), 138.85, 131.34, 129.58, 121.42, 119.86, 119.65, 117.09, 111.21 (dd, *J* = 22.9, 2.3 Hz), 95.54 (t, *J* = 24.5 Hz), 56.28, 49.91, 48.50, 37.28, 34.70, 33.21, 31.47, 24.66. HRMS (ESI^+^) *m*/*z* for C_25_H_29_N_5_BrOF_2_ ([M + H]^+^): found 532.152; calculated 532.152. HPLC: *t*_R_ = 6.545 min (>99% at 220 nm). *T*_m_ = 135–137 °C.

##### 4.1.7.24 Synthesis of 3-(1-(2-(4-((4-Bromo-3,5-difluorobenzyl)­amino)­cyclohexyl)­ethyl)-1*H*-1,2,3-triazol-4-yl)­aniline (**28**)

The title compound was obtained by dissolving **27** (20 mg, 0.04 mmol, 1 equiv) in 1,4-dioxane (5 mL) and adding concentrated HCl (37%, 5 mL). The reaction mixture was stirred at 80 °C for 24 h. Then the solvent was evaporated and 50% NaOH was added so that the pH reached 10. The aqueous solution was extracted with EtOAc (3 × 20 mL). The organic phases were combined, dried over Na_2_SO_4_ and evaporated to give **28** as a light orange solid (7 mg, 38%). ^1^H NMR (400 MHz, CDCl_3_) δ: 7.68 (s, 1H), 7.39–7.29 (m, 1H), 7.19 (t, *J* = 7.7 Hz, 1H), 7.11 (dt, *J* = 7.7, 1.3 Hz, 1H), 7.00–6.93 (m, 2H), 6.65 (ddd, *J* = 7.9, 2.4, 1.1 Hz, 1H), 4.45–4.37 (m, 2H), 3.78 (s, 2H), 3.75 (br s, 2H), 2.47–2.38 (m, 1H), 1.99–1.92 (m, 2H), 1.87–1.79 (m, 4H), 1.35–1.21 (m, 2H), 1.15–0.95 (m, 4H). ^13^C NMR (100 MHz, CDCl_3_) δ: 159.99 (dd, *J* = 248.8, 4.5 Hz), 148.04, 147.05, 131.67, 130.55, 129.89, 119.44, 116.10, 115.03, 112.32, 111.39 (dd, *J* = 23.2, 2.6 Hz), 95.83 (t, *J* = 24.6 Hz), 56.38, 49.84, 48.46, 37.37, 34.73, 33.06, 31.52. HRMS (ESI^+^) *m*/*z* for C_23_H_27_N_5_BrF_2_ ([M + H]^+^): found 490.141; calculated 490.141. HPLC: *t*_R_ = 6.423 min (88% at 220 nm). *T*_m_ = 108–110 °C.

### 4.2 Antibacterial Testing

#### 4.2.1 Minimum Inhibitory Concentration (MIC) Determination

MICs were determined by the broth microdilution method in 96-well plate format according to Clinical and Laboratory Standards Institute guidelines and European Committee on Antimicrobial Susceptibility Testing (EUCAST) recommendations. The bacterial suspension of the specific bacterial strain equivalent to the 0.5 McFarland turbidity standard was diluted with cation-adapted Mueller-Hinton broth with TES (Thermo Fisher Scientific) to obtain a final inoculum of 10^5^ CFU/mL. The compounds dissolved in DMSO and the inoculum were mixed together and incubated at 35 °C for 20 h. After incubation, MIC values were determined by visual inspection, as the lowest dilution of compounds showing no turbidity. MICs were determined against various bacterial strains. Tetracycline was used as a positive control on assay plates and showed MIC values on *S. aureus* 0.25 μg/mL, *E. coli* 1 μg/mL, *E. coli* D22 1 μg/mL, *E. coli* N43 0.25 μg/mL and *P. aeruginosa* 16 μg/mL. Ciprofloxacin was used as a positive control on MRSAQA-12.1 8 μg/mL, MRSAQA-11.7 8 μg/mL, MRSAQA-11.2 ≤ 0.125 μg/mL, *K. pneumoniae* 0.125 μg/mL and *A. baumannii* 0.008 μg/mL.

#### 4.2.2 Time-Kill Assay

*A. baumannii* (ATCC19606), *E. coli* (ATCC25922), and MRSA (ATCC 43300) were grown in Mueller-Hinton broth (MH) overnight at 37 °C. The overnight culture was diluted to approximately 10^6^ CFU/mL and then treated with **13** or gepotidacin at MIC, and 8 × MIC for 24 h. The untreated culture served as the control. Aliquots of the cultures were serially diluted in PBS and plated on Luria–Bertani (LB) agar at the 0, 2, 4, 6, 8, and 24 h time points. The CFU were calculated for each time point. Bactericidal concentrations were defined as those achieving a 99.9% (3log_10_) reduction in CFU/mL compared to the starting inoculum. The experiment was performed in triplicate.

#### 4.2.3 Postantibiotic Effect (PAE)

PAE was determined by viable counting method according to McDonald et al.^55^ *A. baumannii* (ATCC19606), *E. coli* (ATCC25922), and MRSA (ATCC 43300) were grown in MH broth overnight at 37 °C. The overnight culture was diluted to approximately 10^6^ CFU/mL in MH broth. Cultures were treated with 1 × MIC **13**, 1 × MIC gepotidacin, 8 × MIC **13**, and 8 × MIC gepotidacin. All treatments were performed in triplicate. Three aliquots of the culture were treated with DMSO and served as negative controls. The cultures were incubated for 60 min and the bacteria harvested by centrifugation (5000 g, 5 min). Bacteria were washed three times with MH broth and finally, the cell pellets were resuspended in fresh MH broth using volumes equivalent to the original culture volumes. The new cultures were then incubated at 37 °C. Samples were removed for viable counting before washing, immediately after washing (time 0) and at hourly intervals for 8 h. Samples were serially diluted in PBS, plated on LB agar and the CFUs were calculated after incubation at 37 °C for 20 h. The duration (*D*) of the PAE was calculated according to *D* = *T* – *C* where *T* and *C* represent the time (h) required for the treated and untreated culture, respectively, to increase CFU by 10-fold (by 1log_10_ CFU/mL).

#### 4.2.4 Resistance Development

The potential of *A. baumannii* (ATCC19606), *E. coli* (ATCC25922), and MRSA (ATCC 43300) to develop resistance to **13** and gepotidacin was evaluated following a previously described procedure with some modifications.^56^ Initially, MICs were determined as described above. Each day, bacteria from the wells exposed to subinhibitory concentrations (1/2 × MIC) of either **13** or gepotidacin were harvested, their density was adjusted to 10^5^ CFU/mL and the inoculum was subjected to the next passage MIC testing. The procedure was repeated over a period of 14 days (14 passages). All concentrations were tested in triplicates.

### 4.3 Enzyme-Based Assays

#### 4.3.1 DNA Gyrase and TopoIV High-Throughput Assays

A Gyrase Supercoiling High Throughput Plate Assay Kit and a TopoIV Relaxation High Throughput Plate Assay Kit (Inspiralis, Norwich, UK) were used to determine the residual activity values of the compounds for *S. aureus* and *E. coli* topoisomerases. Assays were performed on black streptavidin-coated 96-well microtiter plates (Thermo Scientific Pierce, Norwich, UK). The wells were first rehydrated with the supplied wash buffer (20 mM Tris-HCl (pH 7.6), 137 mM NaCl, 0.005% [w/v] BSA, and 0.05% [v/v] Tween-20). The biotinylated oligonucleotide was diluted with the wash buffer and then immobilized onto each well; the excess was removed with the wash buffer. An appropriate amount of enzyme (predetermined by an enzyme titration), together with 0.75 μg of relaxed (in the Gyrase assay) or supercoiled pNO1 plasmid (in the TopoIV assay) as substrate, was incubated in the presence of 3 μL inhibitor solution in 10% DMSO and 0.008% Tween-20 at 37 °C for 30 min in a final reaction volume of 30 μL in assay buffer (*S. aureus* DNA gyrase: 40 mM HEPES-KOH (pH 7.6), 10 mM magnesium acetate, 10 mM DTT, 2 mM ATP, 500 mM potassium glutamate, and 0.05 mg/mL albumin; *E. coli* DNA gyrase: 35 mM Tris-HCl (pH 7.5), 24 mM KCl, 4 mM MgCl_2_, 2 mM DTT, 1.8 mM spermidine, 1 mM ATP, 6.5% (w/v) glycerol, and 0.1 mg/mL albumin; *S. aureus* TopoIV: 50 mM Tris-HCl (pH 7.5), 5 mM MgCl_2_, 5 mM DTT, 1.5 mM ATP, 350 mM potassium glutamate, and 0.05 mg/mL albumin; *E. coli* TopoIV: 40 mM HEPES-KOH (pH 7.6), 100 mM potassium glutamate, 10 mM magnesium acetate, 10 mM DTT, 1 mM ATP, and 50 μg/mL albumin). The reaction was stopped by adding the 100 μL of TF buffer (50 mM NaOAc (pH 4.7), 50 mM NaCl, and 50 mM MgCl_2_) to allow triplex formation (biotin oligonucleotide plasmid) for another 30 min. Finally, the unbound plasmid was washed off with the TF buffer and the Promega Diamond dye was added to the T10 buffer solution (10 mM Tris-HCl (pH 8.0), 1 mM EDTA). After 15 min, the solution was mixed and fluorescence was read using a BioTek Synergy H4 microplate reader (excitation, 495 nm; emission, 537 nm). Screening was performed at an inhibitor concentration of 1 μM. Raw data were converted to mean values ± SD percent of residual enzyme activity determined by two independent measurements. The compounds exhibiting the highest on-target activity and good MIC values were used in further IC_50_ determination.

#### 4.3.2 DNA Gyrase and TopoIV Gel-Based Assay

IC_50_ values of the selected compounds were determined using a Gyrase Supercoiling Assay Kit and a Topoisomerase IV Relaxation Assay Kit (Inspiralis, Norwich, UK). An appropriate amount of enzyme (predetermined by an enzyme titration), together with 0.5 μg of relaxed (in the Gyrase assay) or supercoiled pNO1 plasmid (in the TopoIV assay) as substrate, was incubated in the presence of 0.3 μL inhibitor solution in DMSO at 37 °C for 30 min in a final reaction volume of 30 μL in assay buffer (*S. aureus* DNA gyrase: 40 mM HEPES-KOH (pH 7.6), 10 mM magnesium acetate, 10 mM DTT, 2 mM ATP, 500 mM potassium glutamate and 0.05 mg/mL albumin; *E. coli* DNA gyrase: 35 mM Tris-HCl (pH 7.5), 24 mM KCl, 4 mM MgCl_2_, 2 mM DTT, 1.8 mM spermidine, 1 mM ATP, 6.5% (w/v) glycerol, and 0.1 mg/mL albumin; *S. aureus* TopoIV: 50 mM Tris-HCl (pH 7.5), 5 mM MgCl_2_, 5 mM DTT, 1.5 mM ATP, 350 mM potassium glutamate, and 0.05 mg/mL albumin; *E. coli* TopoIV: 40 mM HEPES-KOH (pH 7.6), 100 mM potassium glutamate, 10 mM magnesium acetate, 10 mM DTT, 1 mM ATP, and 50 μg/mL albumin). The reaction was stopped by the addition of 6 μL of 10X GSTEB (40% (w/v) Glycerol, 100 mM Tris-HCl pH 8, 10 mM EDTA, 0.5 mg/mL Bromophenol Blue) in 24 μL of water and 30 μL of chloroform/isoamyl alcohol (v:v, 24:1). The mixture was vortex, centrifuged and then 20 μL of the upper blue phase was loaded onto a 1% (w/v) agarose gel. The gel was run at ∼90 V for approximately 1.5 h, stained for 15 min with 1 μg/mL ethidium bromide in water, destained for 10 min in water and visualized with a gel documentation system (G-box, Syngene). The inhibitor concentration at which the residual activity of the enzyme is 50% (IC_50_) was calculated by a nonlinear regression-based fit of the inhibition curves using the log [inhibitor] versus slope of the reaction variables (four-parameter symmetric equation) in GraphPad Prism 10 software (GraphPad Software, CA, USA). The IC_50_ value represents the average of two independent measurements. Gepotidacin was used as a positive control and showed an IC_50_ value for DNA gyrase of 0.374 μM and 0.244 μM for *S. aureus* and *E. coli*, and TopoIV of 8.30 μM and 0.049 μM for *S. aureus* and *E. coli*.

#### 4.3.3 Human Topoisomerase IIα Selectivity Assay

A Human Topoisomerase II Alpha Relaxation High Throughput Plate Assay Kit, purchased from Inspiralis (Norwich, UK), was used to evaluate the compounds’ selectivity for the bacterial enzymes over the orthologous human topoisomerase IIα enzyme. The assay was performed on black streptavidin-coated 96-well microtiter plates (Thermo Scientific Pierce). First, wells were rehydrated with the provided wash buffer (20 mM Tris-HCl (pH 7.6), 137 mM NaCl, 0.005% (w/v) BSA, 0.05% (v/v) Tween 20). Biotinylated oligonucleotide in wash buffer was immobilized in the wells and the excess oligonucleotide was washed off with wash buffer. 1.5 U of Human topoisomerase IIα enzyme was incubated together with 0.75 μg of supercoiled pNO1 plasmid as substrate in the presence of 3 μL inhibitor solution in 10% DMSO and 0.008% Tween 20 and 1 μL 30 mM ATP, at 37 °C for 30 min in a final reaction volume of 30 μL in buffer (50 mM Tris-HCl (pH 7.5), 125 mM NaCl, 10 mM MgCl_2_, 5 mM DTT, and 100 μg/mL albumin). The reactions were stopped by adding the TF buffer (50 mM NaOAc (pH 4.7), 50 mM NaCl, and 50 mM MgCl_2_) to allow triplex formation (biotin-oligonucleotide plasmid) for additional 30 min. Then, the unbound plasmid was washed off with TF buffer and the solution of Promega Diamond dye in T10 buffer (10 mM Tris-HCl (pH 8.0), 1 mM EDTA) was added. Ten minutes later, the solution was mixed and fluorescence was read using a BioTek Synergy H4 microplate reader (excitation, 495 nm; emission, 537 nm). Screening was performed at an inhibitor concentration of 10 μM. Raw data were converted to mean values ± SD percent of residual enzyme activity determined by two independent measurements.

### 4.4 hERG Inhibition

The hERG screening was performed at TCG Lifesciences Pvt. Ltd. in Kolkata, West Bengal, India, using a Fluorescence Polarization-based hERG binding assay (Invitrogen kit). The same kit was used in-house to test additional compounds. The assay was performed according to the instructions in a 384-well format, on black untreated low-volume polystyrene plates. A solution of the compounds in DMSO was diluted in the assay buffer in order to obtain an intermediate solution in 4% DMSO. To this solution, 10 μL of the hERG membrane and 5 μL of tracer dye was added, so that the final concentrations of the compounds were obtained in 1% DMSO. The plates were covered and incubated at room temperature for 3 h, then the fluorescence polarization was measured on Spark multimode microplate reader, Tecan Group Ltd., Switzerland. The compounds were tested at 8 concentrations (30–0.013 μmol) in triplicate, and the data were fitted using GraphPad Prism using the nonlinear regression fit, in order to determine the IC_50_.

### 4.5 Metabolic Activity Assessment

HepG2 (ATCC, VA, USA) cell lines were cultured in Dulbecco’s Modified Eagle Medium (DMEM; Sigma-Aldrich, MO, USA) supplemented with 10% fetal bovine serum (Gibco, NY, USA), 2 mM l-glutamine, 100 U/mL penicillin, and 100 μg/mL streptomycin (all from Sigma-Aldrich, MO, USA) in a humidified chamber at 37 °C and 5% CO_2_. Cells were seeded into 96-well plates at a density of 8,000 cells per well and allowed to attach overnight. After 24 h, cells were treated with compound of interest (concentration range 1.25–100 μM) or corresponding vehicle as control. The metabolic activity was assessed after 72 h treatment using the CellTiter 96 Aqueous One Solution Cell Proliferation Assay (Promega, WI, USA). The absorbance was measured at 492 nm on an automated microplate reader Spark Multimode Microplate Reader (Tecan Group Ltd., Switzerland). Raw data were converted to mean values ± SD percent of residual cell activity determined by two independent measurements.

### 4.6 Antibiofilm Assays

#### 4.6.1 Biofilm Formation Inhibition Assay

The antibiofilm activity of the compounds was evaluated on *A. baumannii* (ATCC19606), *E. coli* (ATCC25922), and MRSA (ATCC 43300) biofilms in a 96-well plate format. *A. baumannii* and MRSA were grown in brain-heart infusion (BHI) medium, 1% glucose was added to MRSA. *E. coli* was grown in LB medium without NaCl. The effect on biofilm formation was measured after 24 h of incubation with increasing concentrations of the compounds that were dissolved in DMSO. Treatment with DMSO was used as control. After 24 h, planktonic bacteria were removed from the wells and remaining biofilms washed three times with water. 150 μL of 0.01% crystal violet was used to stain the biofilms for 20 min. After washing three times with water and complete drying, 150 μL of 30% acetic acid was added to each well and the biofilm biomass was quantified using a spectrophotometer at 595 nm.

#### 4.6.2 Mature Biofilm Eradication Assay

Overnight cultures of *A. baumannii* (ATCC19606), *E. coli* (ATCC25922), and MRSA (ATCC 43300) were diluted to 5 × 10^7^ cells/mL in BHI, LB without NaCl, and BHI with 1% glucose, respectively. 100 μL was added to the wells in 96-well microtiter plates and incubated for 24 h at 37 °C. After removal of planktonic bacteria from the wells and two washing steps with PBS, adherent cells were exposed to 150 μL of different concentrations of the compounds in six replicates for additional 24 h and the biofilm biomass was quantified using crystal violet as described above, for the biofilm formation inhibition assay. The wells without treatment served as a control.

#### 4.6.3 Resazurin Assay

In order to evaluate the viability of bacterial cells in the mature biofilm after the treatment with the compounds, resazurin assay was performed. Mature biofilms of *A. baumannii* (ATCC19606), *E. coli* (ATCC25922), and MRSA (ATCC 43300) were prepared in 96-well microtiter plates and treated with compounds as described above. Then, a 0.02% resazurin solution was prepared in 1-part Milli-Q water, 3-part LB medium. 200 μL of resazurin was added to the wells and incubated for 1–2 h at 37 °C. Fluorescence was measured (ex. 560 nm, em. 590 nm). The wells without treatment served as a control.

### 4.7 Zebrafish Embryo Acute Toxicity (FET) Test

No formal ethics review was required for the experiments involving zebrafish embryos. Experiments involving zebrafish embryos were performed in accordance with the general rules stated in the OECD Guidelines for the Testing of Chemicals (OECD, 2013, Test No. 236) and in compliance with the European Directive 2010/63/EU and the ethical guidelines for the care and use of laboratory animals of the Institute of Molecular Genetics and Genetic Engineering, University of Belgrade. Wild-type (AB) zebrafish were raised to adult stage in a temperature- and light-controlled facility at 28 °C and a standard 14:10 h light–dark photoperiod, and were fed with commercial dry food twice a day and *Artemia* *nauplii* daily. Embryos produced by pairwise mating were collected, washed from debris and distributed into 24-well plates containing 1 mL of E3 medium (5 mM NaCl, 0.17 mM KCl, 0.33 mM CaCl_2_ and 0.33 mM MgSO_4_ in distilled water), and kept at 28 °C. For assessing acute (lethal) and developmental (teratogenic) toxicity, the embryos were treated with increasing concentrations of the tested compounds (0.125, 0.25, 1, 2, 6.25, 8, 10, and 12.5 μg/mL) 6 h postfertilization (hpf). The highest tested dose was 12.5 μg/mL due to solubility and DMSO concentration limitations (0.25% max). DMSO (0.25%) was used as negative control. Every 24 h, dead embryos were discarded and live embryos were photographed and inspected for signs of toxicity. Heart rate was assessed by counting heartbeats per minute, and differences between control and treated groups were analyzed using the Kruskal–Wallis test followed by Dunn’s multiple comparison post hoc test. Experiments were performed in triplicate, and 24 embryos were tested per concentration. At 120 hpf, embryos were photographed, anesthetized by the addition of 0.1% (w/v) tricaine solution (Sigma-Aldrich, St. Louis, MO), and killed by freezing at −20 °C.

### 4.8 Infection Model of *S. aureus* in Zebrafish Embryos

#### 4.8.1 *S. aureus* Culture and Preparation for Microinjection

*S. aureus* ATCC25923 cells were cultured overnight in LB medium. The overnight culture was diluted 1:500 in fresh LB medium and incubated at 37 °C with shaking to promote growth until it reached the midexponential phase. After three rounds of centrifugation and washing in sterile PBS, the bacterial cells were labeled with 2 μM CellTracker RedCMTPX following the manufacturer’s instructions. The labeled cells were centrifuged again, washed three times in PBS to eliminate excess dye, and finally resuspended in a 2% polyvinylpyrrolidone (PVP) solution to achieve a final concentration of 5 × 10^8^ cells/mL for microinjection.

#### 4.8.2 Infection of Zebrafish Embryos

Infection experiment was performed according to the previously described procedure with minor modifications.^52^ Briefly, embryos at 26 h postfertilization (hpf) stage were manually dechorionated and kept at 28 °C. At 36 hpf, dechorionated embryos were anesthetized with 200 μg/mL of tricaine (MS-222) solution and microinjected with 1500–1600 labeled *S. aureus* cells into the pericardial cavity by a pneumatic picopump (PV820, World Precision Instruments, USA). Injected embryos were allowed to recover for 2 h at 28 °C, dead embryos were discarded, and live embryos were transferred into 24-well plates containing 1 mL of E3 medium and 10 embryos per well. The infected embryos were exposed to three concentrations (1 × MIC, 2 × MIC, and 4 × MIC) of compound **13**, and maintained at 31 °C by 120 hpf. The embryos injected with 2% PVP served as negative control. Ciprofloxacin was used as used an internal standard at the concentration 2 × MIC (MIC = 0.25 μg/mL, therefore 0.5 μg/mL was used). Twenty embryos were used per concentration, and experiment was performed three times. The survival and development of *S. aureus*-infected embryos was recorded every day until 120 hpf (4 days postinfection). The antistaphylococcal efficacy of the applied compounds was assessed based on the survival rate of the treated embryos in comparison to the untreated group. Survival experiments were evaluated using the Kaplan–Meier method. For comparisons between curves, log rank test was applied. The data analysis in the study was conducted using R software version 4.3.0. The level of significance was denoted as follows: *p < 0.05, **p < 0.01, ***p < 0.001.

### 4.9 *In Vitro* ADME Assays

#### 4.9.1 Kinetic Solubility in PBS, pH 7.4

A standard curve was generated by LC-MS/MS analysis of different concentrations of the test compounds. 5–20 μL of a 1 mM stock solution of the compound were added to 1 mL of PBS (pH 7.4), in increments of 5 μL each, until either turbidity was observed or a total of 20 μL of stock solution had been added (up to 2% DMSO). The solution was mixed using a Roto Spin at 50 rpm, 25 °C for 4 h to achieve equilibrium. The solution was filtered using 0.45 μm nylon injector filter to remove insoluble compound, and the filtrate was analyzed by LC-MS/MS for test compound concentration.

#### 4.9.2 Metabolic Stability in Mouse Liver Microsomes

A 1 μM solution of each test compound was prepared by diluting a 0.5 mM DMSO stock solution with PBS (pH 7.4). To each test solution, 110 μL of 10 mM NADPH was added and the samples incubated at 37 °C for 15 min. Subsequently, prewarmed mouse liver microsomes (27.5 μL; final protein conc. 0.5 mg/mL) were added and the samples incubated at 37 °C. Aliquots were withdrawn at 0, 15, 30, 60, 90, and 120 min, the reaction stopped by using chilled acetonitrile containing internal standard, the samples centrifuged, and the supernatants analyzed for test compound by LC-MS/MS. Percentage of compound remaining at each time point with respect to 0 min, and intrinsic clearance were calculated.

#### 4.9.3 LogD_7.4_ determination

LogD_7.4_ determination was outsourced to Wuxi AppTec Co., Ltd. The preparation of the 100 mM phosphate buffer (PB) at pH 7.4 began with the individual preparation of its components. A 100 mM NaH_2_PO_4_ solution was prepared by dissolving 5.999 g of NaH_2_PO_4_ in 500 mL of water, resulting in a solution with a pH of approximately 4.5. Similarly, a 100 mM Na_2_HPO_4_ solution was prepared by dissolving 7.098 g of Na_2_HPO_4_ in 500 mL of water, yielding a pH of about 9.4. To obtain the final 100 mM PB at pH 7.4, 15 mL of the Na_2_HPO_4_ solution was added into a 50 mL tube, and the pH was carefully adjusted to 7.4 ± 0.05 using the NaH_2_PO_4_ solution. For phase saturation, 100 mL of the prepared PB was mixed with 10 mL of 1-octanol, shaken vigorously, and left to stand overnight at room temperature to produce PB saturated with 1-octanol. Conversely, 1-octanol saturated with PB was prepared by mixing 100 mL of 1-octanol with 10 mL of PB (pH 7.4), followed by vigorous shaking and overnight equilibration at room temperature.

For the experimental procedure, 2 μL aliquots of stock solutions of test and control compounds were dispensed into tubes in duplicate. Each tube then received 149 μL of 1-octanol saturated with PB (pH 7.4), followed by the addition of 149 μL of PB (pH 7.4) saturated with 1-octanol into the corresponding tubes. The mixtures were vigorously mixed for 2 min and subsequently shaken for 1 h at 800 rpm at room temperature to allow partitioning. Afterward, the samples were centrifuged at 4000 rpm for 5 min at room temperature to achieve phase separation. Appropriate volumes of both the aqueous (buffer) and organic (1-octanol) layers were then collected, diluted as necessary, and analyzed using LC-MS/MS without the use of a calibration curve. Finally, the log D values for each compound were calculated based on the obtained analytical data.

#### 4.9.4 Plasma Protein Binding in CD-1 Mouse Plasma

Plasma protein binding was outsourced to Wuxi AppTec Co., Ltd. The dialysis setup utilized an HT-Dialysis plate (Model HTD 96b, Cat #1006) along with dialysis membranes (molecular weight cutoff 12–14 kDa, Cat #1101), both obtained from HT Dialysis LLC (Gales Ferry, CT). Phosphate-buffered saline (PBS) was prepared using BupHTM PBS packs from Thermo Fisher Scientific (Product #28372), which contained 0.1 M sodium phosphate and 0.15 M sodium chloride, yielding a pH of 7.2 when dissolved in 500 mL of deionized water. Prior to use, the pH was adjusted to 7.4 ± 0.1 using 1% phosphoric acid or 1 N sodium hydroxide, and the solution was stored at 4 °C for up to 1 month. The stop solution consisted of acetonitrile containing tolbutamide and labetalol, each at 250 nM, for both test and control compounds.

On the day of the experiment, plasma samples were thawed under cold running water and centrifuged at 3220 × g for 5 min to remove clots. Dialysis membrane strips were soaked in ultrapure water at room temperature for approximately 1 h, then separated and further soaked in an ethanol:water mixture (20:80, v/v) for about 20 min. These membranes were either used immediately or stored at 2–8 °C for up to 1 month, and prior to use, they were rinsed and soaked again in ultrapure water for 20 min. Working solutions (400 μM) of both test and control compounds were prepared by diluting 4 μL of stock solution with 96 μL DMSO. Loading matrix solutions (2 μM) were then prepared by diluting 3 μL of the working solution with 597 μL of blank matrix and mixing thoroughly.

For the assay, 50 μL aliquots of loading matrix containing test or control compounds were transferred in triplicate into a sample collection plate and mixed with an equal volume of blank PBS to achieve a final volume of 100 μL at a 1:1 (v/v) matrix-to-PBS ratio. A 500 μL stop solution was added to these T_0_ samples, followed by sealing and shaking at 800 rpm for 10 min before storage at 2–8 °C. The dialysis apparatus was assembled according to the manufacturer’s instructions. Subsequently, 100 μL of loading matrix was added to the donor side of each dialysis well in triplicate, while 100 μL of PBS was added to the receiver side. The plate was incubated at 37 °C in a humidified incubator with 5% CO_2_ and rotated at approximately 100 rpm for 4 h. After dialysis, 50 μL samples from both donor and receiver compartments were transferred into new plates, mixed with equal volumes of the opposite blank solution to maintain a 1:1 ratio, and treated with 500 μL stop solution. The mixtures were vortexed, centrifuged at 4000 rpm for about 20 min, and 100 μL of the supernatant was collected for LC-MS/MS analysis. Blank samples were prepared similarly using blank matrix and PBS, ensuring matrix matching to the experimental species, and processed with stop solution containing internal standards following the same procedure.

### 4.10 *In Silico* Physicochemical Prediction

*In silico* physicochemical prediction of the synthesized compounds was done using Chemaxon, Chemicalize online platform. cLogP, cLogD_7.4_ and tPSA were predicted. LipE was calculated using the available *S. aureus* DNA gyrase IC_50_ values of the synthesized compounds and the predicted cLogD_7.4_, according to the following formula:

$${\mathrm{LipE}=\mathrm{pIC}}_{50}-{\mathrm{cLogD}}_{7.4}$$

### 4.11 Molecular Dynamics Simulations

All-atom molecular dynamics (MD) simulations were performed according to a previously described protocol, with some minor modifications.^13^ The AMBER20 package was used instead of AMBER18.^57,58^ The MD simulations were performed for compounds **6**, **13**, **20**, **24**, and **25**, as well as gepotidacin, using the docking poses from *S. aureus* DNA Gyrase (PDB ID: 6Z1A). MD production simulations were performed on the canonical (*NVT*) ensemble using periodic boundary conditions on fully unrestrained systems in total duration of 500 ns p*er* system using a 2 fs time step. MD trajectory analysis was performed separately for each system, utilizing the VMD software package, *cpptraj* module of AmberTools, as well as UCSF Chimera.^59–61^ MD trajectory snapshots were saved for each complex by sampling the production trajectory at 100, 300, and 500 ns. Root-mean-square deviation (RMSD) and radius of gyration (Rg) calculations were conducted for each simulated system to monitor their stability and robustness over the entire simulation time.

## Supplementary Material





## Notes

Molecular formula strings (CSV)
Additional tables and figures, NMR spectra, and HPLC spectra (PDF)

## Abbreviations

CV: crystal violet
FET: fish embryo toxicity assay
Gepo: gepotidacin
GyrA: DNA gyrase subunit A
GyrB: DNA gyrase subunit B
HepG2: human hepatocellular carcinoma cell line
HUVEC: human umbilical vein endothelial cells
LHS: left-hand-side
MD: molecular dynamics
MMPA: matched molecular pair analysis
NBTI(s): novel bacterial topoisomerase inhibitor(s)
ParC: topoisomerase IV subunit C
PPB: plasma protein bidning
RHS: right-hand-side
TopoIV: topoisomerase IV
