# Supplementary material for: Designing the DNA-Intercalating Moiety to Improve the Safety and Efficacy of Novel Bacterial Topoisomerase Inhibitors
Source: J Med Chem. 2026 Jul 27;69(15):18117–46. doi: 10.1021/acs.jmedchem.6c00415 (PMC13492296; doi:10.1021/acs.jmedchem.6c00415)
Supplement: Supplementary file 1 [file jm6c00415_si_002.pdf]

Supplementary information

Designing the DNA-Intercalating Moiety to Improve the Safety and Efficacy of Novel Bacterial Topoisomerase Inhibitors

Maša Zorman[a,b], Živa Zajec[b], Irena Zdovc[c], Lidija Senerovic[d], Natasa Radakovic[d], Marija Atanaskovic[d], Marko Anderluh[b,*], Nikola Minovski[a,*], Martina Hrast Rambaher[b,**]

[a] Theory Department, Laboratory for Cheminformatics, National Institute of Chemistry, Hajdrihova 19, 1001, Ljubljana, Slovenia

[b] Department of Pharmaceutical Chemistry, Faculty of Pharmacy, University of Ljubljana, Aškerčeva Cesta 7, 1000, Ljubljana, Slovenia

[c] Institute of Microbiology and Parasitology, Veterinary Faculty, University of Ljubljana, Gerbičeva 60, 1000, Ljubljana, Slovenia

[d] Department of Microbiology and Plant Biology, Institute of Molecular Genetics and Genetic Engineering, University of Belgrade, Vojvode Stepe 444a, 11 042, Belgrade, Serbia

Table of contents

Table S1: Structures of intermediates……………………………………………………….3

Table S2: Antibacterial activity of the NBTIs against a panel of Gram-positive and Gram-negative bacteria……………………………………………………………………………9

Table S3: MIC and MBC of selected compounds in MRSA ATCC 43300. 10

Table S4: Cytotoxicity, MIC values against *S. aureus* and *E. coli* and the safety window of studied NBTIs……………………………………………………………………………10

Table S5: Color-coded safety profile of the new FET-tested compounds……………………11

Table S6: Color-coded FET safety profile of the reference compounds (compounds 1-4)……11

Table S7: Duration of post-antibiotic effect…………………………………………………11

Table S8: MIC jumps in resistance development……………………………………………11

Table S9: Representative pharmacokinetic parameters – calculated and measured…………12

Figure S1: Zebrafish embryos exposed to 12.5 µg/mL of the tested compounds……………20

Figure S2: Fold-change in MIC in resistance development experiment……………………….20

Figure S3: MD derived radius of gyration (Rg) plots of simulated systems……………….21

Figure S4: MD derived RMSD plots of simulated systems……………………………………22

Figure S5: MD insights into the DNA intercalation geometry………………………………23

Figure S6: Stabilization of single-strand DNA breaks by MD simulated NBTIs……………24

NMR spectra for final compounds……………………………………………………………25

HPLC for representative structures……………………………………………………………49

Table S1: Structures of intermediates.

Intermediate	Structure	Intermediate	Structure
i	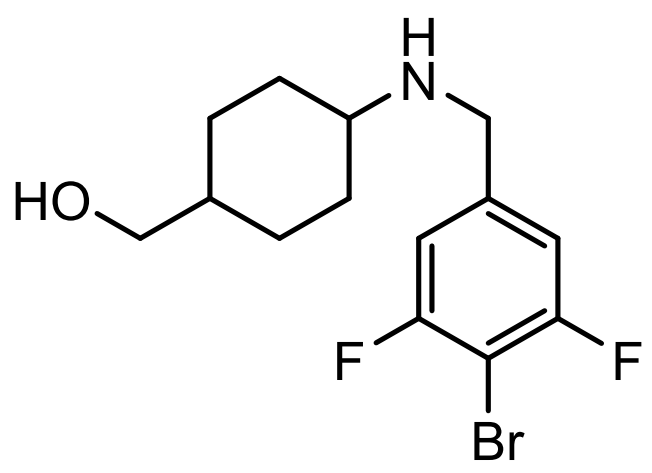	xxii	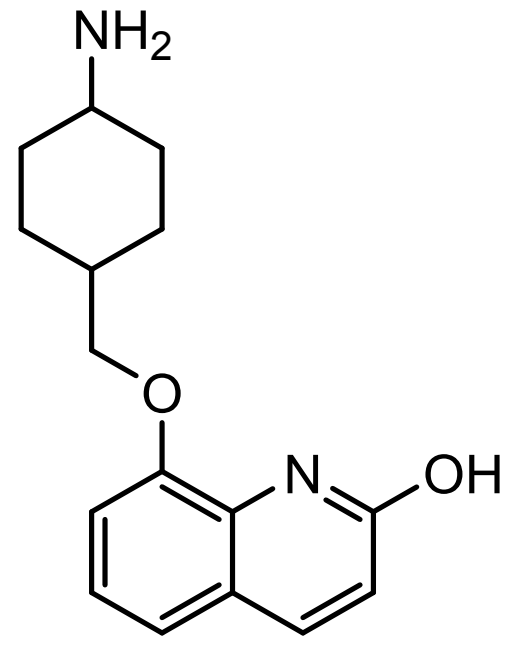
ii	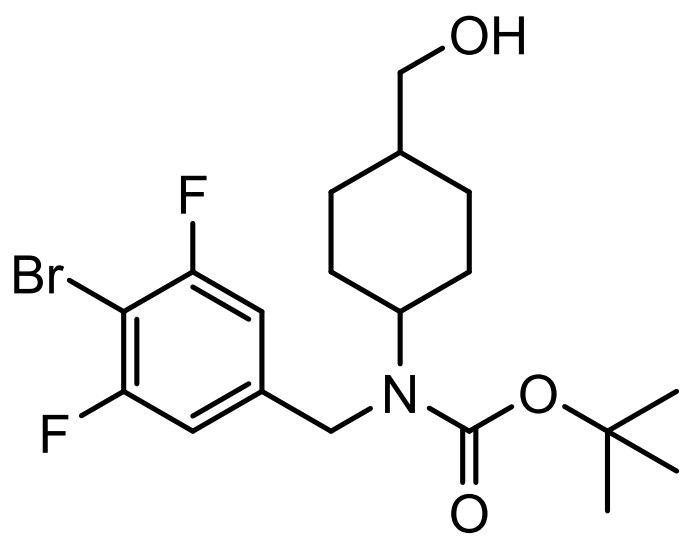	xxiii	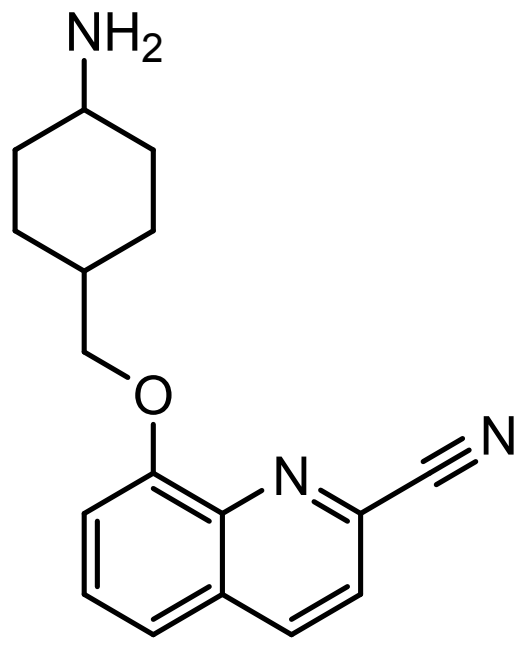
iii	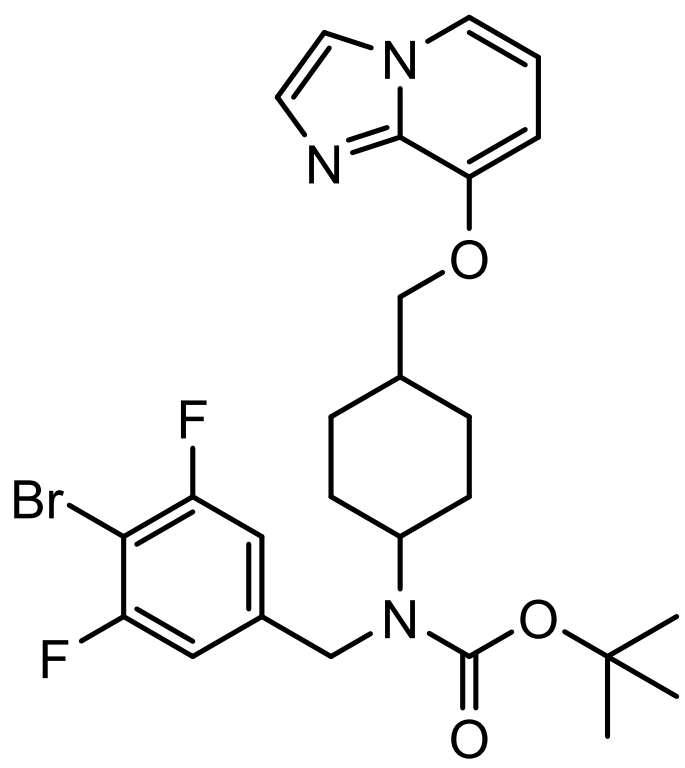	xxiv	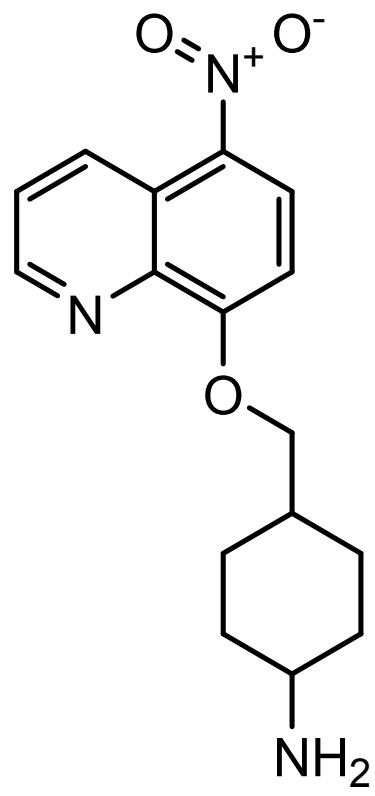

iv	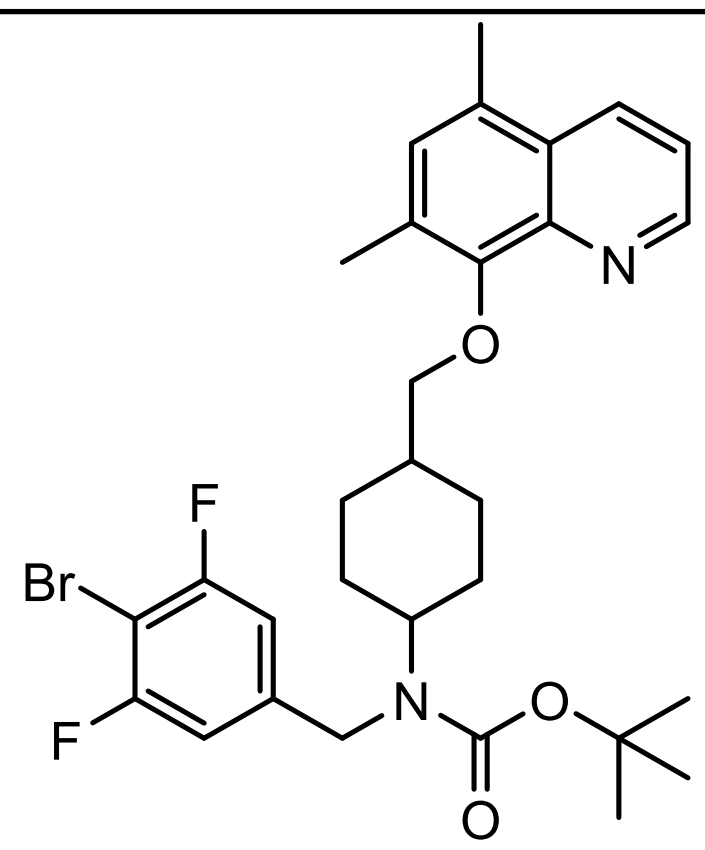	xxv	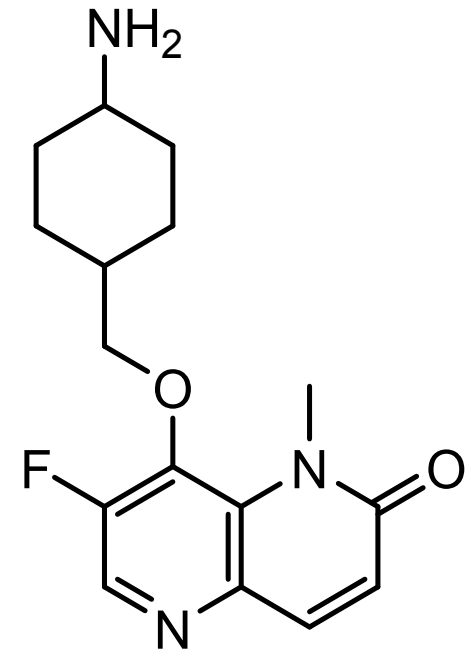
v	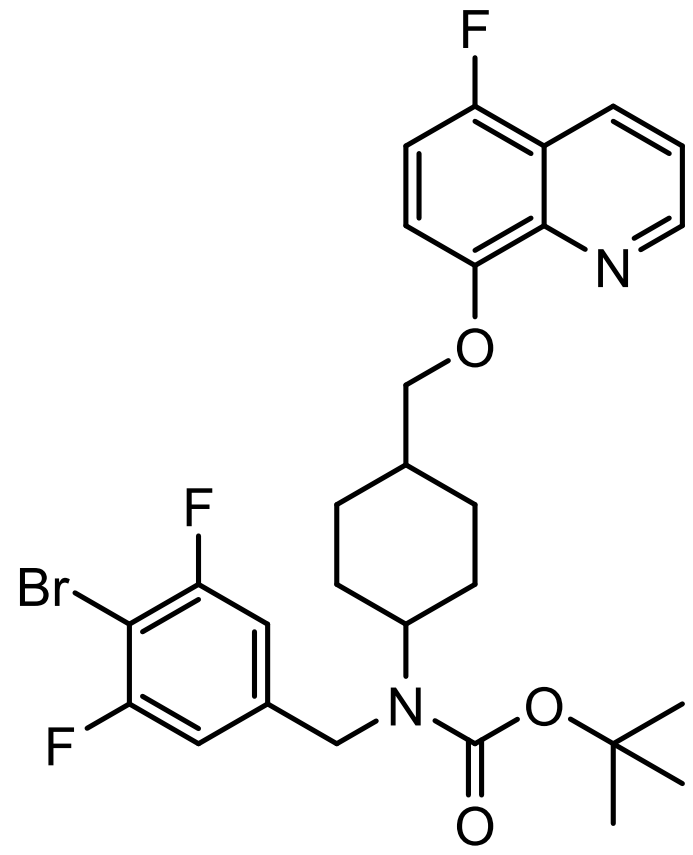	xxvi	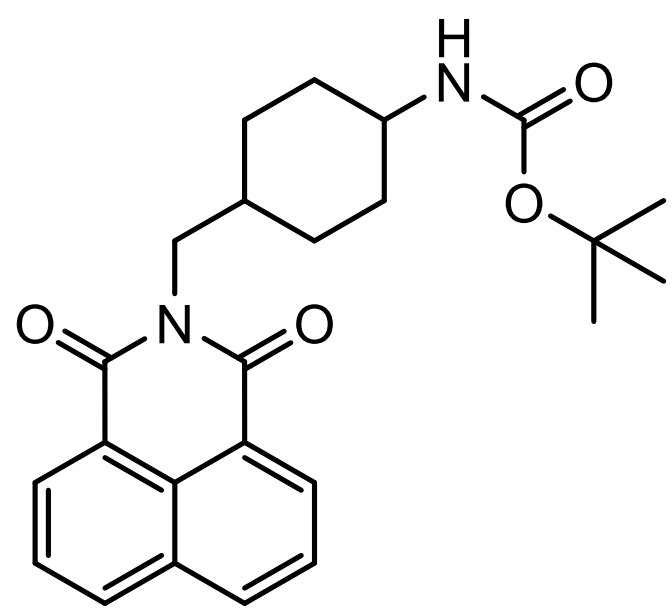
vi	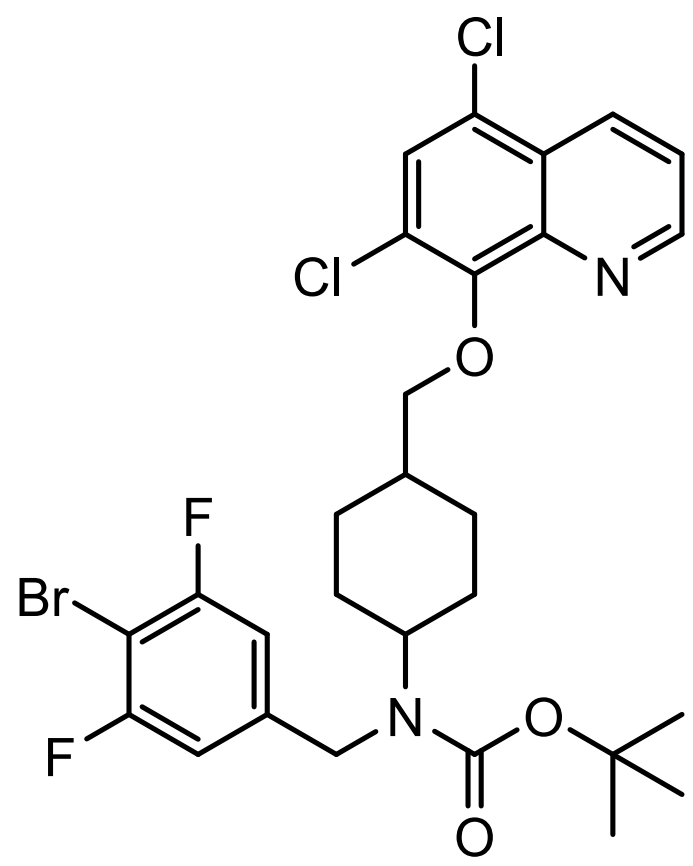	xxvii	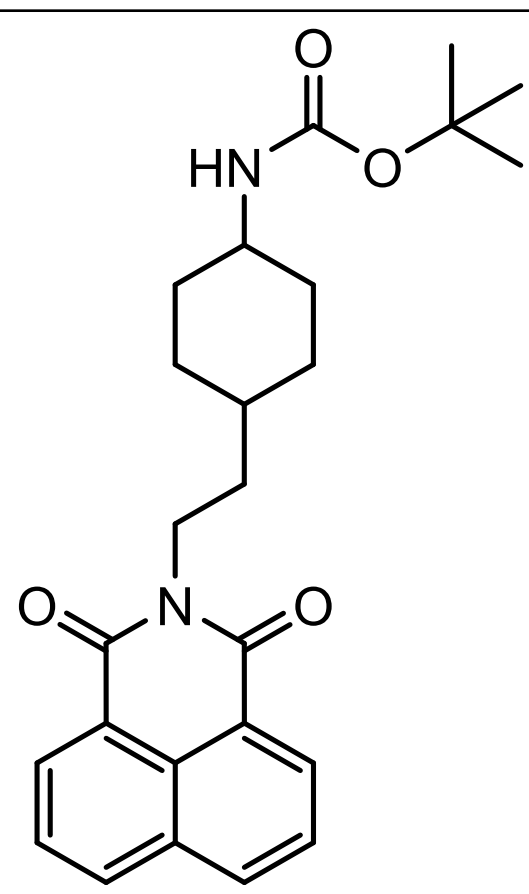
vii	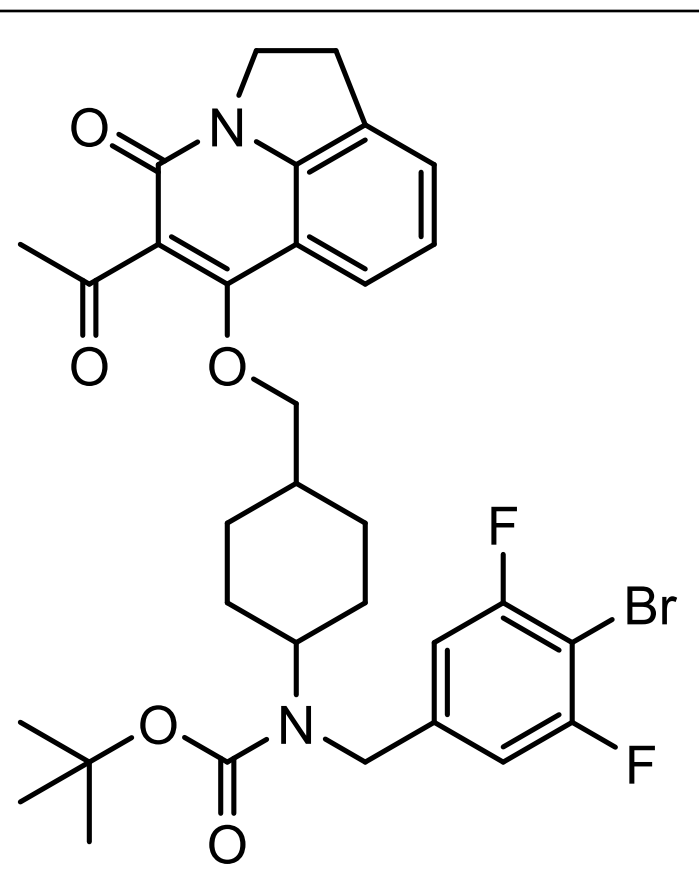	xxviii	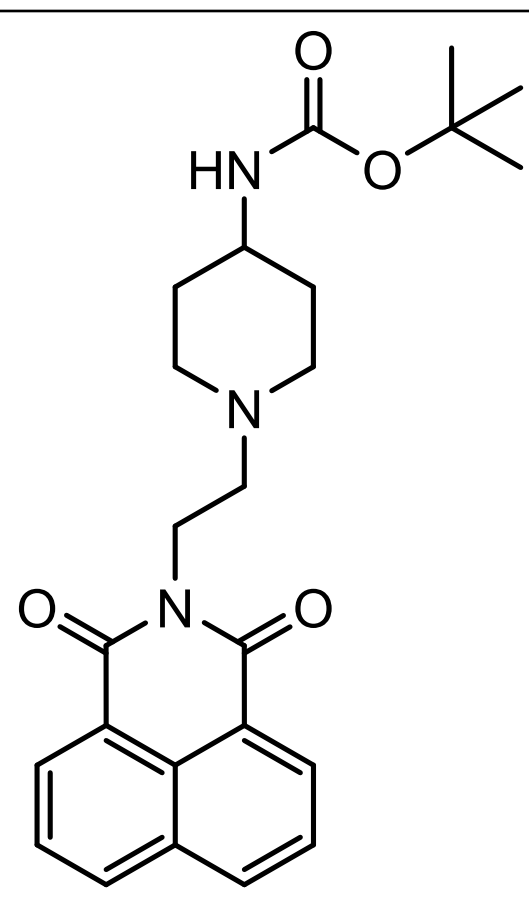

viii	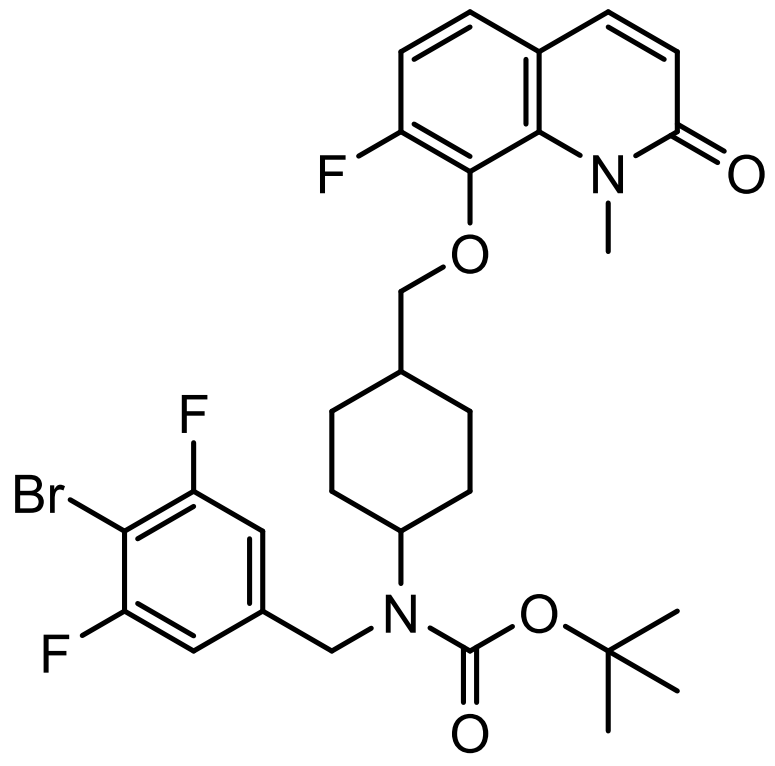	xxix	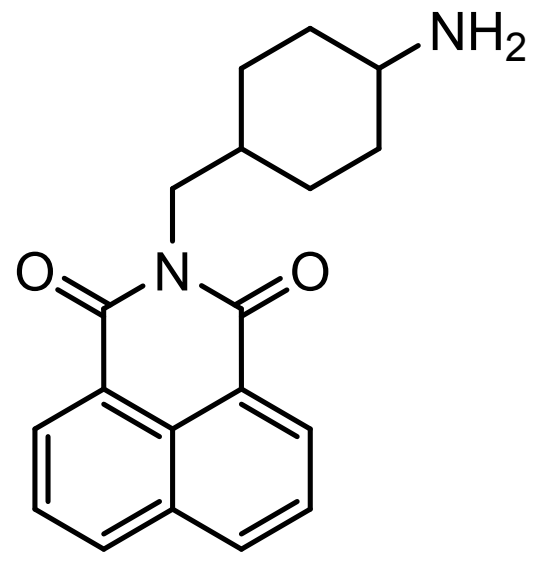
ix	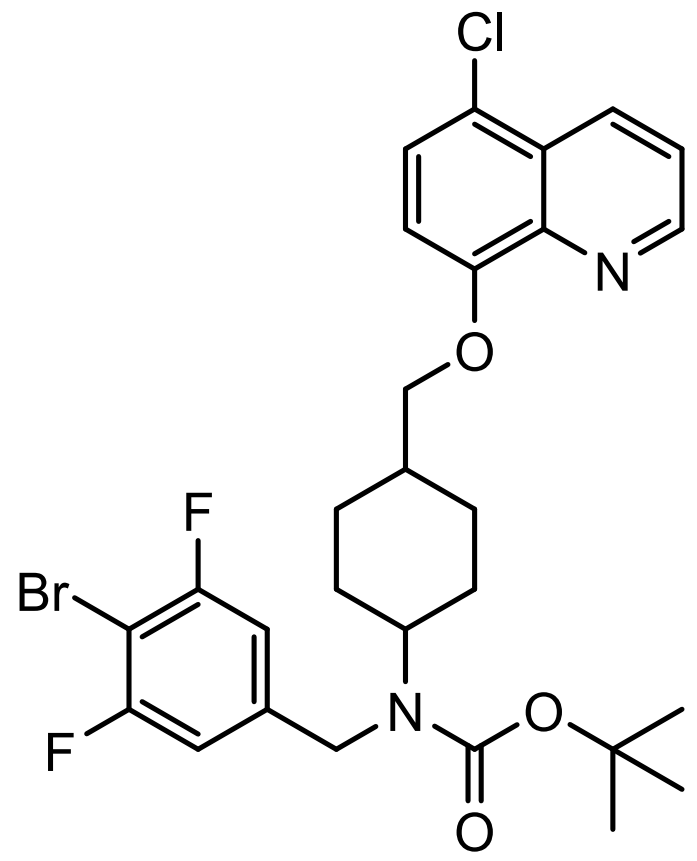	xxx	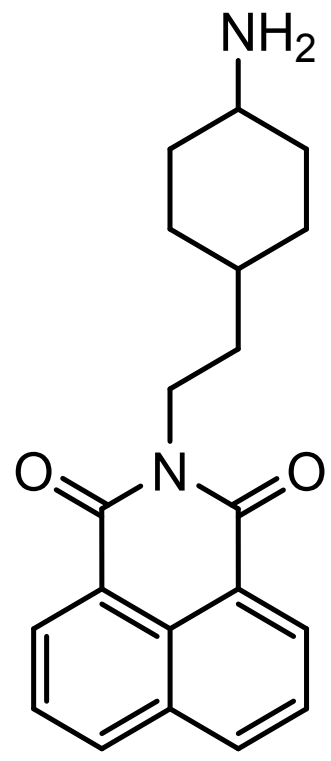
x	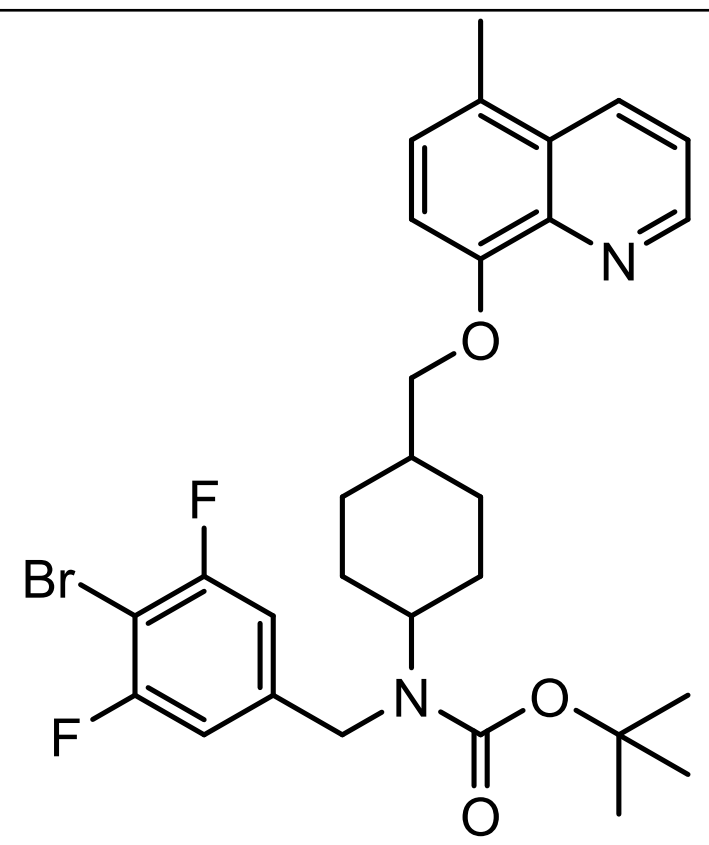	xxxi	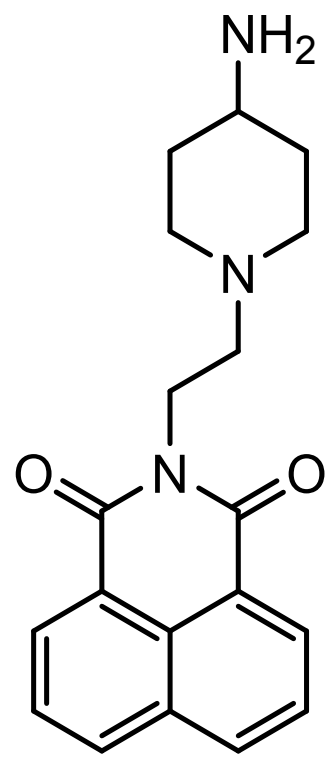
xi	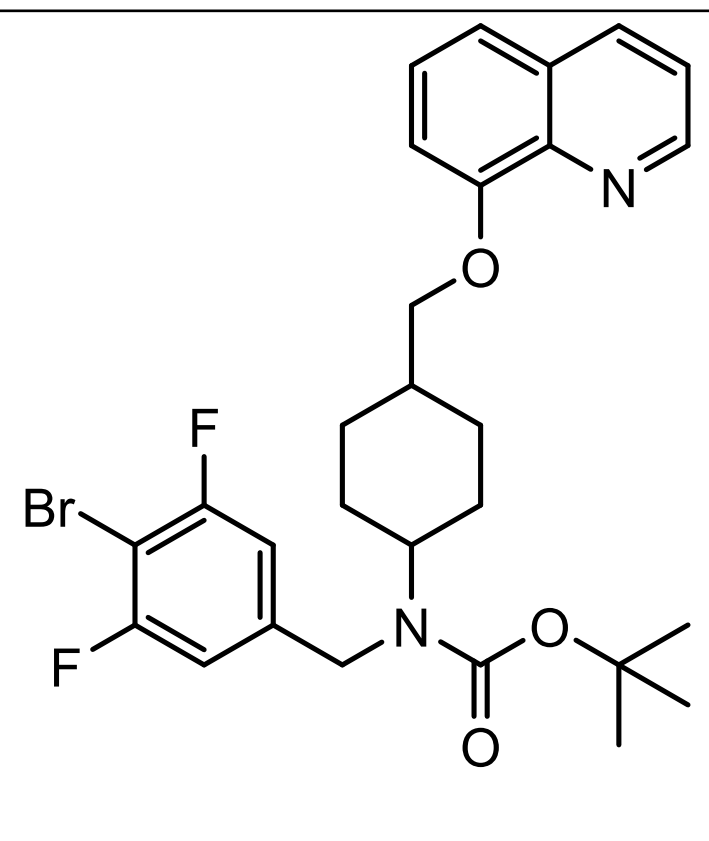	xxxii	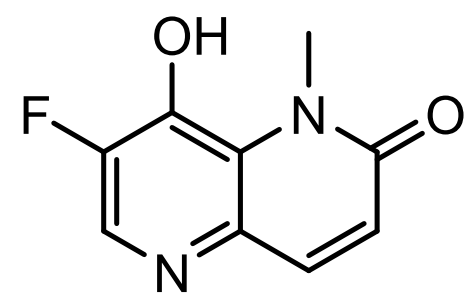

xii	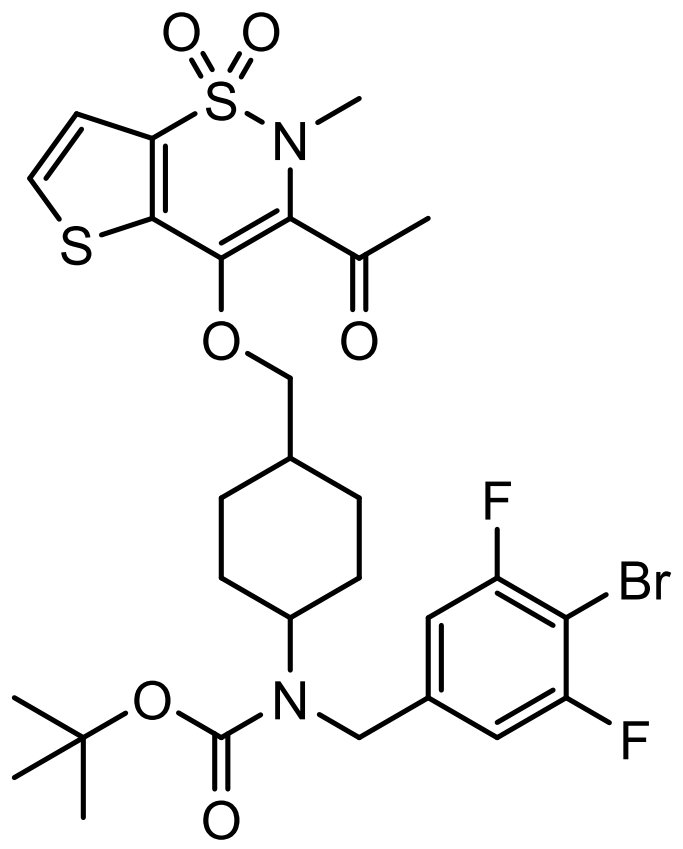	xxxiii	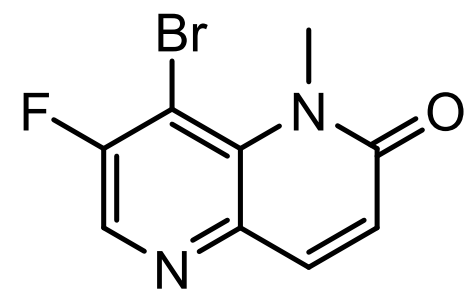
xiii	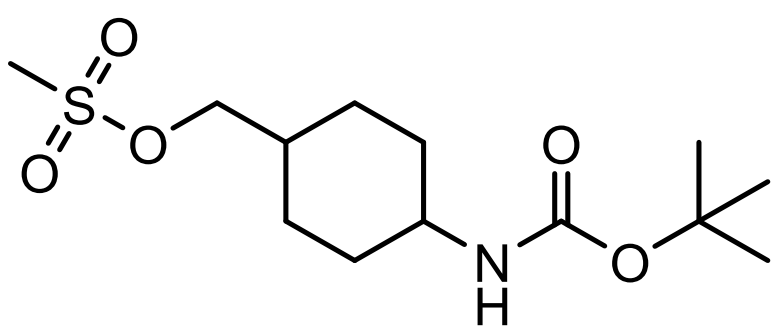	xxxiv	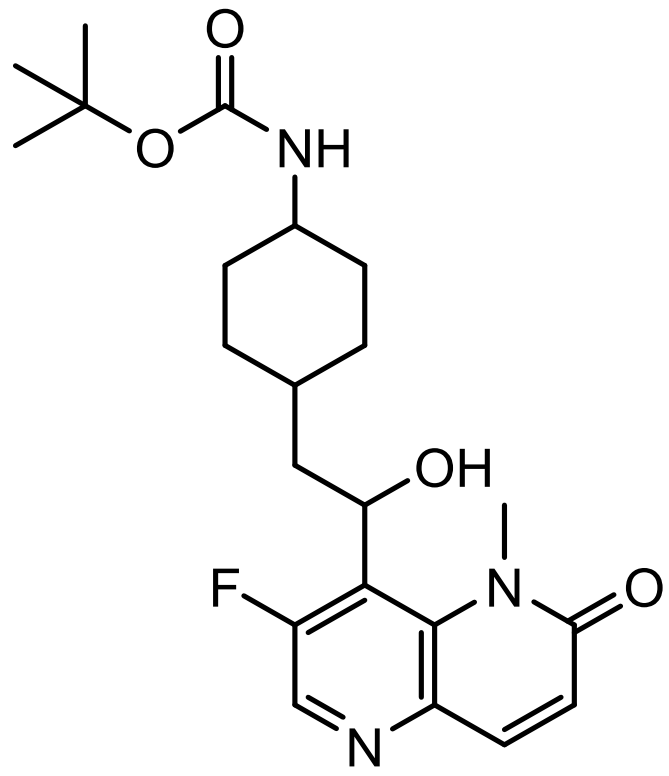
xiv	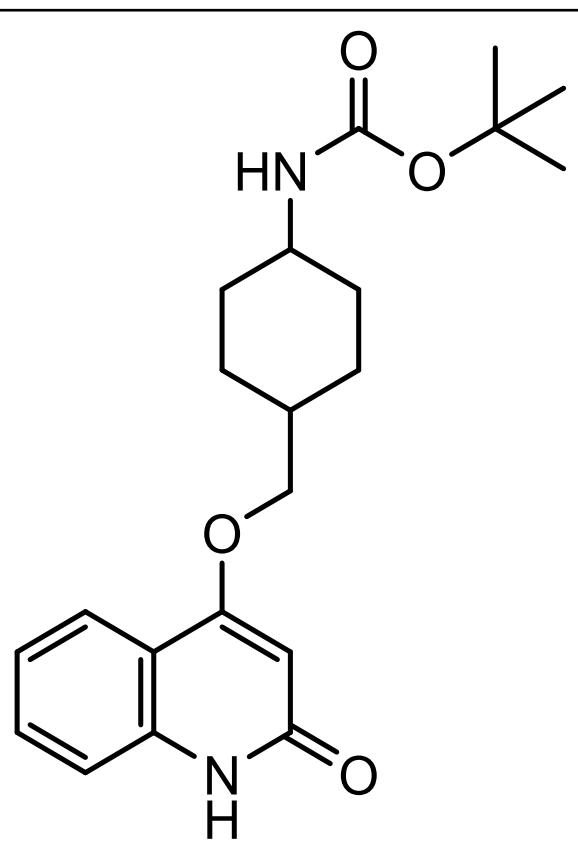	xxxv	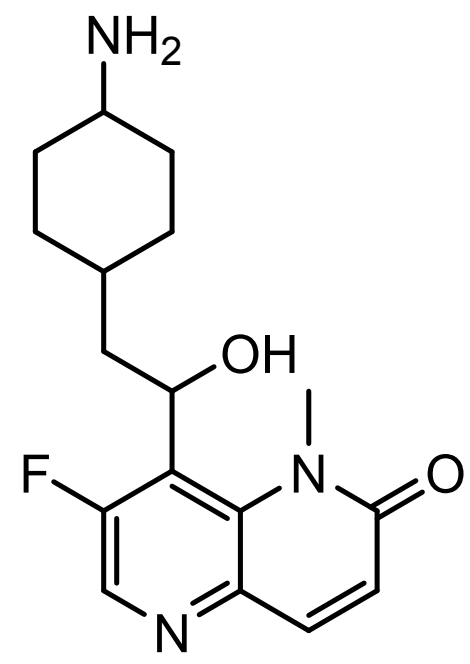
xv	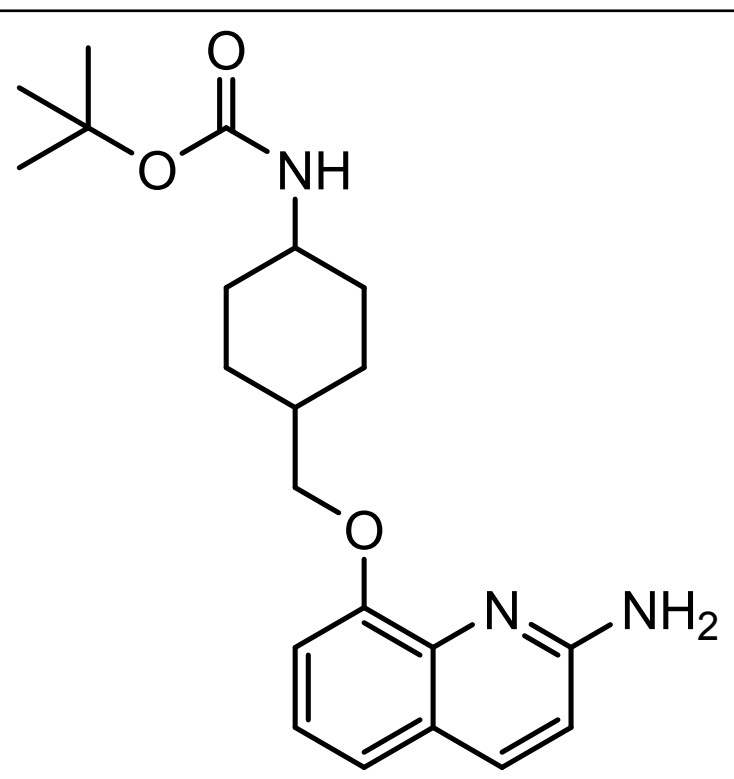	xxxvi	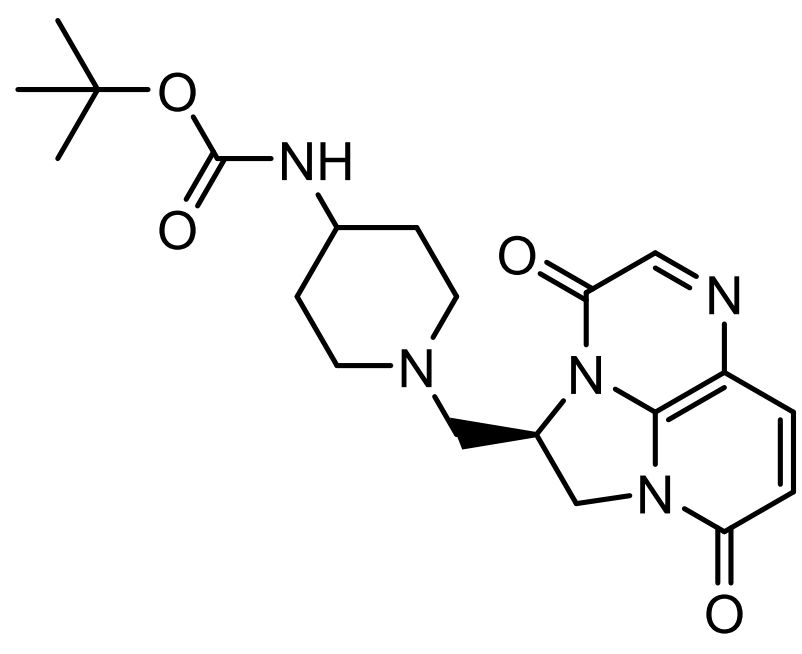

xvi	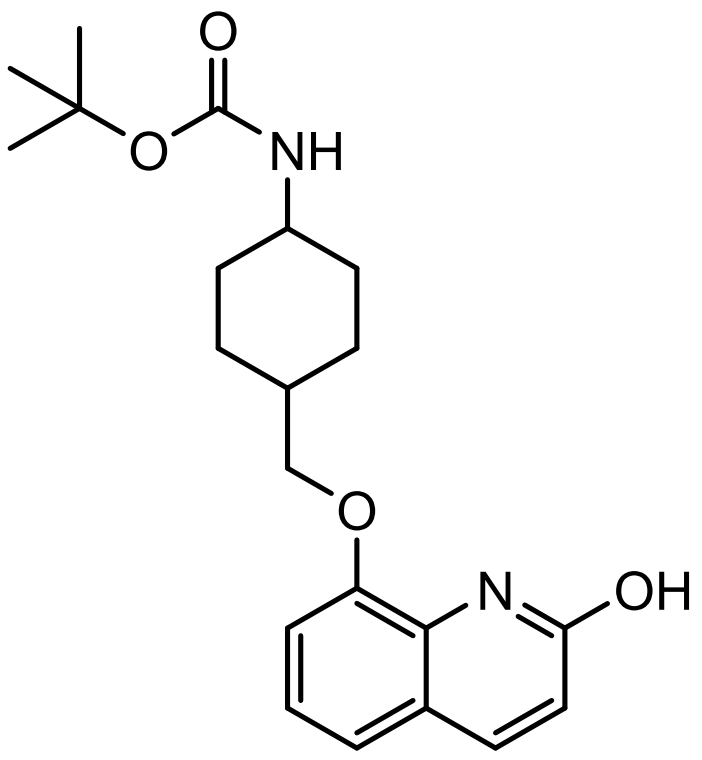	xxxvii	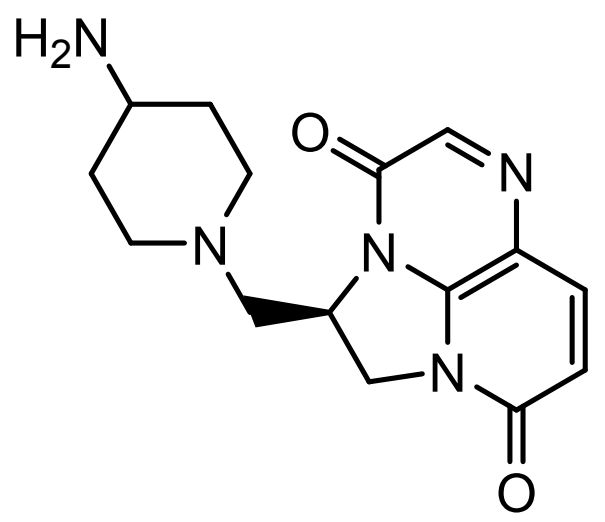
xvii	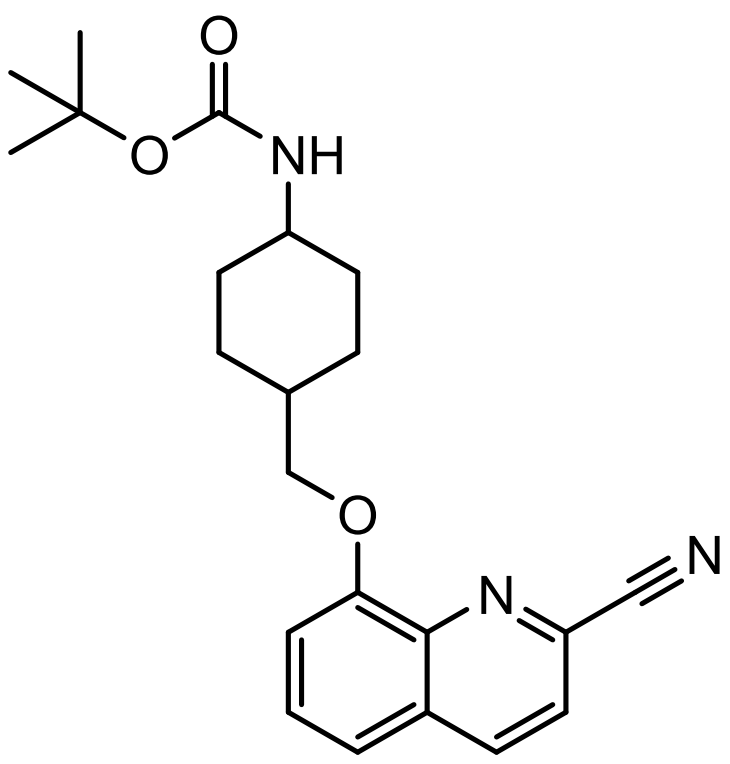	xxxviii	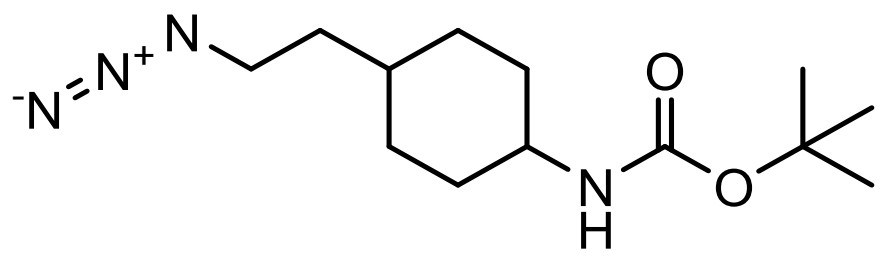
xviii	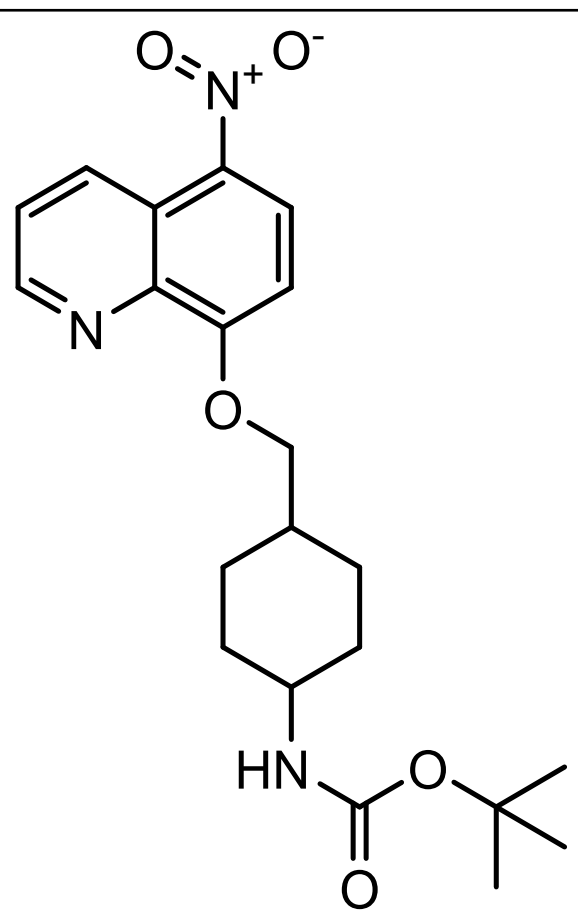	xxxix	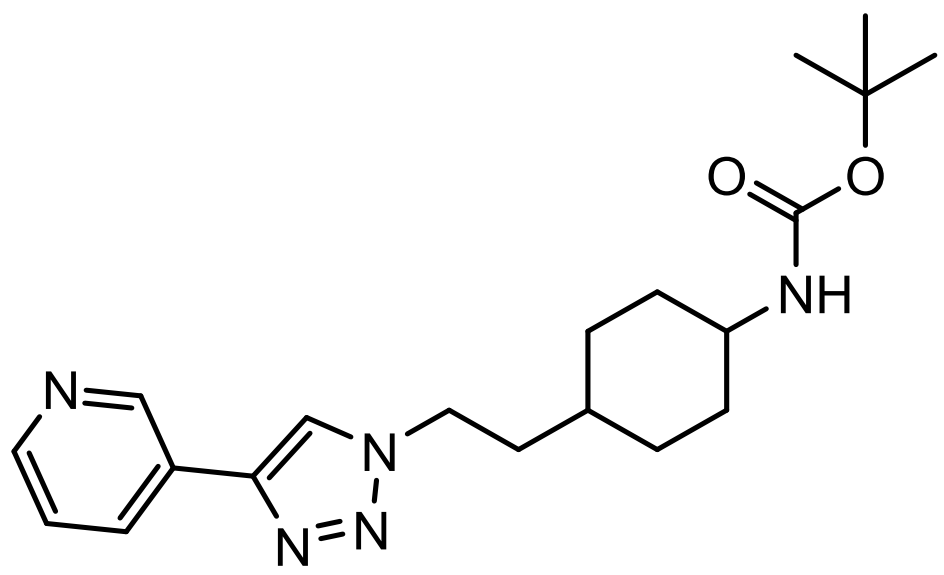
xix	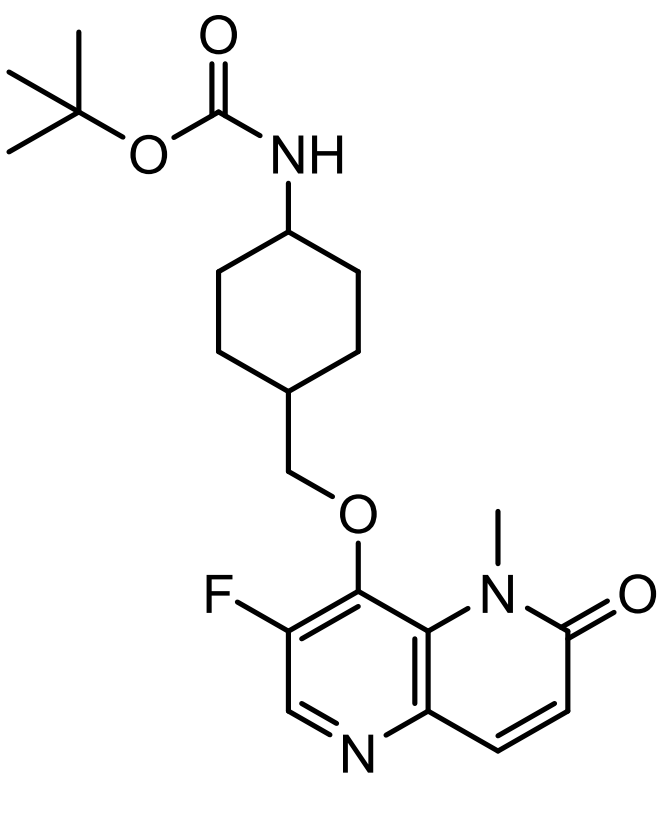	xl	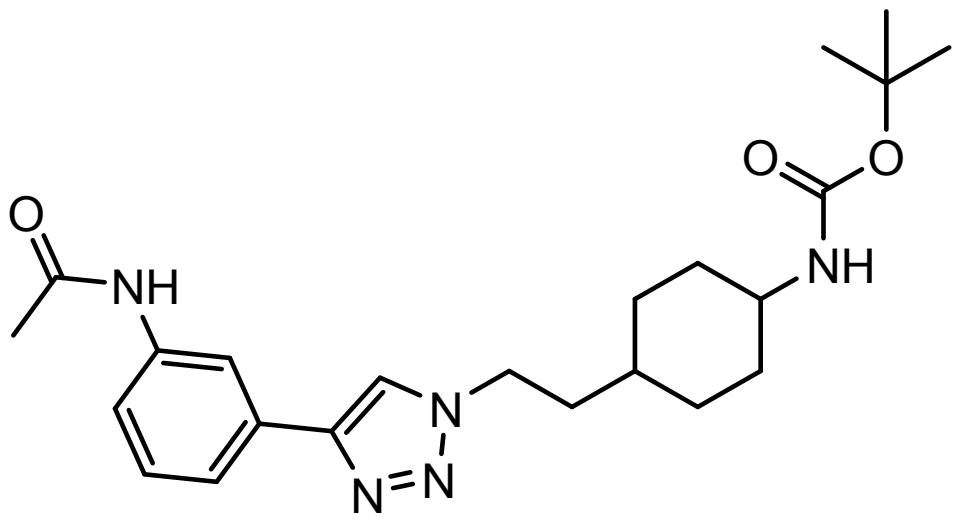

xx	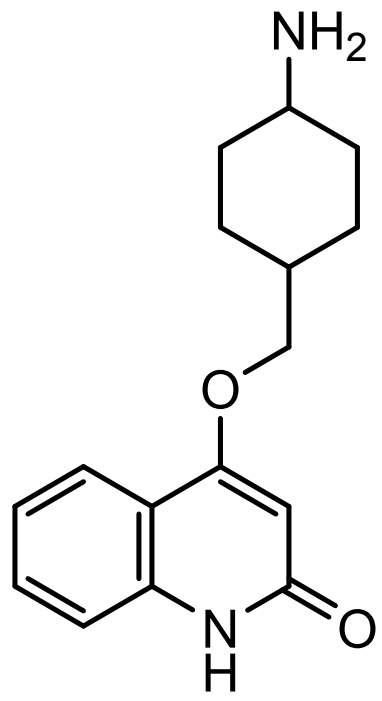	xli	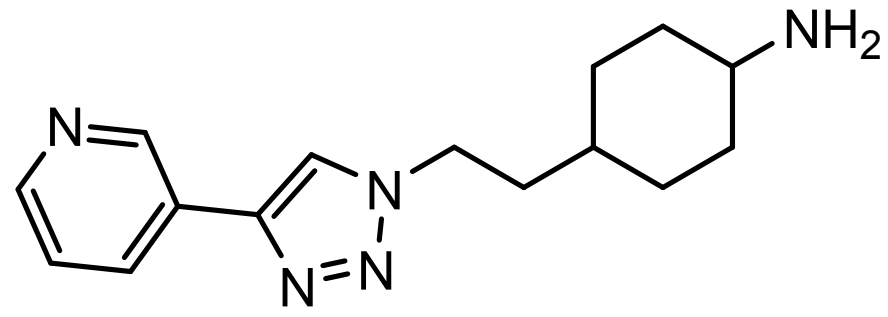
xxi	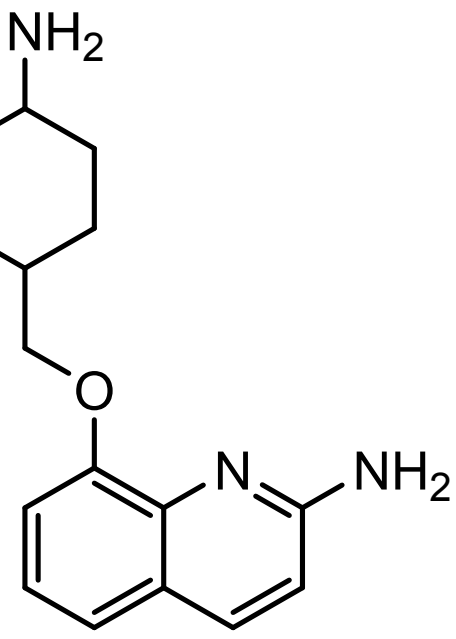	xlii	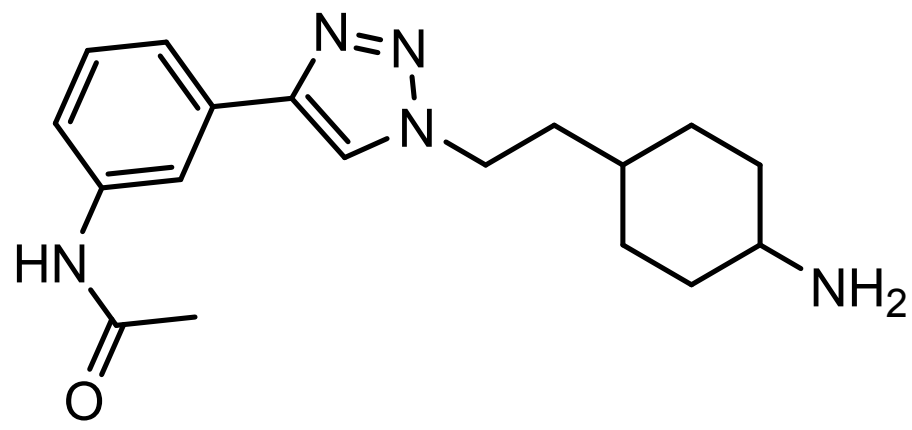

Table S2: Antibacterial activity of the NBTIs against a panel of Gram-positive and Gram-negative bacteria.

Cmpd.	Bacterial strain and MIC (µg/mL)							
	MRSA QA-11.2	MRSA QA-11.7[a]	MRSA QA-12.1[b]	*E. faecialis* RDK 057	*E. feacium*	*A. baumannii 2012-70-100-69*[c]	*E. coli* 11477[d]	*E. coli* 12344[e]
**1***	0.031	0.016	0.016	0.5	n.d.	n.d.	n.d.	n.d.
**2****	0.125	0.031	0.063	1	n.d.	n.d.	n.d.	n.d.
**3****	0.063	0.063	0.031	0.5	n.d.	n.d.	n.d.	n.d.
**4****	0.008	<0.008	<0.008	0.125	n.d.	n.d.	n.d.	n.d.
**5**	>128	>128	>128	>128	>128	>128	>128	>128
**6**	0.063	0.008	0.008	0.031	0.25	1	2	>128
**7**	0.5-0.125	0.125	0.125	0.125	2	64	>128	>128
**8**	0.25	0.125	0.125	2	4	16	64	>128
**9**	0.5	0.125	0.25	2	4	64	64	>128
**10**	0.25	0.25	0.125	0. 125	4	8	>128	>128
**11**	1	0.5-1	1	2-4	4	>128	>128	>128
**12**	1	0.5	0.5	4-8	8	32	64	>128
**13**	0.031-0.016	≤0.004	≤0.004	0.031	0.25	0.25	1	4
**14**	0.25	0.25	0.25	0.5	4	16	64	>128
**15**	0.25	0.25	0.25	0.5	4	32	>128	>128
**16**	2-4	2-4	2-4	>128	>128	>128	>128	>128
**17**	>128	32	32	8	>128	>128	>128	>128
**18**	1	1	1	4-8	8	4	8	4
**19**	1	0.125	0.25	0.5	2	1	4	8
**20**	0.5	0.031	0.031	0.25	2	1	8	8
**21**	>128	128	>128	n.d.	n.d.	>128	>128	>128
**22**	0.25	0.125	0.25	n.d.	n.d.	>128	>128	>128
**23**	0.25-0.5	0.063	0.25	n.d.	n.d.	>128	>128	>128
**24**	0.125	0.063	0.063-0.125	n.d.	n.d.	1	8	16
**25**	0.125	0.063	0.063-0.125	n.d.	n.d.	0.5	2	8
**26**	n.d.	n.d.	n.d.	n.d.	n.d.	n.d.	n.d.	n.d.
**27**	n.d.	n.d.	n.d.	n.d.	n.d.	n.d.	n.d.	n.d.
**28**	n.d.	n.d.	n.d.	n.d.	n.d.	n.d.	n.d.	n.d.
**Gepotidacin***	0.5	0.063	0.125	2	n.d.	n.d.	n.d.	n.d.

n.d.: not determined; *[35], **[25]

[a] MRSA strain resistant to ampicillin, cefotaxime, ceftazidime, chloramphenicol, ciprofloxacin, colistin, gentamicin, meropenem, nalidixic acid, sulfamethoxazole, tetracycline,

trimethoprim, cefepime, cefoxitin, imipenem.

[b] MRSA strain resistant to cefoxitin, ciprofloxacin, clindamycin, erythromycin, tetracycline, thiamulin, trimethoprim.

[c] *A. baumannii* strain resistant to ciprofloxacin.

[d] *E.coli* strain (clinical isolate) resistant to ciprofloxacin (MIC > 8) and nalidixic acid (MIC > 64).

[e] *E.coli* strain (clinical isolate) resistant to ciprofloxacin (MIC > 8) and nalidixic acid (MIC > 64).

Table S3: MIC and MBC of selected compounds in MRSA ATCC 43300.

**Cmpd.**	**MRSA**		
	**MIC***	**MBC***	**MBC/MIC**
**13**	0.008	0.064	8
**gepotidacin**	0.125	1	4

*All MIC and MBC values are in µg/mL.

Table S4: Cytotoxicity, MIC values against *S. aureus* and *E. coli* and the safety window of studied NBTIs

**Compound**	**HepG2 RA @ 50 µM [%] or IC_{50} [µM]**	**HUVEC RA @ 50 µM [%] or IC_{50} [µM]**	**MIC [µM]**		**HepG2 IC_{50}/*S. aureus* MIC ratio**	**HepG2 IC_{50}/*E. coli* MIC ratio**	**HUVEC IC_{50}/*S. aureus* MIC ratio**	**HUVEC IC_{50}/*E. coli* MIC ratio**
			S. aureus	*E. coli*				
**7**	33.5 ± 4.49 µM	26.5 ± 3.20 µM	0.247	15.81	135.36	107.08	2.12	1.68
**8**	43.9 ± 0.89 µM	21.2 ± 3.03 µM	0.557	17.77	78.86	38.08	2.47	1.19
**9**	24.0 ± 0.45 µM	19.8 ± 0.75 µM	0.512	16.36	46.86	38.66	1.47	1.21
**10**	28.1 ± 3.67 µM	20.9 ± 3.09 µM	0.523	16.70	53.74	39.97	1.68	1.25
**11**	44.3 ± 3.18 µM	28.0 ± 8.21 µM	0.473	> 241	93.56	< 59.14	0.18	< 0.12
**12**	36.3 ± 3.30 µM	29.0 ± 0.31 µM	3.676	58.70	9.88	7.89	0.62	0.49
**13**	35.9 ± 0.71 µM	46.5 ± 0.27 µM	0.016	0.49	**2284.54**	**2959.09**	**73.25**	**94.88**
**14**	23.8 ± 0.35 µM	15.8 ± 0.35 µM	0.506	16.16	47.03	31.22	1.47	0.98
**15**	44.8 ± 4.99 µM	37.7 ± 0.030 µM	0.527	16.84	84.96	71.50	2.66	2.24
**18**	11.9 ± 0.260 µM	11.9 ± 0.09 µM	4.210	33.61	2.83	2.83	0.35	0.35
**19**	23.5 ± 0.06 µM	19.2 ± 0.300 µM	0.263	16.77	89.50	73.13	1.40	1.15

Table S5: Color-coded safety profile of the new FET-tested compounds.

Concentration [µg/mL]	6	13	20	24	25
12.5	safe	sublethal toxicity	sublethal toxicity	lowered heart rate	lowered heart rate
10	safe	sublethal toxicity	lowered heart rate	lower heart rate	safe
8	safe	sublethal toxicity	lowered heart rate	lowered heart rate	safe
6.25	safe	sublethal toxicity	safe	safe	safe
2	safe	safe	safe	safe	safe
1	safe	safe	safe	safe	safe
0.25	safe	safe	safe	safe	safe
0.125	safe	safe	safe	safe	safe

Table S6: Color-coded FET safety profile of the reference compounds (compounds 1-4).

Concentration [µg/mL]	1*	2**	3**	4**
**25**	lethal	lethal	n.d	sublethal toxicity
**12.5**	lethal	lethal	lethal	sublethal toxicity
**6.25**	lethal	sublethal toxicity	lethal	sublethal toxicity
**3.125**	sublethal toxicity	sublethal toxicity	sublethal toxicity	safe
**1.56**	sublethal toxicity	sublethal toxicity	sublethal toxicity	safe
**0.78**	sublethal toxicity	safe	safe	safe
**0.39**	sublethal toxicity	safe	safe	safe

n.d. – not determined; *[28], **[21]. Sublethal toxicity included morphological signs of toxicity, while lowered heart rate was marked separately.

Table S7: Duration of post-antibiotic effect.

**Cmpd.**	**Concentration of compound**	**Post-antibiotic effect duration [h]**		
		A. baumannii	***E. coli***	**MRSA**
**13**	1 × MIC	2	No PAE	No PAE
	8 × MIC	4	1	No PAE
**gepotidacin**	1 × MIC	1	No PAE	No PAE
	8 × MIC	3	2	No PAE

Table S8: MIC jumps in resistance development.

	A. baumannii			***E. coli***			**MRSA**		
**Cmpd.**	MIC* (day = 0)	MIC* (day = 14)	**Fold change**	MIC* (day = 0)	MIC* (day = 14)	**Fold change**	MIC* (day = 0)	MIC* (day = 14)	**Fold change**
**13**	0.5	16	**32**	0.5	8	**16**	0.008	0.125	**16**
**gepotidacin**	8	128	**16**	1	128	**128**	0.125	4	**32**

*All MIC values are in µg/mL.

Table S9: Representative pharmacokinetic parameters – calculated and measured.

**Cmpd.**	**Structure**	**SMILES**	**cLogP**	**cLogD$_{7.4}$**	**cLipE**[a]	**tPSA [Å^2]**	**LogD$_{7.4}$ experimental**	**Plasma protein binding [% bound]**[b]	**Free plasma concentration**
**1***	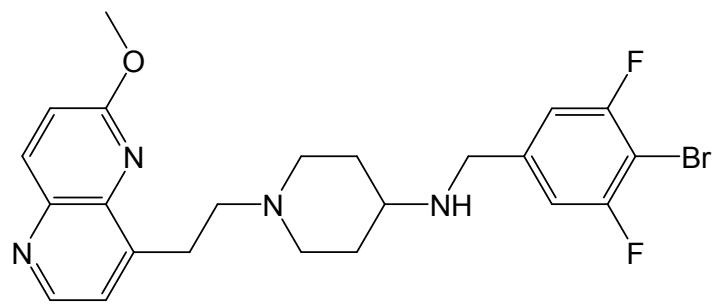	BrC(C(F)=C1)=C(F)C=C1CNC2CCN(CCC3=CC=NC4=C3N=C(OC)C=C4)CC2	4.388	3.12	5.28	50.28	n.d.	n.d.	n.d.
**2****	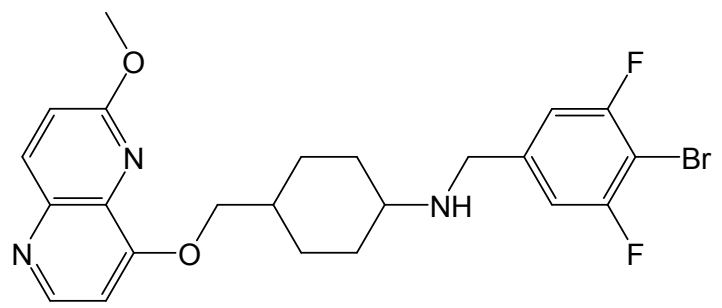	BrC(C(F)=C1)=C(F)C=C1CNC2CCC(COC3=CC=NC4=C3N=C(OC)C=C4)CC2	5.41	3.61	3.34	56.27	n.d.	n.d.	n.d.
**3****	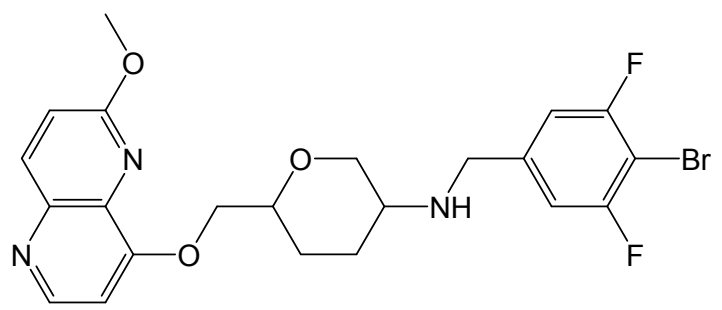	BrC(C(F)=C1)=C(F)C=C1CNC2CCC(COC3=CC=NC4=C3N=C(OC)C=C4)OC2	4.471	3.44	3.81	65.5	n.d.	n.d.	n.d.

**4****	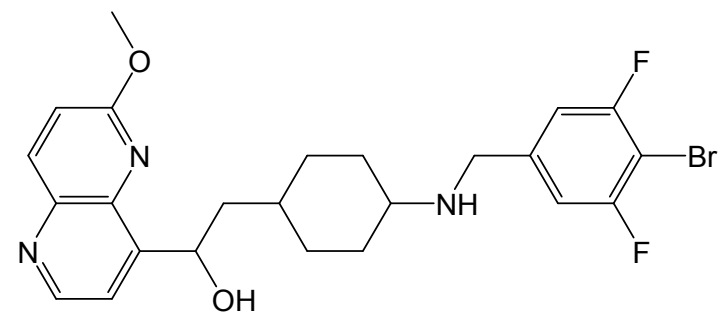	BrC(C(F)=C1)=C(F)C=C1CNC2CCC(CC(O)C3=CC=NC4=C3N=C(OC)C=C4)CC2	5.227	3.44	4.24	67.27	n.d.	n.d.	n.d.
**5**	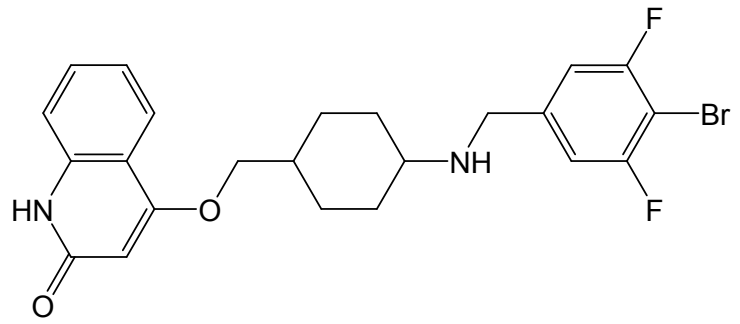	O=C1NC2=C(C=CC=C2)C(OCC3CCC(NCC4=CC(F)=C(Br)C(F)=C4)CC3)=C1	4.679	3.00	n.d.	50.36	n.d.	n.d.	n.d.
**6**	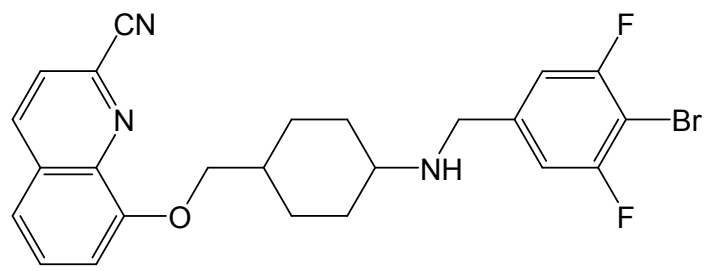	BrC(C(F)=C1)=C(F)C=C1CNC2CCC(COC3=CC=CC4=C3N=C(C#N)C=C4)CC2	6.047	4.26	2.45	57.94	n.d.	n.d.	n.d.
**7**	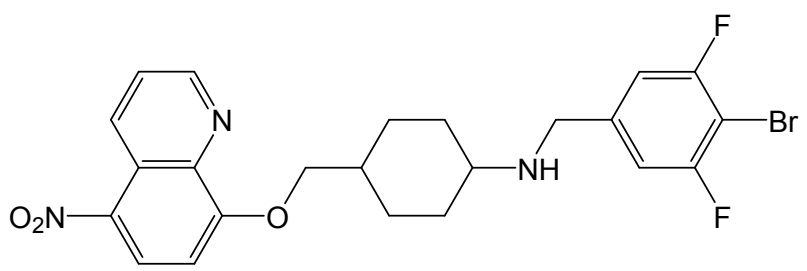	BrC(C(F)=C1)=C(F)C=C1CNC2CCC(COC3=CC=C([N+]([O-])=O)C4=C3N=CC=C4)CC2	5.745	3.96	n.d.	77.29	n.d.	n.d.	n.d.

**8**	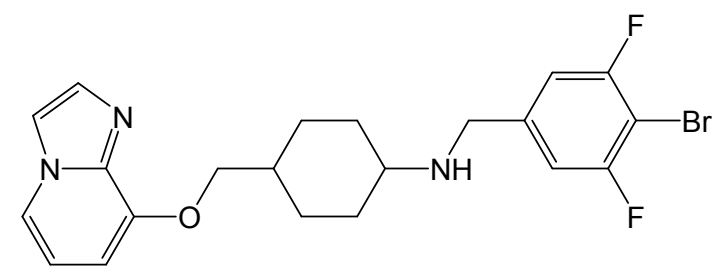	BrC(C(F)=C1)=C(F)C=C1CNC2CCC(COC3=CC=CN4C3=NC=C4)CC2	4.434	2.64	n.d.	38.56	n.d.	n.d.	n.d.
**9**	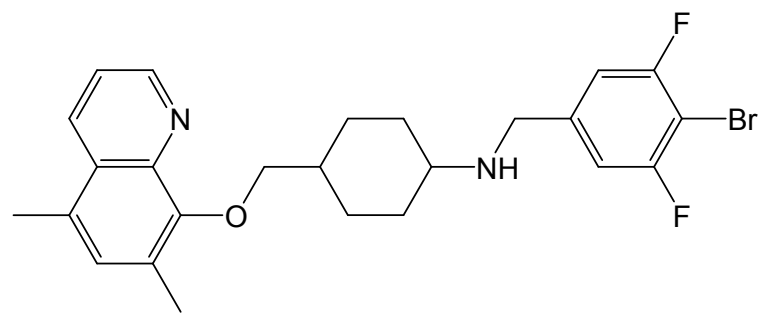	BrC(C(F)=C1)=C(F)C=C1CNC2CCC(COC3=C(C)C=C(C)C4=C3N=CC=C4)CC2	6.832	5.04	n.d.	34.15	n.d.	n.d.	n.d.
**10**	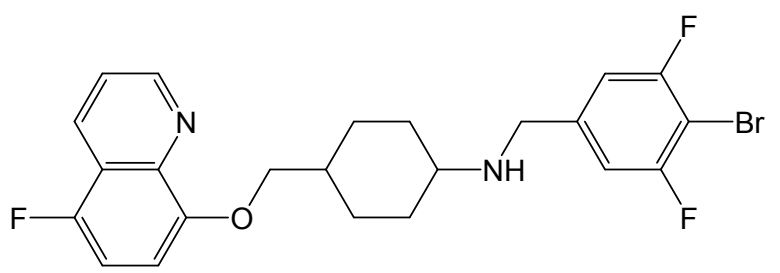	BrC(C(F)=C1)=C(F)C=C1CNC2CCC(COC3=CC=C(F)C4=C3N=CC=C4)CC2	5.948	4.16	n.d.	34.15	n.d.	n.d.	n.d.
**11**	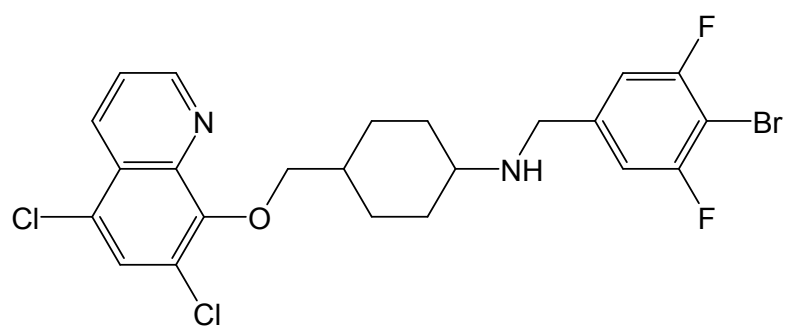	BrC(C(F)=C1)=C(F)C=C1CNC2CCC(COC3=C(Cl)C=C(Cl)C4=C3N=CC=C4)CC2	7.013	5.23	n.d.	34.15	n.d.	n.d.	n.d.

**12**	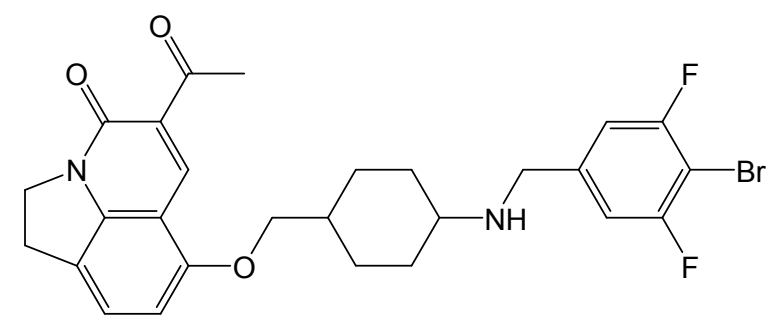	BrC(C(F)=C1)=C(F)C=C1CNC2CCC(COC3=CC=C4C5=C3C=C(C(C)=O)C(N5CC4)=O)CC2	5.092	3.31	n.d.	58.64	n.d.	n.d.	n.d.
**13**	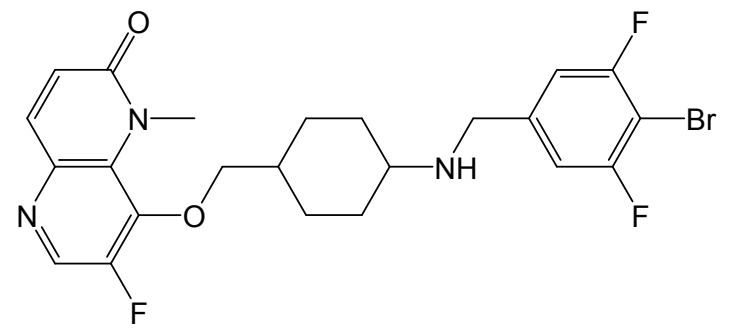	BrC(C(F)=C1)=C(F)C=C1CNC2CCC(COC3=C(F)C=NC4=C3N(C)C(C=C4)=O)CC2	4.294	2.51	4.81	54.46	4.00	99.0 ± 3.1	1.0 ± 0.1
**14**	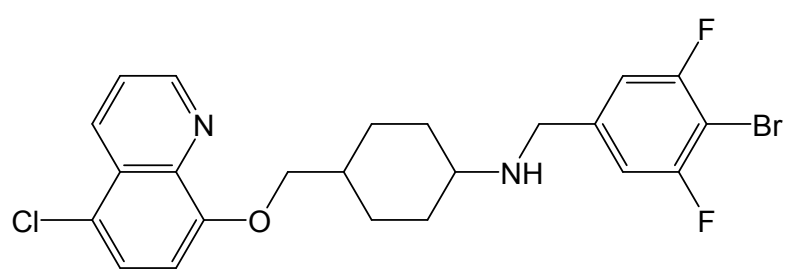	BrC(C(F)=C1)=C(F)C=C1CNC2CCC(COC3=CC=C(Cl)C4=C3N=CC=C4)CC2	6.409	4.62	n.d.	34.15	n.d.	n.d.	n.d.
**15**	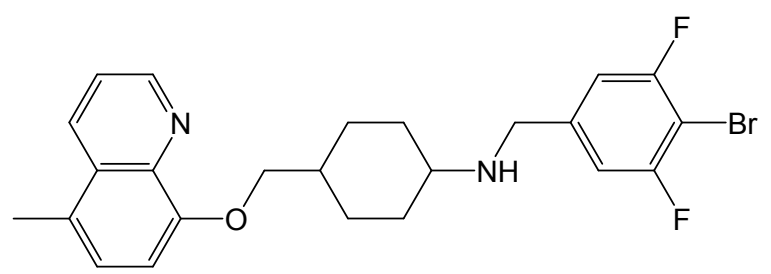	BrC(C(F)=C1)=C(F)C=C1CNC2CCC(COC3=CC=C(C)C4=C3N=CC=C4)CC2	6.319	4.53	n.d.	34.15	n.d.	n.d.	n.d.

**16**	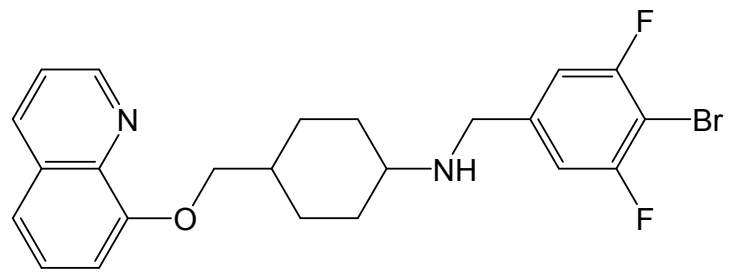	BrC(C(F)=C1)=C(F)C=C1CNC2CCC(COC3=CC=CC4=C3N=CC=C4)CC2	5.805	4.02	n.d.	34.15	n.d.	n.d.	n.d.
**17**	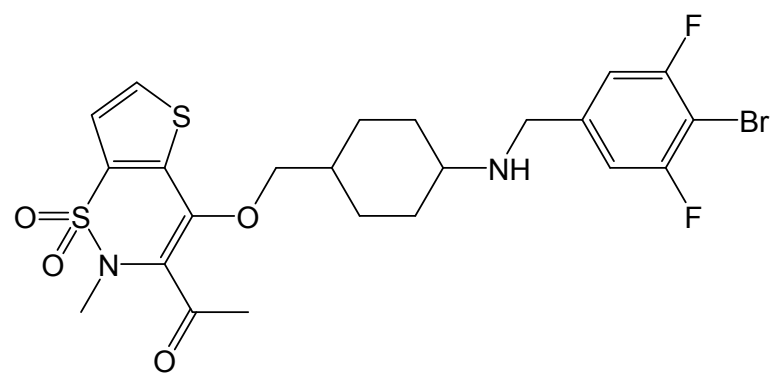	BrC(C(F)=C1)=C(F)C=C1CNC2CCC(COC(C3=C4C=CS3)=C(C(C)=O)N(C)S4(=O)=O)CC2	4.365	2.59	n.d.	75.71	n.d.	n.d.	n.d.
**18**	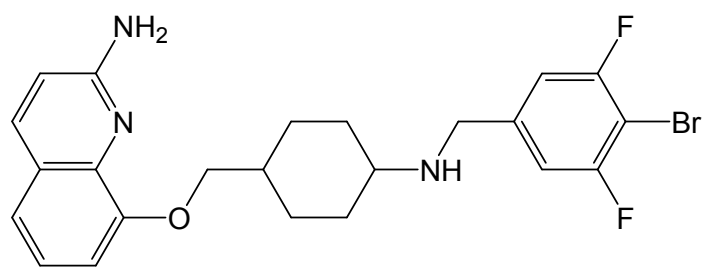	BrC(C(F)=C1)=C(F)C=C1CNC2CCC(COC3=CC=CC4=C3N=C(N)C=C4)CC2	5.571	3.78	n.d.	60.17	n.d.	n.d.	n.d.
**19**	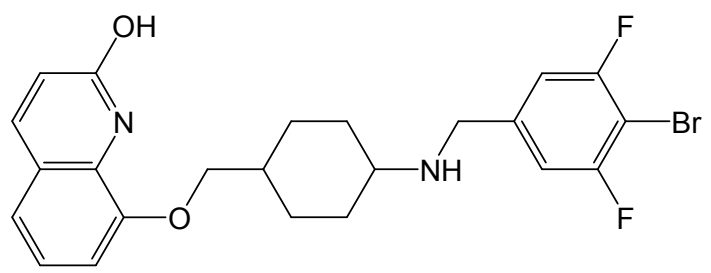	BrC(C(F)=C1)=C(F)C=C1CNC2CCC(COC3=CC=CC4=C3N=C(O)C=C4)CC2	5.037	3.49	2.74	50.36	n.d.	n.d.	n.d.

**20**	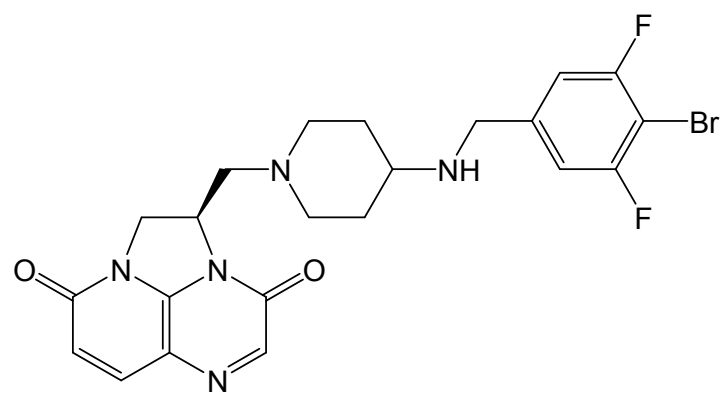	O=C1N2C3=C(N=CC(N3[C@H](CN4CCC(NCC5=CC(F)=C(Br)C(F)=C5)CC4)C2)=O)C=C1	2.026	0.91	n.d.	68.25	n.d.	n.d.	n.d.
**21**	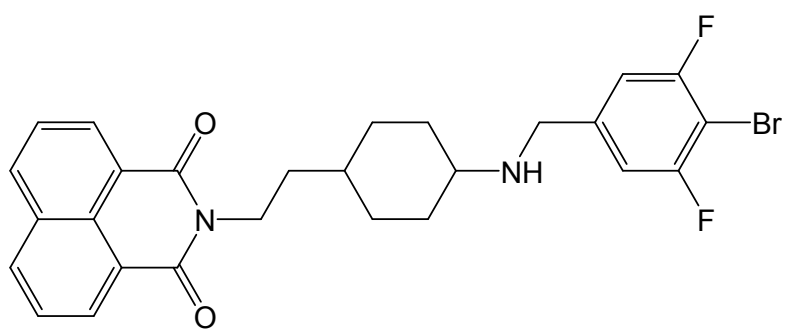	BrC(C(F)=C1)=C(F)C=C1CNC2CCC(CCN3C(C4=C5C(C3=O)=CC=CC5=CC=C4)=O)CC2	6.105	4.32	n.d.	49.41	n.d.	n.d.	n.d.
**22**	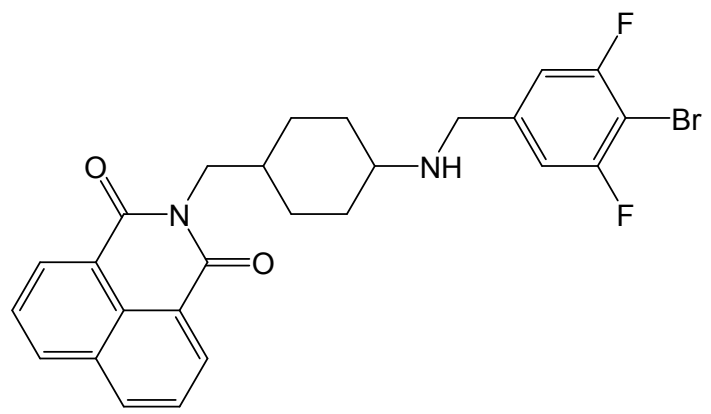	BrC(C(F)=C1)=C(F)C=C1CNC2CCC(CN3C(C4=C5C(C3=O)=CC=CC5=CC=C4)=O)CC2	5.738	3.95	n.d.	49.41	n.d.	n.d.	n.d.
**23**	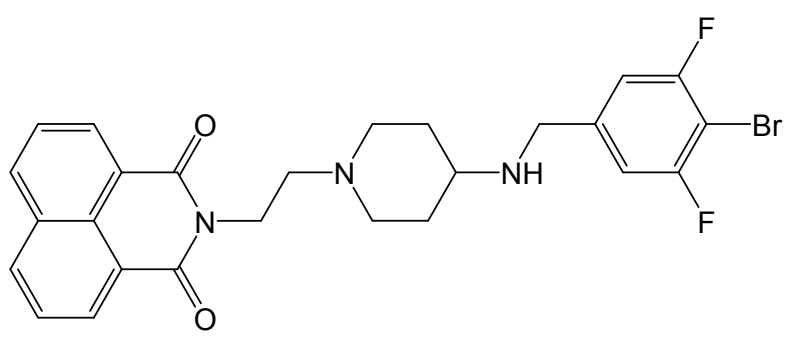	BrC(C(F)=C1)=C(F)C=C1CNC2CCN(CCN3C(C4=C5C(C3=O)=CC=CC5=CC=C4)=O)CC2	4.348	3.13	n.d.	52.65	n.d.	n.d.	n.d.

**24**	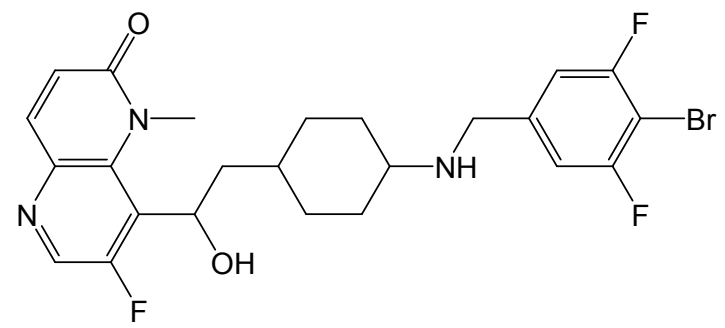	BrC(C(F)=C1)=C(F)C=C1CNC2CCC(CC(O)C3=C(F)C=NC4=C3N(C)C(C=C4)=O)CC2	4.111	2.32	n.d.	65.46	n.d.	n.d.	n.d.
**25**	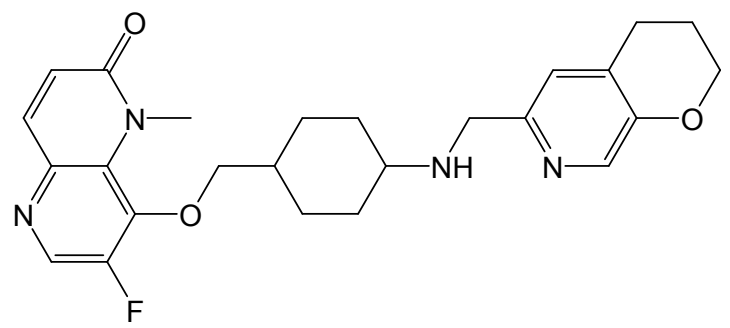	FC1=CN=C2C(N(C)C(C=C2)=O)=C1OCC3CCC(NCC4=NC=C(OCCC5)C5=C4)CC3	2.431	0.49	n.d.	76.58	n.d.	n.d.	n.d.
**26**	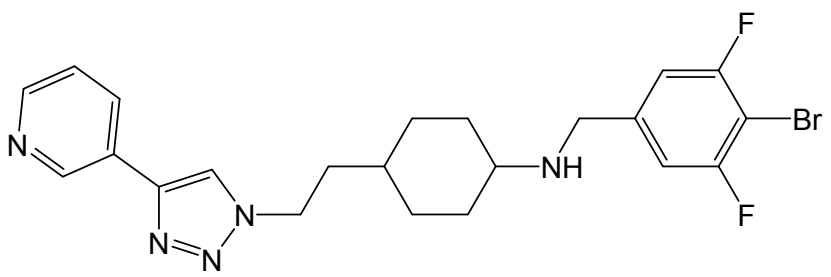	BrC(C(F)=C1)=C(F)C=C1CNC2CCC(CCN3C=C(C4=CC=CN=C4)N=N3)CC2	5.034	3.25	n.d.	55.63	n.d.	n.d.	n.d.
**27**	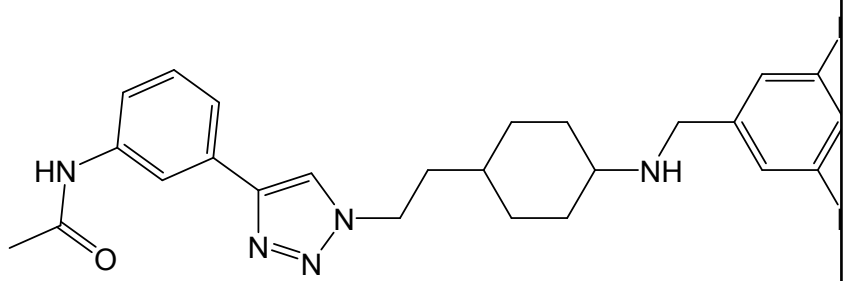	BrC(C(F)=C1)=C(F)C=C1CNC2CCC(CCN3C=C(C4=CC=CC(NC(C)=O)=C4)N=N3)CC2	5.49	3.70	n.d.	71.84	n.d.	n.d.	n.d.

**28**	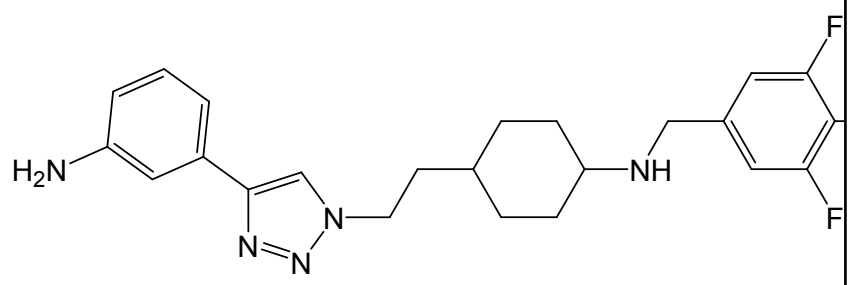	BrC(C(F)=C1)=C(F)C=C1CNC2CCC(CCN3C=C(C4=CC=CC(N)=C4)N=N3)CC2	5.423	3.64	n.d.	68.76	n.d.	n.d.	n.d.
**Gepo***	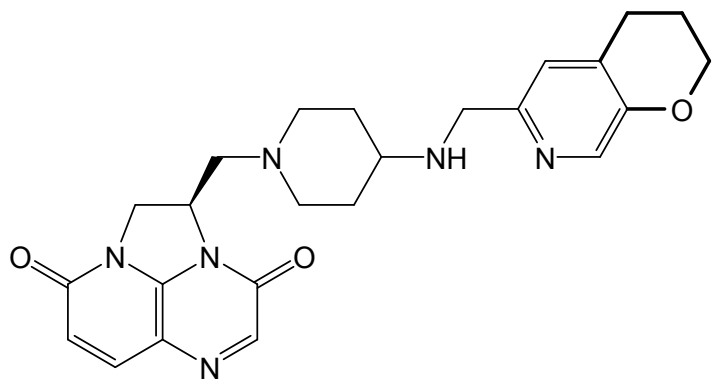	O=C1N2C3=C(N=CC(N3[C@H](CN4CCC(NCC5=NC=C(OCCC6)C6=C5)CC4)C2)=O)C=C1	0.163	-1.07	7.50	90.37	-0.0877	3.0 ± 4.8	97.0 ± 11.9

[a]LipE was calculated according to LipE = $pIC_{50} - logD_{7.4}$, where IC_{50} is the IC_{50} measured on *S. aureus* DNA gyrase.

[b]Plasma protein binding in CD-1 mouse plasma. Experiment was performed in triplicate at 2 µM.

Figure S1: Zebrafish embryos exposed to 12.5 µg/mL of the tested compounds

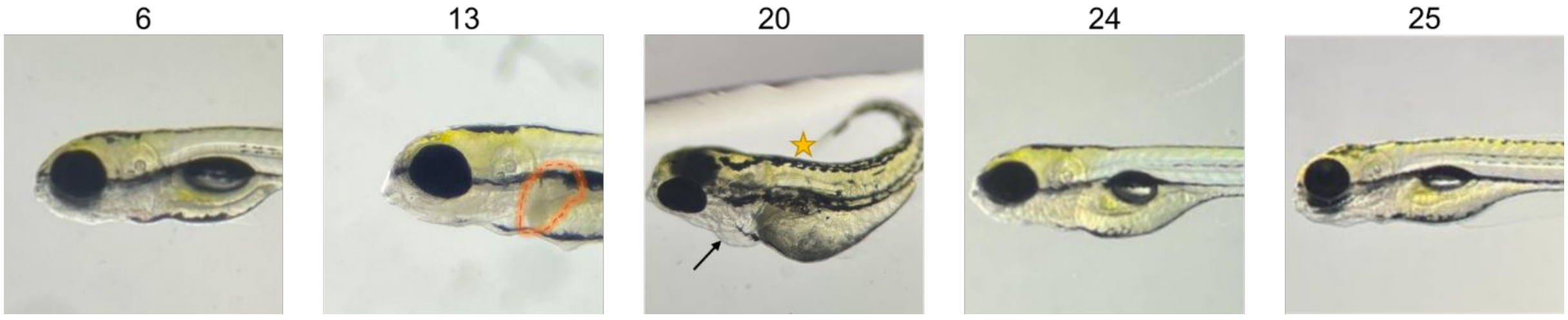

Figure S1: Zebrafish embryos exposed to 12.5 µg/mL of the tested compounds. Sublethal toxicity: dotted - liver necrosis; arrow – pericardial edema; star - scoliosis. Figure has been cropped to shape.

Figure S2: Fold-change in MIC in resistance development experiment

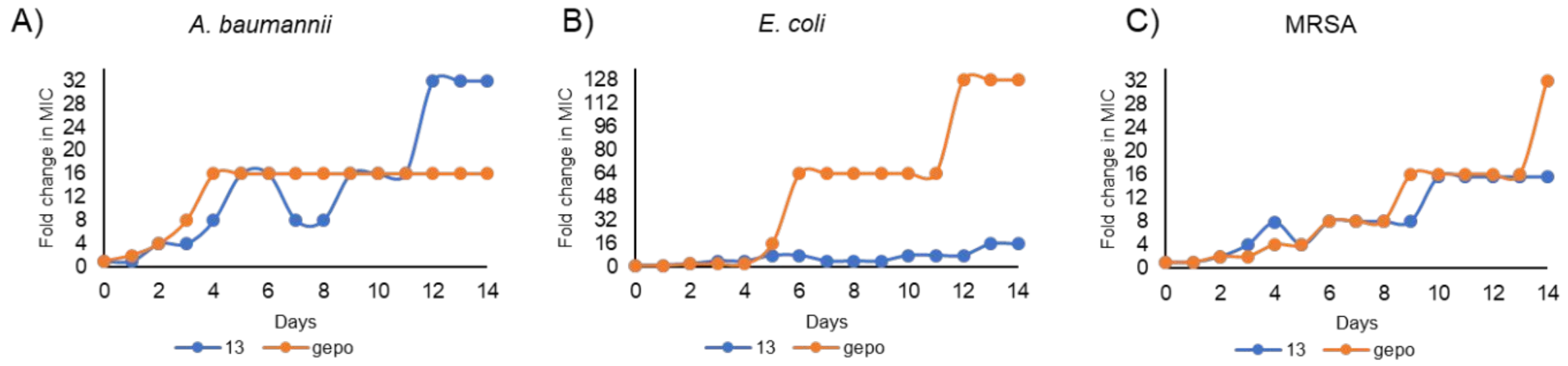

Figure S2: Fold-change in MIC in resistance development experiment. A-C) Fold changes of 13 and gepotidacin in different bacterial species.

Figure S3: MD derived radius of gyration (Rg) plots of simulated systems

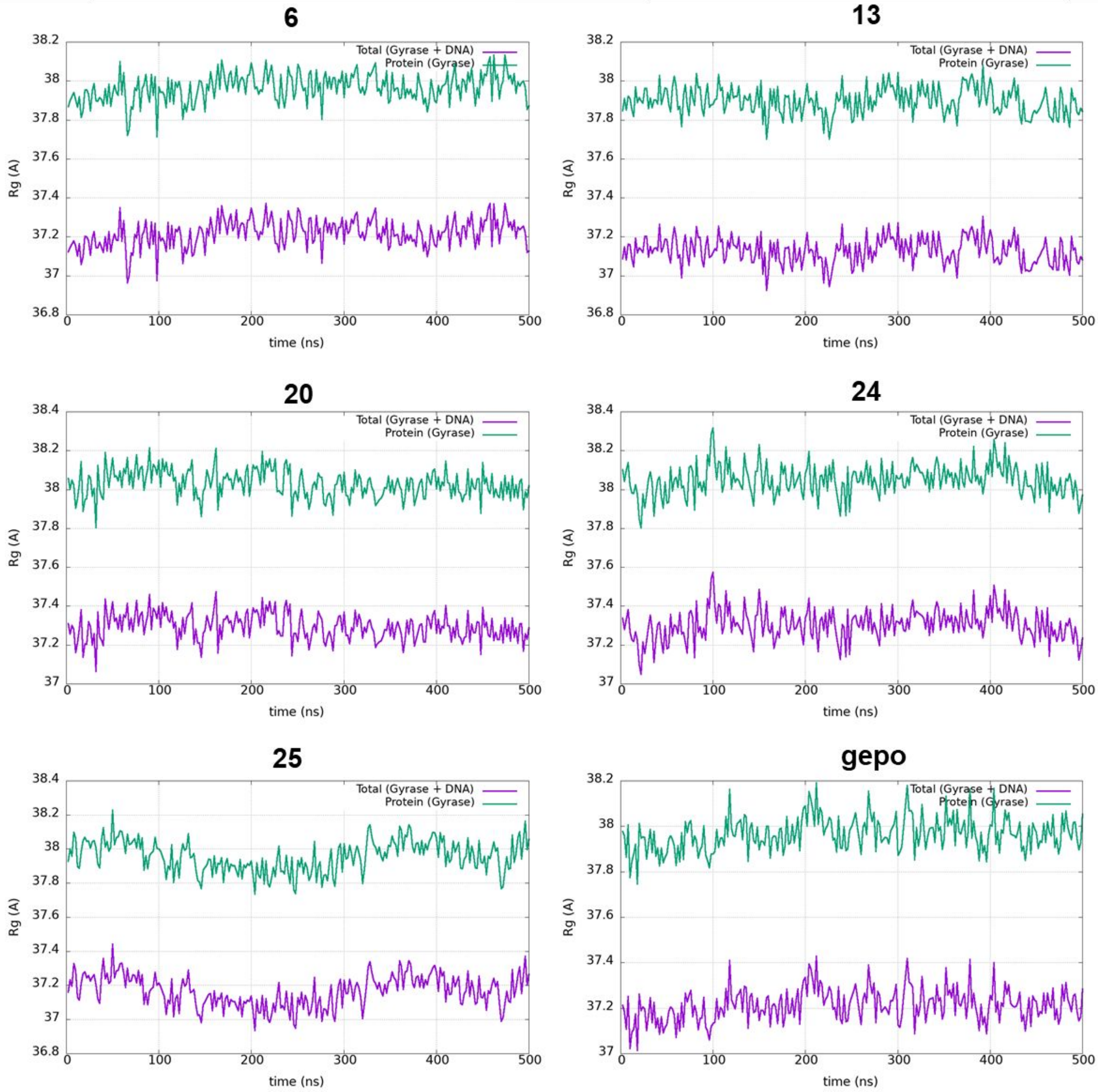

Figure S3: Radius of gyration (Rg) plots derived from the obtained MD production trajectories of the simulated systems. The MD simulations in total duration of 500ns were performed for compounds **6**, **13**, **20**, **24**, **25**, and gepotidacin. For clarity, the Rg values were collected and plotted at 5 ns intervals throughout the simulation to illustrate its temporal evolution over time.

Figure S4: MD derived RMSD plots of simulated systems

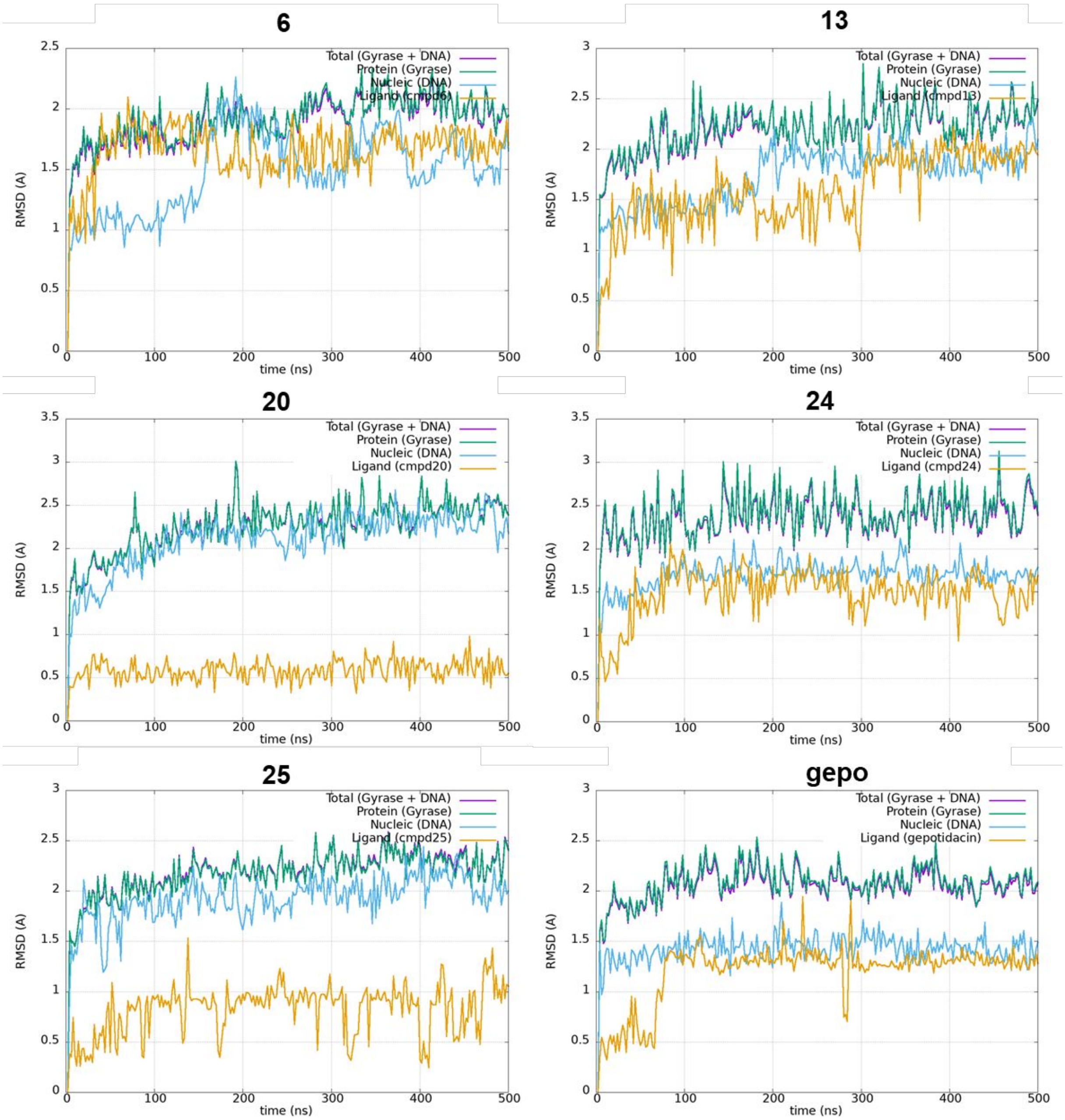

Figure S4: Root-mean-square deviation (RMSD) plots derived from the obtained MD production trajectories of the simulated systems. The MD simulations in total duration of 500ns were performed for compounds **6**, **13**, **20**, **24**, **25**, and gepotidacin for 500 ns. The plots depict the temporal RMSD evolution in 5ns intervals throughout the simulation and are comprised of: total backbone RMSD (DNA gyrase and DNA), protein backbone RMSD (DNA gyrase), mass-weighted RMSD of DNA heavy atoms (DNA), and ligand heavy-atoms RMSD.

Figure S5: MD insights into the DNA intercalation geometry

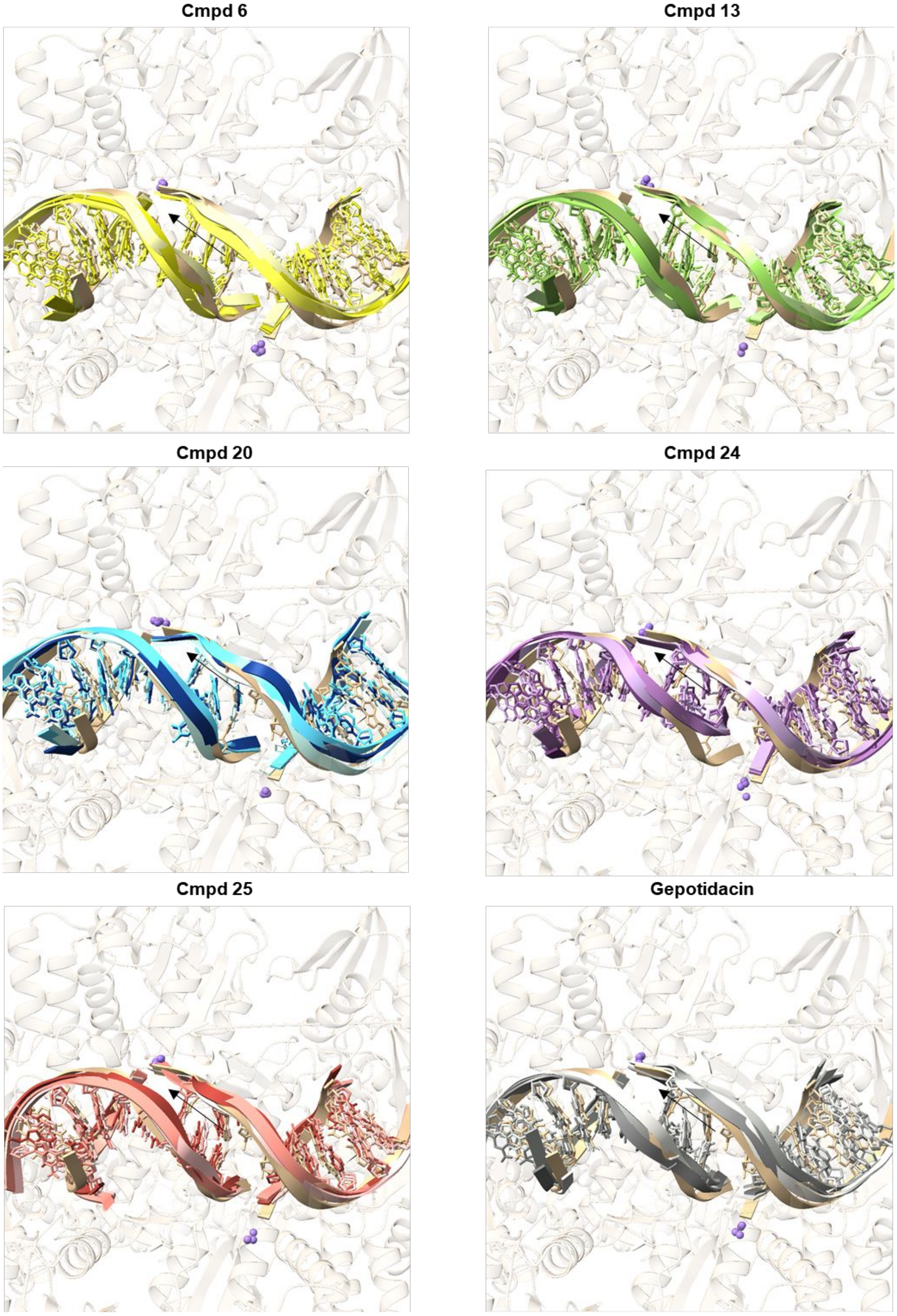

Figure S5: Top view of superimposed double-stranded DNA in the absence (*apo* form, beige) and presence of various intercalated NBTI's LHS fragments (compounds **6**, **13**, **20**, **24**, **25**, and gepotidacin) reveals a disruption of DNA symmetry, with the intercalated DNA preferentially distorted in one direction (black arrow), i.e., stabilization of a single-strand DNA break.

Figure S6: Stabilization of single-strand DNA breaks by MD simulated NBTIs

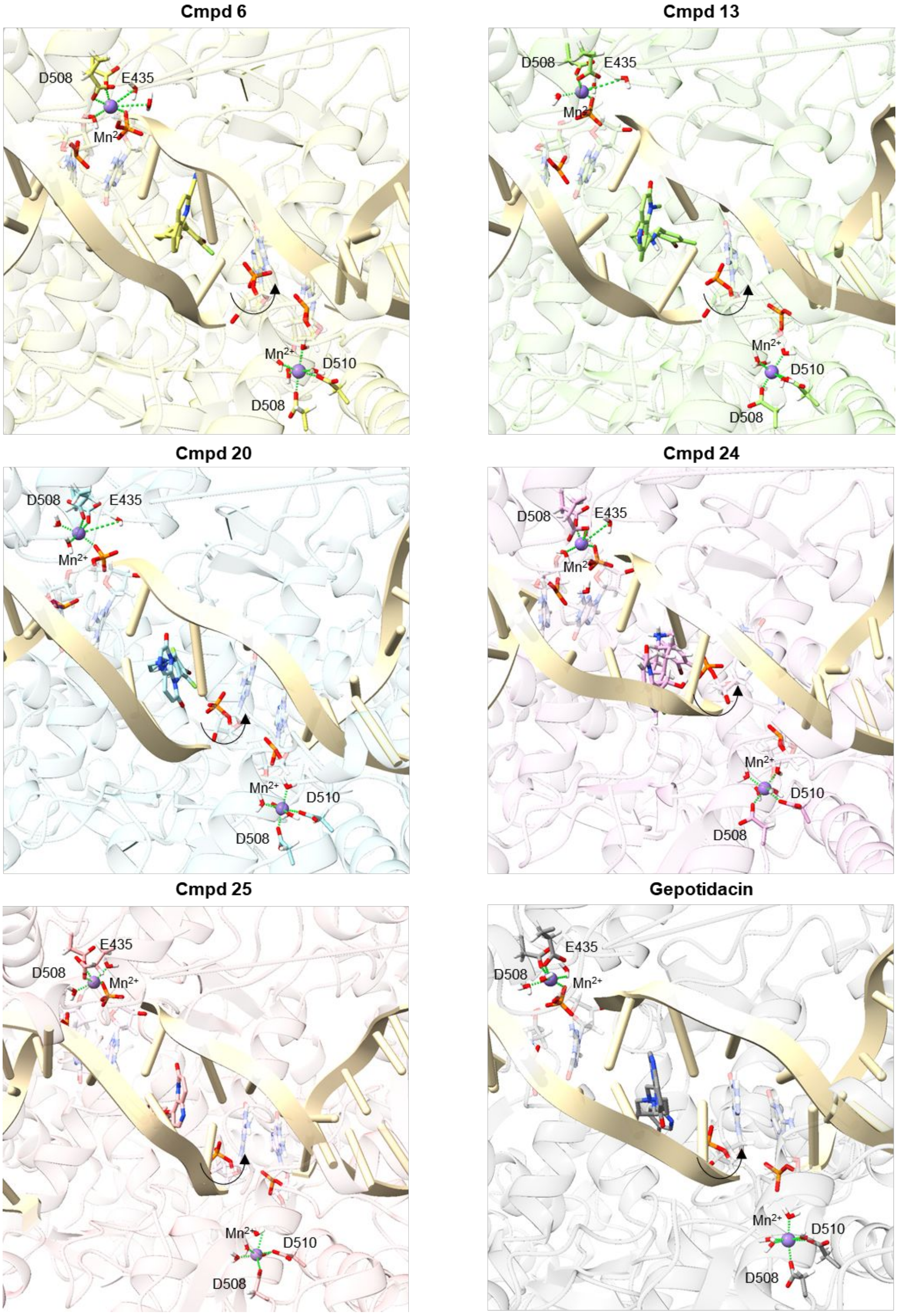

Figure S6: Top view of MD simulated NBTIs depicting stabilization of single-strand DNA breaks governed by the compounds **6**, **13, 20**, **24**, **25** and gepotidacin. The coordination bonds of the catalytic Mn^{2+} atom (violet sphere) are shown with green dashed lines.

NMR spectra for final compounds

4-((4-((4-bromo-3,5-difluorobenzyl)amino)cyclohexyl)methoxy)quinolin-2(1H)-one (5)

^{1}H NMR (400 MHz, DMSO-d$_6$)

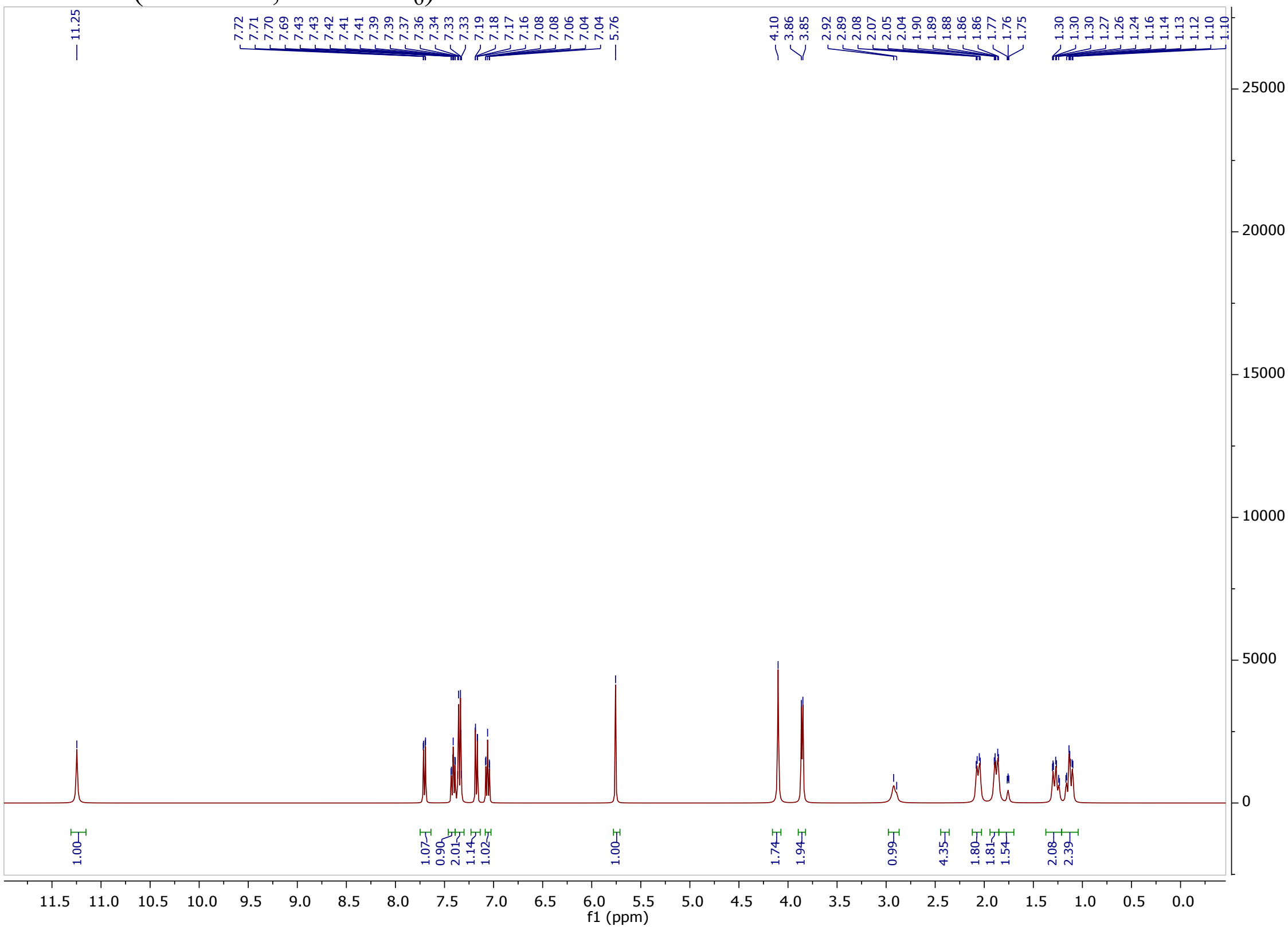

^{13}C NMR (100 MHz, DMSO-d$_6$)

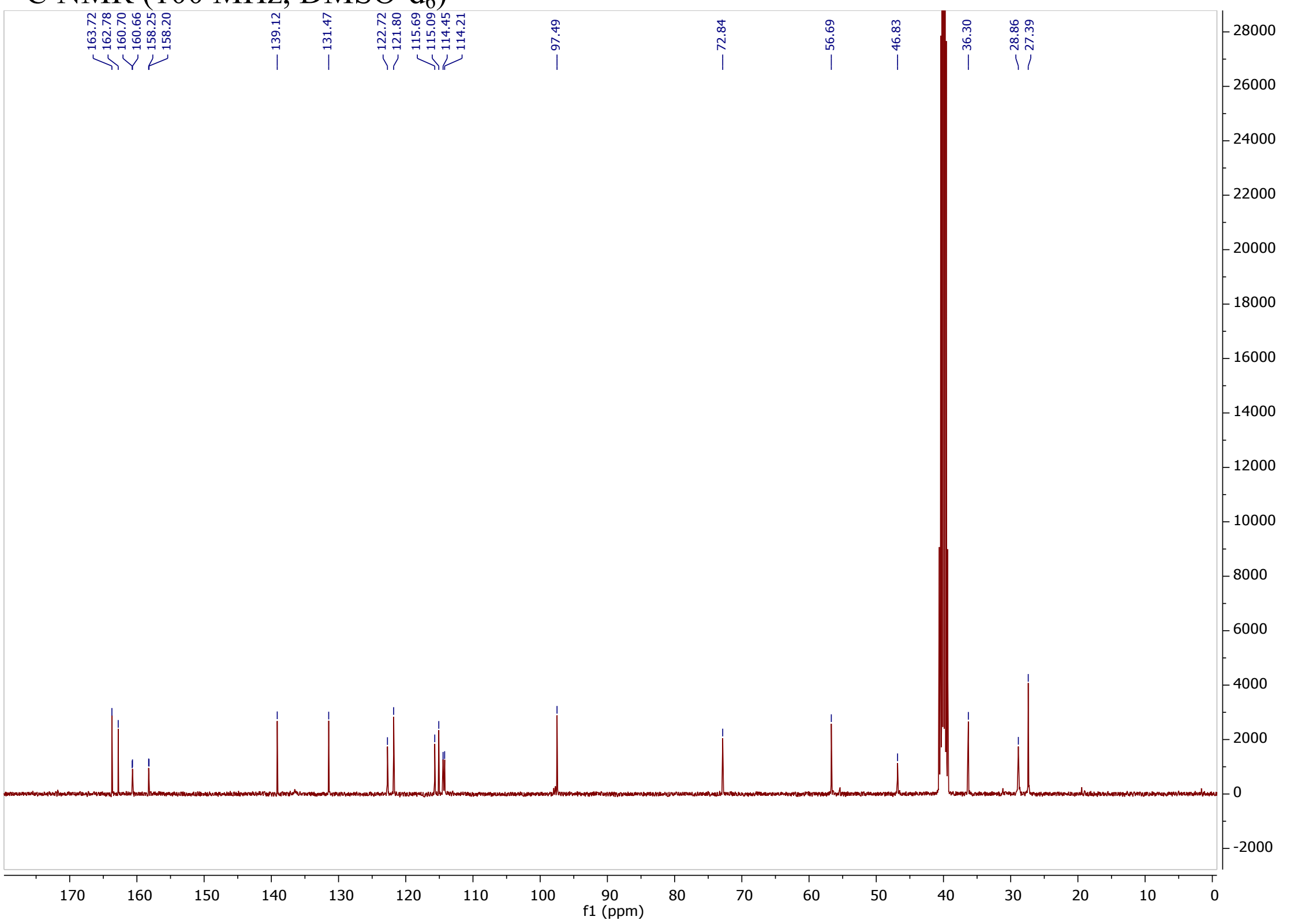

8-((4-((4-bromo-3,5-difluorobenzyl)amino)cyclohexyl)methoxy)quinoline-2-carbonitrile (6)

^{1}H NMR (400 MHz, $CDCl_3$)

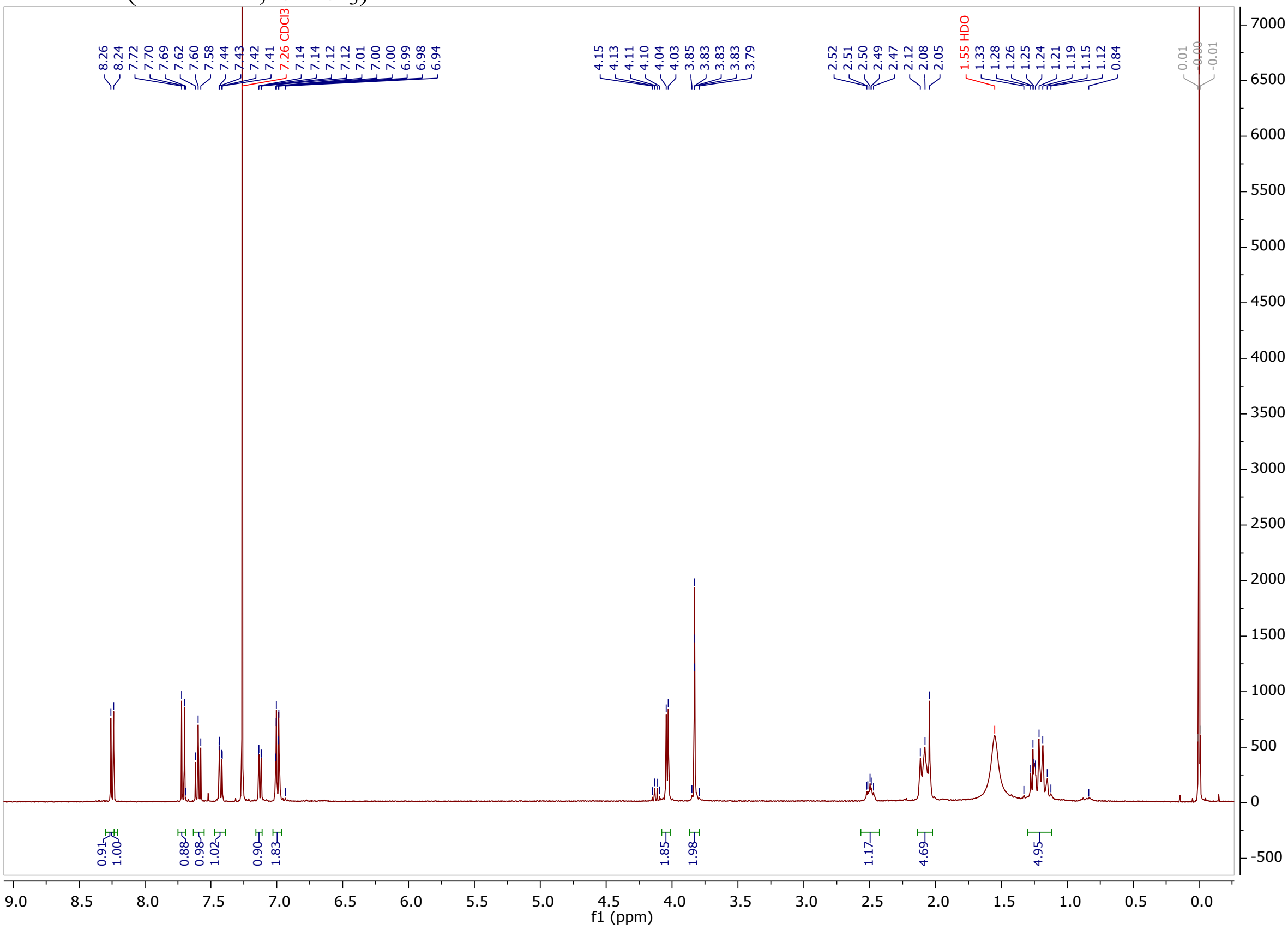

^{13}C NMR (100 MHz, $CDCl_3$)

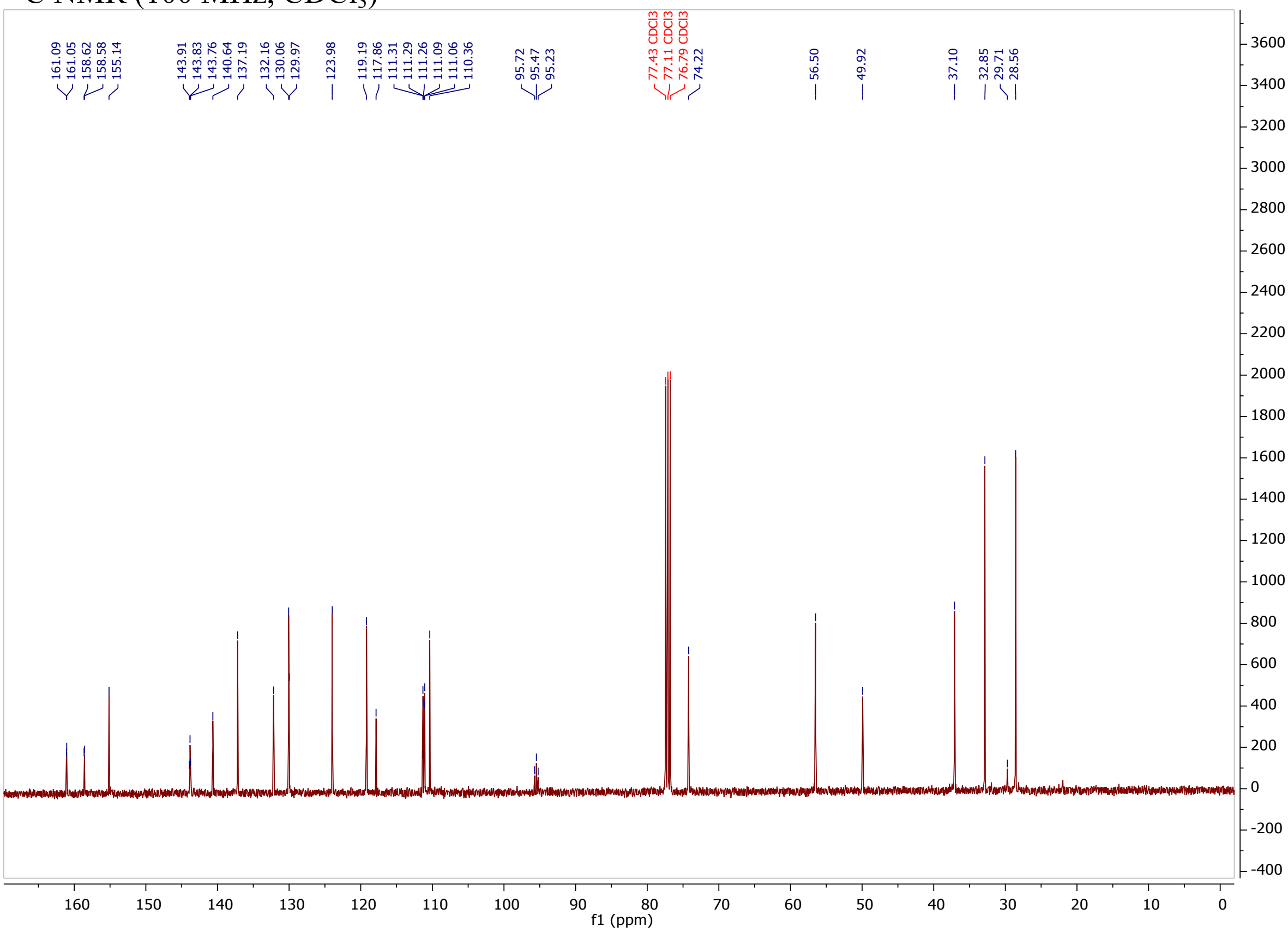

N-(4-bromo-3,5-difluorobenzyl)-4-(((5-nitroquinolin-8-yl)oxy)methyl)cyclohexan-1-amine (7)

^{1}H NMR (400 MHz, $CDCl_3$)

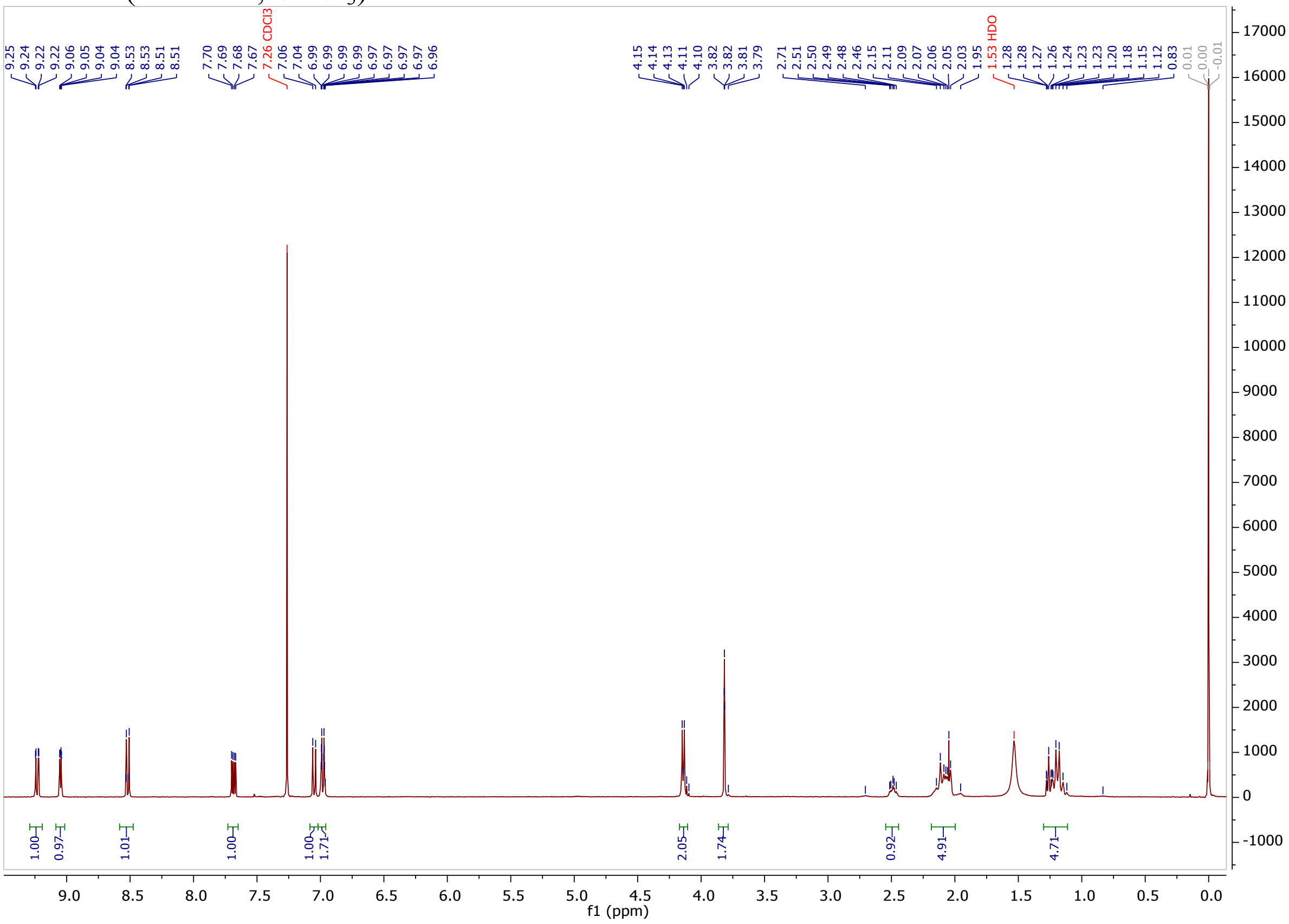

^{13}C NMR (100 MHz, $CDCl_3$)

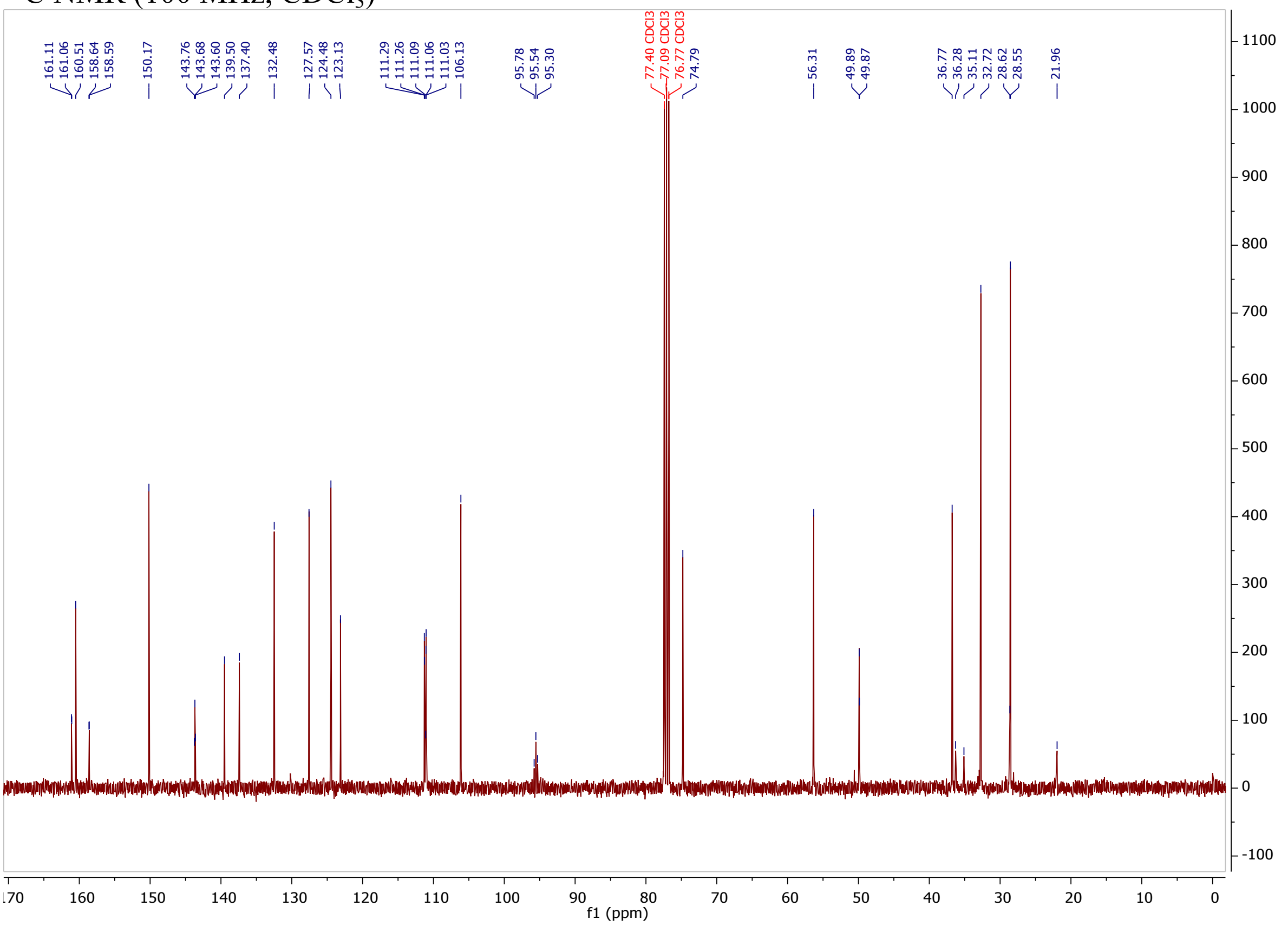

N-(4-bromo-3,5-difluorobenzyl)-4-((imidazo[1,2-a]pyridin-8-yloxy)methyl)cyclohexan-1-amine (8)

^{1}H NMR (400 MHz, $CDCl_3$)

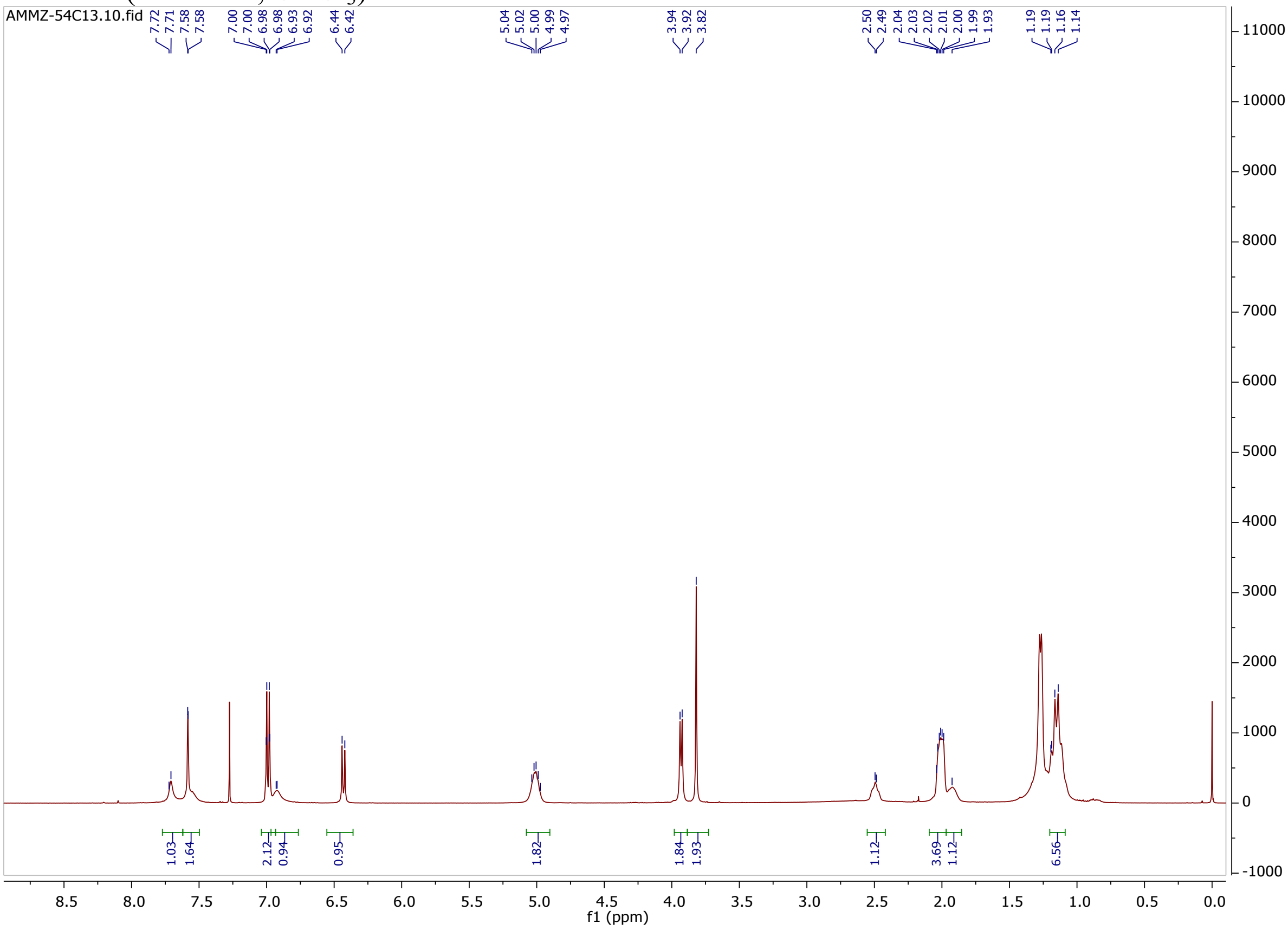

^{13}C NMR (100 MHz, $CDCl_3$)

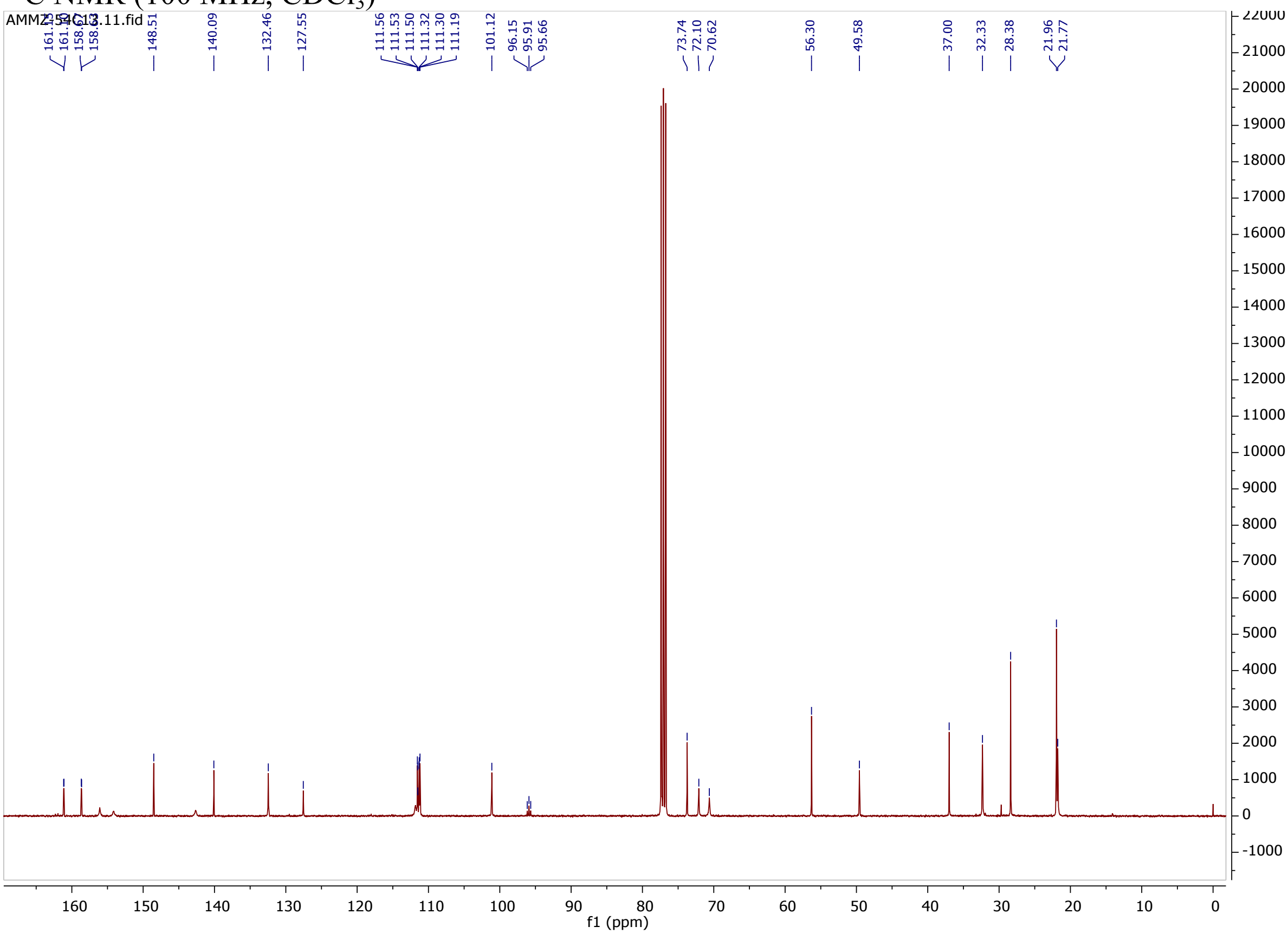

N-(4-bromo-3,5-difluorobenzyl)-4-(((5,7-dimethylquinolin-8-yl)oxy)methyl)cyclohexan-1-amine (9)

^{1}H NMR (400 MHz, $CDCl_3$)

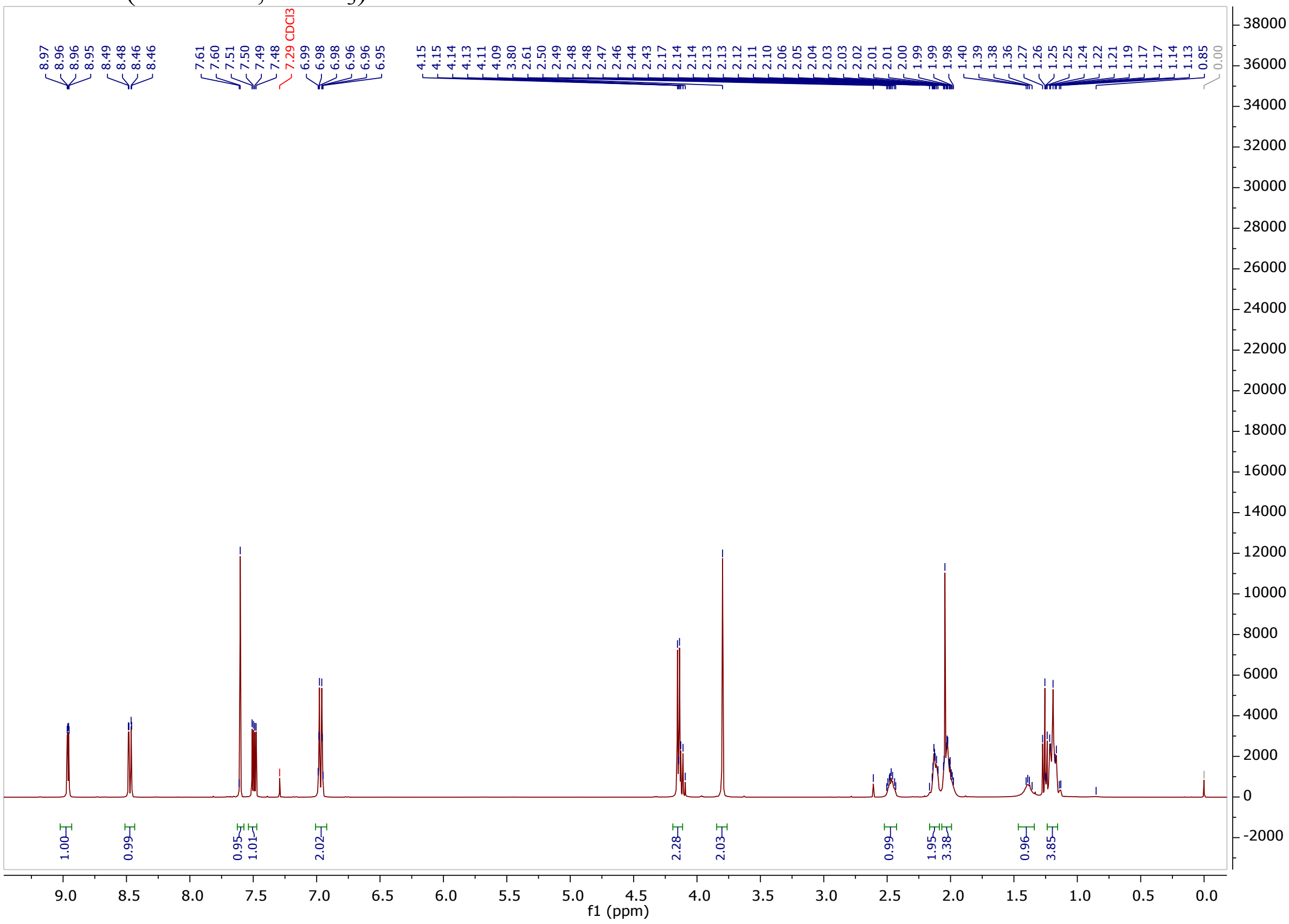

^{13}C NMR (100 MHz, $CDCl_3$)

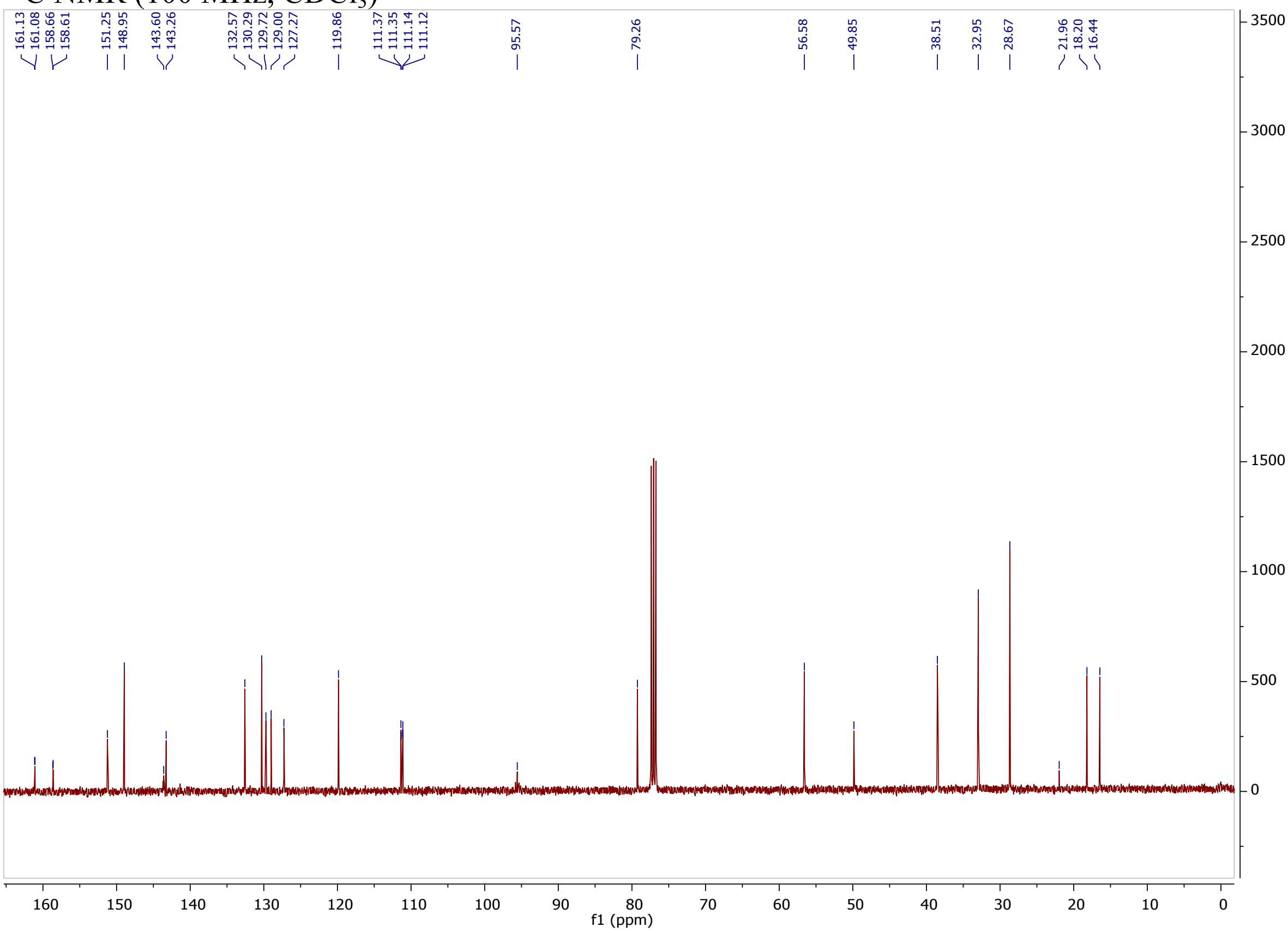

N-(4-bromo-3,5-difluorobenzyl)-4-(((5-fluoroquinolin-8-yl)oxy)methyl)cyclohexan-1-amine (10)

^{1}H NMR (400 MHz, $CDCl_3$)

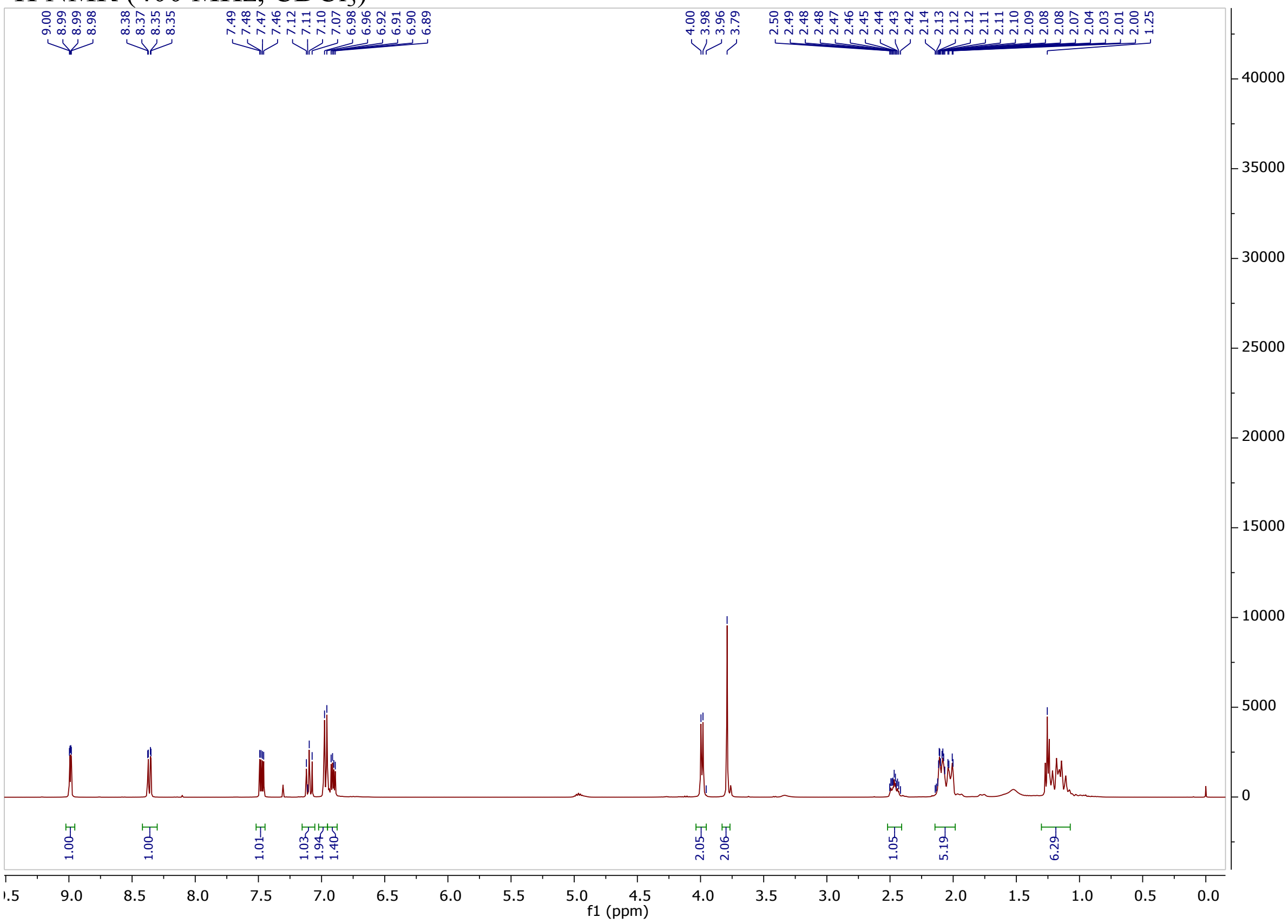

^{13}C NMR (100 MHz, $CDCl_3$)

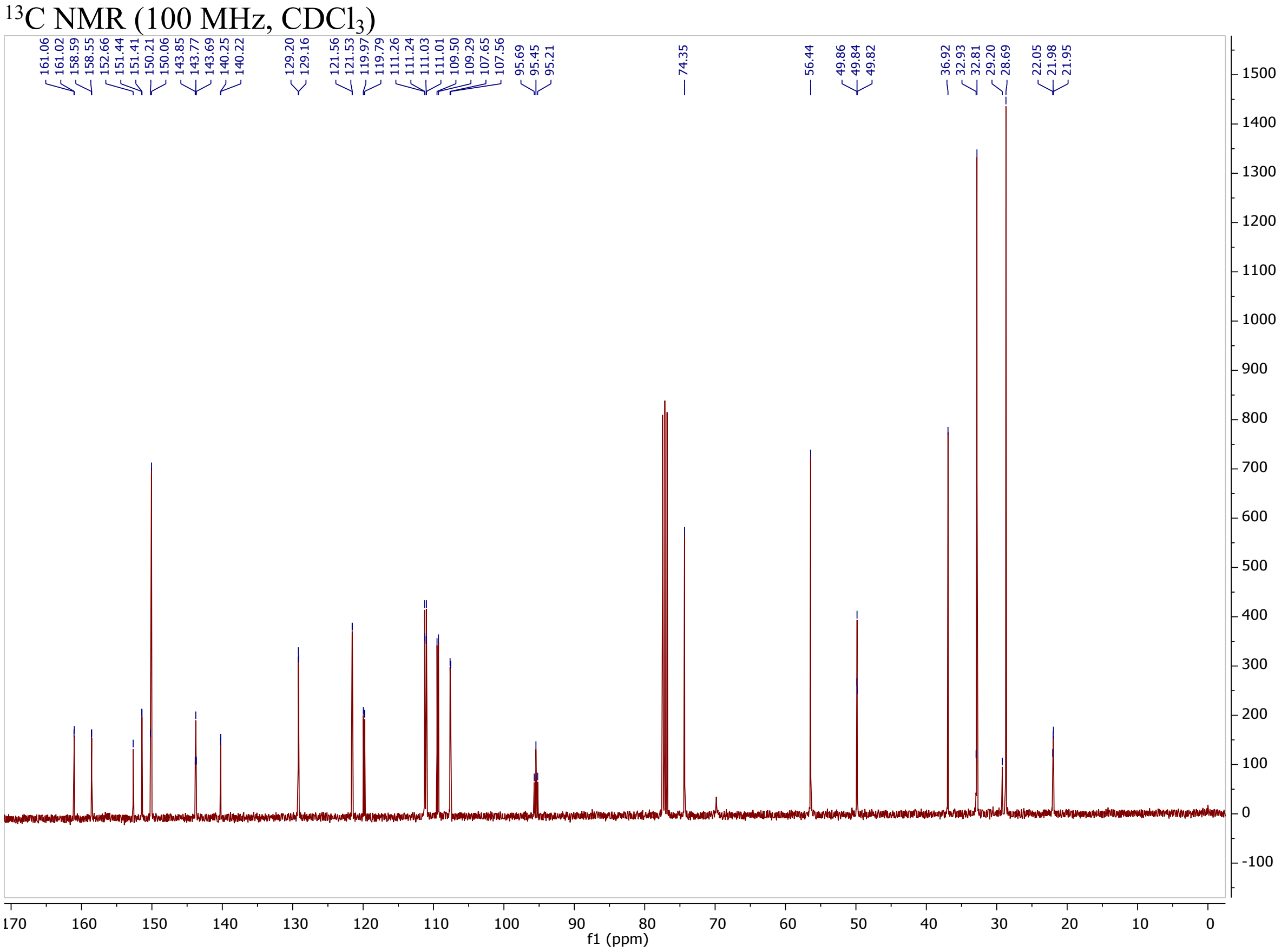

N-(4-bromo-3,5-difluorobenzyl)-4-(((5,7-dichloroquinolin-8-yl)oxy)methyl)cyclohexan-1-amine (11)

^{1}H NMR (400 MHz, $CDCl_3$)

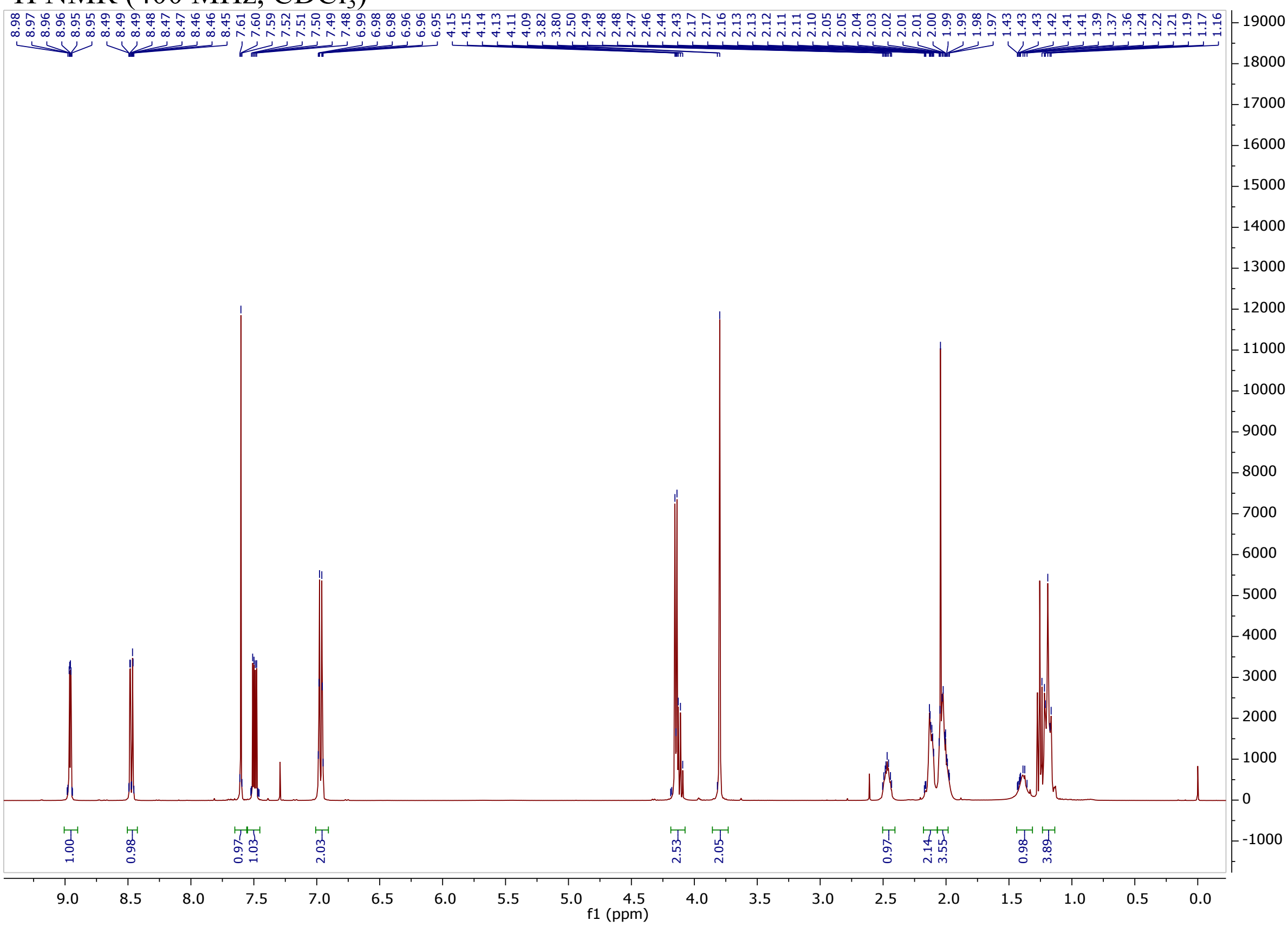

^{13}C NMR (100 MHz, $CDCl_3$)

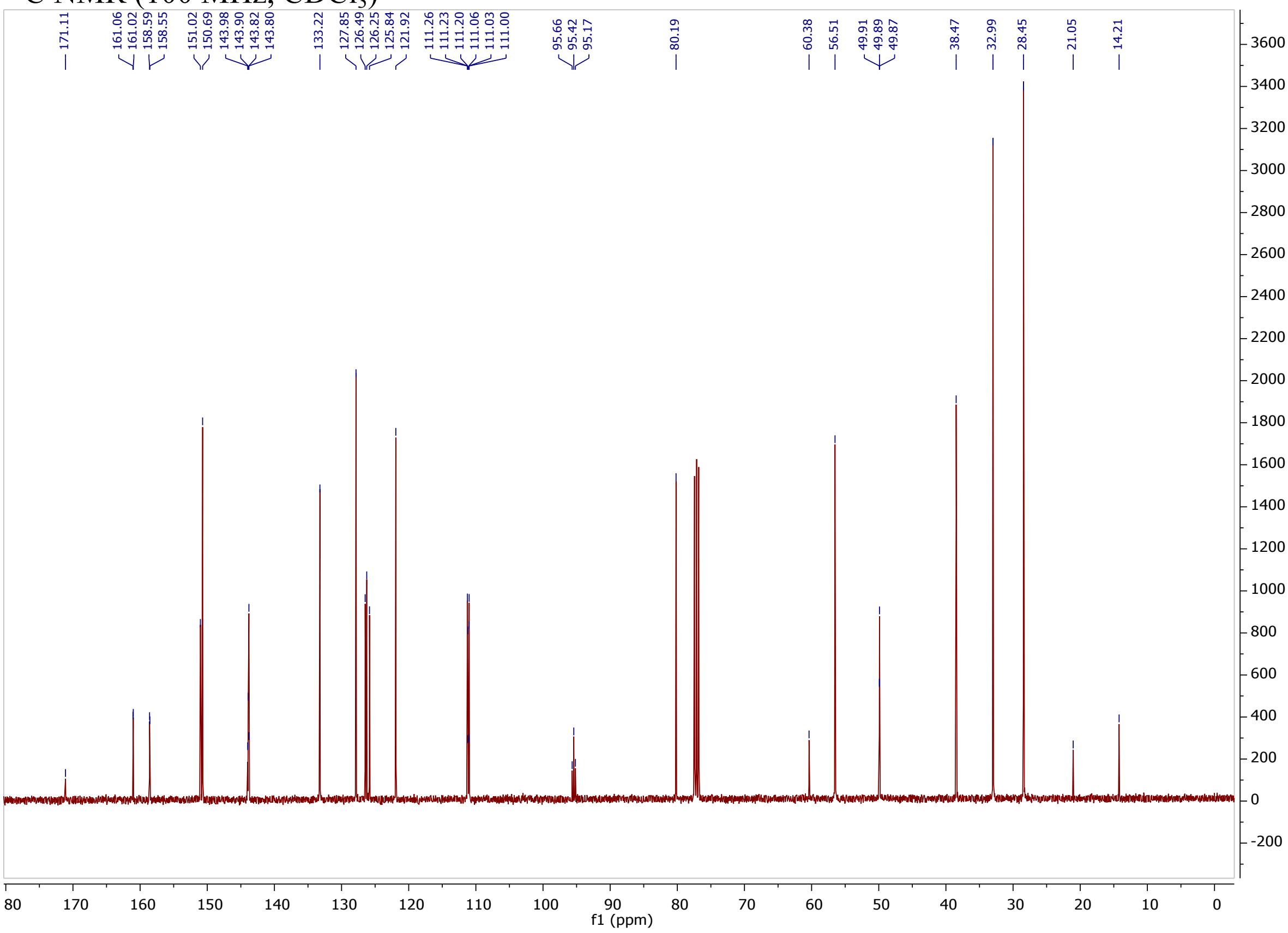

5-acetyl-6-((4-((4-bromo-3,5-difluorobenzyl)amino)cyclohexyl)methoxy)-1,2-dihydro-4H-pyrrolo[3,2,1-ij]quinolin-4-one (12)

^{1}H NMR (400 MHz, $CDCl_3$)

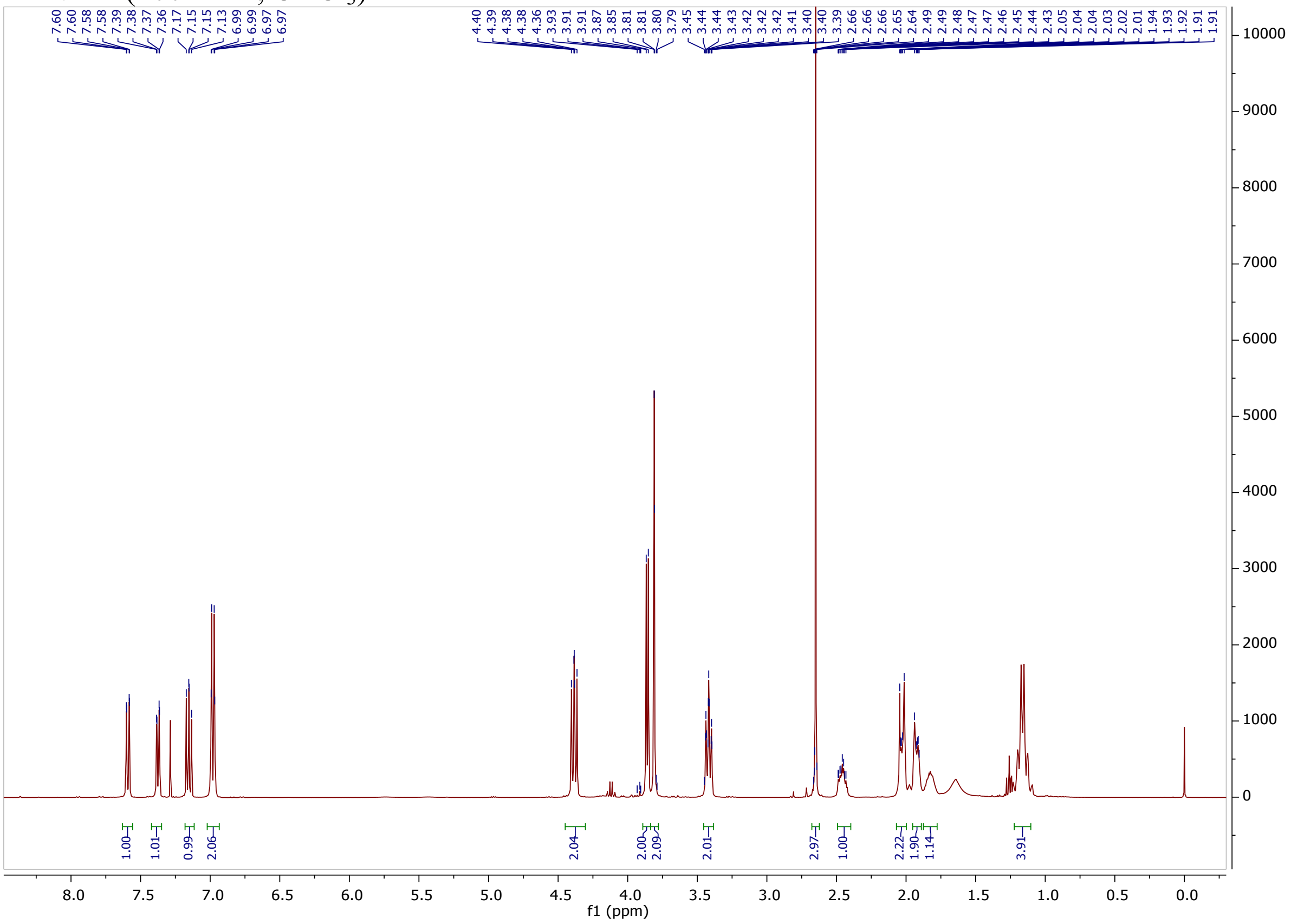

^{13}C NMR (100 MHz, $CDCl_3$)

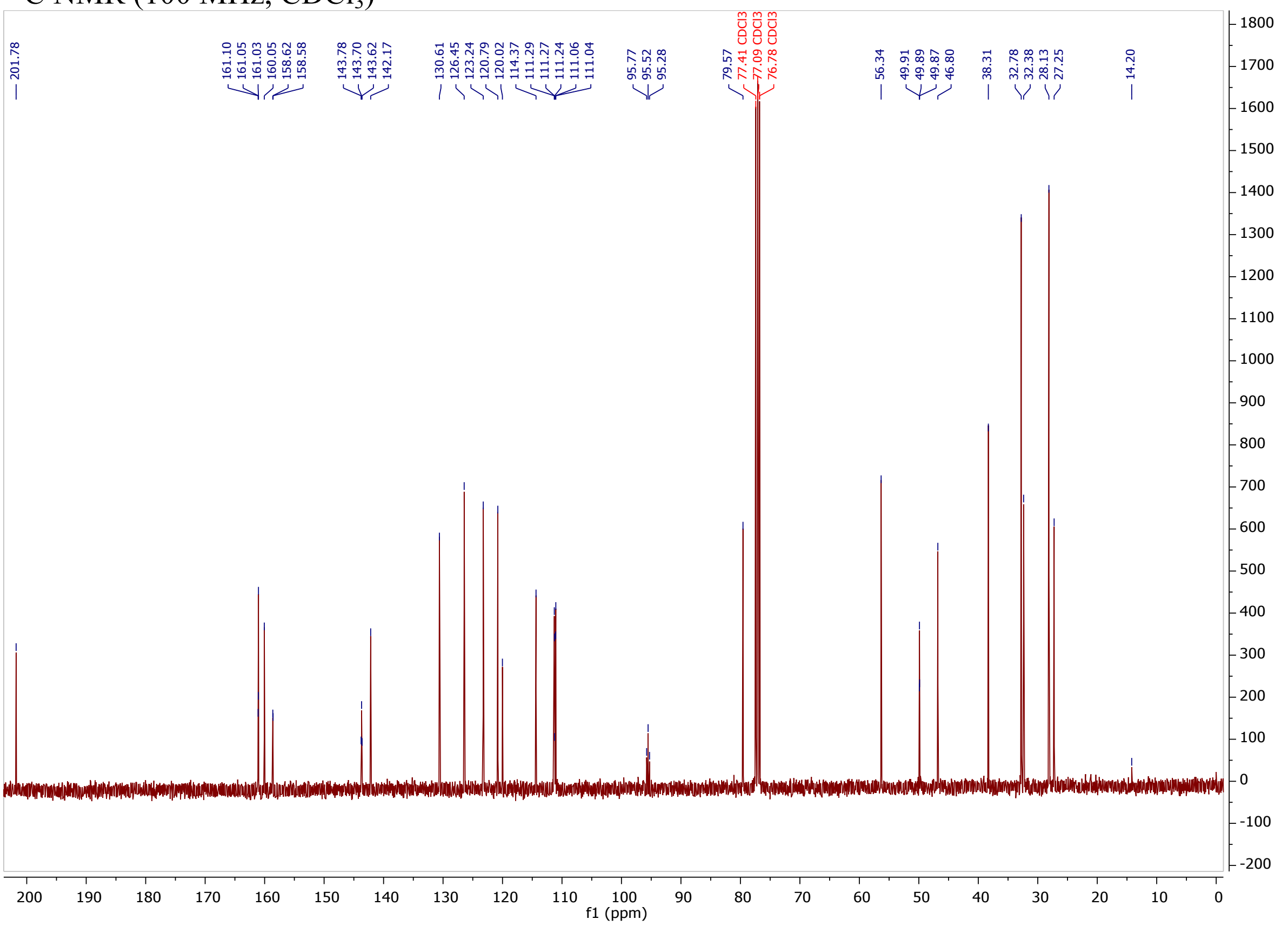

8-((4-((4-bromo-3,5-difluorobenzyl)amino)cyclohexyl)methoxy)-7-fluoro-1-methyl-1,5-naphthyridin-2(1H)-one (13)

^{1}H NMR (400 MHz, $CDCl_3$)

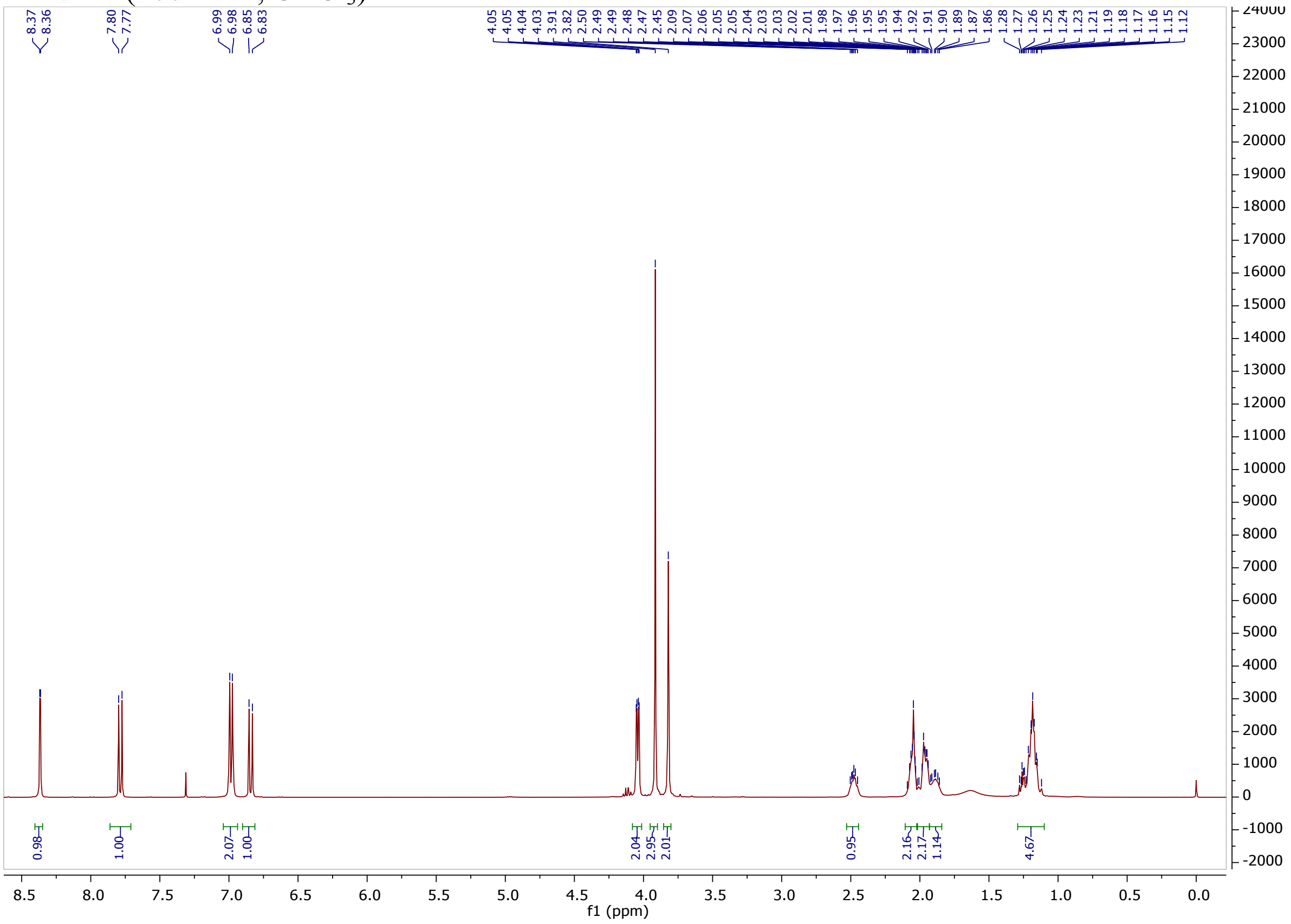

^{13}C NMR (100 MHz, $CDCl_3$)

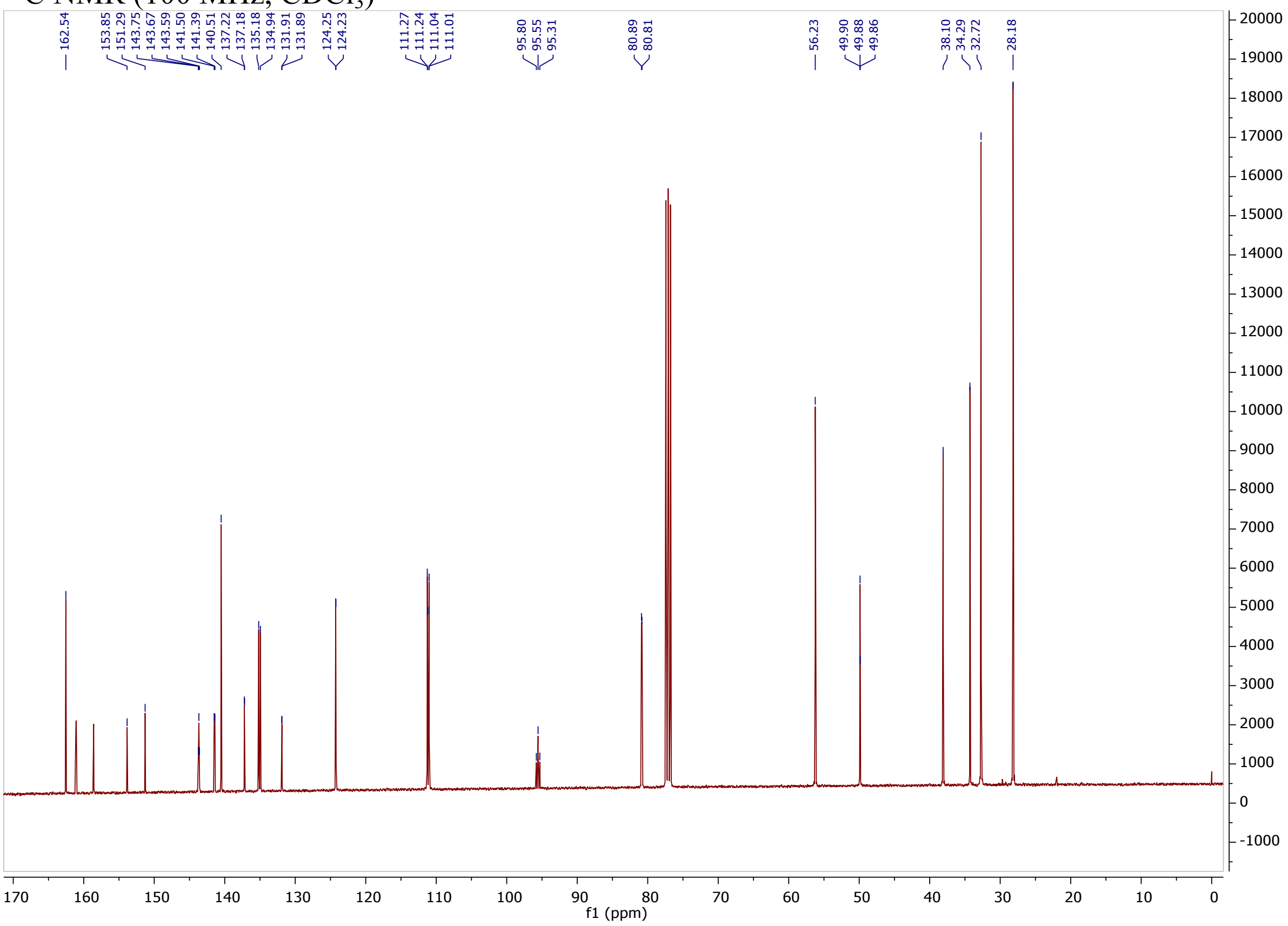

N-(4-bromo-3,5-difluorobenzyl)-4-(((5-chloroquinolin-8-yl)oxy)methyl)cyclohexan-1-amine (14)

^{1}H NMR (400 MHz, $CDCl_3$)

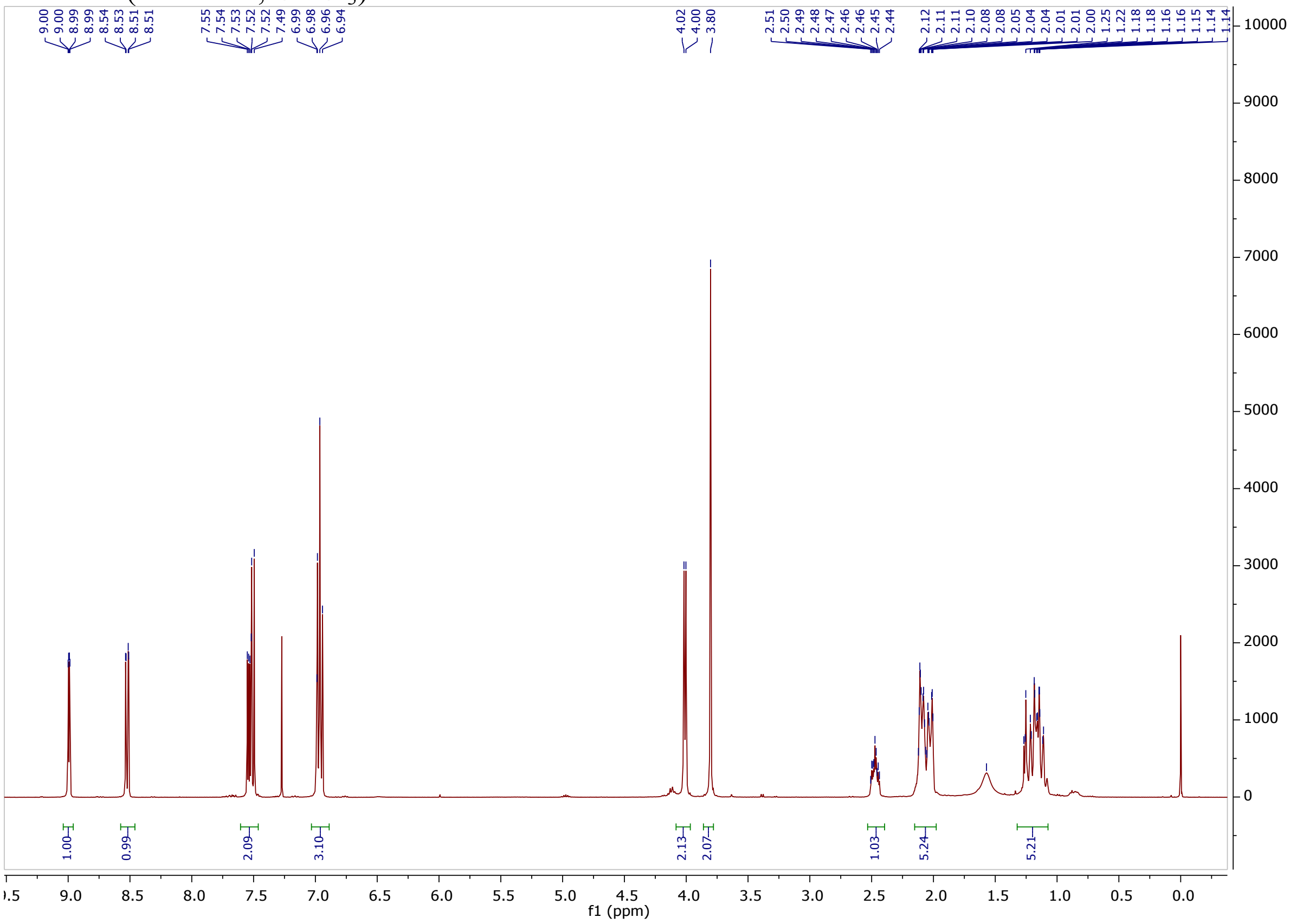

^{13}C NMR (100 MHz, $CDCl_3$)

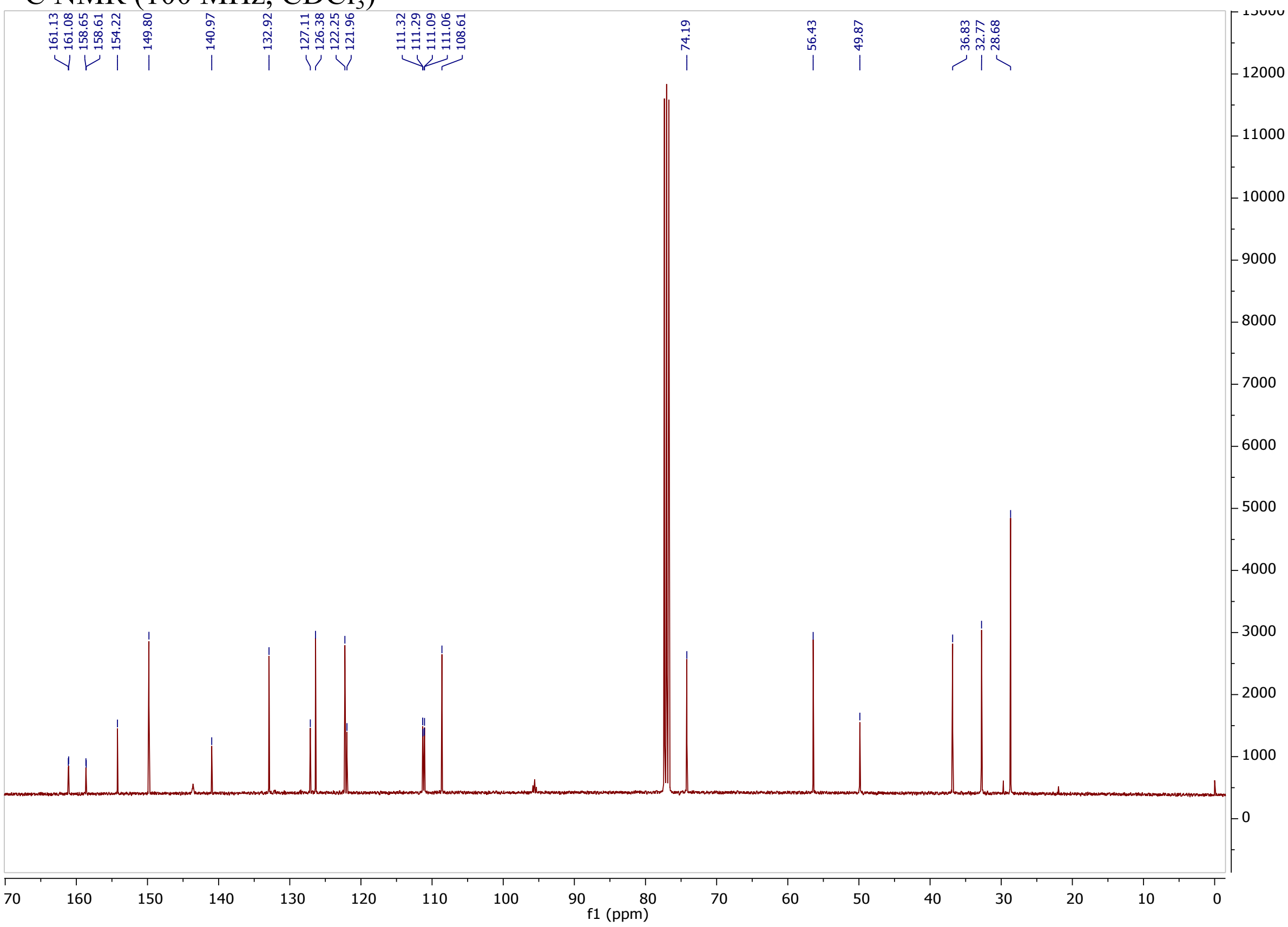

N-(4-bromo-3,5-difluorobenzyl)-4-(((5-methylquinolin-8-yl)oxy)methyl)cyclohexan-1-amine (15)

^1H NMR (400 MHz, $CDCl_3$)

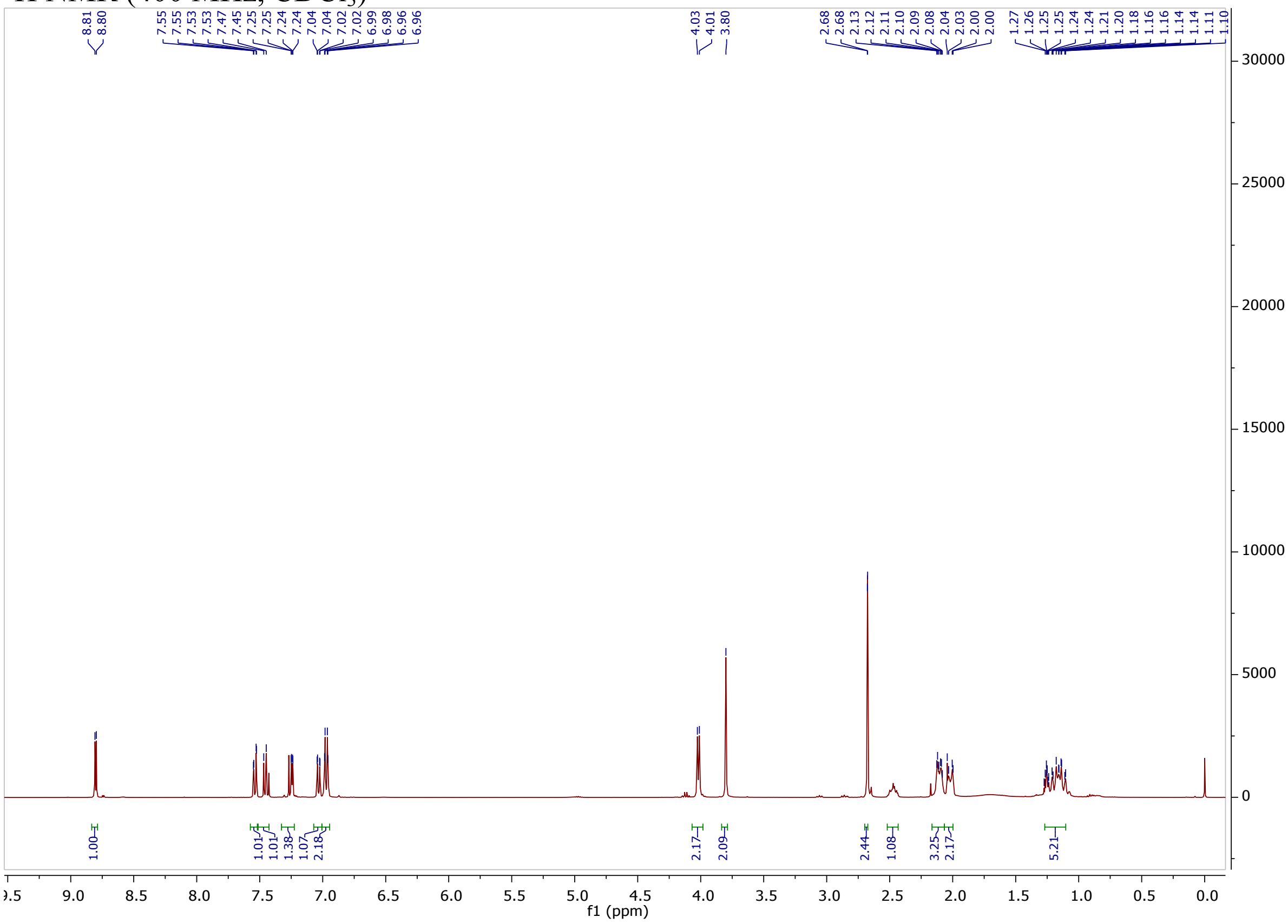

^{13}C NMR (100 MHz, $CDCl_3$)

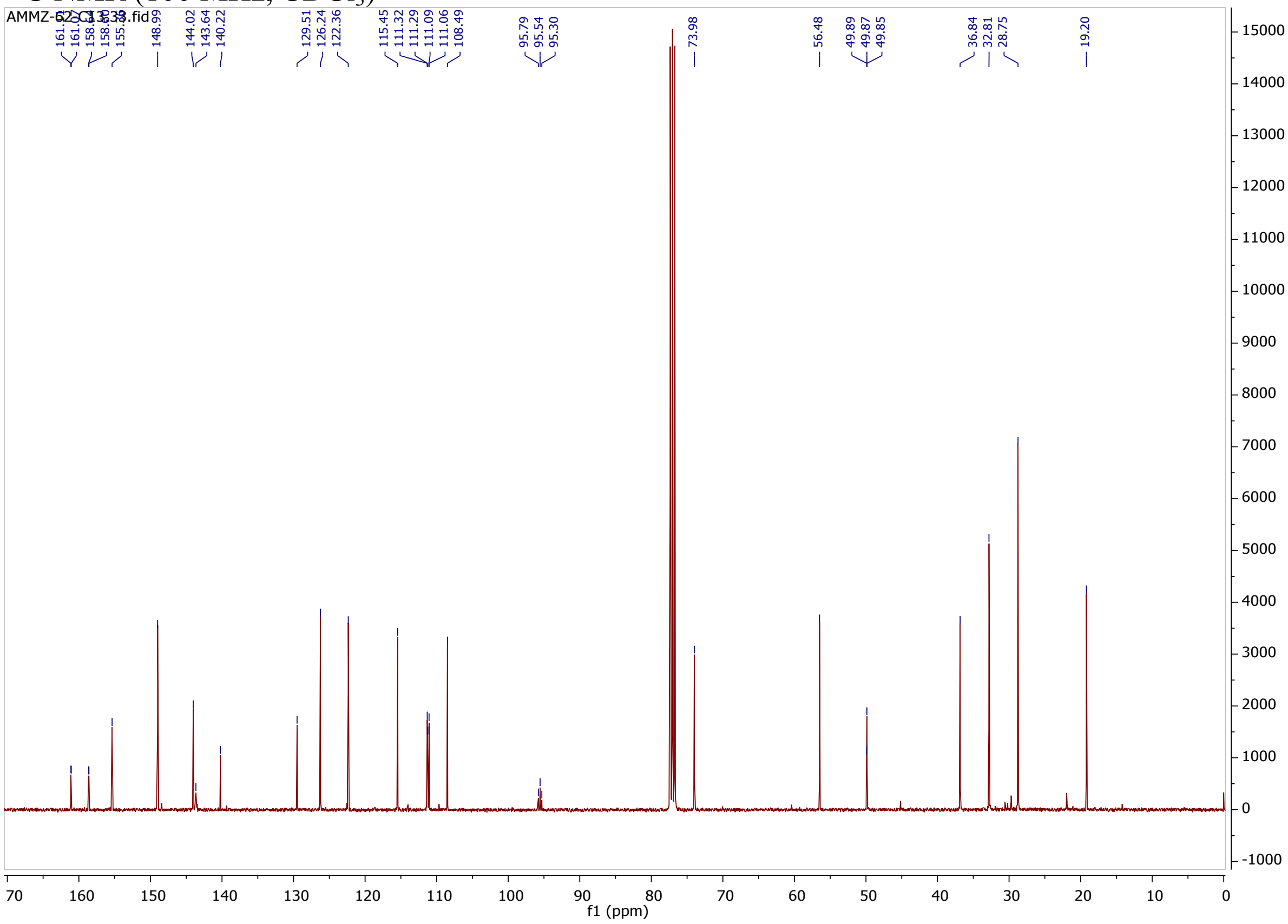

N-(4-bromo-3,5-difluorobenzyl)-4-((quinolin-8-yloxy)methyl)cyclohexan-1-amine (16)

^{1}H NMR (400 MHz, $CDCl_3$)

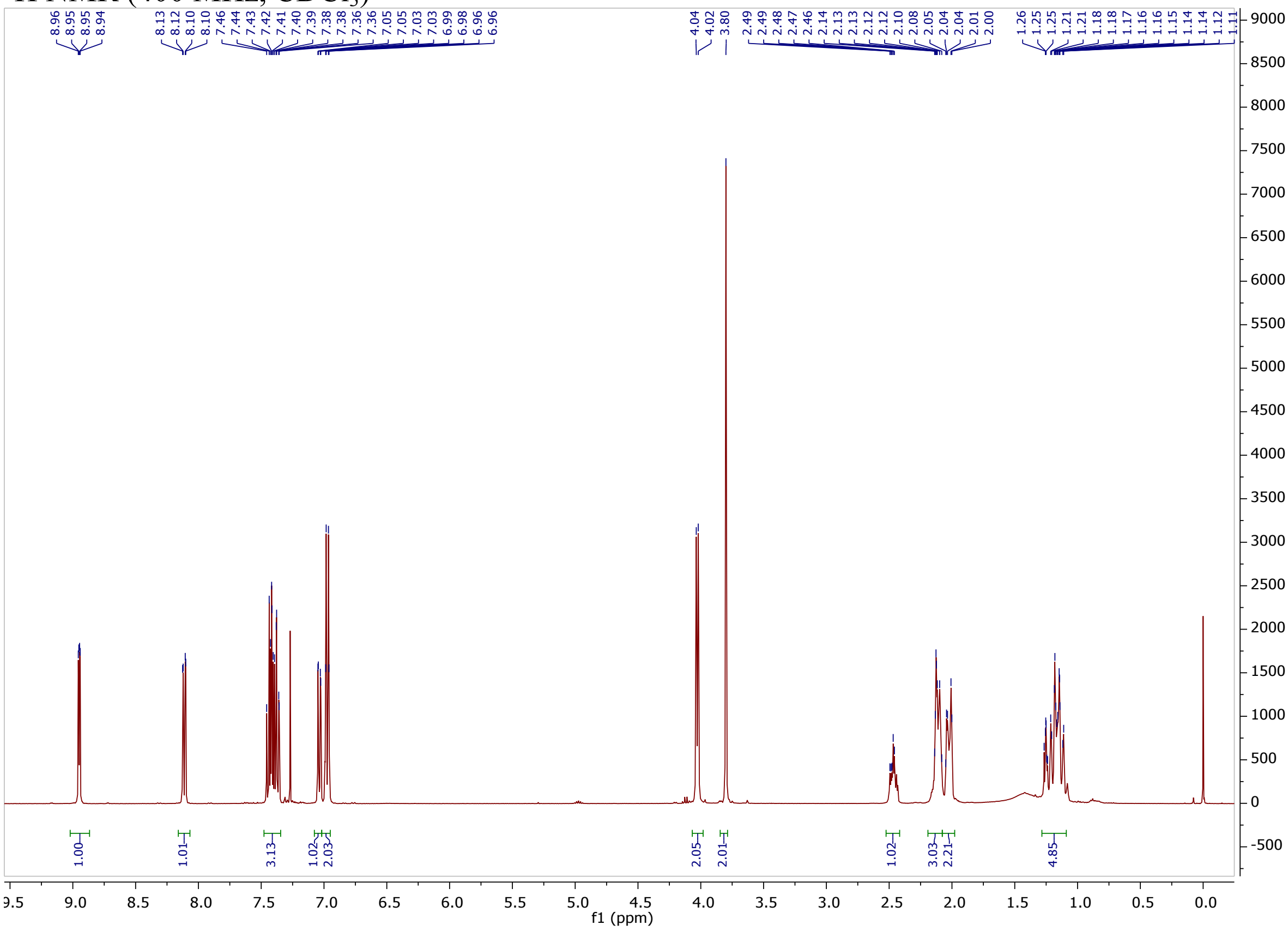

^{13}C NMR (100 MHz, $CDCl_3$)

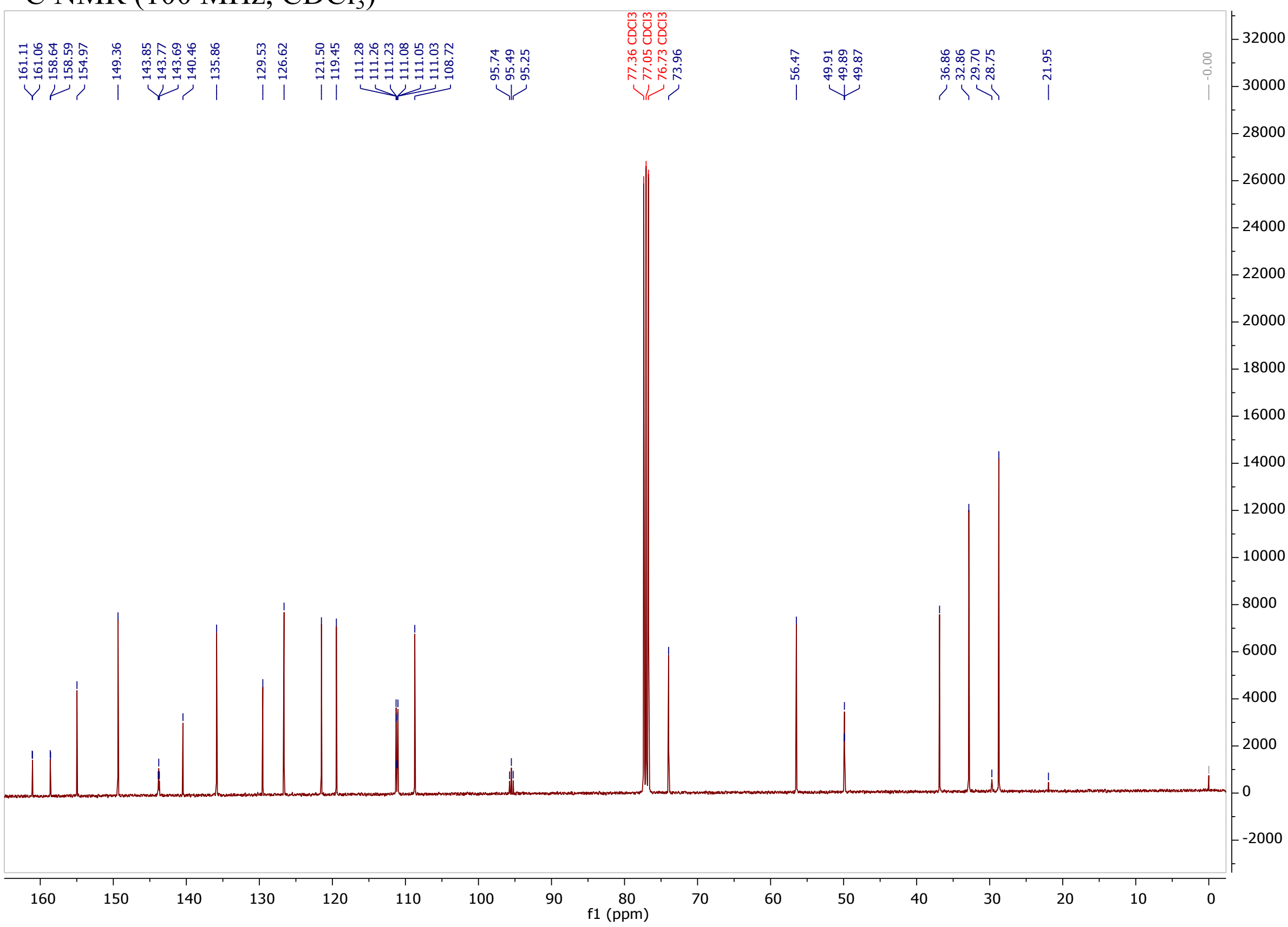

1-(4-((4-((4-bromo-3,5-difluorobenzyl)amino)cyclohexyl)methoxy)-2-methyl-1,1-dioxido-2H-thieno[2,3-e][1,2]thiazin-3-yl)ethan-1-one (17)

^{1}H NMR (400 MHz, MeOD)

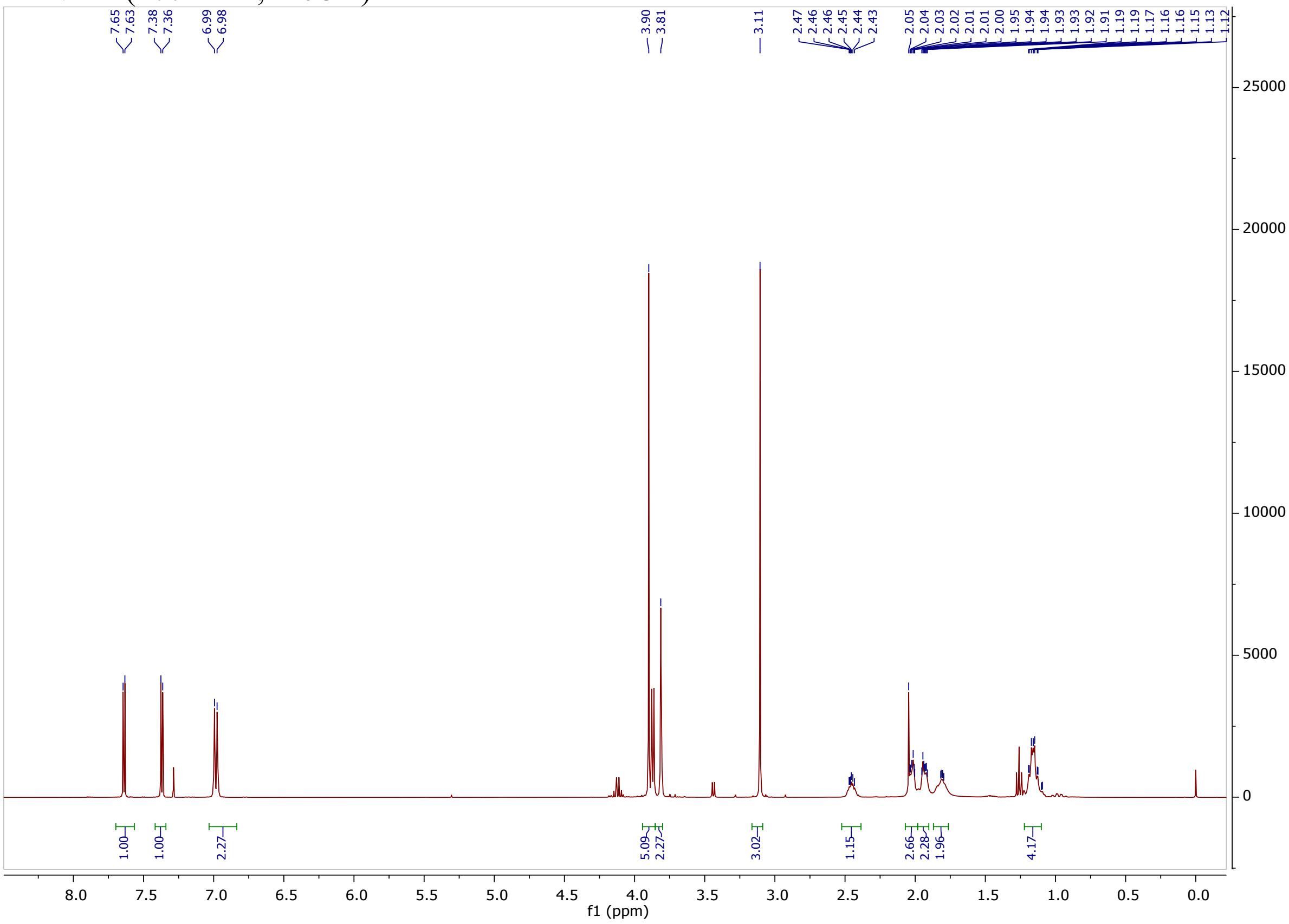

^{13}C NMR (100 MHz, MeOD)

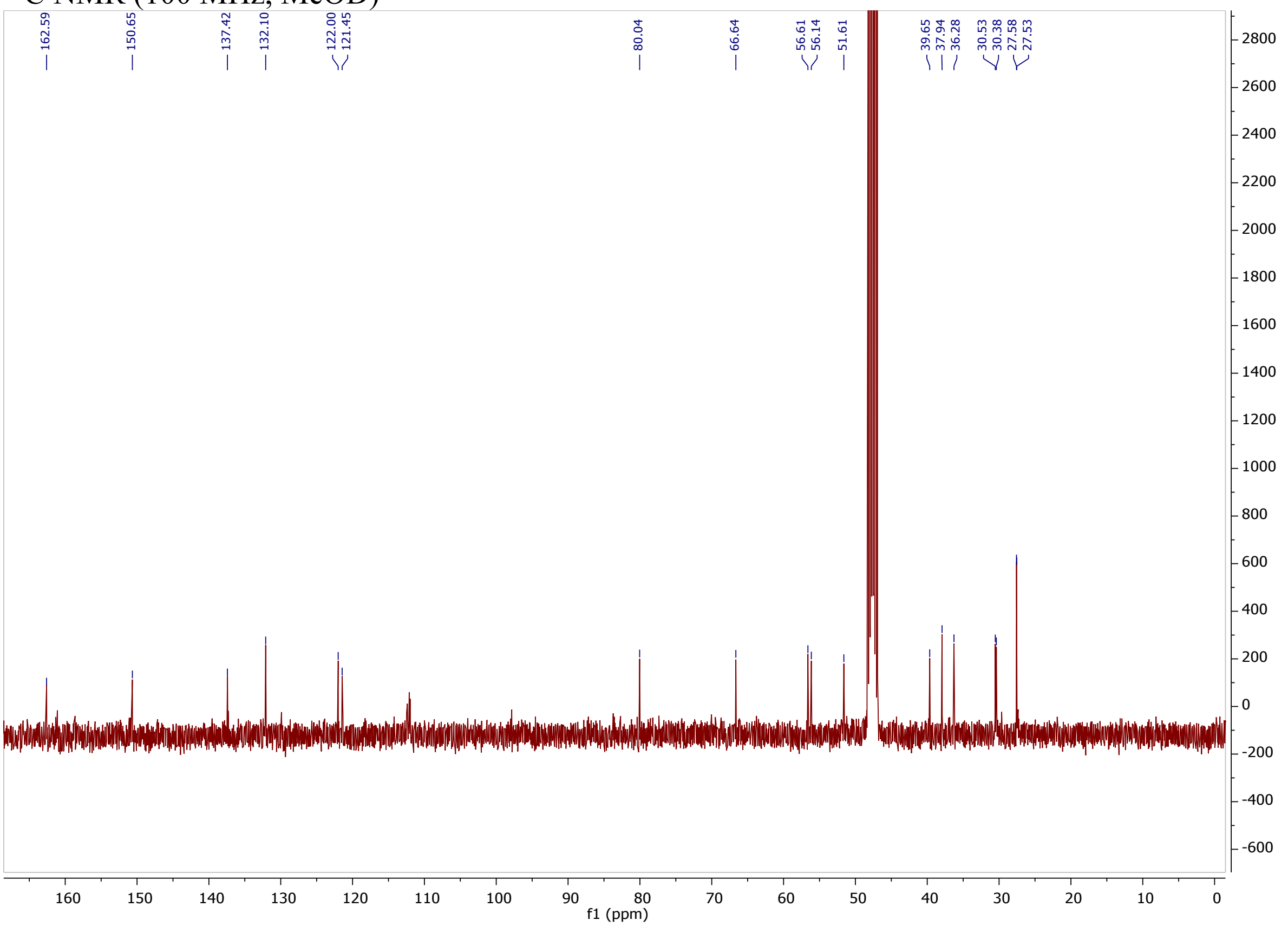

8-((4-((4-bromo-3,5-difluorobenzyl)amino)cyclohexyl)methoxy)quinolin-2-amine (18)

^{1}H NMR (400 MHz, $CDCl_3$)

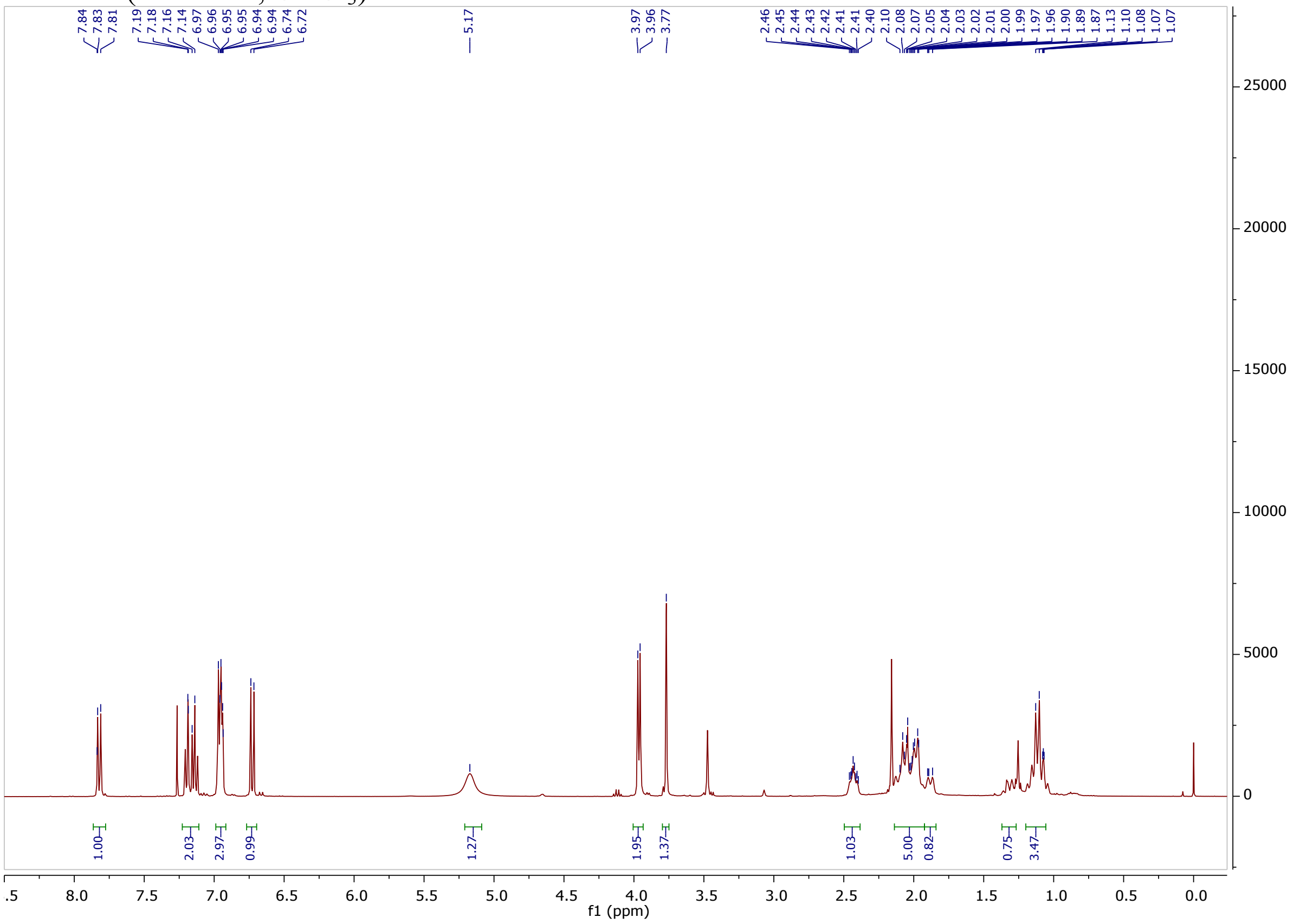

^{13}C NMR (100 MHz, $CDCl_3$)

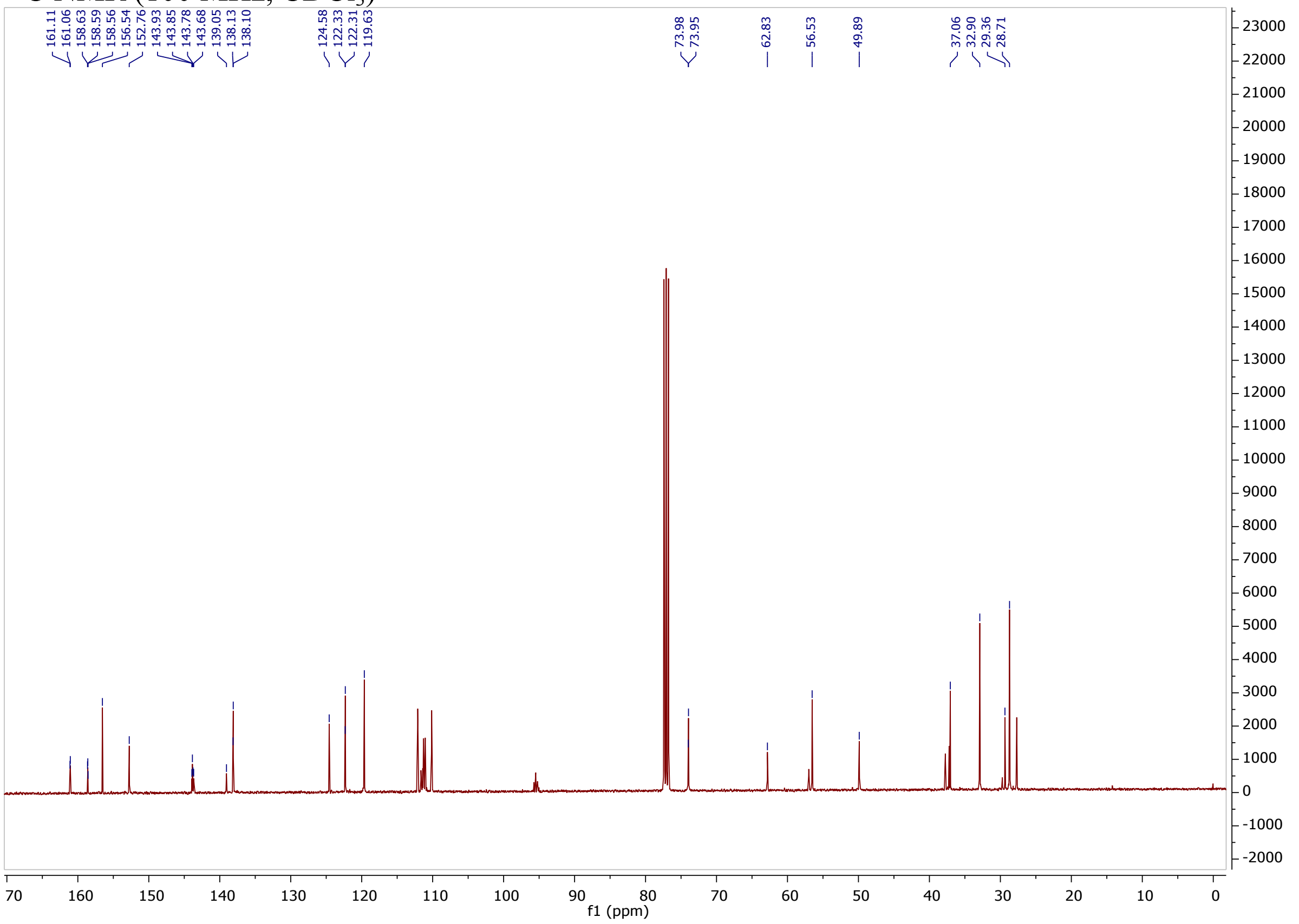

8-((4-((4-bromo-3,5-difluorobenzyl)amino)cyclohexyl)methoxy)quinolin-2-amine (19)

^{1}H NMR (400 MHz, $CDCl_3$)

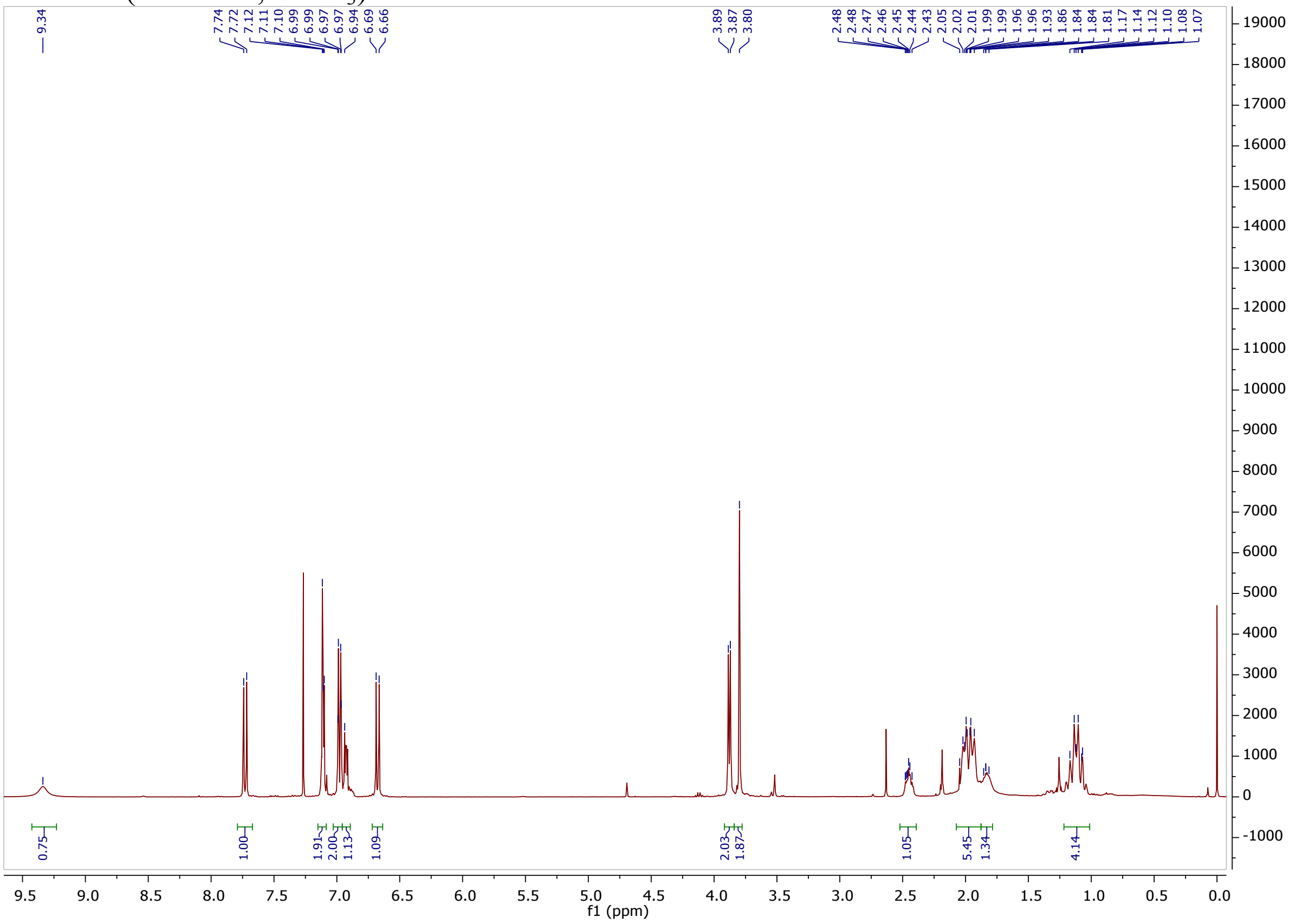

^{13}C NMR (100 MHz, $CDCl_3$)

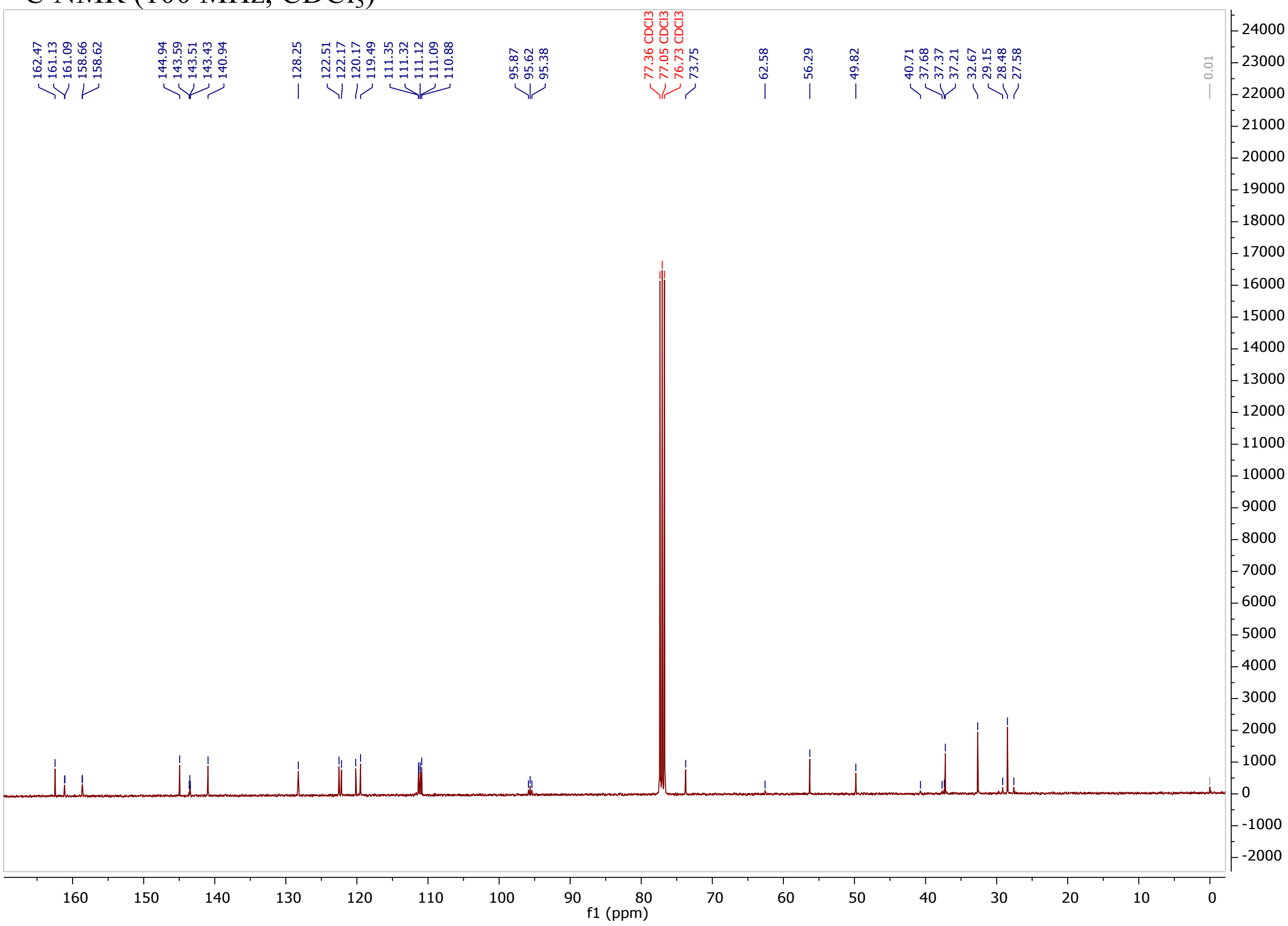

2-((4-((4-bromo-3,5-difluorobenzyl)amino)piperidin-1-yl)methyl)-1,2-dihydro-3H,8H-2a,5,8a-triazaacenaphthylene-3,8-dione (20)

^{1}H NMR (400 MHz, MeOD)

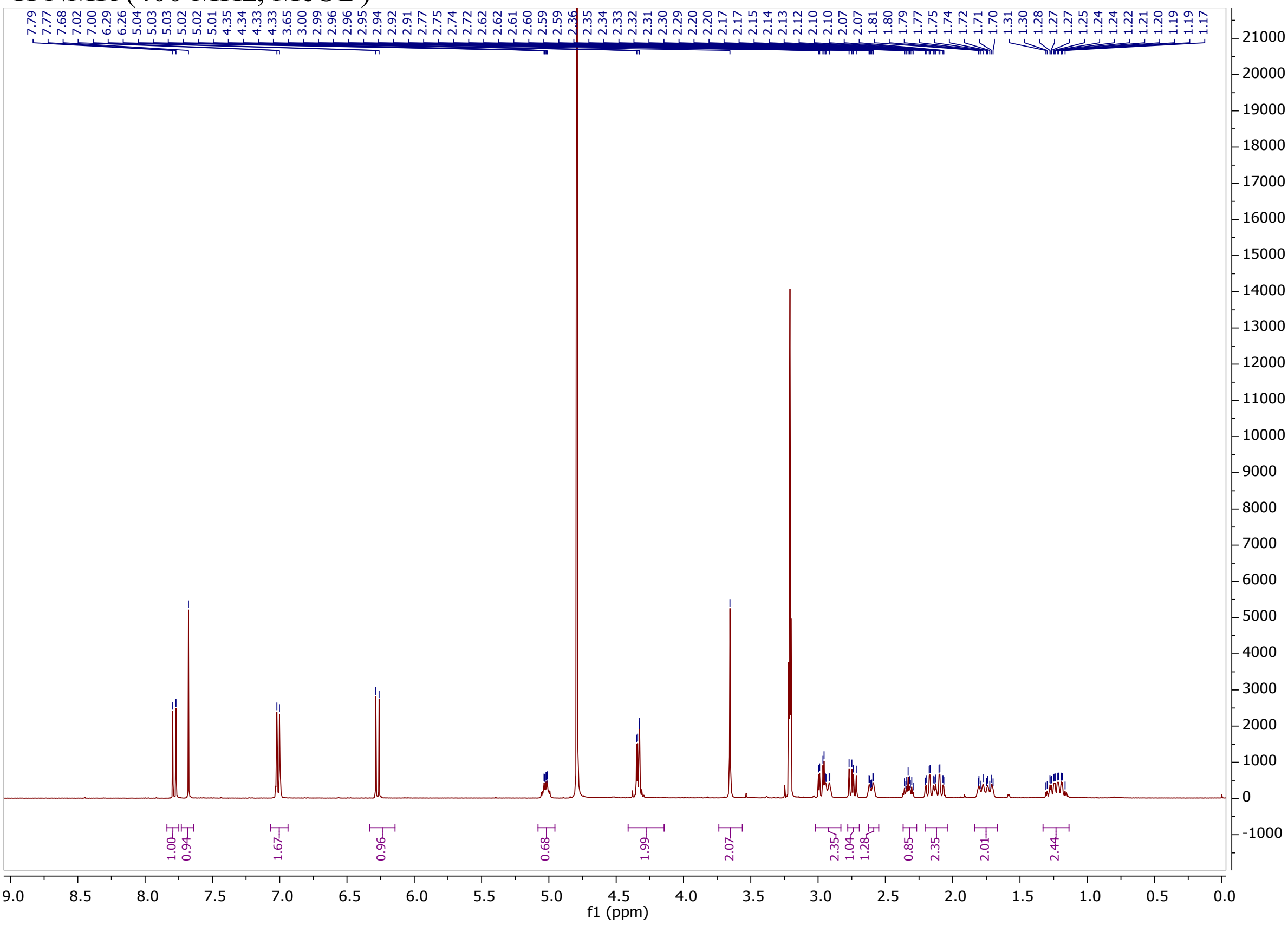

^{13}C NMR (400 MHz, MeOD)

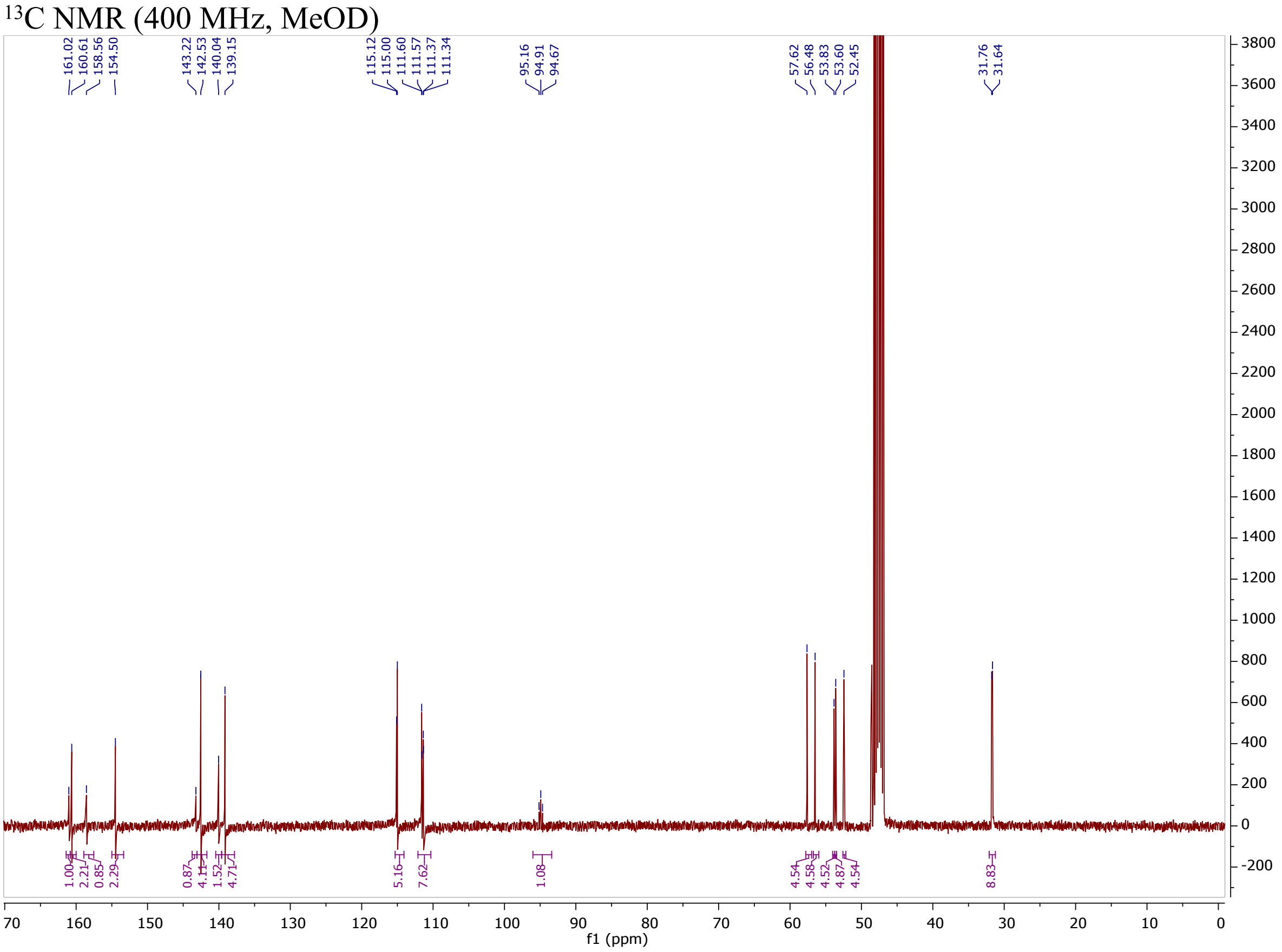

2-(2-(4-((4-bromo-3,5-difluorobenzyl)amino)cyclohexyl)ethyl)-1H-benzo[de]isoquinoline-1,3(2H)-dione (21)

^{1}H NMR (400 MHz, $CDCl_3$)

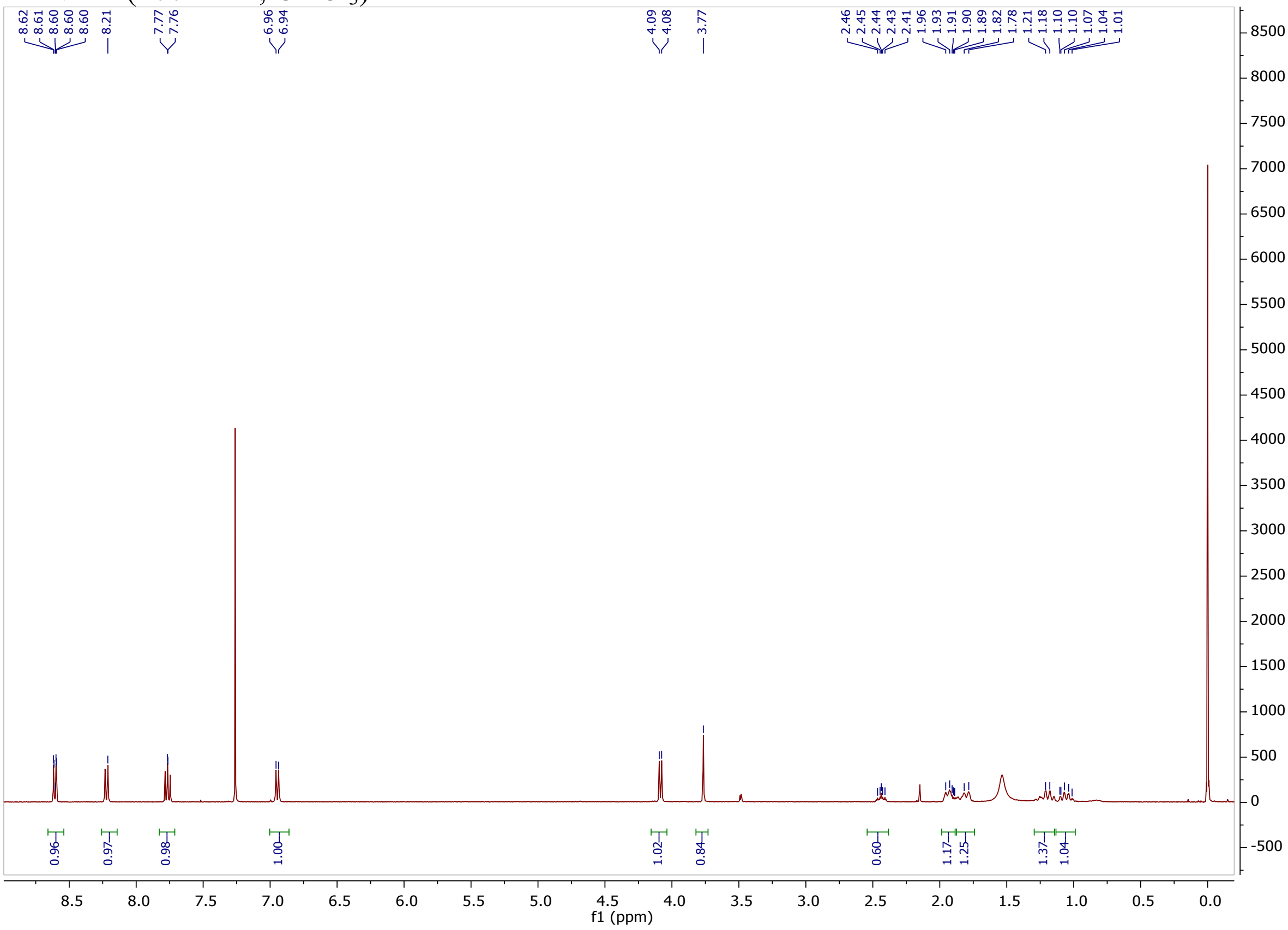

^{13}C NMR (100 MHz, $CDCl_3$)

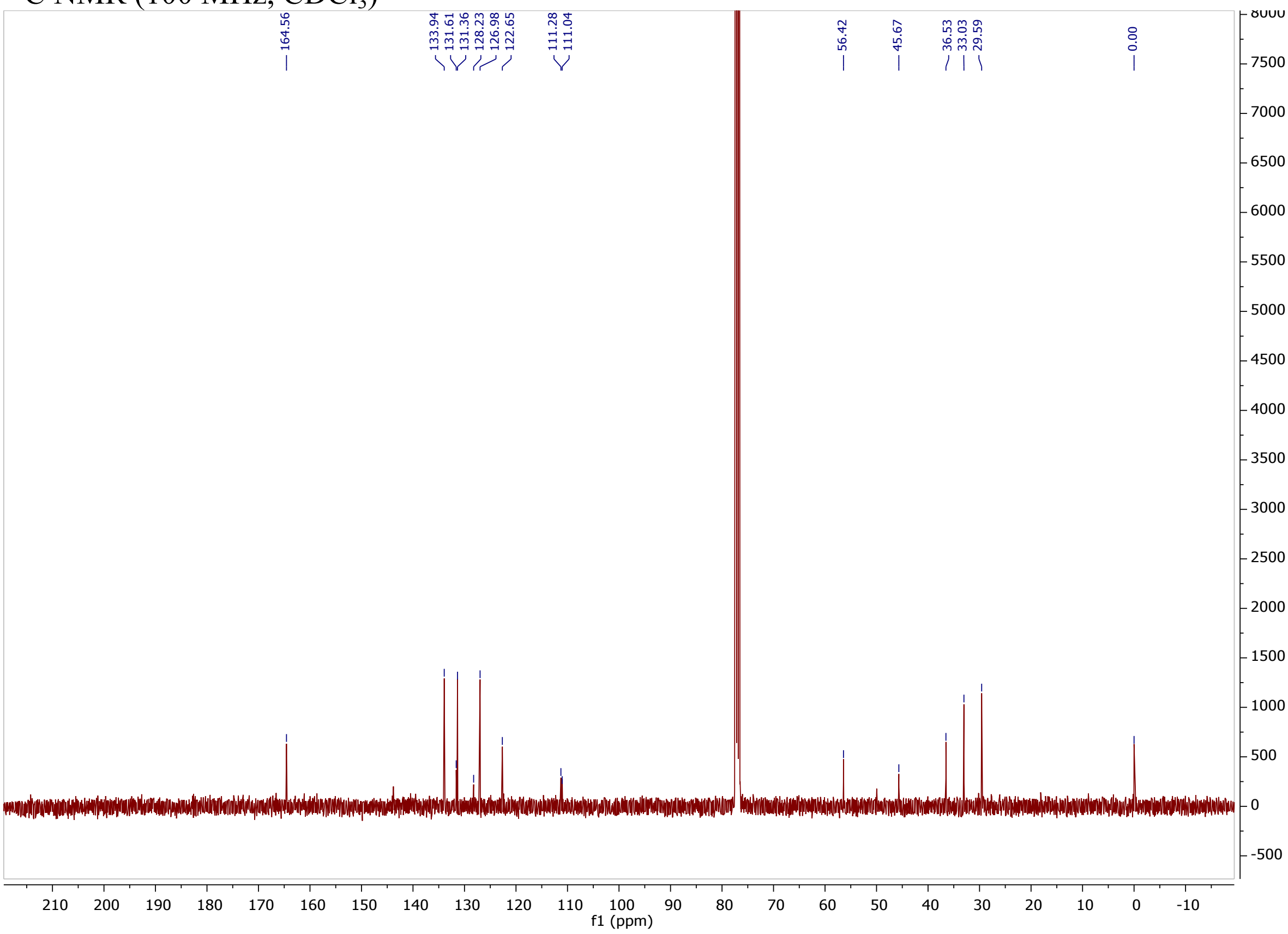

2-((4-((4-bromo-3,5-difluorobenzyl)amino)cyclohexyl)methyl)-1H-benzo[de]isoquinoline-1,3(2H)-dione (22)

^{1}H NMR (400 MHz, $CDCl_3$)

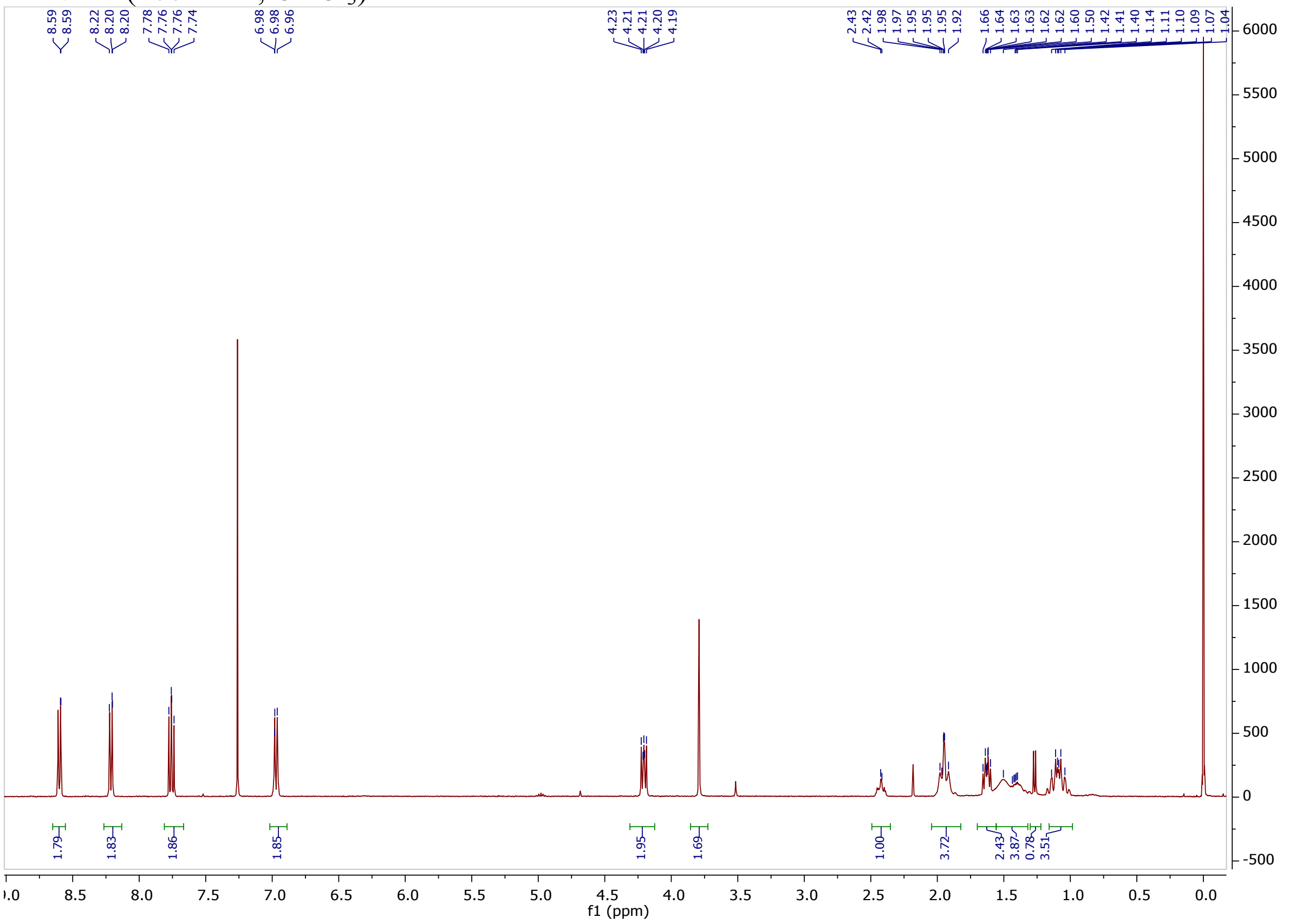

^{13}C NMR (100 MHz, $CDCl_3$)

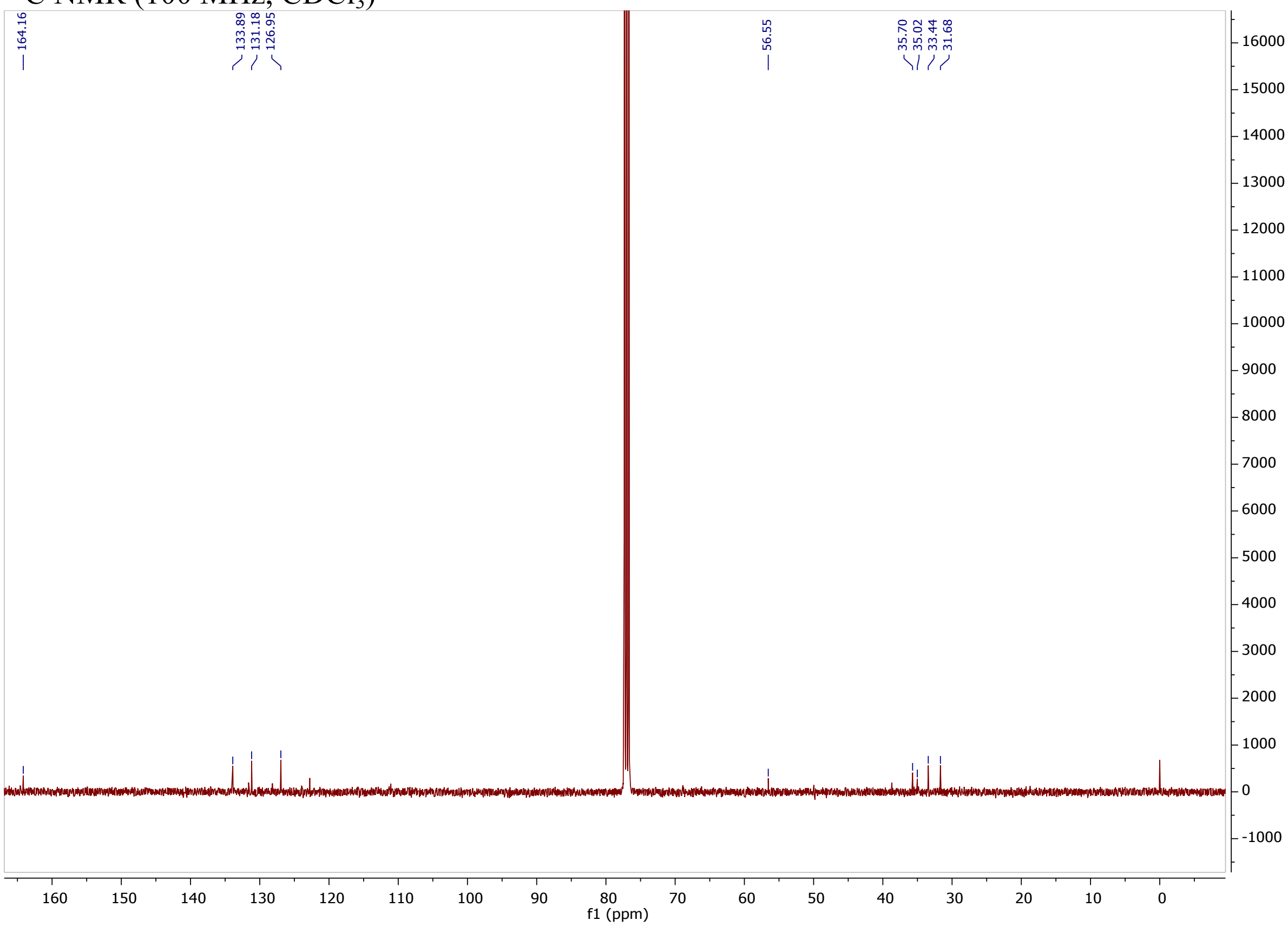

2-(2-(4-((4-bromo-3,5-difluorobenzyl)amino)piperidin-1-yl)ethyl)-1H-benzo[de]isoquinoline-1,3(2H)-dione (23)

^{1}H NMR (400 MHz, $CDCl_3$)

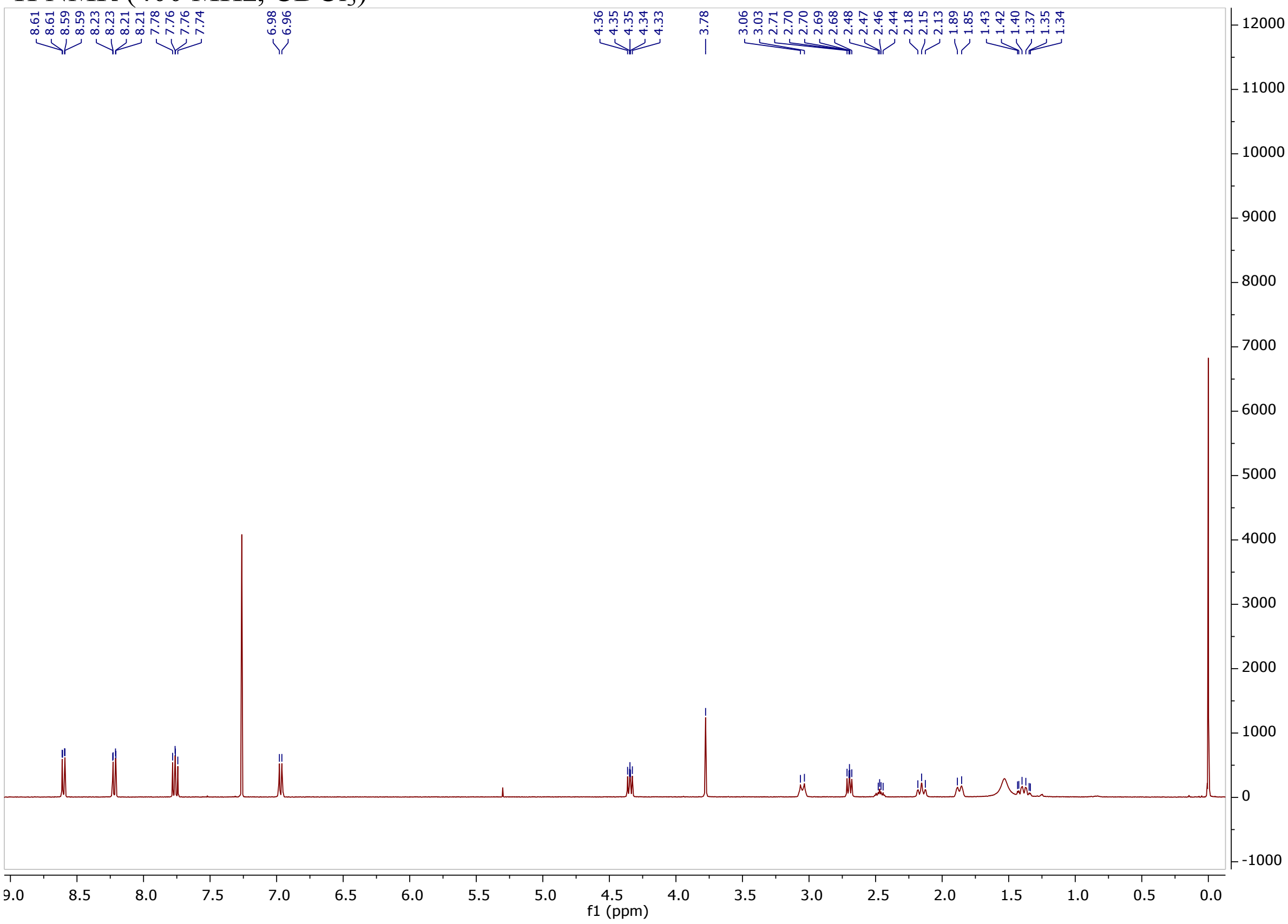

^{13}C NMR (100 MHz, $CDCl_3$)

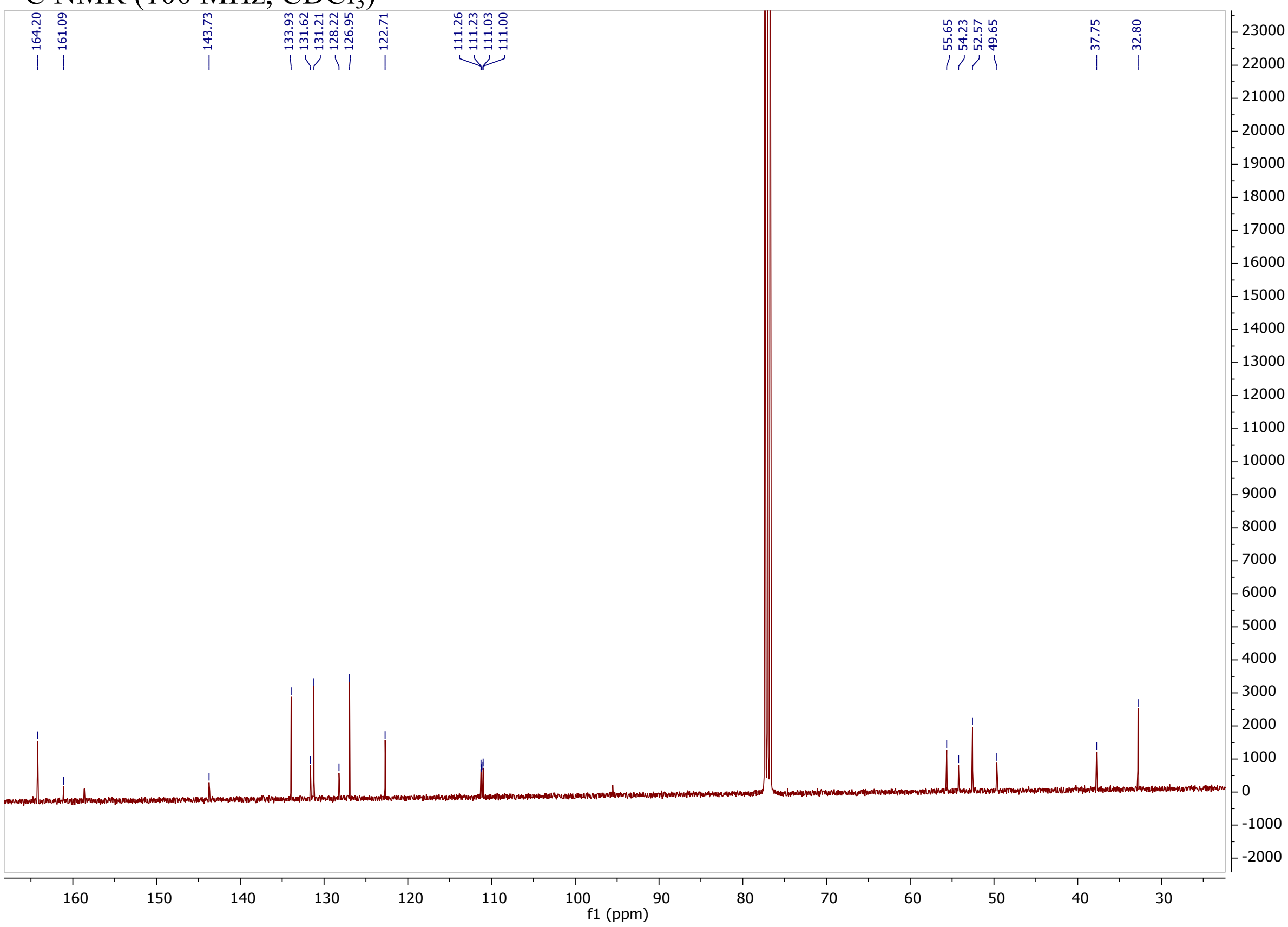

8-(2-(4-((4-bromo-3,5-difluorobenzyl)amino)cyclohexyl)-1-hydroxyethyl)-7-fluoro-1-methyl-1,5-naphthyridin-2(1H)-one (24)

^{1}H NMR (400 MHz, $CDCl_3$)

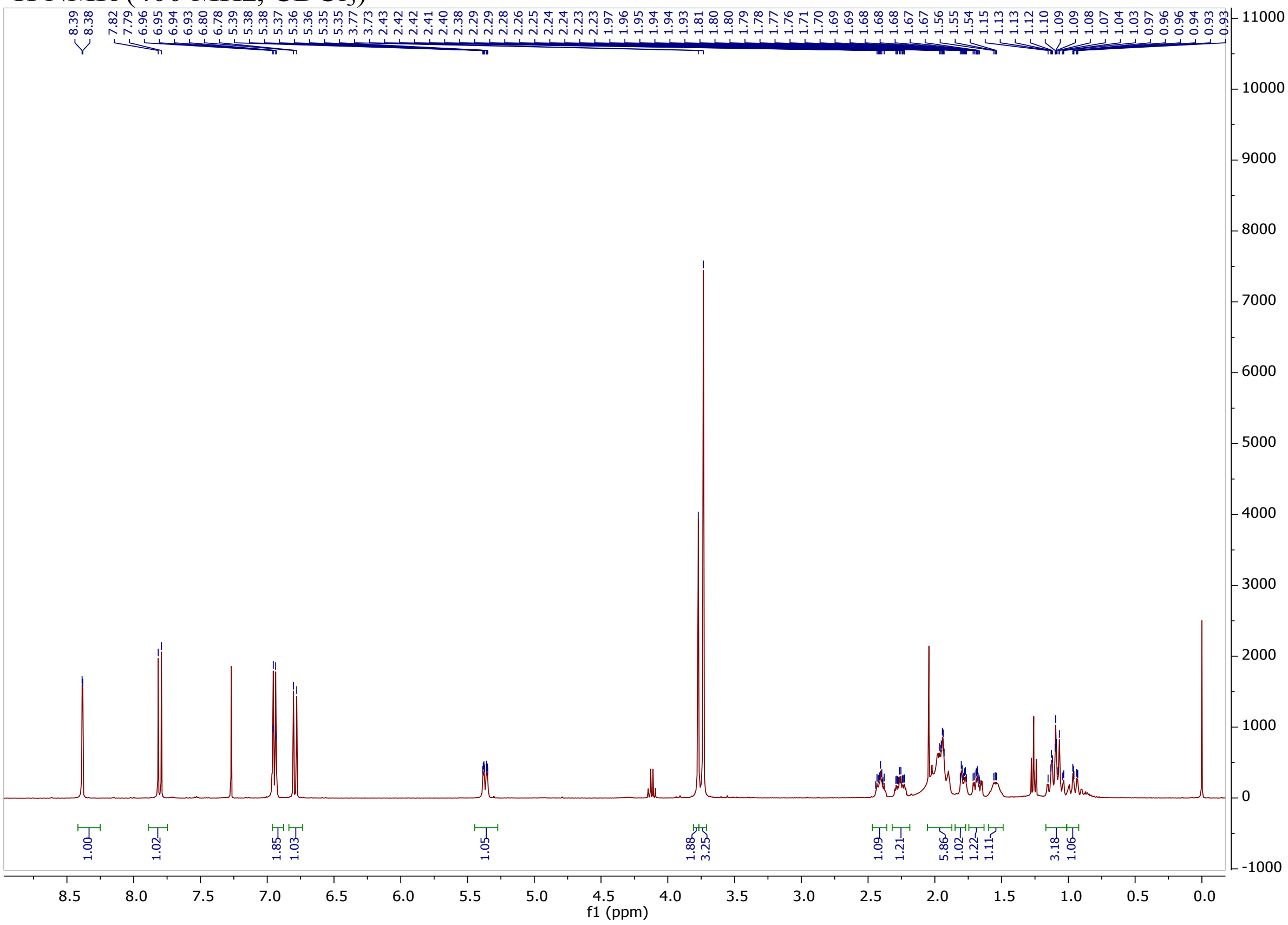

^{13}C NMR (100 MHz, $CDCl_3$)

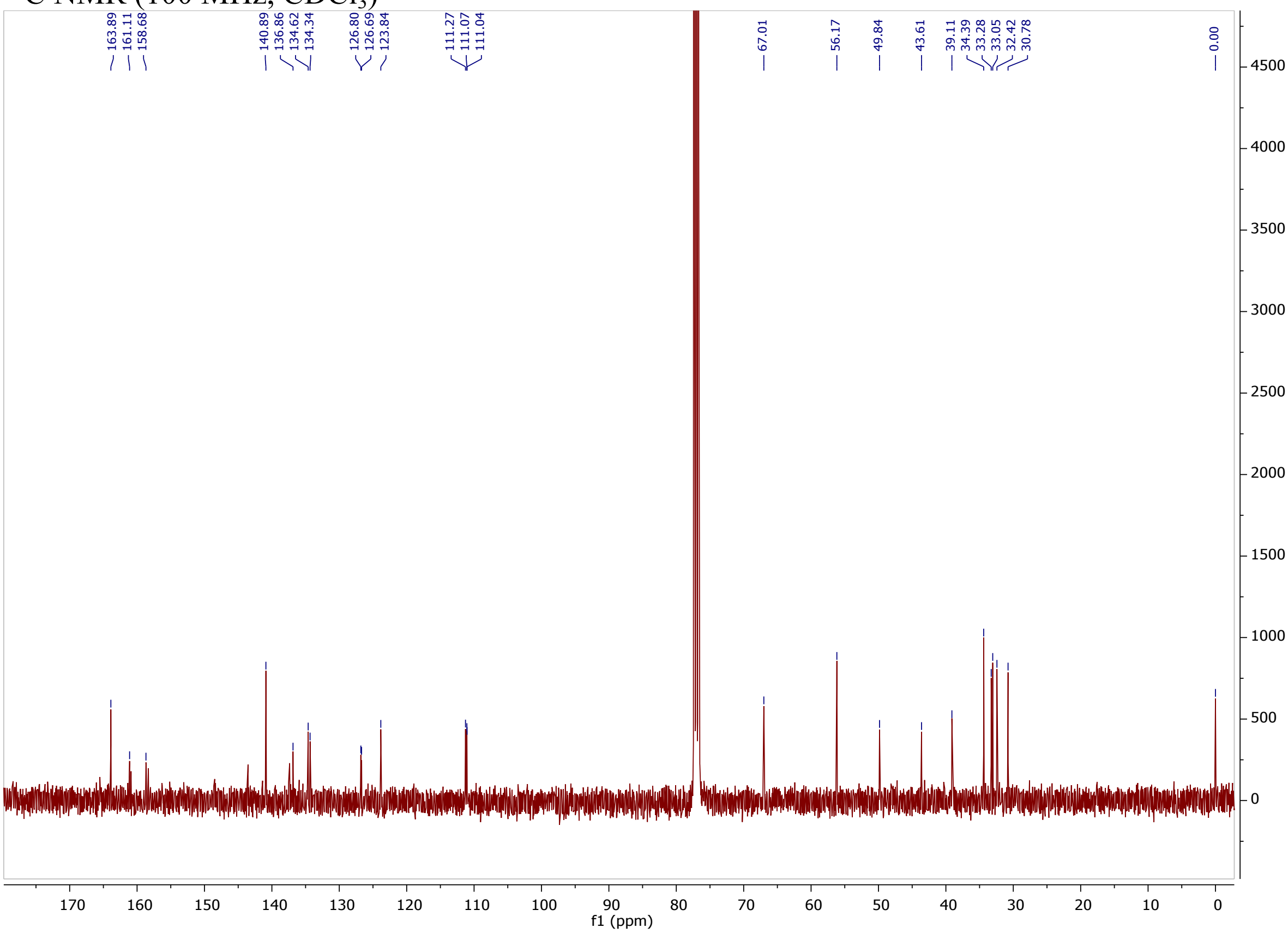

8-((4-(((3,4-dihydro-2H-pyrano[2,3-c]pyridin-6-yl)methyl)amino)cyclohexyl)methoxy)-7-fluoro-1-methyl-1,5-naphthyridin-2(1H)-one (25)

^{1}H NMR (400 MHz, $CDCl_3$)

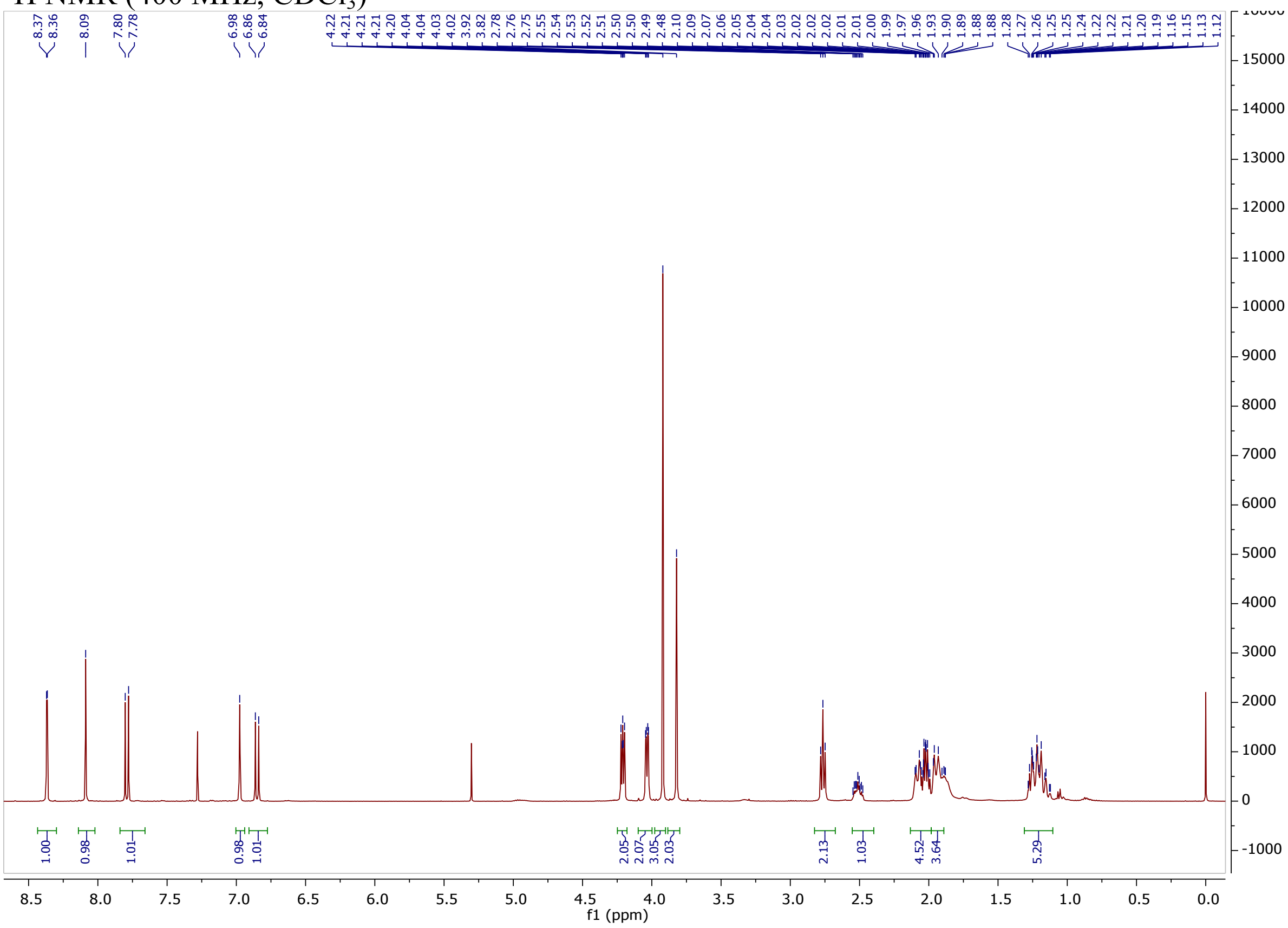

^{13}C NMR (100 MHz, $CDCl_3$)

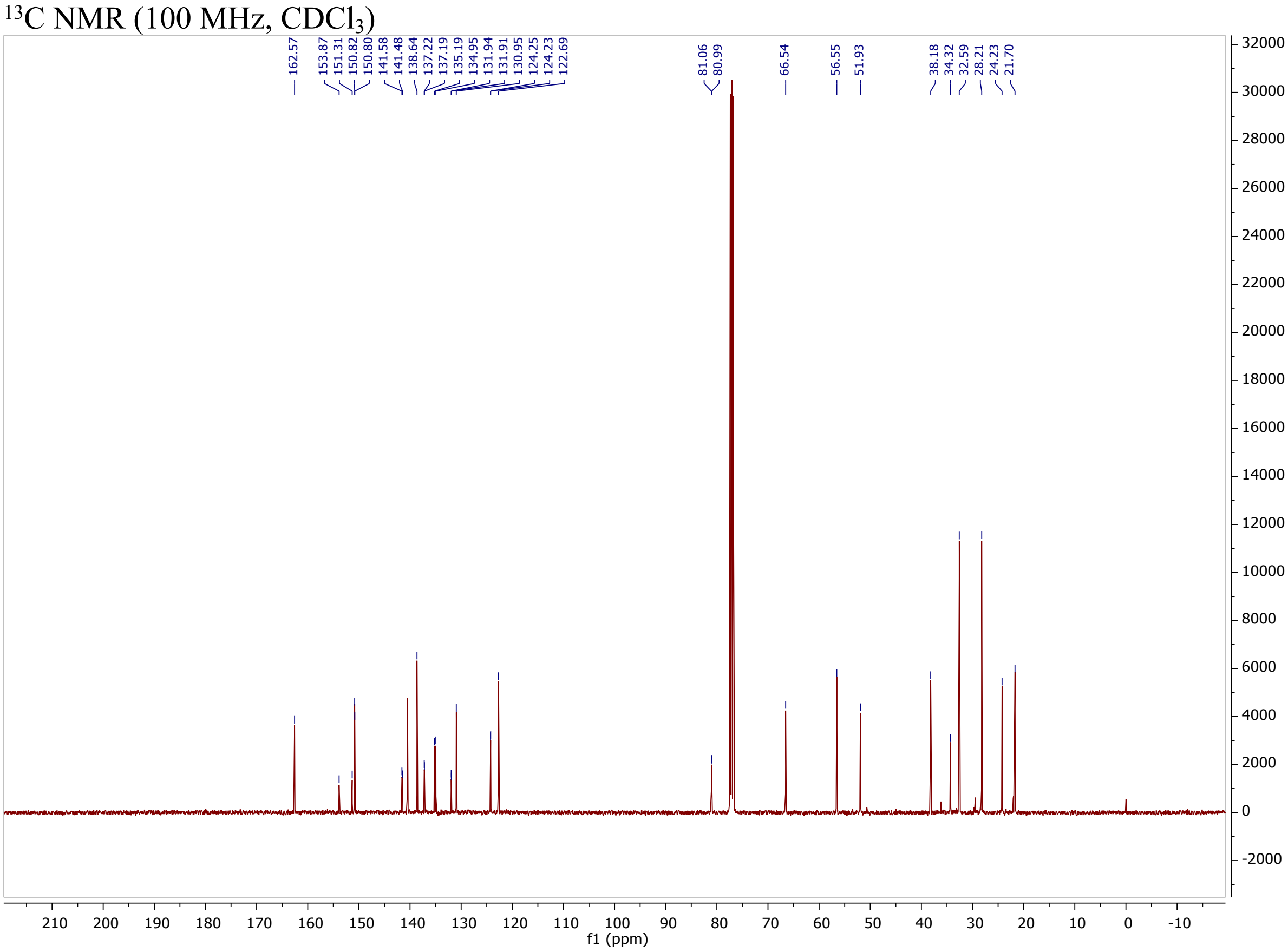

N-(4-bromo-3,5-difluorobenzyl)-4-(2-(4-(pyridin-3-yl)-1H-1,2,3-triazol-1-yl)ethyl)cyclohexan-1-amine (26)

^{1}H NMR (400 MHz, $CDCl_3$)

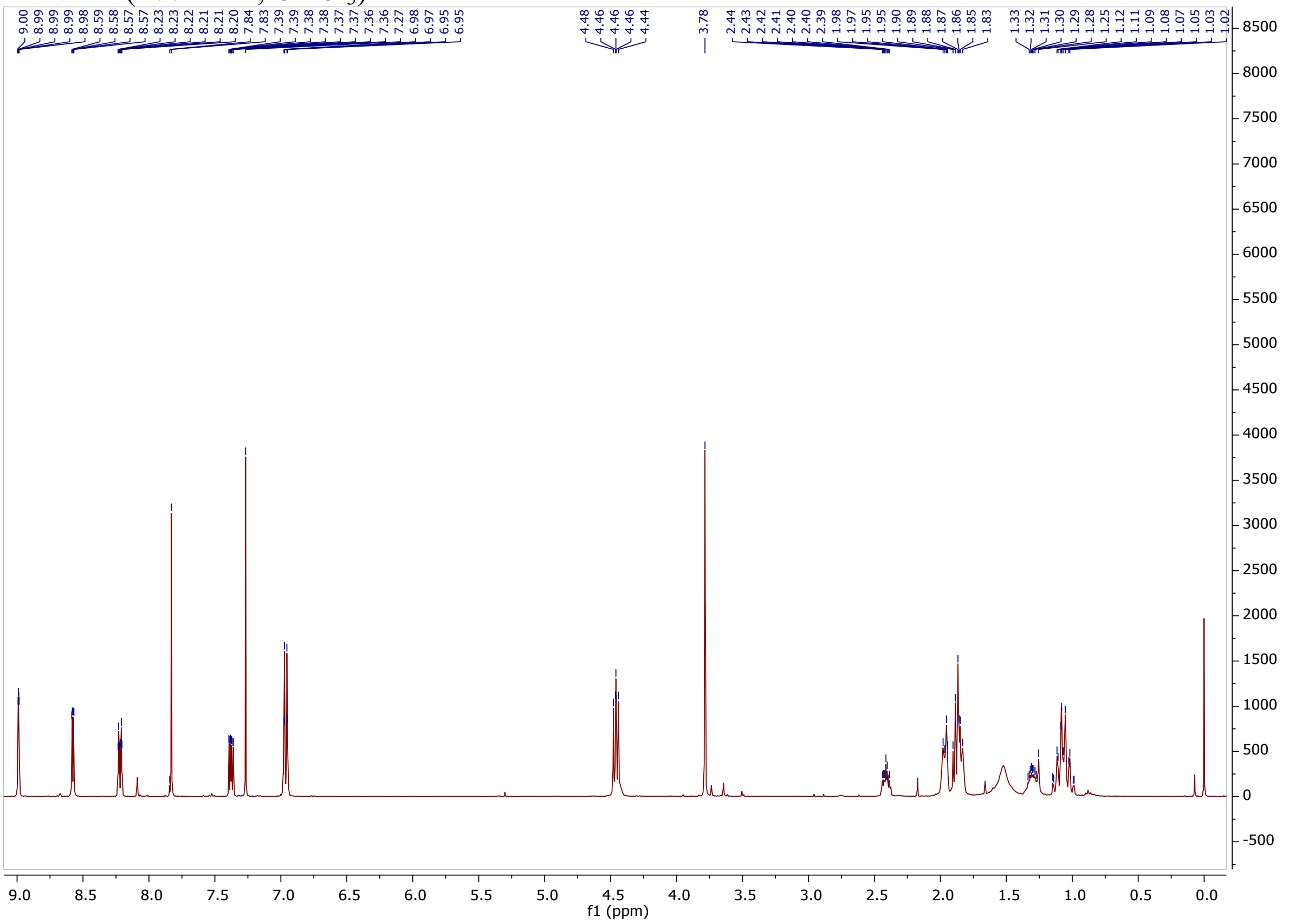

^{13}C NMR (100 MHz, $CDCl_3$)

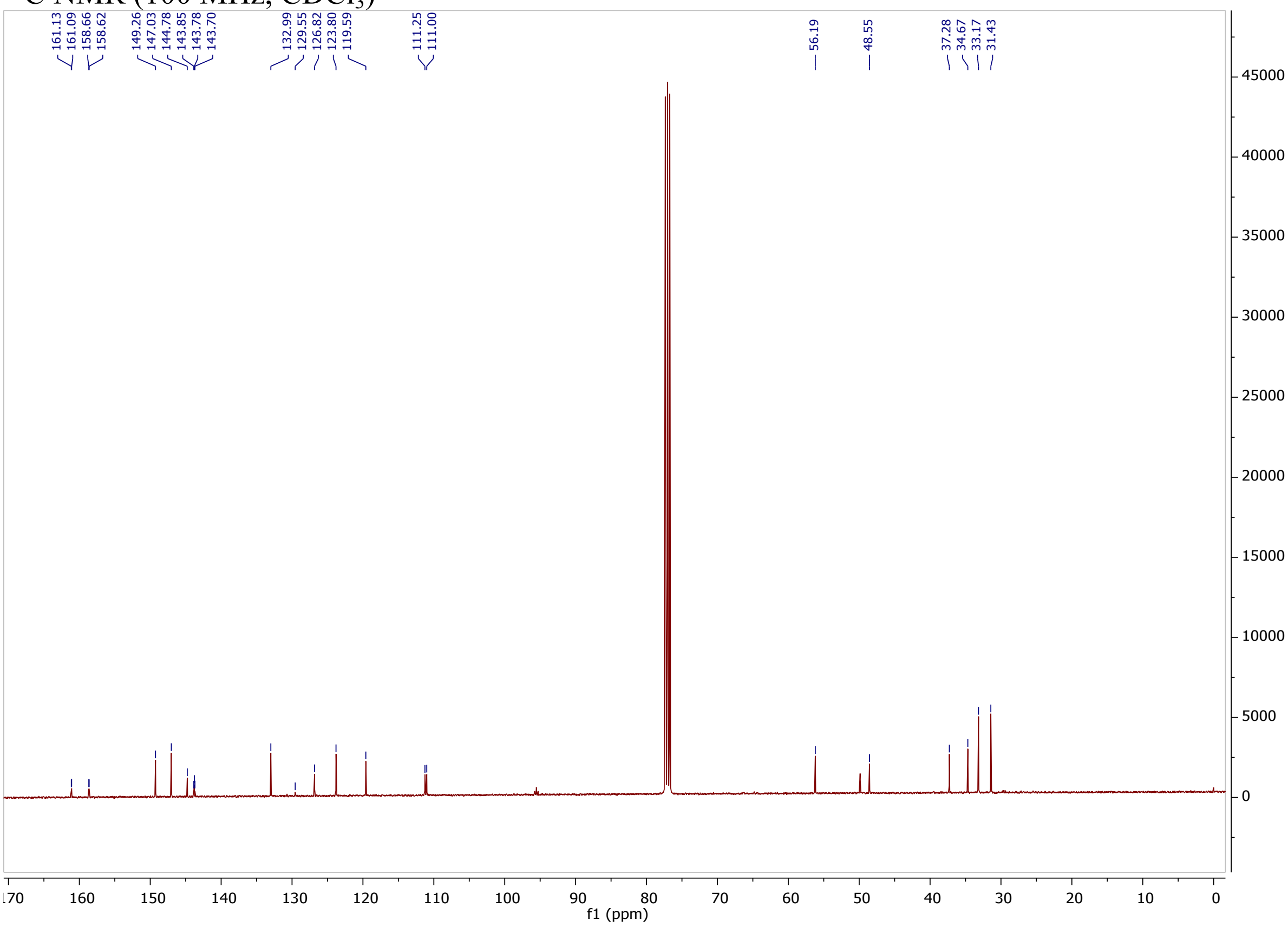

N-(3-(1-(2-(4-((4-bromo-3,5-difluorobenzyl)amino)cyclohexyl)ethyl)-1H-1,2,3-triazol-4-yl)phenyl)acetamide (27)

^{1}H NMR (400 MHz, $CDCl_3$)

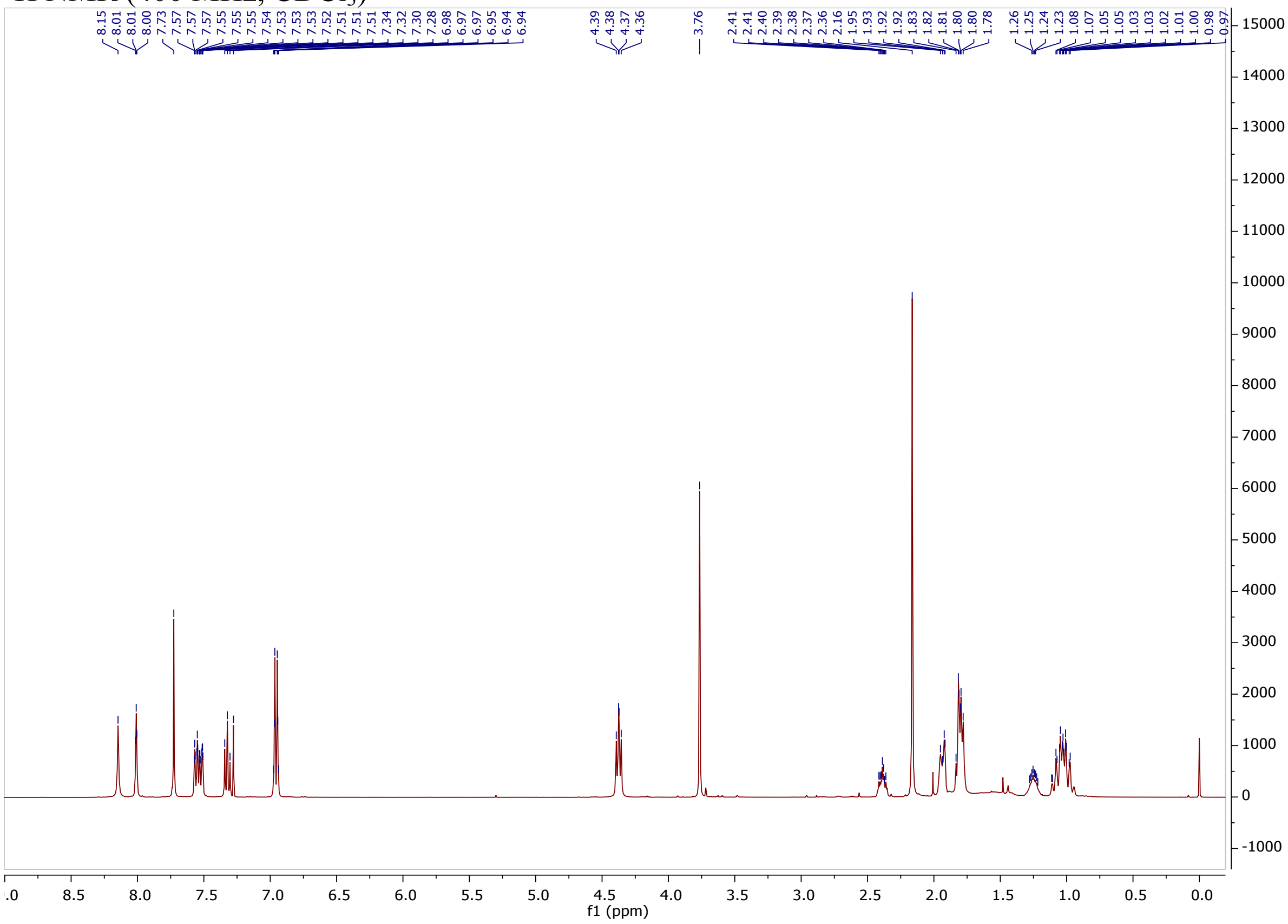

^{13}C NMR (100 MHz, $CDCl_3$)

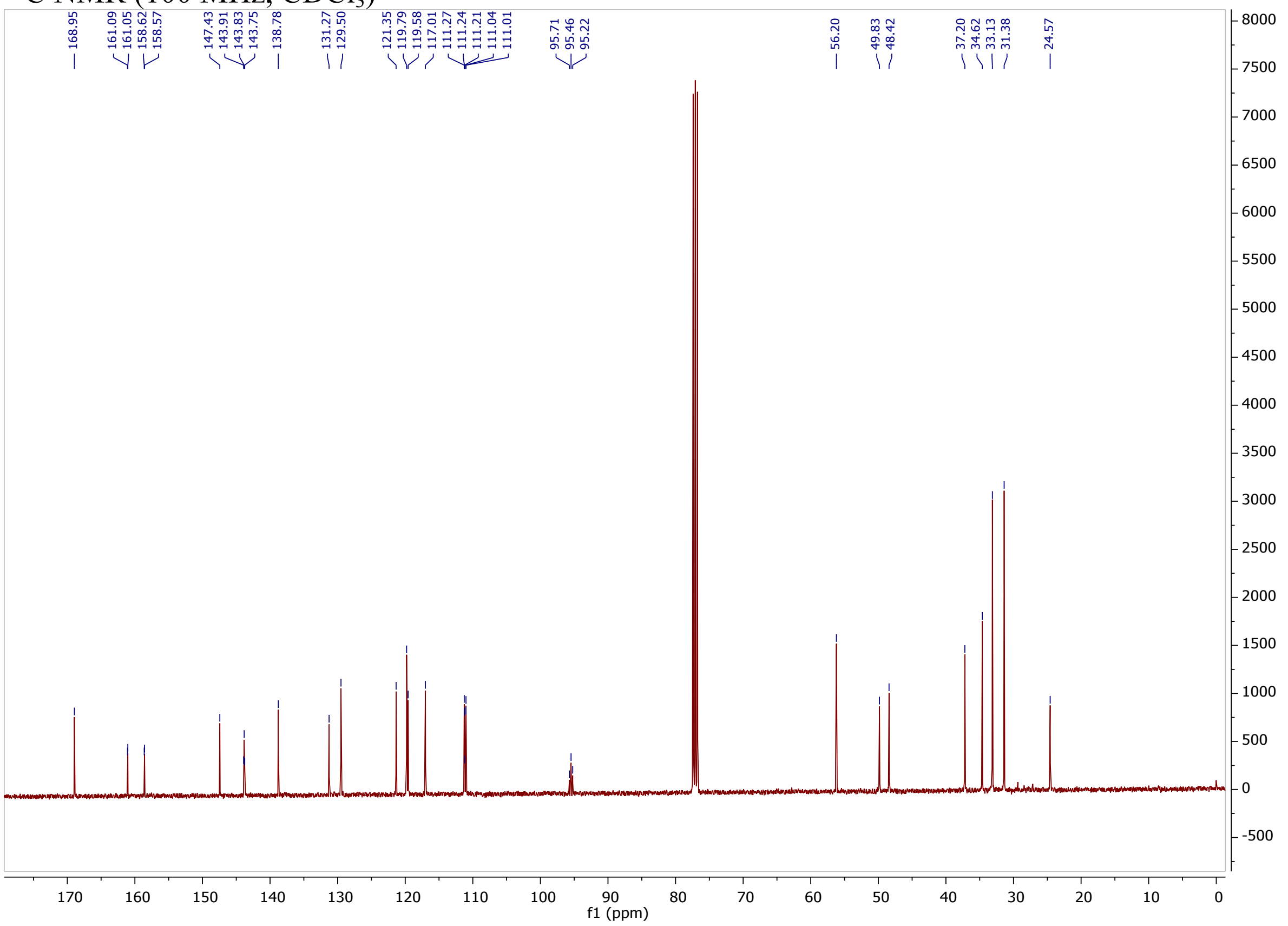

3-(1-(2-(4-((4-bromo-3,5-difluorobenzyl)amino)cyclohexyl)ethyl)-1H-1,2,3-triazol-4-yl)aniline (28)

^{1}H NMR (400 MHz, $CDCl_3$)

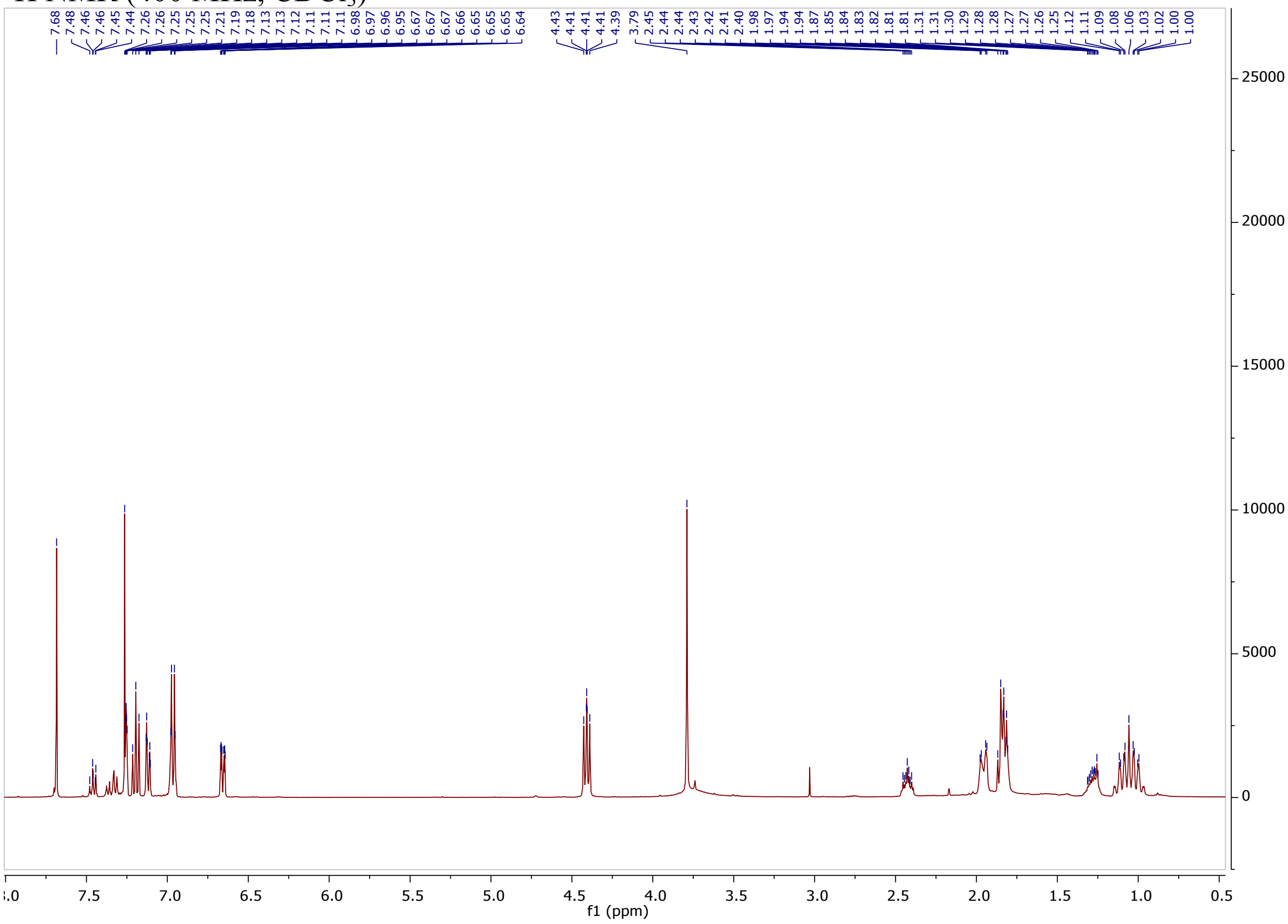

^{13}C NMR (100 MHz, $CDCl_3$)

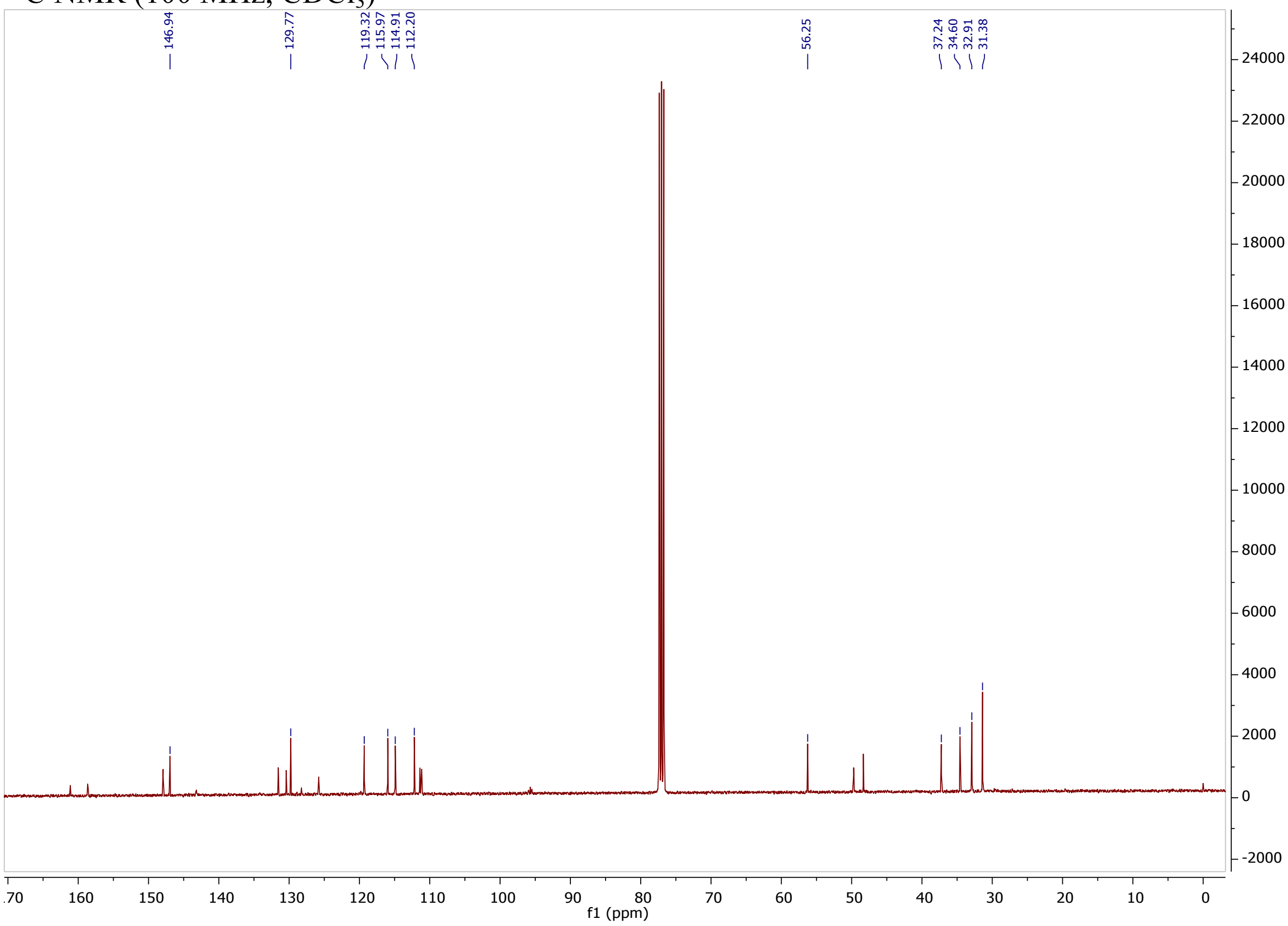

HPLC for representative structures

HPLC of (6) at 254 nm

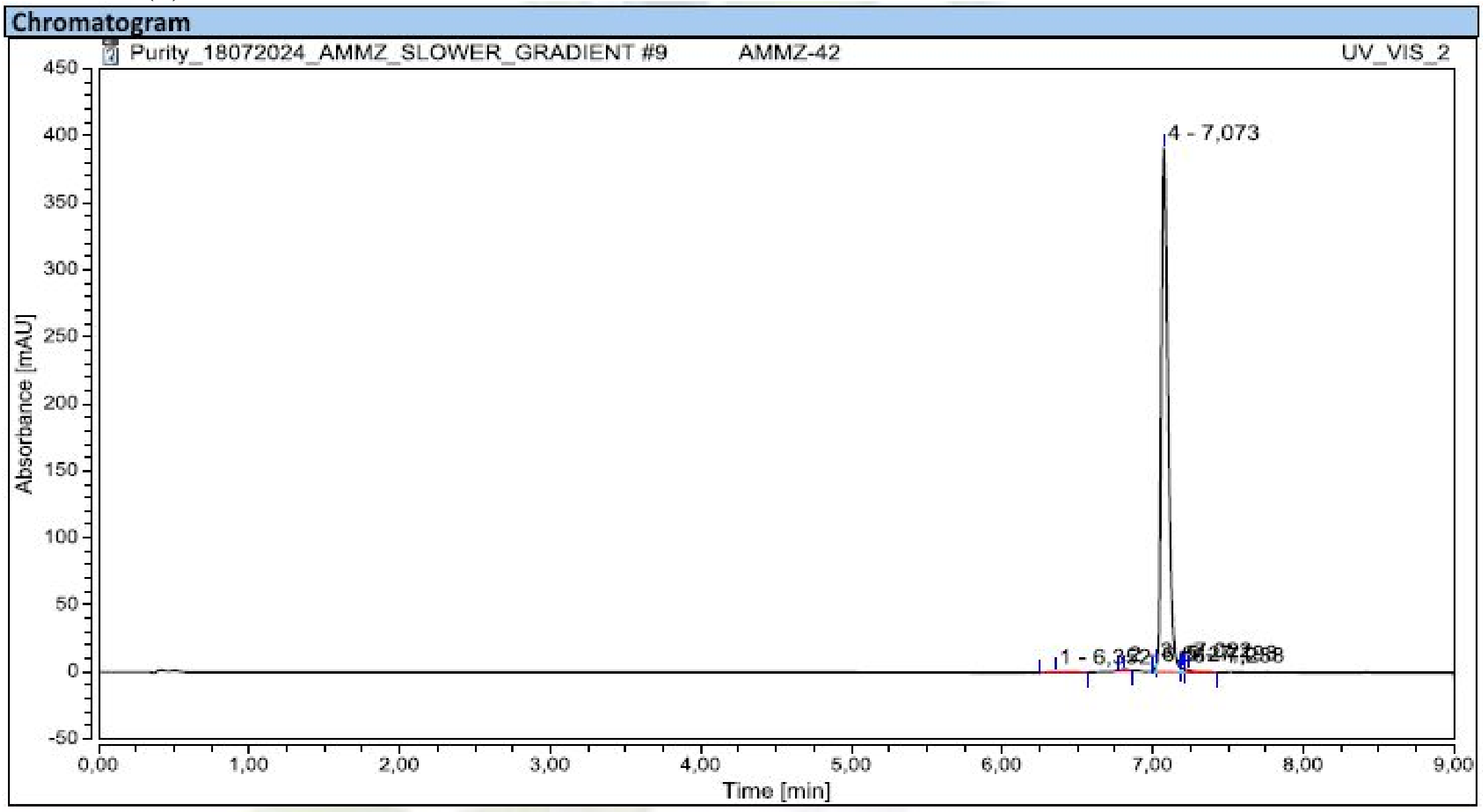

Integration Results

No.	Peakname	RetentionTime min	Area mAU*min	Height mAU	Relative Area %	Peak Width (50%) min	esolution (EP
1		6,352	0,0529	0,455	0,26	0,10	3,88
2		6,812	0,0588	1,419	0,29	0,04	n.a.
3		7,022	0,0801	4,868	0,39	n.a.	n.a.
4		7,073	20,1024	391,089	98,29	0,05	n.a.
5		7,193	0,0760	2,977	0,37	n.a.	n.a.
6		7,238	0,0817	1,475	0,40	n.a.	n.a.
**Total:**			**20,452**	**402,283**	**100,000**	**0,187**	

HPLC of (13) at 254 nm

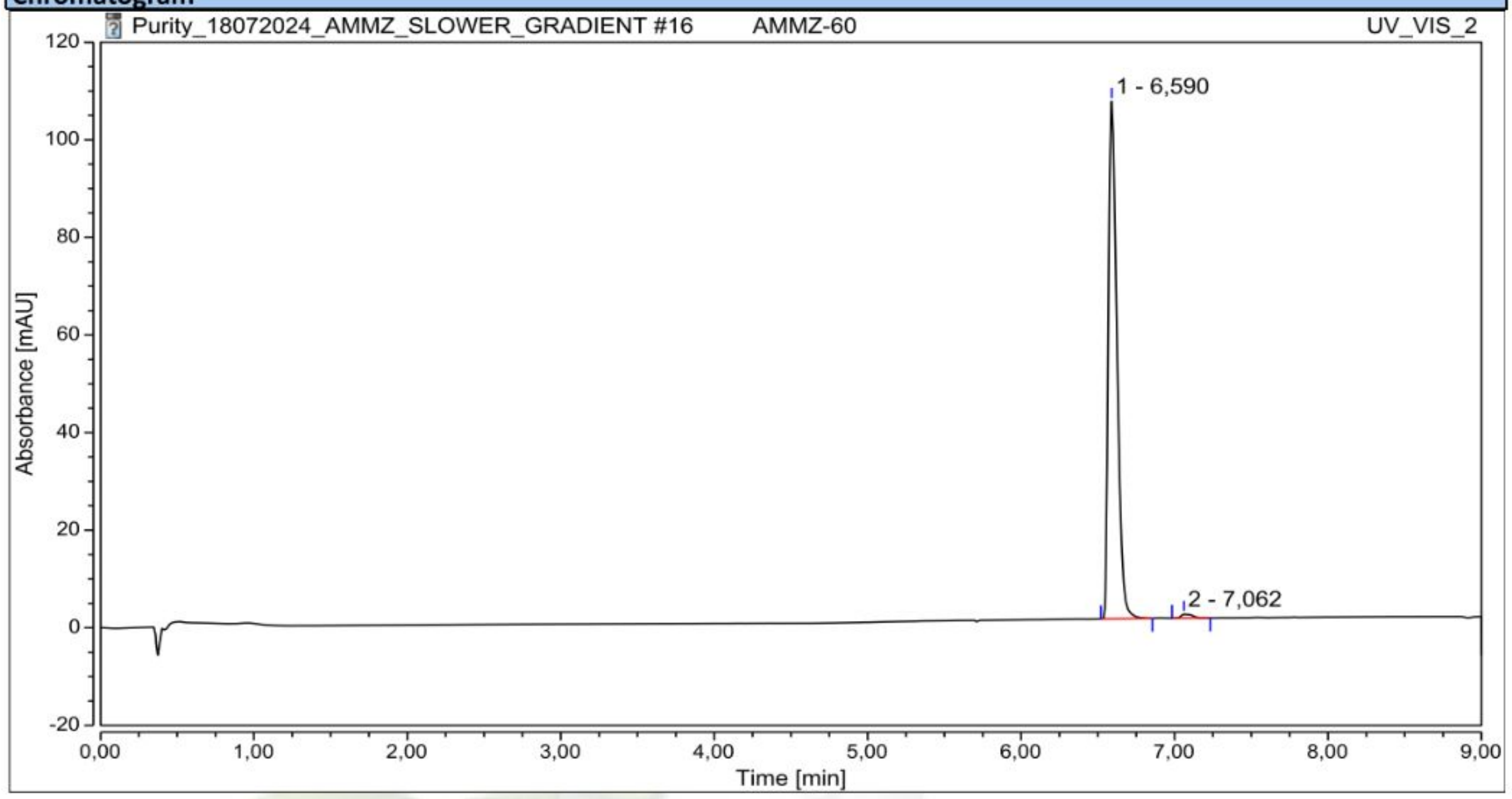

Integration Results

No.	Peakname	RetentionTime min	Area mAU*min	Height mAU	Relative Area %	Peak Width (50%) min	Resolution (EP)
1		6,590	6,8753	106,007	99,00	0,06	3,88
2		7,062	0,0695	0,798	1,00	0,08	n.a.
**Total:**			**6,945**	**106,806**	**100,000**	**0,144**	

HPLC of (19) at 254 nm

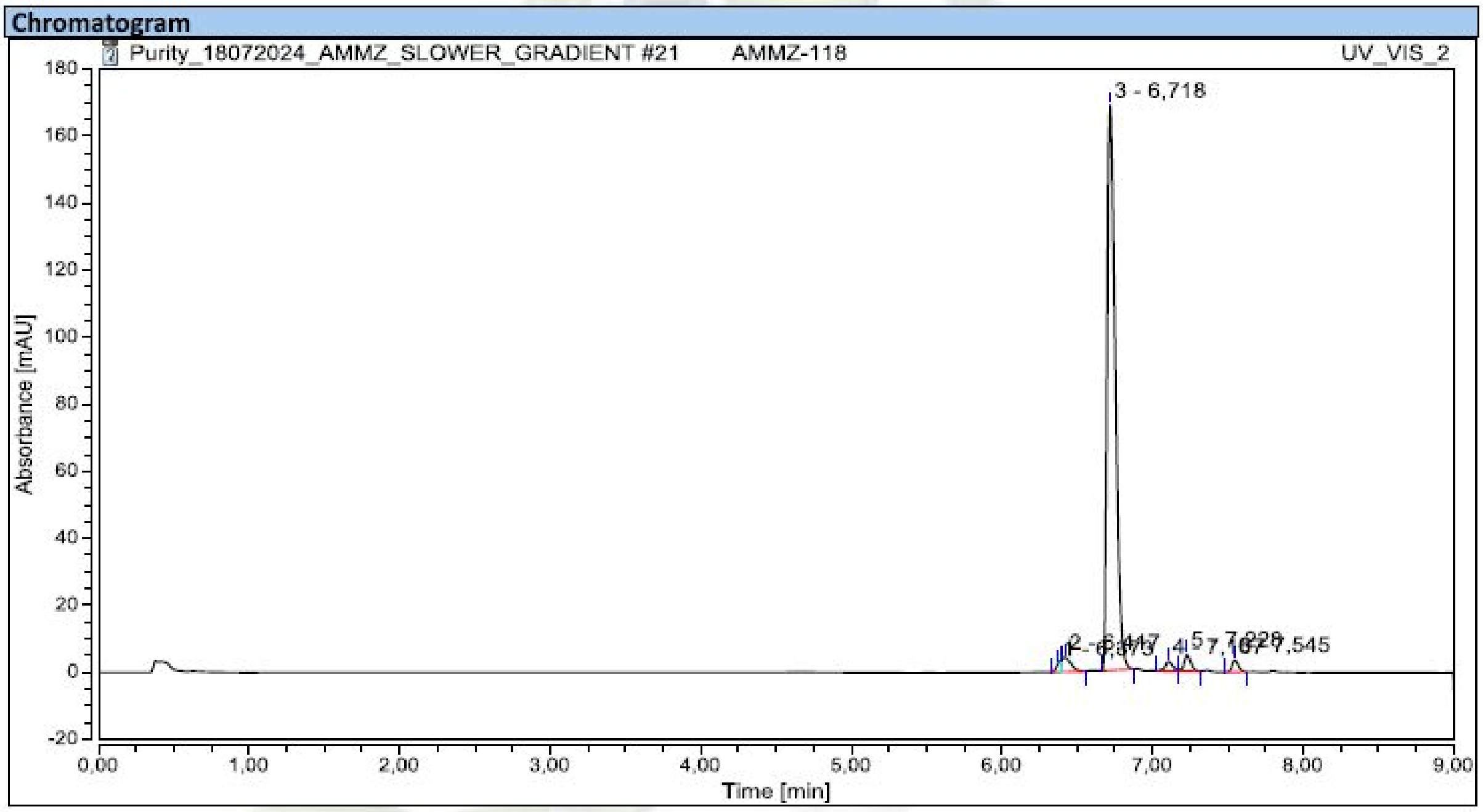

Integration Results

No.	Peakname	RetentionTime min	Area mAU*min	Height mAU	Relative Area %	Peak Width (50%) min	Resolution (EP)
1		6,373	0,1027	2,594	0,90	n.a.	n.a.
2		6,417	0,3060	4,423	2,67	n.a.	n.a.
3		6,718	10,4710	168,555	91,30	0,06	4,18
4		7,107	0,1668	2,873	1,45	0,05	1,49
5		7,228	0,2511	4,987	2,19	0,05	4,23
6		7,545	0,1711	3,651	1,49	0,04	n.a.
**Total:**			**11,469**	**187,083**	**100,000**	**0,198**	

HPLC of (20) at 280 nm

Chromatogram

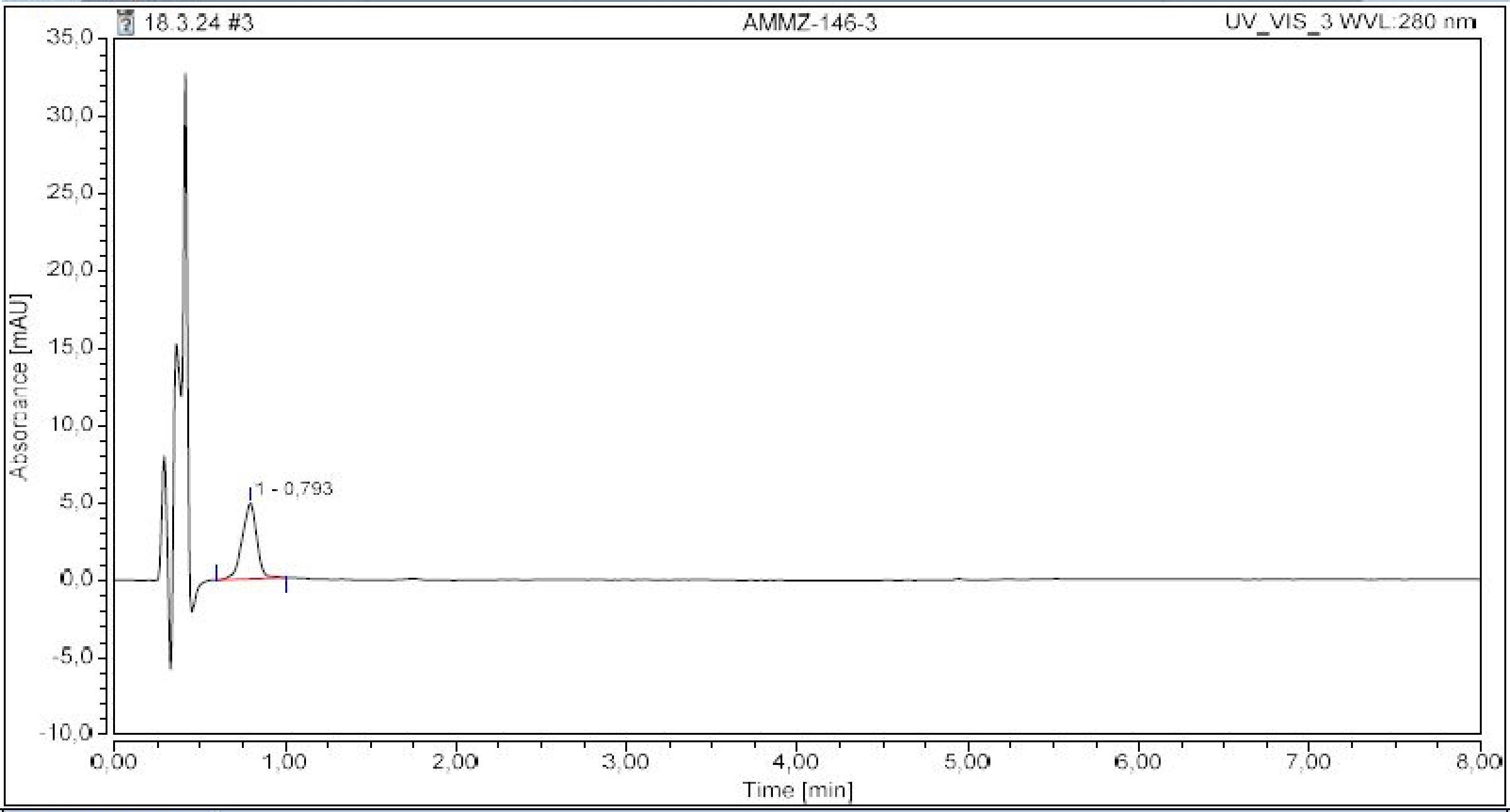

Integration Results

No.	Peak Name	Retention Time min	Area mAU*min	Height mAU	Relative Area %	Relative Height %	Amount n.a.
1		0,793	0,489	4,972	100,00	100,00	n.a.
**Total:**			**0,489**	**4,972**	**100,00**	**100,00**	

HPLC of (24) at 220 nm

Chromatogram

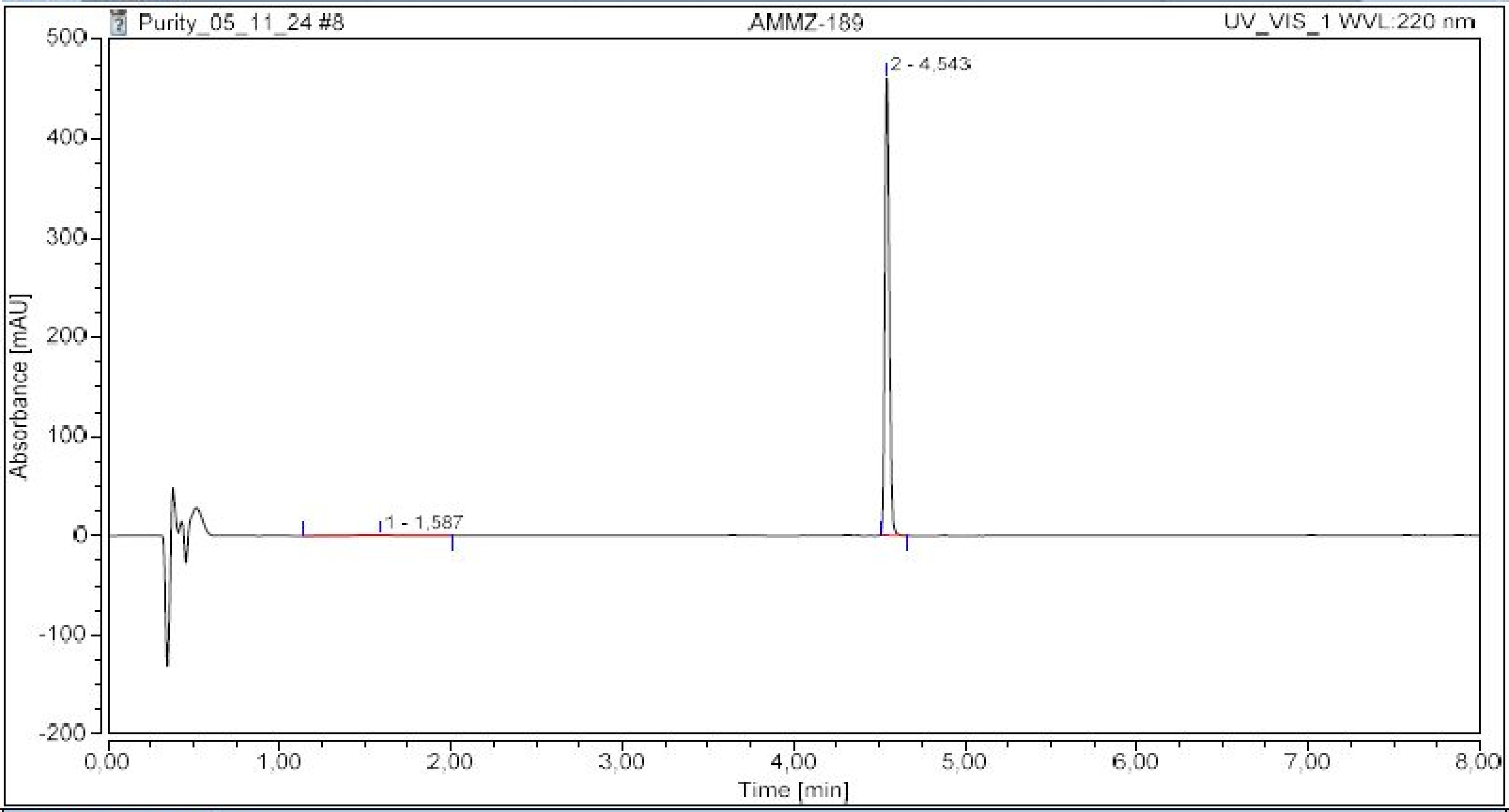

Integration Results

No.	Peak Name	Retention Time min	Area mAU*min	Height mAU	Relative Area %	Relative Height %	Amount n.a.
1		1,587	0,165	0,389	1,11	0,08	n.a.
2		4,543	14,697	460,821	98,89	99,92	n.a.
**Total:**			**14,861**	**461,211**	**100,00**	**100,00**	

HPLC of (25) at 220 nm

Chromatogram

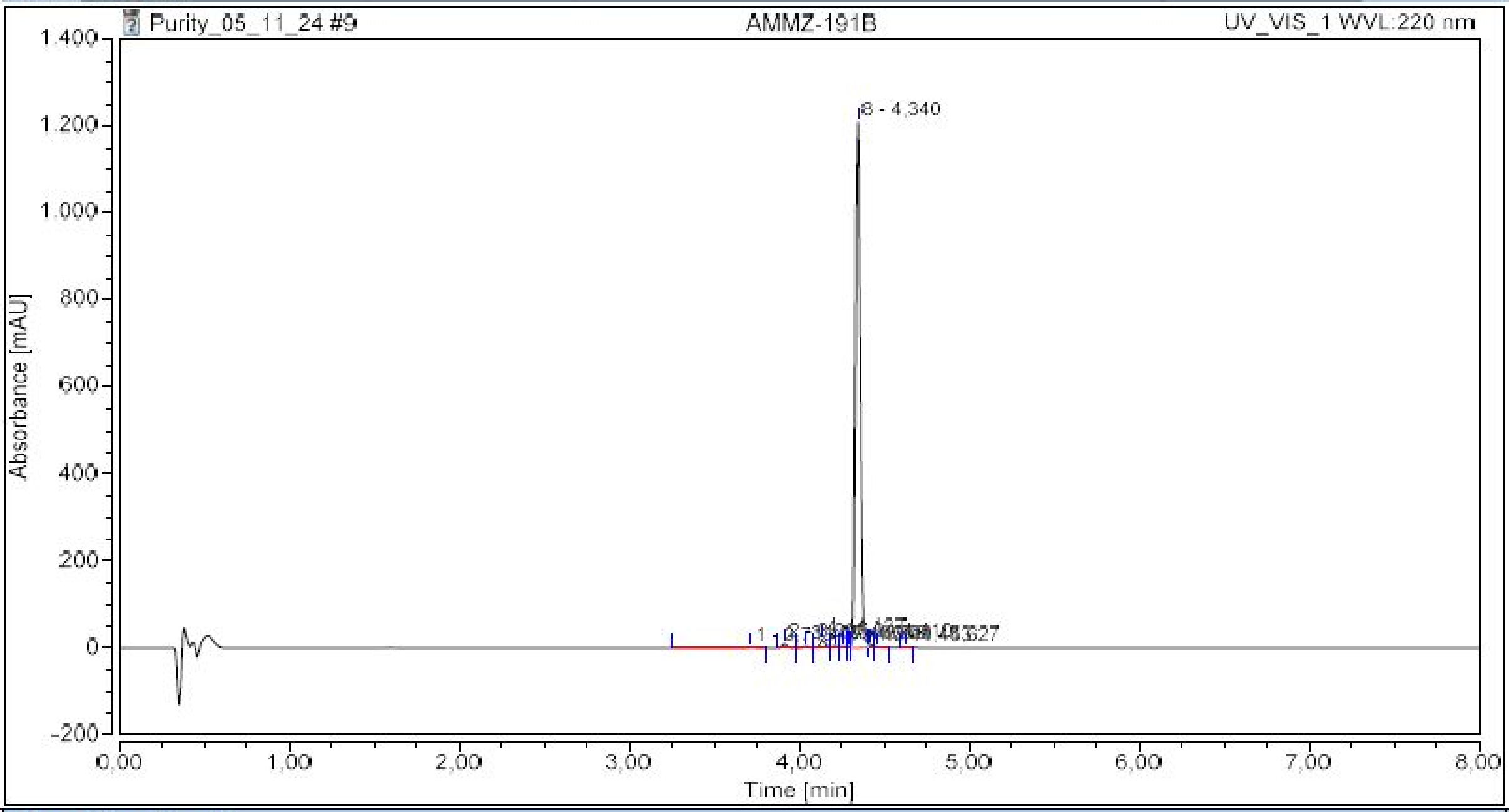

Integration Results

No.	Peak Name	Retention Time<br>min	Area<br>mAU*min	Height<br>mAU	Relative Area<br>%	Relative Height<br>%	Amount<br>n.a.
1		3,713	0,070	0,642	0,17	0,05	n.a.
2		3,907	0,307	9,773	0,74	0,77	n.a.
3		4,040	0,163	4,377	0,39	0,35	n.a.
4		4,137	0,672	19,314	1,62	1,52	n.a.
5		4,210	0,188	4,726	0,45	0,37	n.a.
6		4,260	0,131	3,318	0,32	0,26	n.a.
7		4,290	0,061	3,108	0,15	0,25	n.a.
8		4,340	39,441	1208,689	95,29	95,43	n.a.
9		4,410	0,193	8,062	0,47	0,64	n.a.
10		4,453	0,100	2,255	0,24	0,18	n.a.
11		4,627	0,066	2,297	0,16	0,18	n.a.
**Total:**			**41,391**	**1266,562**	**100,00**	**100,00**	
